# Genetic liability to childhood Kawasaki disease and adult cardiovascular outcomes

**DOI:** 10.3389/fcvm.2026.1848320

**Published:** 2026-07-23

**Authors:** Mengzhuo Wang, Sha Lin, Xiaoliang Liu, Jinlin Wu, Yifei Li

**Affiliations:** Department of Pediatrics, Ministry of Education Key Laboratory of Women and Children's Diseases and Birth Defects, West China Second University Hospital, Sichuan University, Chengdu, China

**Keywords:** arrhythmia, cardiovascular complications, heart failure, Kawasaki disease, programmed diseases

## Abstract

**Background:**

Kawasaki disease (KD) occurring in childhood has been epidemiologically associated with increased adult cardiovascular risk, particularly in children who develop coronary aneurysms during the acute phase. However, the causal nature of this association remains uncertain. This study employed genetic causal association analysis to investigate potential causal effects of KD on adult-onset cardiovascular complications. This study provides new data from our cohort, representing the largest sample size for a KD genome-wide association study (GWAS) analysis and has not been reported before.

**Methods:**

We first enrolled a prospective KD cohort of 316 patients who received whole-exome sequencing (WES) analysis. We then performed a GWAS analysis and conducted further analyses using summary-level statistics from the IEU Open GWAS database, the GWAS Catalog, and our East Asian KD cohorts. The inverse variance weighted (IVW) method served as the primary analytical approach. All analyses were performed using R software.

**Results:**

We established the largest WES-based KD cohort to date, with 316 children receiving WES and GWAS analyses. Then, in the following assessment, no significant causal associations were observed between KD and major adult cardiovascular outcomes. Replication analyses in European populations revealed modest evidence of potential causal associations with specific conditions. The IVW method indicated weak causal associations with ventricular arrhythmia (OR = 1.0296, 95% CI = 1.0037–1.0562, *P* = 0.025) and chronic heart failure (OR = 1.0110, 95% CI = 1.0007–1.0213, *P* = 0.0354) in particular traits. However, neither association was reproduced across independent GWAS datasets or corroborated by sensitivity analyses; therefore, these observations should be regarded as exploratory.

**Conclusion:**

This analysis provides genetic evidence that does not support a strong causal relationship between childhood KD and an increased risk of most adult cardiovascular diseases. Given the limited KD GWAS sample sizes and relaxed instrument-selection thresholds, these findings should be interpreted cautiously. However, they do not exclude clinically important long-term cardiovascular sequelae in patients with coronary artery involvement after KD.

## 1 Introduction

Kawasaki disease (KD) is a multisystem vasculitis of unknown etiology that predominantly affects infants and young children, with peak incidence occurring between 6 months and 5 years of age (1). This acute inflammatory condition is characterized by necrotizing vasculitis involving small- to medium-sized arteries throughout the body, with a particular predilection for the coronary vessels. Since its initial description in 1967 by Japanese pediatrician Tomisaku Kawasaki, KD has emerged as the leading cause of acquired heart disease in children in developed nations, surpassing acute rheumatic fever in regions where streptococcal infections have been effectively controlled through improved sanitation and antibiotic therapy (2). While KD is generally regarded as a self-limiting condition with a favorable short-term prognosis when treated appropriately with intravenous immunoglobulin (IVIG) and aspirin therapy, mounting clinical evidence indicates that the cardiovascular sequelae of this childhood illness may extend far beyond the acute phase. The pathological hallmark of KD involves widespread arterial inflammation that can result in coronary artery aneurysms, dilatation, and subsequent remodeling processes that persist into adulthood (3, 4). These vascular abnormalities, initially considered transient manifestations of acute inflammation, are increasingly recognized as potential substrates for long-term cardiovascular complications (5).

Contemporary epidemiological investigations have revealed that coronary artery complications arising during childhood KD may not only persist but also progress throughout the lifespan, potentially contributing to premature coronary artery disease (CAD) in young adults (6). The natural history of KD-associated coronary lesions demonstrates a complex trajectory involving initial aneurysm formation, followed by progressive intimal proliferation, smooth muscle cell migration, and eventual luminal narrowing through stenotic remodeling (7, 8). In certain cases, adult-onset CAD may be attributable to previously undiagnosed or subclinical KD episodes during childhood, in which the acute inflammatory phase was either asymptomatic or misdiagnosed as other febrile illnesses. Even following apparent clinical resolution of acute symptoms, KD-related vascular injury may continue to evolve, leading to diverse long-term complications including persistent coronary aneurysms, progressive stenosis, accelerated atherosclerosis, and premature vascular calcification (9, 10).

Childhood cardiovascular diseases, particularly those involving systemic vasculitis such as Kawasaki disease, may initiate complex pathophysiological cascades that potentially program adult cardiovascular events through several interconnected mechanisms (5, 11, 12). Early inflammatory insults to the cardiovascular system during critical developmental periods can induce persistent endothelial dysfunction, characterized by impaired nitric oxide bioavailability, enhanced oxidative stress, and altered vasomotor responses, thereby establishing a pro-atherogenic vascular environment that persists into adulthood (12, 13). Acute vasculitis can cause irreversible structural arterial changes, including arterial wall thickening, elastin fragmentation, and smooth muscle cell proliferation, with coronary artery aneurysms formed during childhood potentially undergoing progressive stenosis through intimal proliferation and thrombosis, creating a substrate for future ischemic events (14, 15). Childhood cardiovascular attacks may establish chronic low-grade inflammatory states through epigenetic modifications and immune system reprogramming, with persistent elevation of inflammatory mediators contributing to accelerated atherosclerosis and plaque instability. Direct myocardial involvement during acute phases can result in subclinical myocardial fibrosis, altered cardiac electrophysiology, and impaired ventricular function, predisposing to arrhythmias, heart failure, and sudden cardiac death in adulthood. In addition, early cardiovascular stress may influence metabolic pathways, potentially affecting lipid metabolism, insulin sensitivity, and blood pressure regulation throughout life, thereby amplifying long-term cardiovascular risk through multifaceted programming mechanisms. The inflammatory milieu generated during acute KD episodes appears to create a predisposing vascular environment that may amplify the deleterious effects of traditional risk factors, including hypertension, dyslipidemia, tobacco exposure, diabetes mellitus, and metabolic syndrome. Epidemiological surveys conducted in populations with high KD incidence indicate that approximately 30%–40% of adult individuals with documented childhood KD history exhibit one or more of these conventional cardiovascular risk factors, potentially creating a multiplicative rather than additive risk profile (16–18).

Of particular clinical concern is the recognition that previously undiagnosed childhood KD may contribute to ostensibly idiopathic coronary events in young adults, including asymptomatic myocardial infarction (MI) and sudden cardiac death. These silent ischemic events may subsequently lead to subclinical left ventricular dysfunction, electrical remodeling, and increased susceptibility to malignant arrhythmias, thereby substantially elevating long-term cardiovascular mortality risk. The diagnostic challenge posed by subclinical KD underscores the potential for significant underestimation of its true epidemiological impact on adult cardiovascular health. Despite these compelling observational associations, the causal nature of the relationship between childhood KD and subsequent adult cardiovascular outcomes remains fundamentally uncertain. While epidemiological studies have consistently identified clinical co-occurrence of KD and various cardiovascular diseases, these associations may be substantially confounded by unmeasured environmental factors, genetic susceptibility, socioeconomic determinants, and residual lifestyle influences that are difficult to account for in conventional study designs. The underlying molecular and cellular mechanisms linking acute childhood vasculitis to adult cardiovascular pathogenesis remain incompletely elucidated, and it remains unclear whether KD plays a direct causal role in cardiovascular disease pathogenesis or is a marker of shared susceptibility factors (15).

Mendelian randomization (MR) has emerged as a particularly powerful methodological approach for establishing causal inference in epidemiological research, effectively addressing many of the fundamental limitations inherent in traditional observational study designs (18). By utilizing naturally occurring genetic variants as instrumental variables (IVs) for exposure assessment, MR substantially reduces the confounding influence of environmental factors, socioeconomic status, behavioral patterns, and unmeasured covariates that typically compromise causal interpretation in observational studies. This genetic epidemiological approach leverages the random assortment of alleles during meiosis to create natural randomized experiments within populations, thereby approximating the conditions of controlled trials for exposures that cannot be ethically or practically randomized. To address the critical knowledge gaps identified in previous research and provide robust evidence regarding the causal relationship between childhood KD and adult cardiovascular disease risk, we first established a prospective cohort of pediatric KD patients, all of whom underwent whole-exome sequencing (WES). To our knowledge, this represents the largest pediatric patient cohort with WES data to date. We then employed a comprehensive two-sample MR analytical framework to systematically evaluate potential causal associations between KD and diverse cardiovascular outcomes in adulthood.

## 2 Materials and methods

### 2.1 Data sources

Summary-level genome-wide association study (GWAS) data for most of the exposure and outcome traits were obtained from publicly accessible databases, including the GWAS Catalog and the IEU Open GWAS platform. To minimize potential bias caused by population stratification, the MR analyses were performed separately in individuals of European and East Asian ancestry.

We established the largest WES-based KD cohort in an East Asian population, comprising 316 children who underwent WES and GWAS analyses. The genetic summary data for KD were derived from whole-genome analyses of peripheral blood samples collected from pediatric patients diagnosed with KD at West China Second University Hospital, Sichuan University. Details of all datasets, including sample sizes and genotyping platforms, are provided in Supplementary Table S1. The study involving genetic data from patients with KD was approved by the Ethics Committee of West China Second University Hospital, Sichuan University (approval number: 2021-069). Written informed consent was obtained from the parents or legal guardians of all participating children prior to sample collection. All procedures were conducted in accordance with the Declaration of Helsinki and relevant national and institutional ethical guidelines. This cohort was collected between April 2020 and January 2022. All patients were diagnosed with completed KD according to the AHA guidelines for the diagnosis and management of KD. The following patients were excluded: those with (1) hereditary metabolic diseases, (2) congenital heart disease or cardiomyopathy, (3) coronary artery developmental abnormalities, (4) persistent cardiac arrhythmias, (5) malignant diseases, (6) SARS-CoV-2-induced systemic inflammatory response syndrome, and (7) immunodeficiency disorders or autoimmune diseases. Peripheral blood (2–3 mL) was collected with EDTA-anticoagulated tubes for WES analysis by Novogene Co., Ltd. Genomic DNA was extracted, fragmented, and processed through end repair and A-tailing to construct DNA libraries. Following quality control, the libraries underwent high-throughput sequencing on the Illumina platform. Raw sequencing data were quality-assessed and aligned to the human reference genome (human_B37) using BWA and Samblaster. Alignment results were sorted, duplicate-marked, and analyzed for SNP identification and filtering using SAMtools.

For the European population, summary-level statistics for KD were retrieved from the IEU Open GWAS database (originally deposited via the GWAS Catalog), derived from a Polish cohort comprising 119 cases and 6,071 controls published by Buda et al. (19).

For the outcome traits, we applied the same population-specific filtering strategy to the two publicly accessible GWAS datasets described above to further investigate the causal associations between the exposure and outcomes. All GWAS identifiers used in this study are listed in Supplementary Table S2.

### 2.2 Instrumental variable selection

IVs were selected based on three core assumptions of Mendelian randomization: (i) strong association with the exposure (KD); (ii) independence from potential confounders; and (iii) influence on the outcome only through the exposure. For the East Asian ancestry dataset, SNPs associated with KD were extracted from the corresponding genome-wide association data using a significance threshold of *P* < 1 × 10^⁻⁴^ for the small-sample-size cohort (20, 21). To ensure independence among instruments, linkage disequilibrium (LD) clumping was performed with a window size of 10,000 kb and an LD cutoff of *r^2^* < 0.001. For the European ancestry dataset, SNPs significantly associated with KD were initially identified as candidate instruments at the genome-wide significance threshold (*P* < 5 × 10^⁻⁸^). As the number of genome-wide significant variants was limited (fewer than three independent loci), the selection threshold was relaxed to *P* < 1 × 10^⁻⁵^ to increase the number of eligible instruments and facilitate downstream MR analyses (22). All selected SNPs were harmonized across exposure and outcome datasets, and weak instruments (F-statistic < 10) were removed prior to analysis. Second, SNPs with a minor allele frequency of less than 0.01 were excluded to avoid potential biases arising from rare variants. Third, to minimize LD among selected IVs, clumping was performed using the European population reference panel from the 1000 Genomes Project. SNPs were retained only if they were in low LD (*r*^2^ < 0.01) and separated by at least 5,000 kb. Potential pleiotropy and confounding associations were assessed using PhenoScanner, and SNPs associated with known confounders were excluded.

After applying the aforementioned selection and quality-control criteria, eight independent SNPs were identified from the IEU Open GWAS database as IVs for KD in the European population. Similarly, six independent SNPs were selected as IVs for KD from the East Asian GWAS dataset, using the same significance thresholds and LD-clumping parameters described above. All selected variants were mutually independent (*r*^2^ < 0.001), met the relevance assumption of Mendelian randomization, and exhibited sufficient instrument strength (F-statistic > 10).

### 2.3 Mendelian randomization analysis

**Figure 1:**
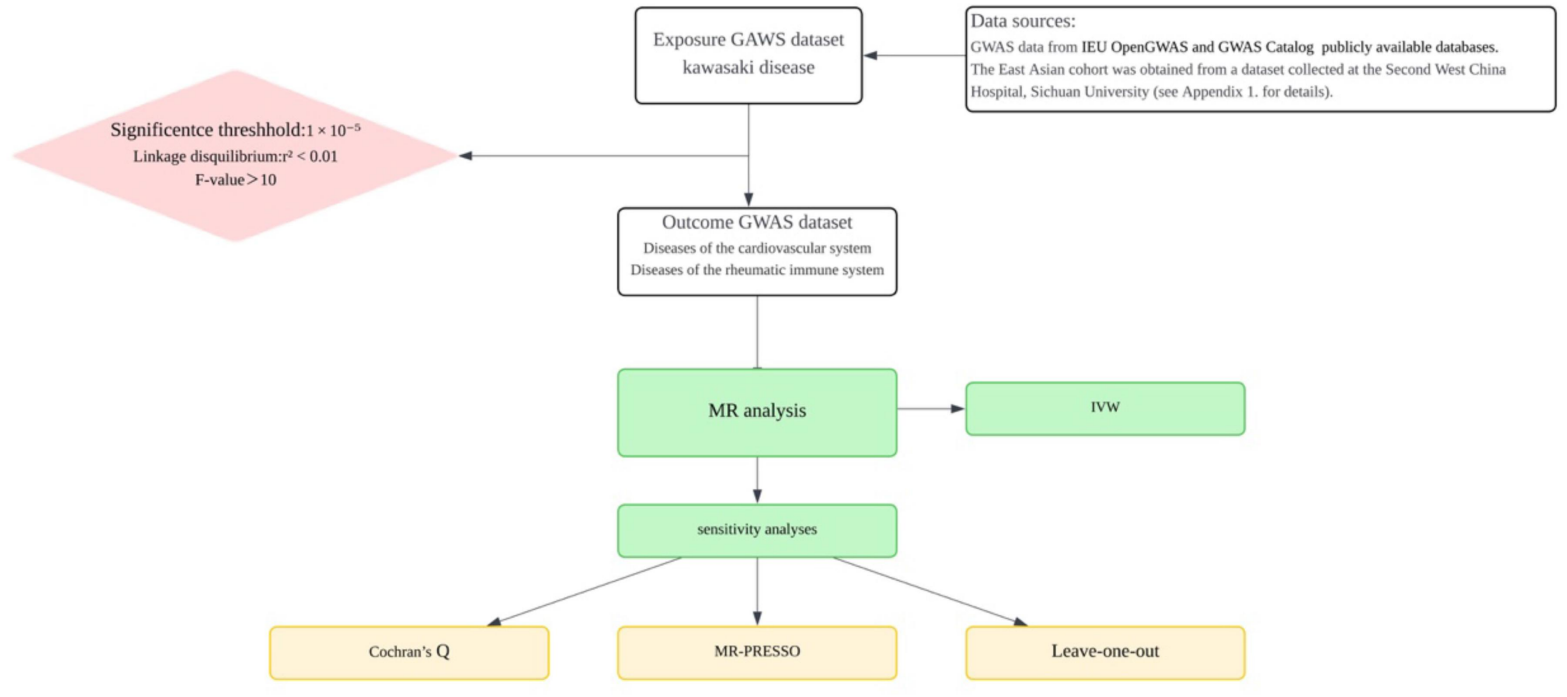
Schematic of the workflow in this study.

Two-sample MR analyses were conducted using the selected IVs in R software (version 4.4.3) with the TwoSampleMR package. Five MR methods were applied to estimate the causal effect of KD on cardiovascular disease: the primary method was the random-effects inverse variance weighted (IVW) approach, supported by four complementary methods—MR-Egger regression, weighted median, simple mode, and weighted mode. The random-effects IVW model served as the primary basis for causal inference in this study. The detailed workflow is presented in Figure 1. Cochran's *Q* statistic was used to assess heterogeneity across instrumental variables; a *P*-value > 0.05 indicated no significant heterogeneity. Horizontal pleiotropy was evaluated using the MR-Egger intercept test, with a *P*-value > 0.05 suggesting no evidence of directional pleiotropy.

For analyses with a sufficient number of harmonized instruments, the MR-PRESSO global test was used to detect evidence of horizontal pleiotropy. If outlying variants were detected, outlier-corrected estimates and distortion test results were recorded. The full MR-PRESSO results are shown in Supplementary Table S8. For the primary IVW analyses, *P* values were additionally adjusted using the Holm–Bonferroni method within the cardiovascular outcome group. In addition, in this analysis, each SNP was iteratively removed from the instrumental variable set to assess its individual influence on the overall causal estimate. The stability of the results was further evaluated by examining whether the association between Kawasaki disease and adverse cardiovascular events was driven by a single genetic variant, thereby supporting the robustness and reliability of the causal inference.

## 3 Results

### 3.1 Associations between Kawasaki disease and cardiovascular outcomes

#### 3.1.1 East Asian population

Initially, 316 children met the study inclusion and exclusion criteria (KD group: *n* = 177; control group: *n* = 139). The KD cohort included 103 boys (58.2%) and 74 girls (mean age: 3.2 ± 2.2 years), with coronary artery lesions documented in 32 patients (18.1%) and IVIG resistance in 55 patients (31%). The control group comprised 50 boys (36%) and 89 girls (mean age: 3.8 ± 2.6 years). Following upstream analysis and screening protocols, 14,142 SNP loci were selected for subsequent investigation. In the East Asian cohort, we conducted eight MR analyses to assess the potential causal impact of genetically predicted KD on multiple cardiovascular endpoints.

**Figure 2:**
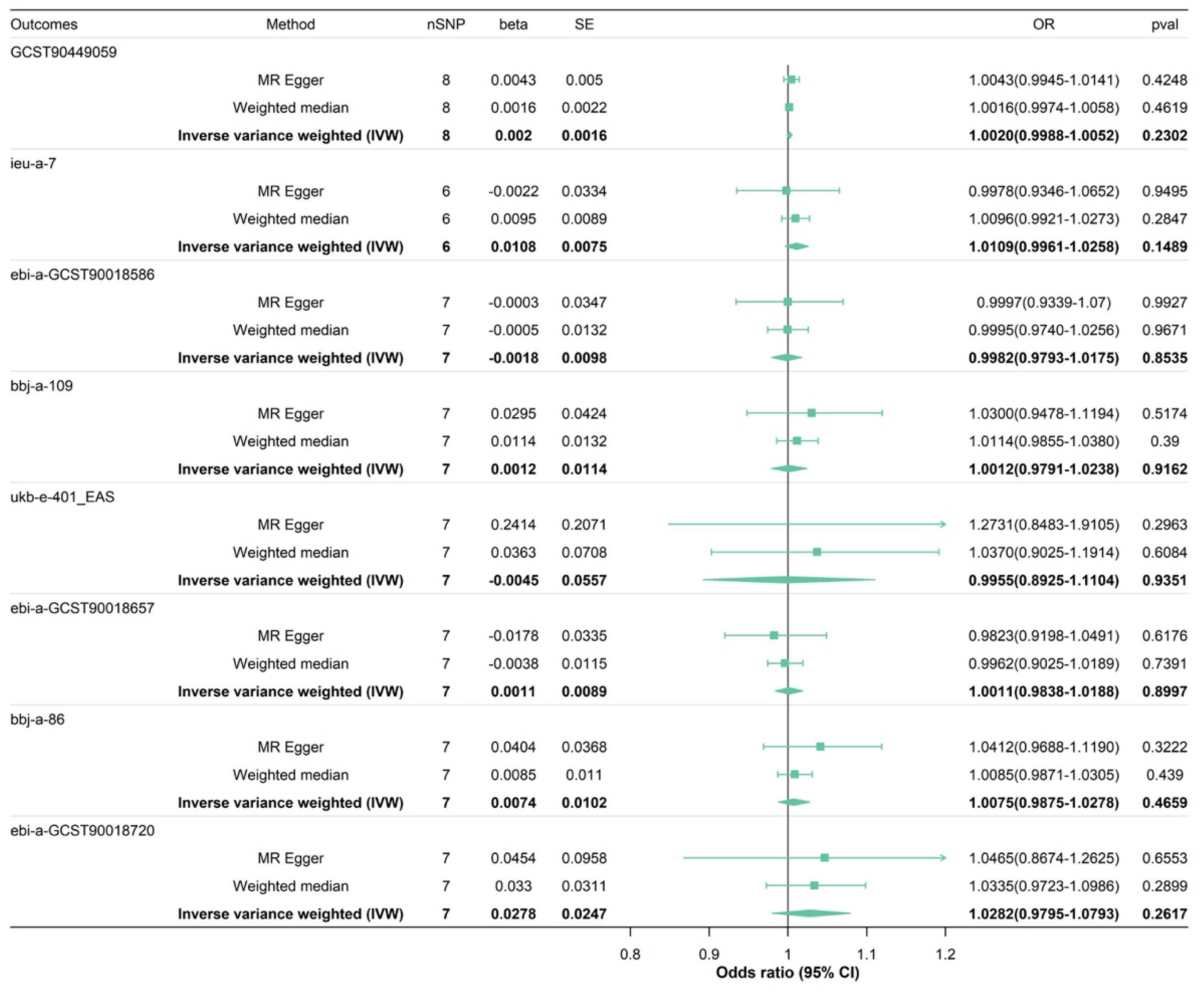
Mendelian randomization estimates of the causal association between KD and adverse cardiovascular outcomes in East Asian populations.

To ensure methodological rigor and address potential violations of the instrumental variable assumption, causal estimates were derived using three complementary analytical approaches: IVW regression as the primary estimator, supplemented by the weighted median and MR-Egger methods for enhanced robustness assessment. As depicted in Figure 2, the Mendelian randomization estimates demonstrated remarkable consistency across all analytical methods employed. The comprehensive analysis encompassed five cardiovascular outcomes: CAD, heart failure, hypertension, MI, and cardiac arrhythmia. Notably, no statistically significant causal associations were identified for any of the investigated outcomes (all *P*-values > 0.05). The IVW-derived effect estimates consistently approximated the null hypothesis, with concordant directional estimates observed across the weighted median and MR-Egger models, thereby supporting the robustness of our findings (detailed results are presented in Supplementary Table S3 and Supplementary File 2). To further validate these null associations, extensive sensitivity analyses were conducted to assess potential methodological limitations. Specifically, tests for heterogeneity among instrumental variables and horizontal pleiotropy (evaluated using the MR-Egger intercept) revealed no evidence of model instability or systematic directional pleiotropy bias. These sensitivity assessments collectively support the validity of our null causal inferences (comprehensive sensitivity analysis results available in Supplementary Table S4 and Supplementary File 3). Overall, these results provide no evidence of genetically mediated causal effects of KD on major adult cardiovascular diseases in East Asian populations. Although acute-phase KD may induce transient coronary abnormalities during childhood, its inherited liability does not appear to exert detectable long-term causal influence on cardiovascular risk trajectories.

To further evaluate ancestry-specific patterns and validate these findings, parallel analyses were performed in individuals of European descent.

#### 3.1.2 European population

**Figure 3:**
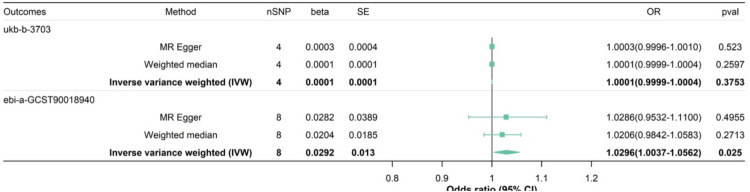
Mendelian randomization estimates for the association between KD and arrhythmia.

**Figure 4:**
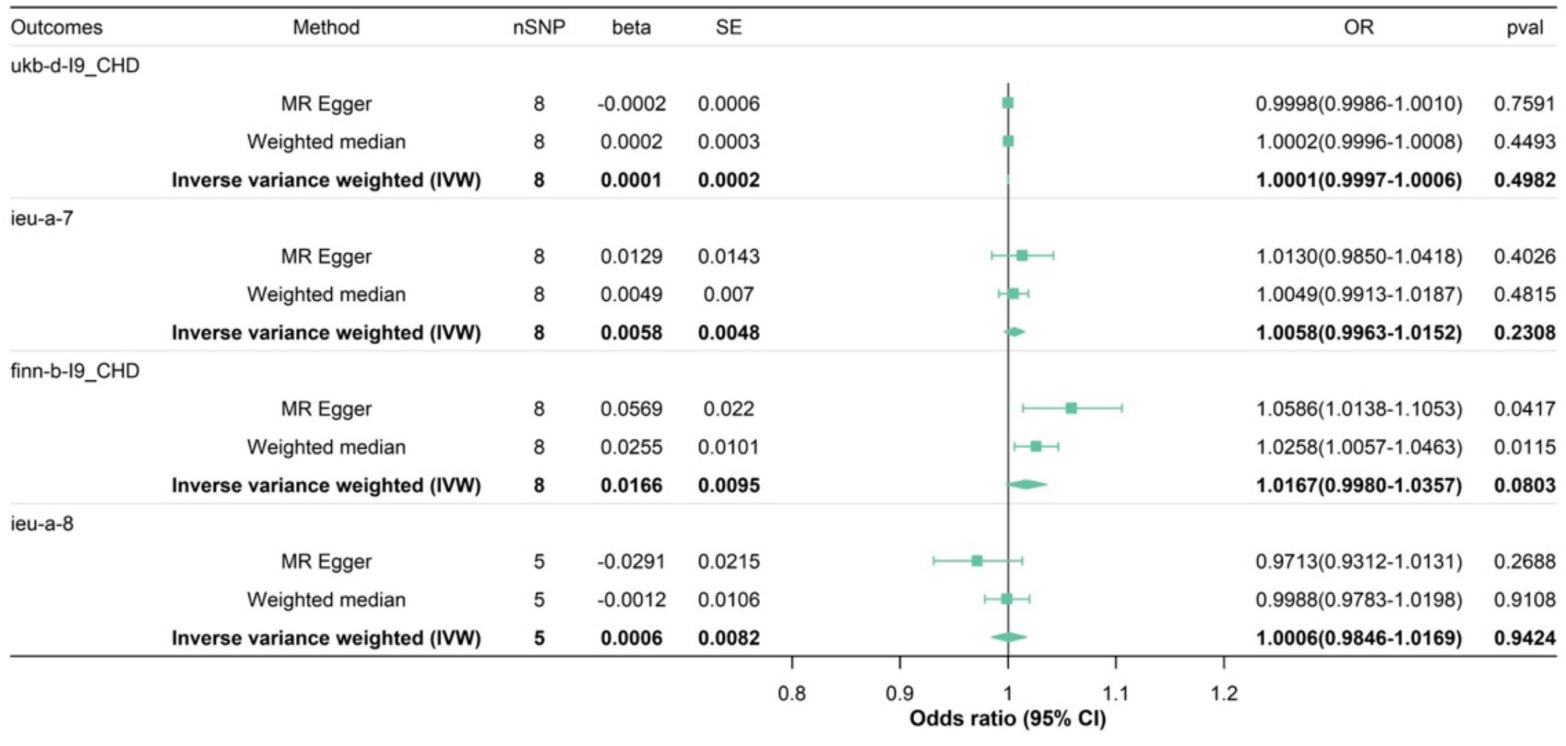
Mendelian randomization estimates for the association between KD and CAD.

**Figure 5:**
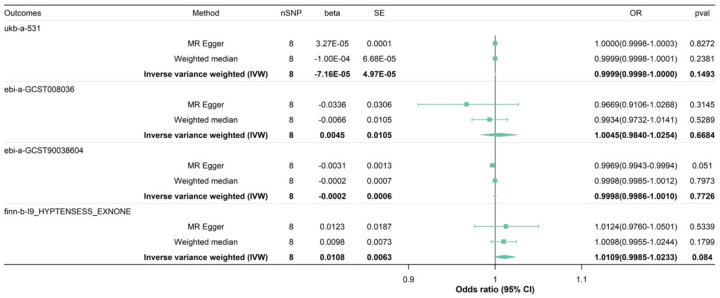
Mendelian randomization estimates for the association between KD and hypertension.

**Figure 6:**
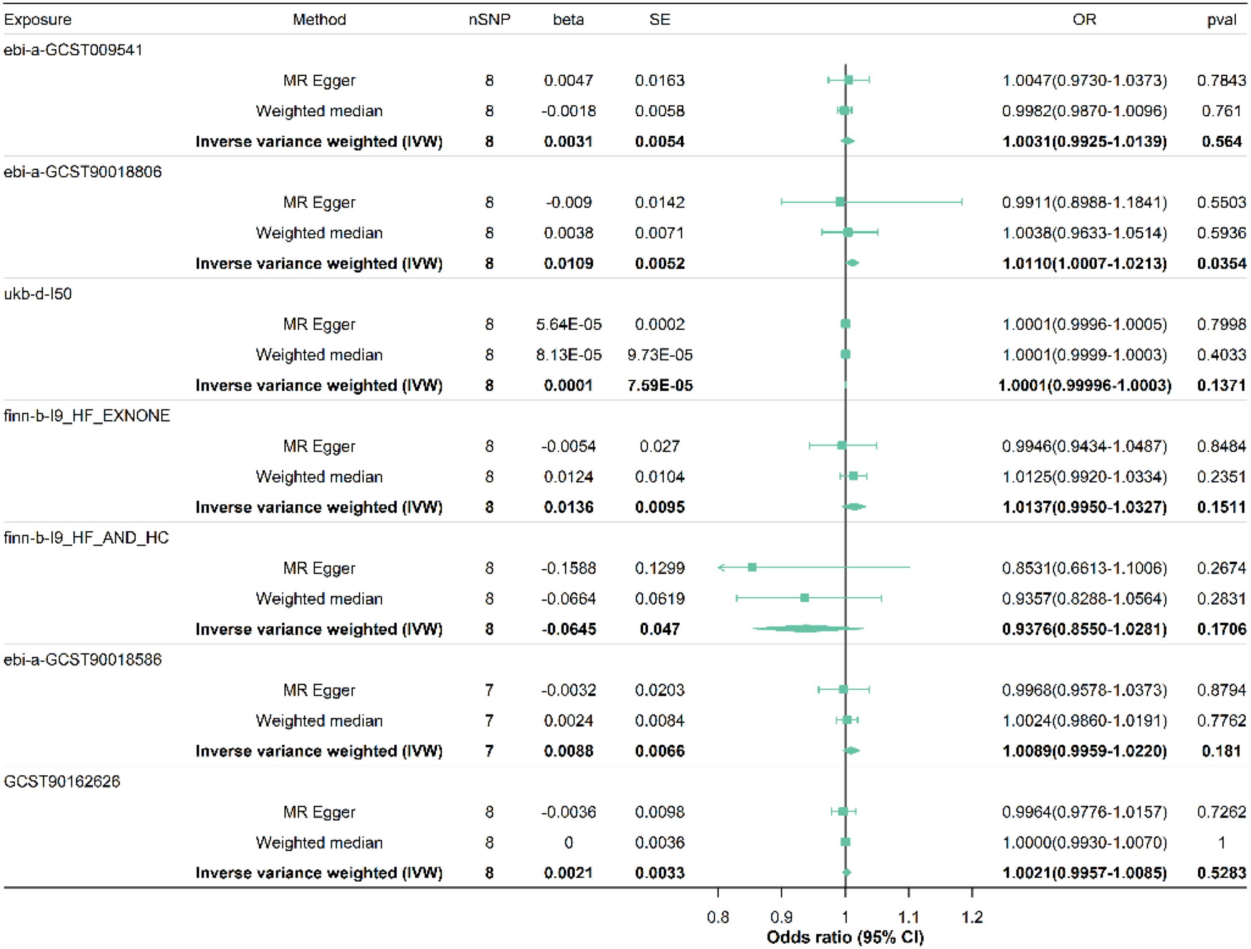
Mendelian randomization estimates for the association between KD and heart failure.

**Figure 7:**
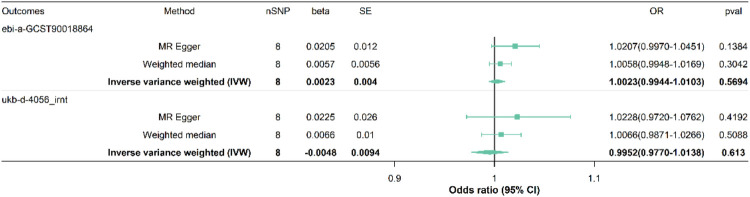
Mendelian randomization estimates for the association between KD and ischemic stroke.

**Figure 8:**
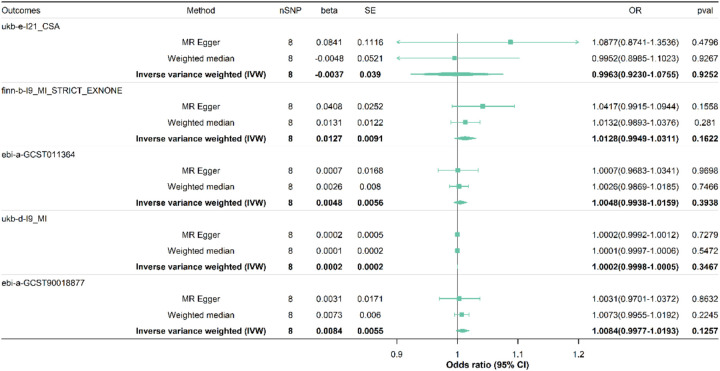
Mendelian randomization estimates for the association between KD and MI.

To evaluate the generalizability of our findings across distinct genetic ancestries, parallel MR analyses were conducted in European cohorts, yielding results that were largely concordant with those observed in East Asian populations. Employing the same triangulated analytical framework, including the IVW, weighted median, and MR-Egger methods, we systematically assessed the causal relationship between KD genetic susceptibility and an expanded spectrum of cardiovascular phenotypes, including CAD, MI, hypertension, cardiac arrhythmia, ischemic stroke, and heart failure (Figures 3–8). Consistent with the East Asian analysis, the majority of cardiovascular traits demonstrated no statistically significant causal associations (all *P*-values > 0.05), with effect estimates consistently centered near the null across all analytical methods (detailed results are provided in Supplementary Table S5). This pattern of null findings reinforces the robustness of our primary conclusions across genetically distinct populations. However, one notable exception emerged in the CAD analysis, where nominal statistical significance was observed in sensitivity estimators (weighted median, weighted mode, and MR-Egger methods; detailed results are provided in Supplementary Table S6). Critically, this association failed to achieve significance in the IVW analysis—the method with superior statistical efficiency under valid instrumental variable assumptions. Furthermore, diagnostic tests indicated potential weak instrument bias or horizontal pleiotropy, suggesting methodological limitations rather than genuine causal effects. Given these methodological concerns and the inconsistency across analytical approaches, this isolated finding warrants cautious interpretation and provides limited evidence for a robust causal relationship between KD susceptibility and CAD risk in European populations.

Within the European analyses, nominal associations were observed for ventricular arrhythmia (ebi-a-GCST90018940; OR = 1.0296, 95% CI = 1.0037–1.0562, *P* = 0.025) and chronic heart failure (ebi-a-GCST90018806; OR = 1.0110, 95% CI = 1.0007–1.0213, *P* = 0.0354). These findings should therefore be interpreted as exploratory. First, neither association demonstrated reproducibility across independent GWAS datasets, raising concerns about the stability and generalizability of these effects. Second, sensitivity analyses failed to corroborate these associations, suggesting potential violations of core instrumental variable assumptions or the presence of unmeasured confounding. The absence of replication across multiple datasets and analytical approaches substantially diminishes the likelihood that these represent genuine causal relationships rather than statistical artifacts or population-specific confounding. Moreover, these nominal associations did not meet the multiple-testing-adjusted significance threshold when considering the cardiovascular outcomes examined in the European analyses. When considered within the broader analytical framework, the European findings predominantly reinforce the pattern of null associations observed in the East Asian cohort. This cross-ancestral consistency strengthens our confidence in the overall conclusions, as genuine causal effects would be expected to manifest across genetically diverse populations, albeit potentially with varying effect sizes.

Integrating evidence across both East Asian and European ancestries, our comprehensive MR investigation provides no substantive evidence that genetic predisposition to KD confers meaningful risk for major cardiovascular diseases in adulthood. The consistency of null findings across diverse genetic backgrounds, multiple analytical methods, and extensive sensitivity testing collectively does not provide evidence supporting clinically relevant causal relationships between KD susceptibility and long-term cardiovascular outcomes.

## 3.2 Associations between Kawasaki disease and immune-mediated diseases

**Figure 9:**
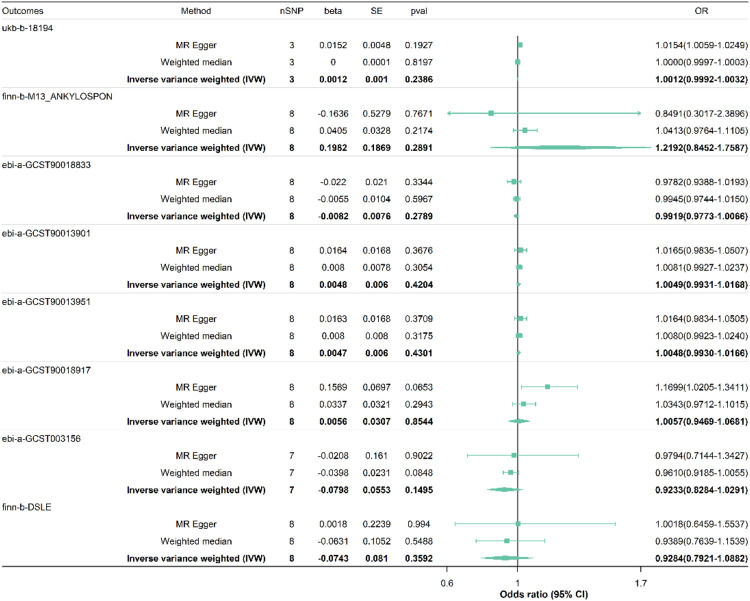
Mendelian randomization estimates for the causal association between KD and immune disorders in European populations.

Given that KD is a systemic immune-mediated vasculitis, with observational evidence suggesting potential long-term immune dysregulation in survivors, we extended our analytical framework to investigate causal relationships between KD genetic liability and immune-related pathologies. This hypothesis-driven approach was motivated by the mechanistic plausibility that shared genetic susceptibility pathways might confer risk for multiple inflammatory conditions. We systematically evaluated four immune-mediated disorders, including ankylosing spondylitis, inflammatory bowel disease, systemic lupus erythematosus, and depression, employing the established triangulation of IVW, weighted median, and MR-Egger estimators (comprehensive results are provided in Supplementary Table S7). As demonstrated in Figure 9, this analysis yielded uniformly null findings across all investigated outcomes.

**Figure 10:**
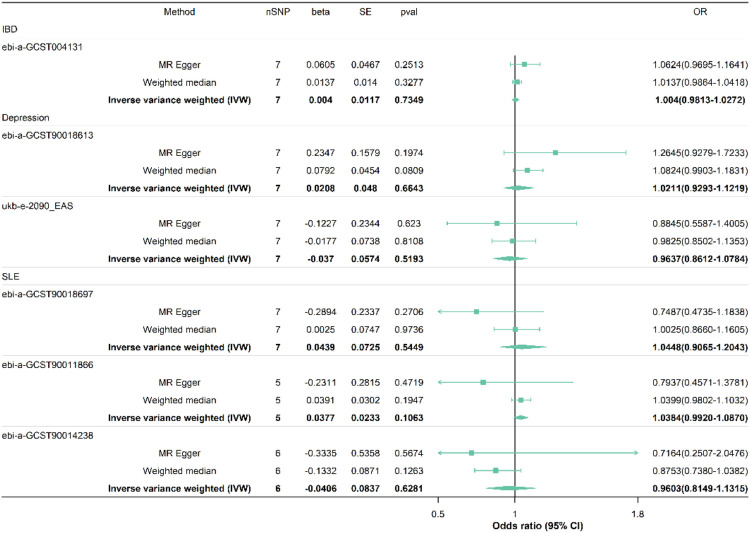
Mendelian randomization estimates for the association between genetically predicted Kawasaki disease and immune-related disorders in East Asian populations.

Specifically, no statistically significant causal associations were detected for any immune-related disorder (all *P*-values > 0.05), with effect estimates consistently approximating unity. To strengthen ancestry-specific evidence, we also performed analyses of KD and immune-related outcomes in the East Asian population. As shown in Figure 10, no statistically significant associations were observed for inflammatory bowel disease, depression, or systemic lupus erythematosus. These results were broadly consistent with the overall immune-related analyses but should be interpreted cautiously given the limited number of instruments and the availability of outcome datasets. While modest directional trends were observed in select datasets (e.g., KD–SLE1: OR = 1.17; KD–IBD1: OR = 1.02), these associations were characterized by imprecise estimates with wide confidence intervals and failed to demonstrate reproducibility across independent GWAS datasets. Comprehensive sensitivity analyses revealed no evidence of substantial instrumental variable heterogeneity or horizontal pleiotropy, thereby supporting the validity of these null associations.

Taken together, the current analyses did not provide evidence supporting a measurable causal association between genetic predisposition to KD and the examined immune-mediated or inflammatory disorders in adulthood. When synthesized alongside our cardiovascular MR analyses in both East Asian and European populations, the cumulative evidence argues against KD representing a persistent, genetically encoded determinant of adult cardiovascular or immunological dysfunction. This pattern of null causal associations has important clinical and mechanistic implications. The long-term morbidities occasionally documented in longitudinal clinical studies may more plausibly reflect acquired pathophysiological sequelae, including early-life coronary arterial injury, environmental exposures, or modifiable metabolic risk factors, rather than constitutive genetic susceptibility mechanisms. This distinction suggests that preventive strategies should focus on mitigating acquired risk factors rather than assuming an inevitable genetic predisposition to adverse outcomes.

The findings of this study should be interpreted in the context of current KD management. Although this MR analysis did not provide evidence supporting a causal association between genetic liability to KD and adult cardiovascular outcomes, it does not diminish the established risk associated with coronary artery sequelae. Patients with coronary involvement, particularly those with persistent or large aneurysms, remain at increased risk of myocardial ischemia, arrhythmia, and heart failure later in life. Therefore, current follow-up strategies and management recommendations should not be altered based on these findings. Long-term surveillance remains essential and should continue to focus on coronary artery structure, myocardial function, and electrophysiological abnormalities.

## 4 Discussion

In this study, we performed a two-sample MR analysis using multiple GWAS datasets to evaluate the causal relationship between KD and cardiovascular diseases in adulthood. In particular, we also involved our KD cohort to establish the causal relationship between KD and adult cardiovascular disease in the East Asian population, which helps validate these findings across various ethnic populations. Our findings suggest that KD, which most commonly occurs between 6 months and 5 years of age, did not provide evidence for an increased risk of several specific cardiovascular events later in life. In particular, genetically predicted childhood KD was only associated with a higher risk of a few cardiovascular outcomes in adulthood, which were also limited to particular traits, including ventricular arrhythmia (for ebi-a-GCST90018940 trait, OR = 1.0296, 95% CI = 1.0037–1.0562, *P* = 0.025) and chronic heart failure (OR = 1.011, 95% CI = 1.001–1.021). In the largest cardiovascular outcome study of KD patients conducted in North America, Robinson et al. identified ventricular arrhythmia as one of the secondary endpoints and demonstrated a significantly higher incidence of composite cardiovascular events among KD patients than among controls (*P* < 0.001), with a median follow-up duration of 11.1 years (23). These results are consistent with previous prospective observational studies identifying KD as an important etiological factor for premature CAD in adults, particularly in some specific cohorts, and may not be consistent across various traits. Undeniably, from a pathophysiological perspective, the acute coronary arteritis observed during KD may induce structural damage to the vessel wall, which could predispose patients to late complications such as thrombosis or progressive atherosclerotic changes years after the initial illness. To our knowledge, this is the first comprehensive MR study to systematically examine the relationship between KD and a spectrum of cardiovascular outcomes in adulthood. This study assessed genetic liability to KD rather than its clinical course. This distinction is important because genetic susceptibility does not capture disease severity, treatment response, or coronary artery involvement, all of which may influence long-term prognosis. Thus, the absence of evidence for causal associations in this MR analysis should be interpreted as relating to genetic predisposition and should not be extrapolated to patients with severe KD phenotypes or inadequate treatment.

Depression is not a classical immune-mediated disease but was considered in this exploratory analysis because inflammatory pathways may contribute to depressive symptoms in some patients (24). Meta-analyses have shown elevated circulating pro-inflammatory markers, particularly interleukin-6 and tumor necrosis factor-α, in patients with depression (25). In parallel, randomized trials have indicated that anti-inflammatory interventions may alleviate depressive symptoms in some individuals, although the effects are heterogeneous and seem more evident in those with an increased inflammatory burden. Collectively, these findings do not support classifying major depressive disorder as a classical autoimmune disease, but they do justify its consideration as an immune-associated condition in analyses of disorders with plausible inflammatory underpinnings.

Although some positive causal effects were identified for some traits, across all MR approaches (IVW, weighted median, and MR-Egger), no significant associations were detected between KD and CAD, heart failure, hypertension, MI, arrhythmia, and stroke. These findings indicate that KD was not supported as a general cardiovascular risk factor in this MR analysis. Although epidemiological studies have consistently reported increased incidence of CAD or MI in long-term KD cohorts (26), our genetic causal inference suggests that such observational associations may be attributable to secondary processes, such as aneurysm-related stenosis and thrombosis, or to the cumulative effects of conventional risk factors in adulthood (e.g., dyslipidemia, smoking, or metabolic syndrome), rather than a direct genetic liability linking KD to systemic atherosclerotic disease. In other words, the cardiovascular risk conferred by KD appears to be driven primarily by localized vascular structural alterations rather than by a generalized predisposition to atherosclerosis.

KD is one of the leading causes of acquired heart disease in children in developed countries. Despite timely IVIG administration in the acute phase, long-term cardiovascular surveillance remains essential due to the risk of late complications (27). Sumitomo et al. performed electrophysiological studies in 40 pediatric patients with moderate to severe coronary artery lesions secondary to KD and identified four cases of ventricular arrhythmias, including premature ventricular contractions, ventricular tachycardia, and ventricular fibrillation, the most severe form (11). These findings provide direct electrophysiological evidence supporting the occurrence of malignant ventricular arrhythmias in KD patients. Notably, 7 of the 40 patients exhibited an abnormal corrected sinus node recovery time (281 ± 130 ms), 4 patients demonstrated decreased Wenckebach points (<164 ± 37 bpm), and 2 patients showed prolonged AH intervals, suggesting atrioventricular nodal conduction abnormalities (11). These conduction disturbances may constitute the electrophysiological substrate underlying the development of ventricular arrhythmias. From a pathophysiological perspective, KD patients with coronary artery involvement are at increased risk of myocardial ischemia due to coronary stenosis or occlusion, which can provoke ventricular electrical instability. Moreover, chronic ischemia or post-infarction myocardial remodeling may serve as a structural and electrophysiological substrate for the initiation and maintenance of arrhythmias (28, 29). Nonetheless, the observed associations between these exposures and outcomes are based on pathophysiological reasoning rather than causal inference and thus do not demonstrate a causal link between KD and arrhythmic events.

Similarly, we found no evidence supporting a causal relationship between KD and hypertension. This finding does not support a detectable genetic contribution of KD liability to systemic blood pressure regulation within the present MR framework. Clinically, these results should not be interpreted as reducing the need for cardiovascular follow-up in patients with a history of KD, particularly those with coronary artery involvement. In contrast to the nominal findings observed in the European cohorts for ventricular arrhythmia and chronic heart failure, analyses in the East Asian populations yielded consistently null results across all outcomes, including arrhythmia, chronic heart failure, CAD, MI, and hypertension. Several explanations may account for these discrepancies. From a pathophysiological perspective, the long-term cardiovascular consequences of KD are more likely mediated by coronary arteritis, aneurysmal sequelae, microvascular perfusion impairment, myocardial fibrosis, and electrophysiological remodeling, rather than through classical atherosclerotic pathways (30). Even in East Asia, where KD incidence is highest (16), genetic evidence did not support causal associations with CHD, MI, or hypertension, suggesting that the long-term cardiovascular risk profile of KD may be “disease-specific” rather than reflecting a generalized predisposition to cardiovascular disease.

Kawasaki disease is clinically heterogeneous, and the extent of coronary artery involvement is a key determinant of long-term cardiovascular risk. Patients with more severe coronary lesions, particularly medium-to-large or giant aneurysms, are at increased risk of later myocardial ischemia, infarction, and heart failure, whereas those without significant coronary involvement generally have more favorable outcomes. Because KD was modeled here as a binary genetic exposure, the present MR framework could not capture this clinical heterogeneity. Thus, the observed null associations should be interpreted as average effects of genetic liability to KD and may not apply to patients with severe coronary artery sequelae. Differences in clinical management may also contribute. In most countries, especially in areas with well-developed healthcare systems, children with KD often receive IVIG treatment earlier and undergo more rigorous coronary follow-up, thereby reducing the incidence of giant or progressive coronary lesions (31, 32). Such improvements in care may dilute the population-level risk of later CAD or MI, producing a “treatment–survivor effect” that biases MR estimates toward the null, particularly when adult cardiovascular outcomes manifest decades after the initial illness and are not entirely mediated by childhood coronary pathology.

From a methodological perspective, several limitations may further explain the null findings in our analysis. The KD GWAS sample size was relatively modest, and allele frequency spectra and LD structures differed from those in Europeans, leading to lower explained variance and reduced F-statistics, thereby increasing the likelihood of causal estimates biased toward zero. Phenotypic heterogeneity also warrants consideration: diagnostic criteria and subtype distributions for arrhythmia and chronic heart failure vary across databases, while composite outcomes such as CAD, MI, and hypertension may obscure KD-related associations confined to specific subgroups. The long latency between childhood KD and adult cardiovascular outcomes introduces additional challenges, as competing risks may attenuate detectable genetic associations. Furthermore, cross-ancestry analyses necessitate exclusion of palindromic variants and stringent allele harmonization, which reduces the number of effective instrumental variables and limits statistical power. Finally, if pleiotropic effects act in opposing directions, methods such as MR-Egger may yield null overall results, appearing to show “no effect” despite heterogeneous underlying mechanisms.

In childhood, the most common cardiac complication of KD is coronary artery lesions, particularly giant aneurysms (≥8 mm) and large aneurysms (6–8 mm) (12), which are prone to progressive stenosis (33). These lesions confer substantial long-term risks, including thrombosis, luminal narrowing, and calcification, thereby increasing the likelihood of myocardial infarction and late cardiac mortality. Such pathological changes are closely associated with chronic coronary ischemia and progressive cardiac dysfunction, constituting a potential mechanism for the development of chronic heart failure in adulthood. As reported by Gordon et al., even after aneurysmal regression, KD patients exhibit persistent vascular abnormalities, such as endothelial dysfunction and an increased risk of calcification, which may predispose them to premature atherosclerosis in adulthood (13). Patients with giant aneurysms generally have poorer long-term outcomes and require close surveillance. Case reports have documented adults with a history of childhood KD who subsequently developed heart failure or required interventional procedures (e.g., covered stent implantation) because of coronary aneurysm-related complications (34–36), underscoring that aneurysms and their sequelae may reduce long-term coronary flow reserve and increase the risk of myocardial ischemia. In a study by Tsuda, two young adults (aged 24 and 26 years) with a history of childhood KD presented with acute coronary syndrome despite angiographically “normal” coronary arteries; both exhibited diffuse ectasia and calcification, highlighting KD-associated endothelial dysfunction and accelerated atherosclerosis (37). The resultant calcification and stenosis can precipitate recurrent ischemic events in early adulthood, progressively impair myocardial reserve, and—through chronic ischemia and post-infarction scar formation—lead to left ventricular systolic dysfunction, ventricular remodeling, and ultimately the clinical onset of chronic heart failure, representing a classic pathophysiological progression.

Compared with previous studies that supported a long-term association between pediatric KD and coronary artery lesions, our MR investigation is the first to comprehensively assess the causal relationship between childhood KD and a spectrum of cardiovascular diseases commonly observed in adulthood. We leveraged two independent exposure GWAS datasets and applied five distinct MR methods, identifying SNPs across two genetic instrument sets to establish causal inference. In our primary analysis, results from the multiplicative random-effects IVW model served as the principal reference. Sensitivity analyses further supported the robustness and accuracy of the causal estimates. Notably, additional experimental evidence strengthens the biological plausibility of our findings. In a laboratory setting, a murine model of vasculitis induced by *Lactobacillus casei* cell wall extract (LCWE)—an established model for human KD—was monitored over a 4-month follow-up period. This model demonstrated that myocardial injury was characterized by myocarditis and early-onset myocardial fibrosis (38). The LCWE murine model provides biological plausibility for myocardial inflammation and fibrosis after vasculitis, but it does not validate a causal association between genetically predicted KD and adult cardiovascular outcomes in humans. Mechanistic hypotheses arising from experimental models should therefore be distinguished from the present MR findings.

Several limitations should be acknowledged when interpreting our findings. First, we adopted a relatively lenient significance threshold (*P* < 1 × 10^⁻⁵^) for SNP selection. Although this approach has been applied in previous MR studies (22), it inherently increases the risk of weak instrument bias. Second, the available GWAS datasets did not provide detailed phenotypic characteristics of pediatric KD, such as the acute or subacute nature of the illness or the extent of coronary artery involvement. Consequently, we were unable to classify KD patients or perform stratified MR analyses based on coronary aneurysm size. Third, we did not distinguish between ventricular arrhythmia subtypes, which might have obscured subtype-specific associations. Finally, our investigation focused solely on the risk, rather than the progression, of adult cardiovascular complications following KD.

Despite these limitations, our study lays an important foundation for future research. Future efforts should prioritize the use of larger, multi-ethnic GWAS datasets and long-term prospective cohorts to validate and extend these findings. Advanced MR approaches—including multivariable, nonlinear, and time-varying methods—may further elucidate the causal pathways linking childhood KD to adult cardiovascular disease. Integrating multi-omics data and mechanistic studies (e.g., LCWE-induced murine models of KD) could provide additional insights into the underlying biological processes driving these associations.

Our findings provide genetic evidence that does not support direct causal associations between KD susceptibility and adult cardiovascular diseases. KD-related cardiovascular complications appear primarily confined to direct sequelae of acute coronary arterial involvement, rather than representing a genetically encoded predisposition to conventional atherosclerotic diseases such as coronary artery disease, myocardial infarction, or hypertension. Importantly, these genetic findings do not negate the established long-term risks among patients with KD-related coronary artery sequelae, for whom surveillance should follow risk-stratified clinical guidance. This targeted approach may enable earlier identification of KD-specific complications and more precise clinical interventions to mitigate cardiovascular sequelae in adulthood.

Importantly, the absence of evidence in this MR analysis should not be interpreted as definitive evidence of the absence of causality, particularly given limitations related to instrument strength, phenotypic heterogeneity, and disease complexity.

## Data Availability

The datasets presented in this study can be found in online repositories. The names of the repository/repositories and accession number(s) can be found in the article/Supplementary Material.
