# Supplementary material for: Genetic liability to childhood Kawasaki disease and adult cardiovascular outcomes
Source: Front Cardiovasc Med. 2026 Jul 23;13:1848320. doi: 10.3389/fcvm.2026.1848320 (PMC13441699; doi:10.3389/fcvm.2026.1848320)
Supplement: Supplementary file 1 [file Datasheet1.pdf]

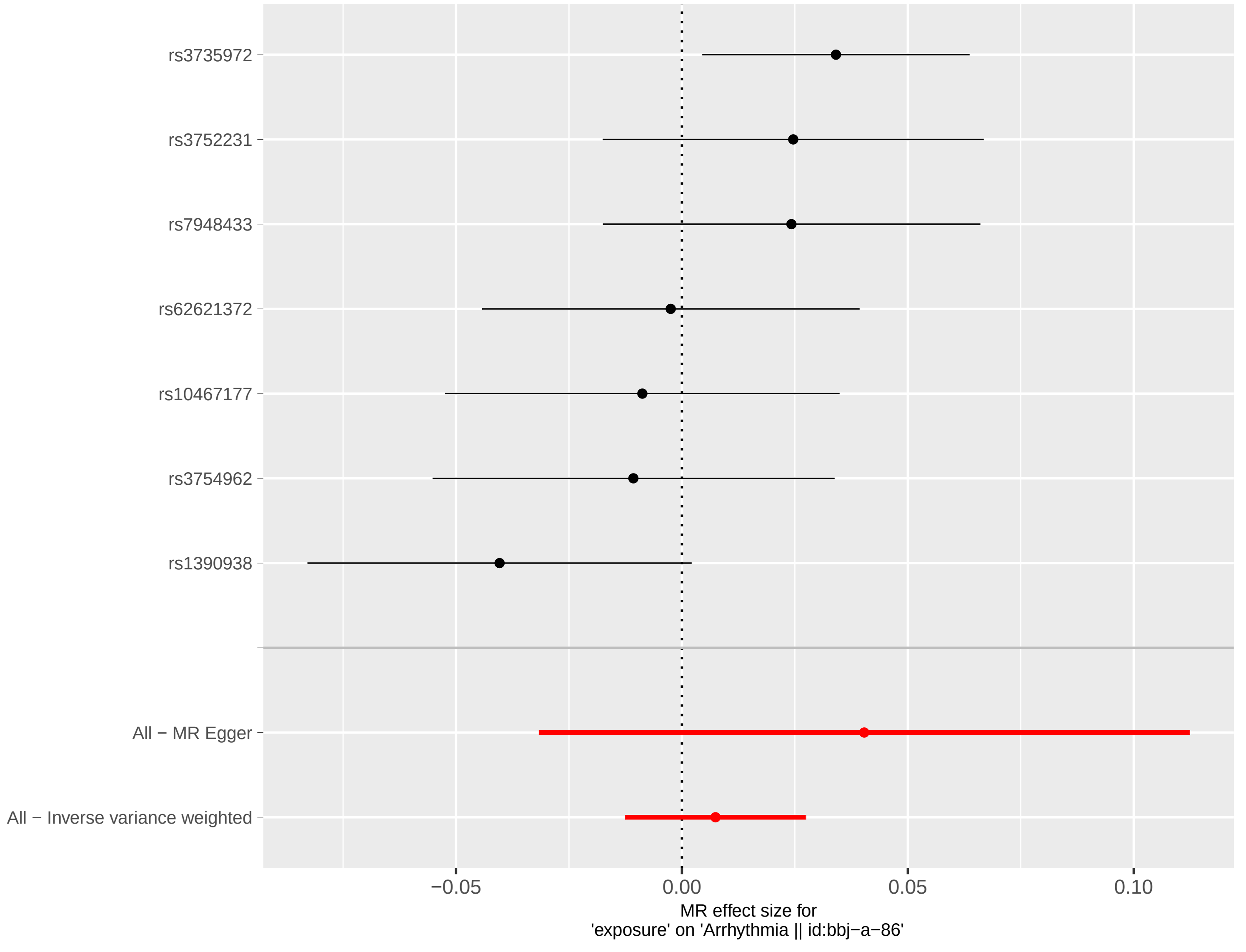
rs3735972
rs3752231
rs7948433
rs62621372
rs10467177
rs3754962
rs1390938
All − MR Egger
All − Inverse variance weighted
−0.05
0.00
0.05
0.10
MR effect size for
'exposure' on 'Arrhythmia || id:bbj−a−86'

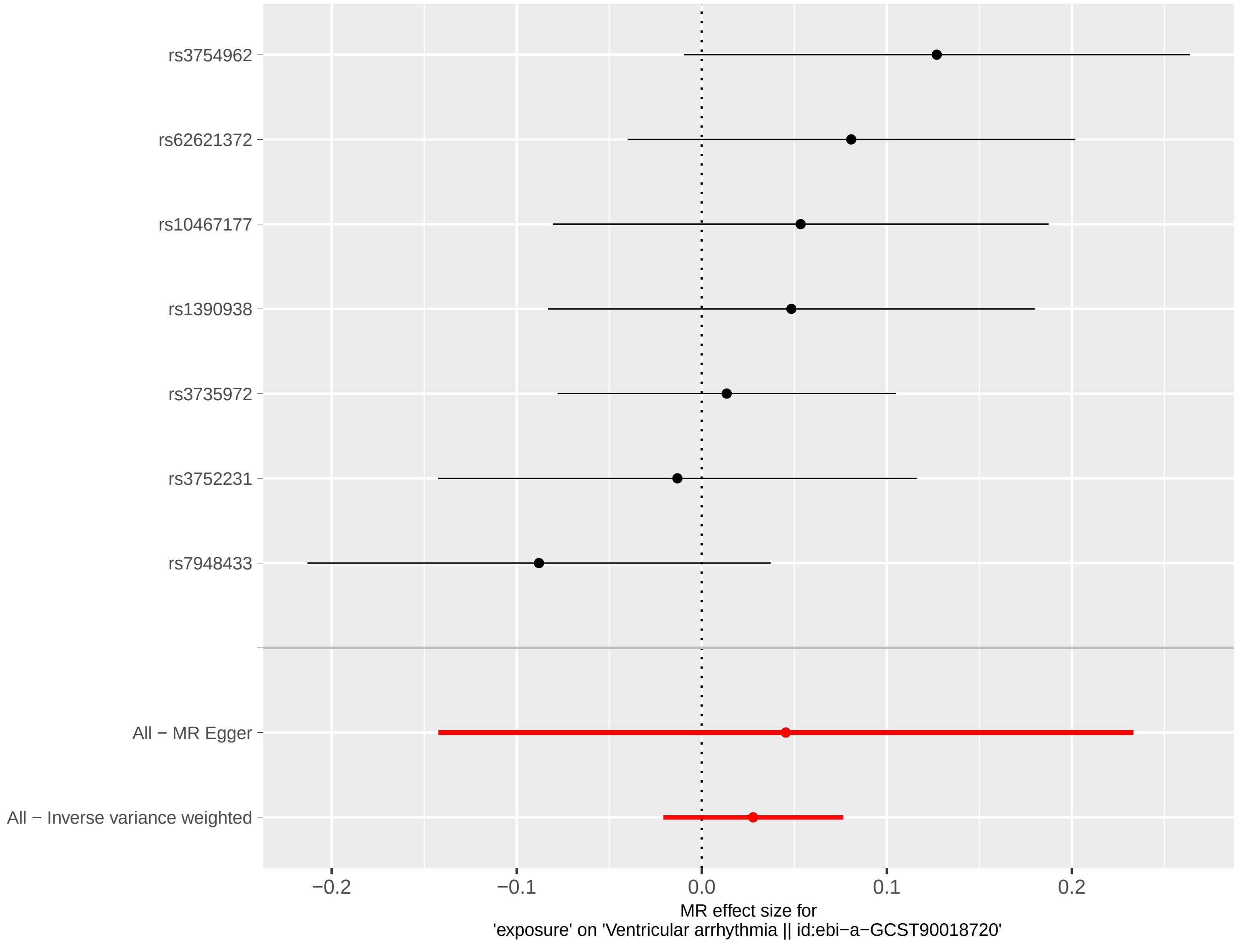

rs3754962
rs62621372
rs10467177
rs1390938
rs3735972
rs3752231
rs7948433
All − MR Egger
All − Inverse variance weighted
−0.2
−0.1
0.0
0.1
0.2
MR effect size for
'exposure' on 'Ventricular arrhythmia || id:ebi−a−GCST90018720'

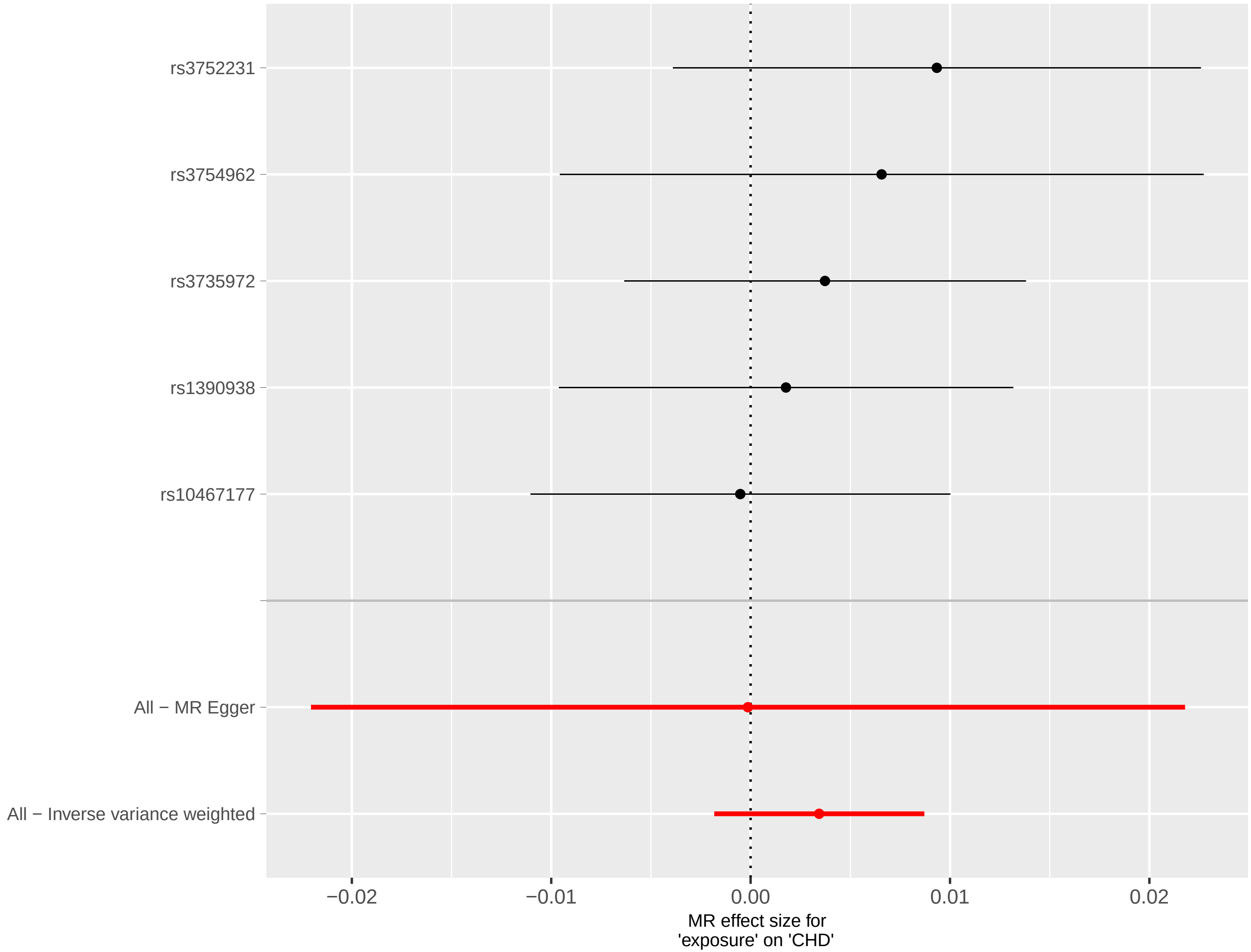
rs3752231
rs3754962
rs3735972
rs1390938
rs10467177
All − MR Egger
All − Inverse variance weighted
−0.02
−0.01
0.00
0.01
0.02
MR effect size for
'exposure' on 'CHD'

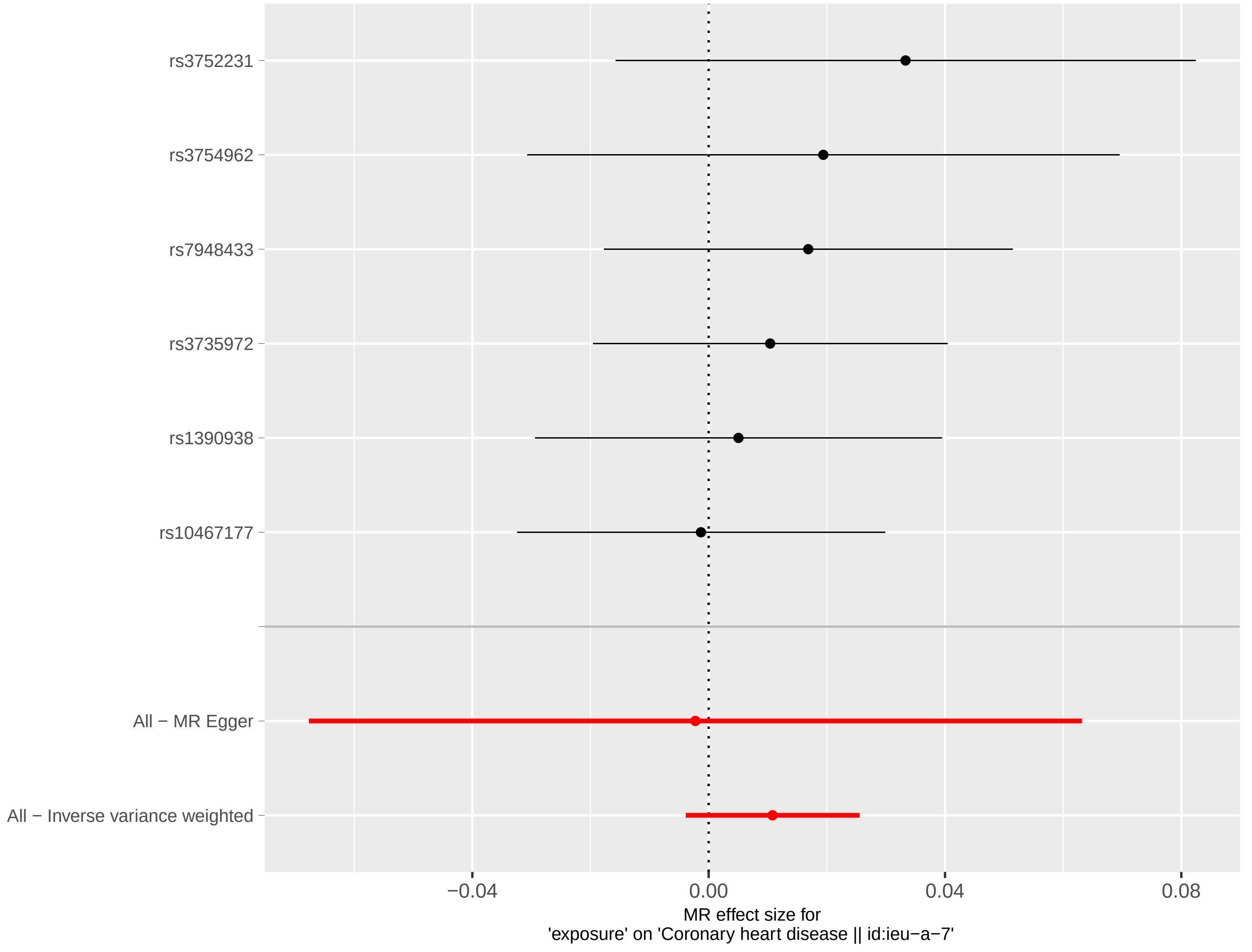

rs3752231
rs3754962
rs7948433
rs3735972
rs1390938
rs10467177
All − MR Egger
All − Inverse variance weighted
−0.04
0.00
0.04
0.08
MR effect size for
'exposure' on 'Coronary heart disease || id:ieu−a−7'

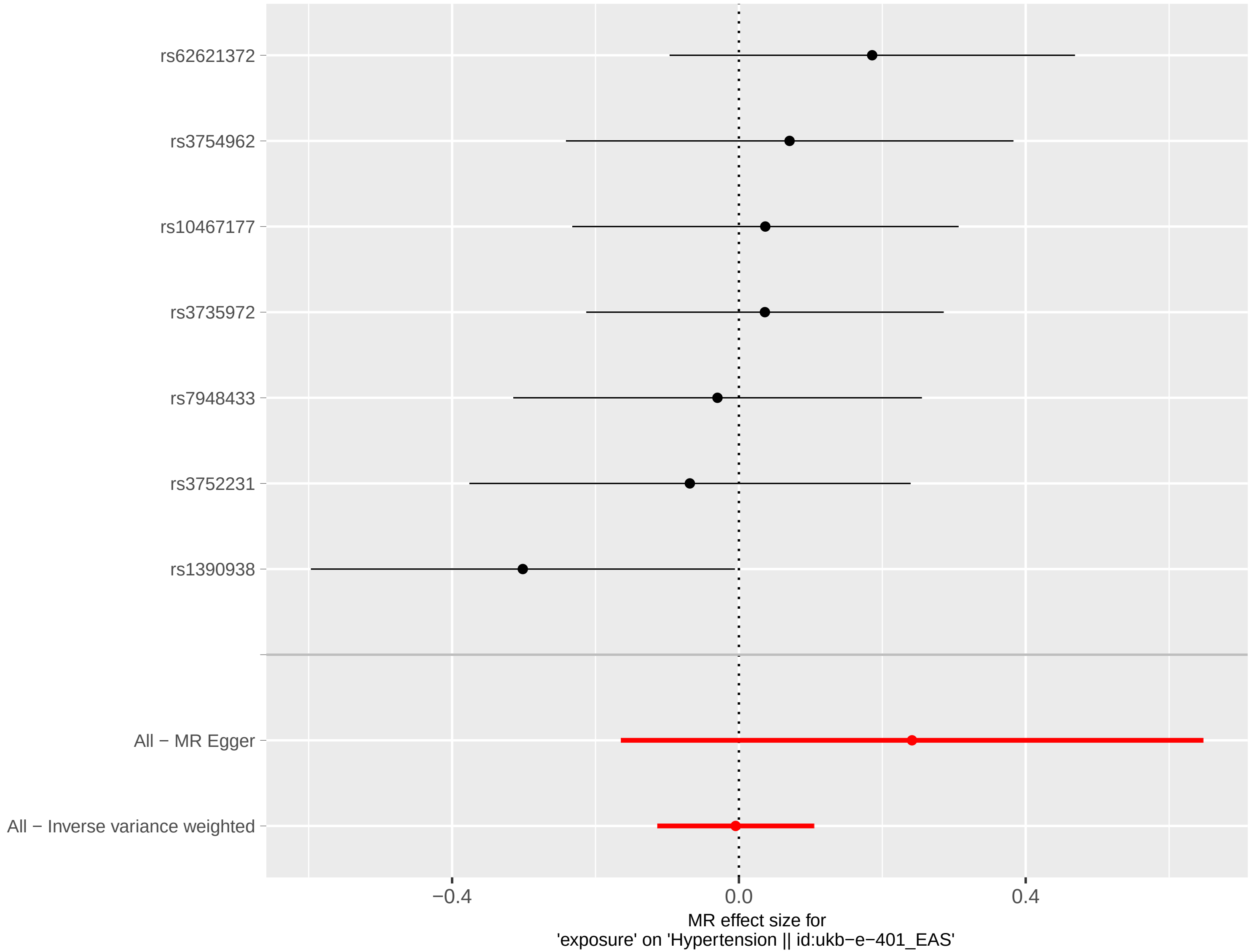

rs62621372
rs3754962
rs10467177
rs3735972
rs7948433
rs3752231
rs1390938
All − MR Egger
All − Inverse variance weighted
−0.4
0.0
0.4
MR effect size for
'exposure' on 'Hypertension || id:ukb−e−401_EAS'

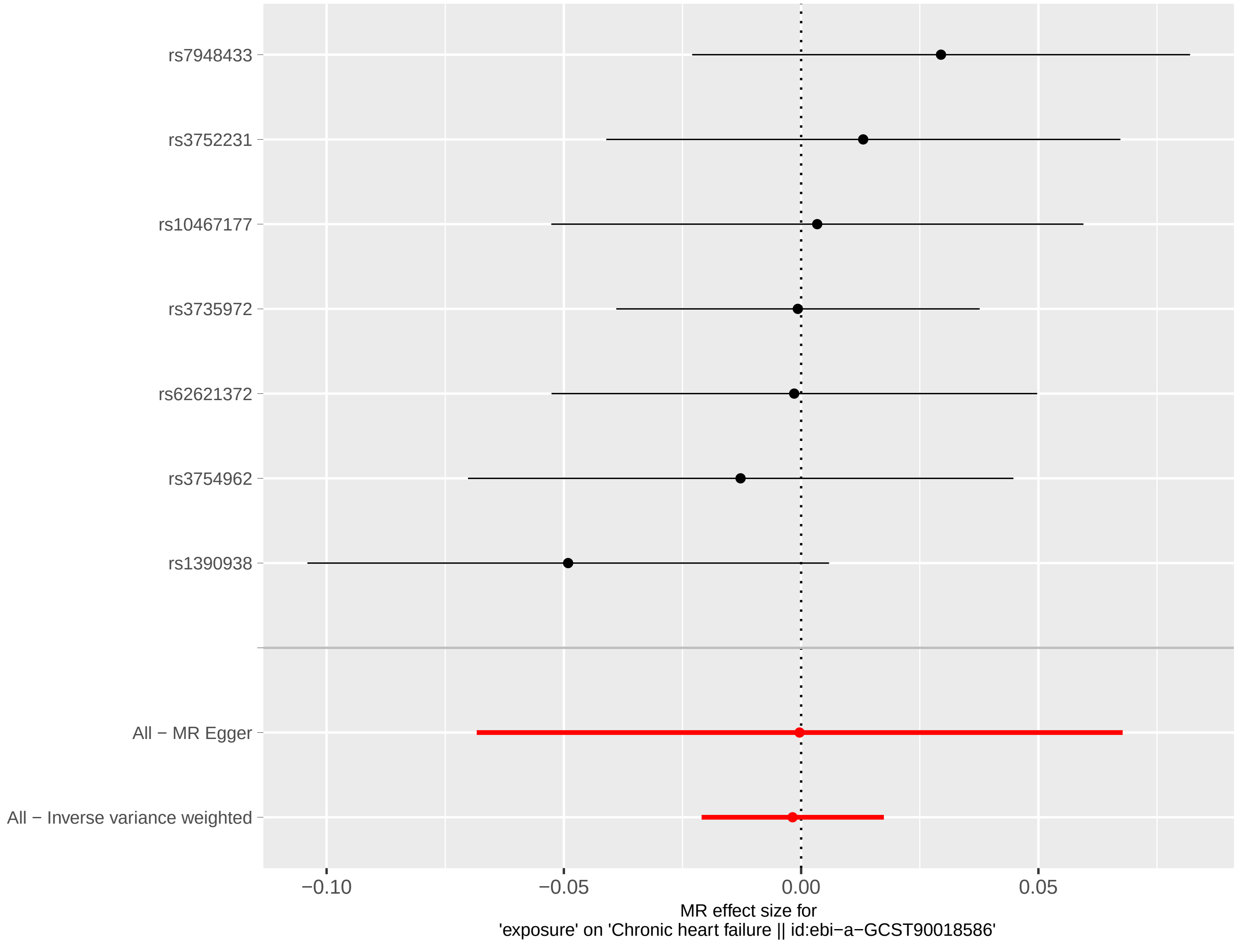
rs7948433
rs3752231
rs10467177
rs3735972
rs62621372
rs3754962
rs1390938
All − MR Egger
All − Inverse variance weighted
−0.10
−0.05
0.00
0.05
MR effect size for
'exposure' on 'Chronic heart failure || id:ebi−a−GCST90018586'

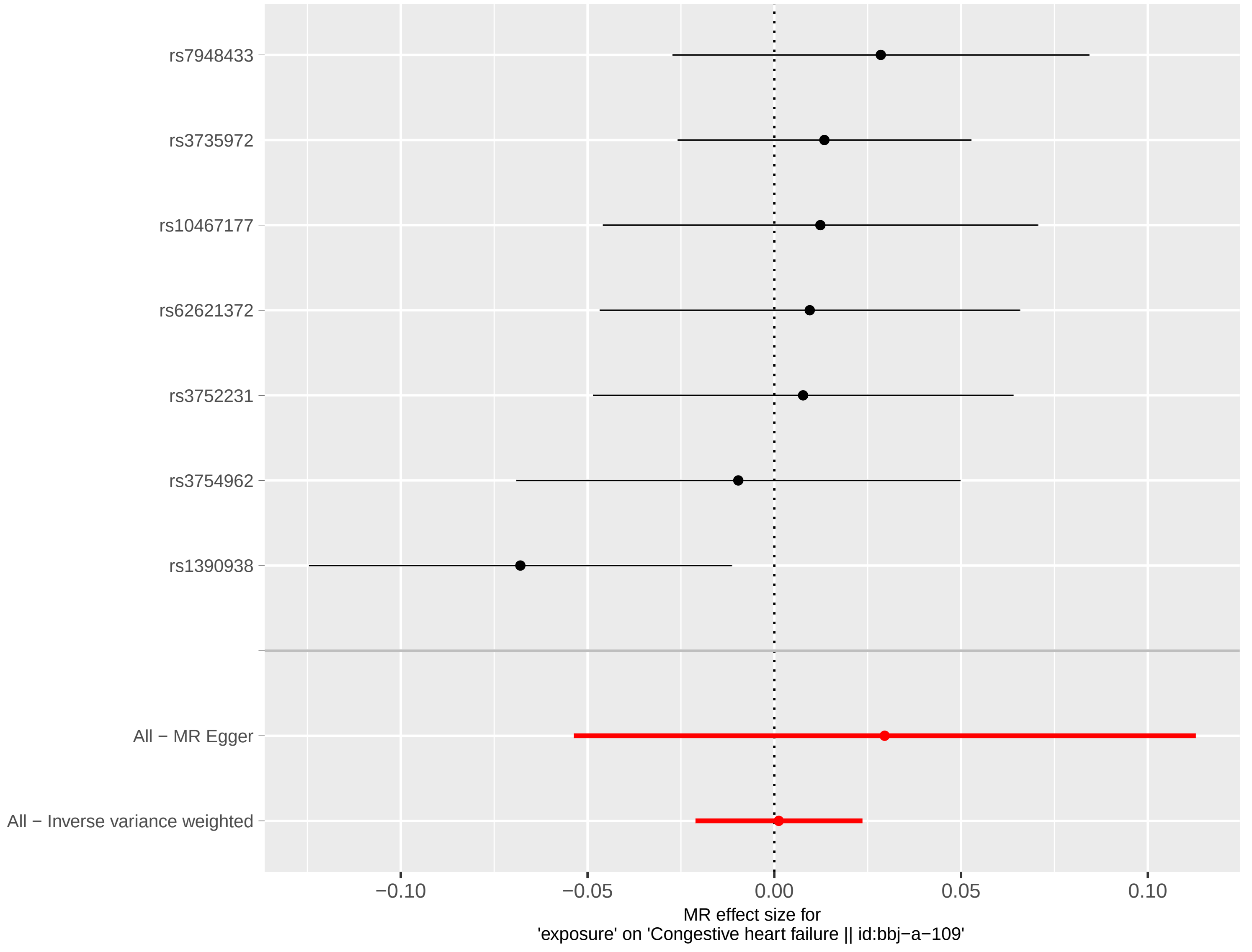
rs7948433
rs3735972
rs10467177
rs62621372
rs3752231
rs3754962
rs1390938
All − MR Egger
All − Inverse variance weighted
−0.10
−0.05
0.00
0.05
0.10
MR effect size for
'exposure' on 'Congestive heart failure || id:bbj−a−109'

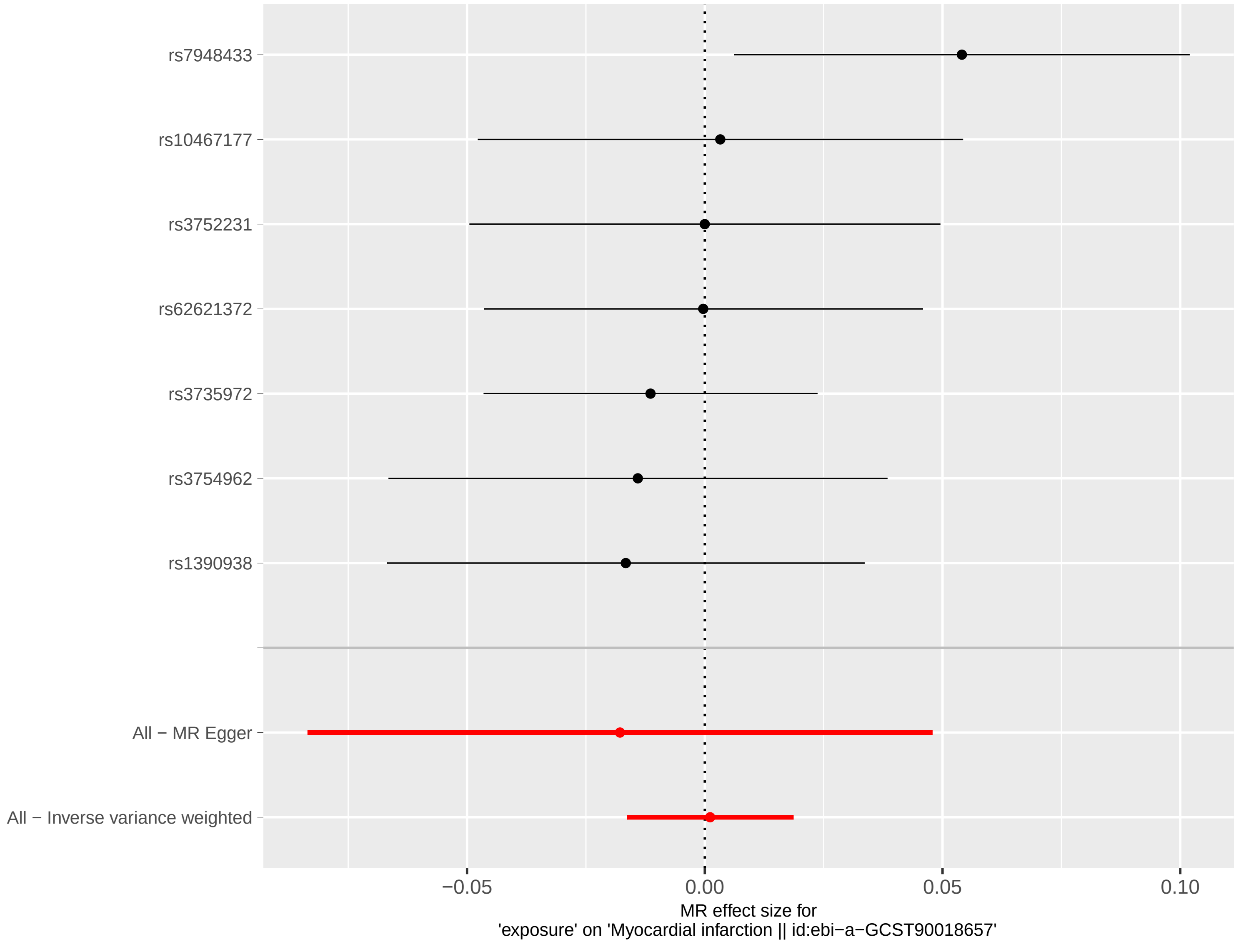
rs7948433
rs10467177
rs3752231
rs62621372
rs3735972
rs3754962
rs1390938
All − MR Egger
All − Inverse variance weighted
−0.05
0.00
0.05
0.10
MR effect size for
'exposure' on 'Myocardial infarction || id:ebi−a−GCST90018657'
