# Supplementary material for: Genetic liability to childhood Kawasaki disease and adult cardiovascular outcomes
Source: Front Cardiovasc Med. 2026 Jul 23;13:1848320. doi: 10.3389/fcvm.2026.1848320 (PMC13441699; doi:10.3389/fcvm.2026.1848320)
Supplement: Supplementary file 2 [file Datasheet2.pdf]

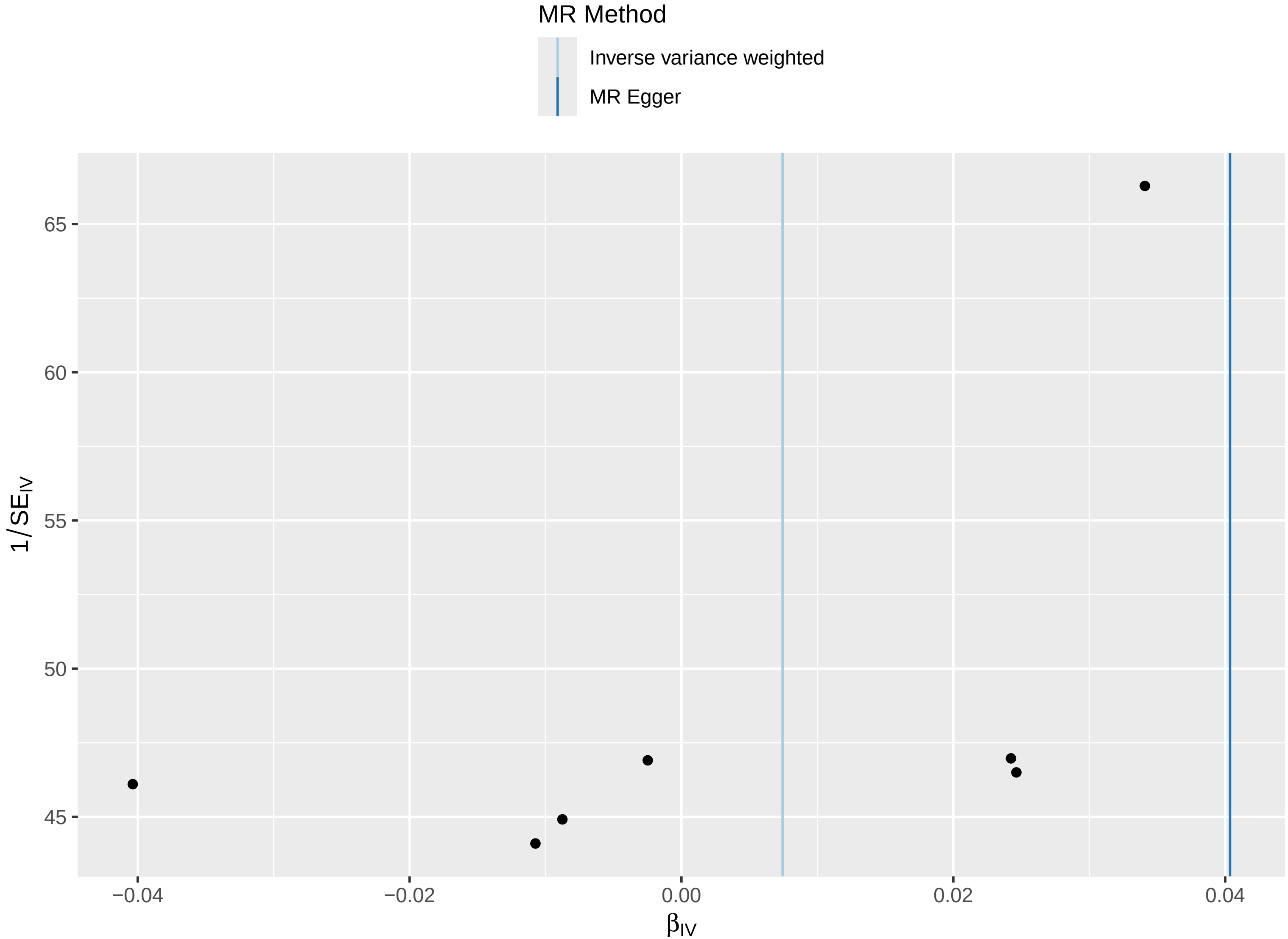

MR Method
Inverse variance weighted
MR Egger
65
60
55
50
45
1/SE_IV
−0.04
−0.02
0.00
0.02
0.04
β_IV

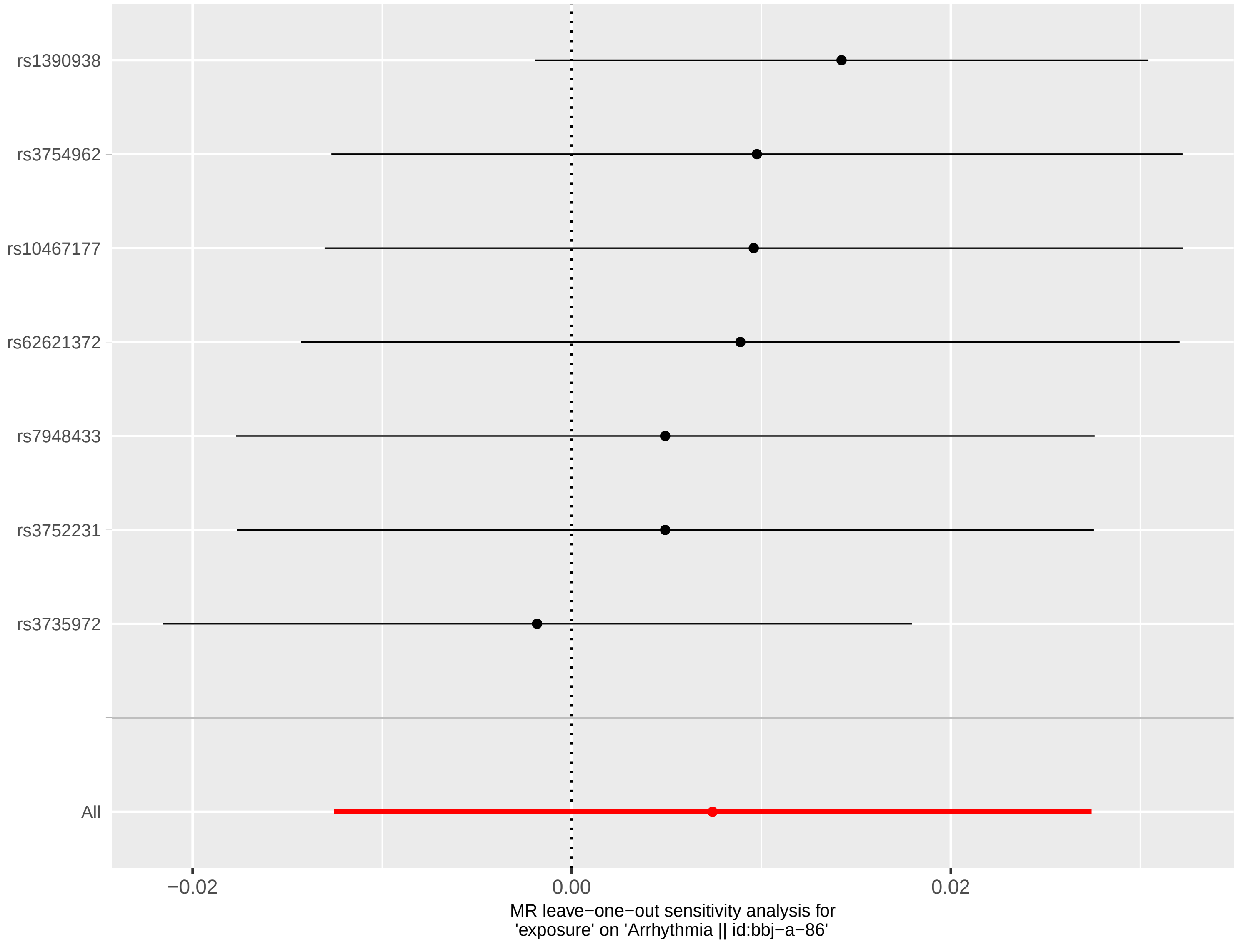
rs1390938
rs3754962
rs10467177
rs62621372
rs7948433
rs3752231
rs3735972
All
−0.02
0.00
0.02
MR leave−one−out sensitivity analysis for
'exposure' on 'Arrhythmia || id:bbj−a−86'

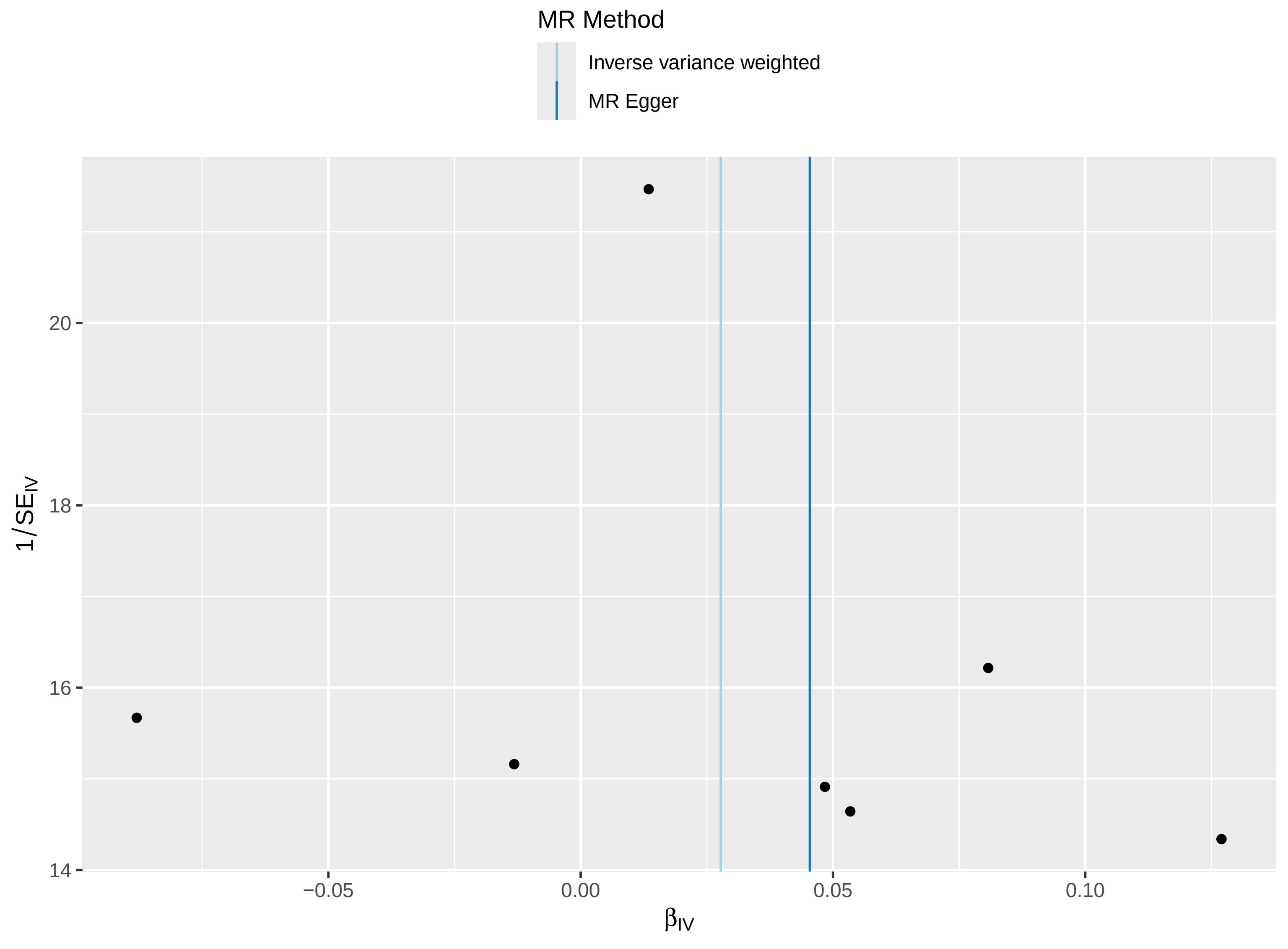
MR Method
Inverse variance weighted
MR Egger
20
18
16
14
1/SE_IV
−0.05
0.00
0.05
0.10
β_IV

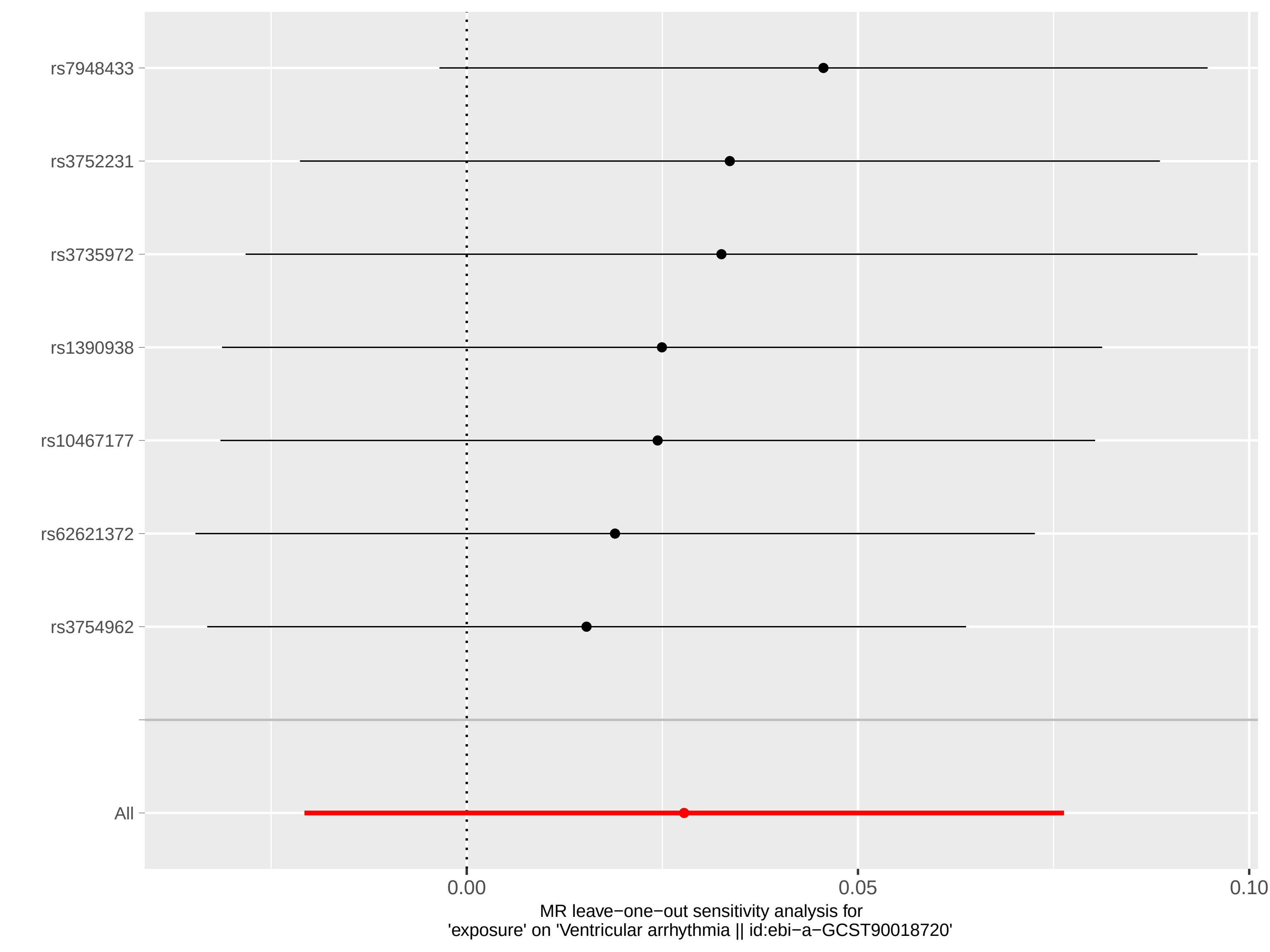

rs7948433
rs3752231
rs3735972
rs1390938
rs10467177
rs62621372
rs3754962
All
0.00
0.05
0.10
MR leave−one−out sensitivity analysis for
'exposure' on 'Ventricular arrhythmia || id:ebi−a−GCST90018720'

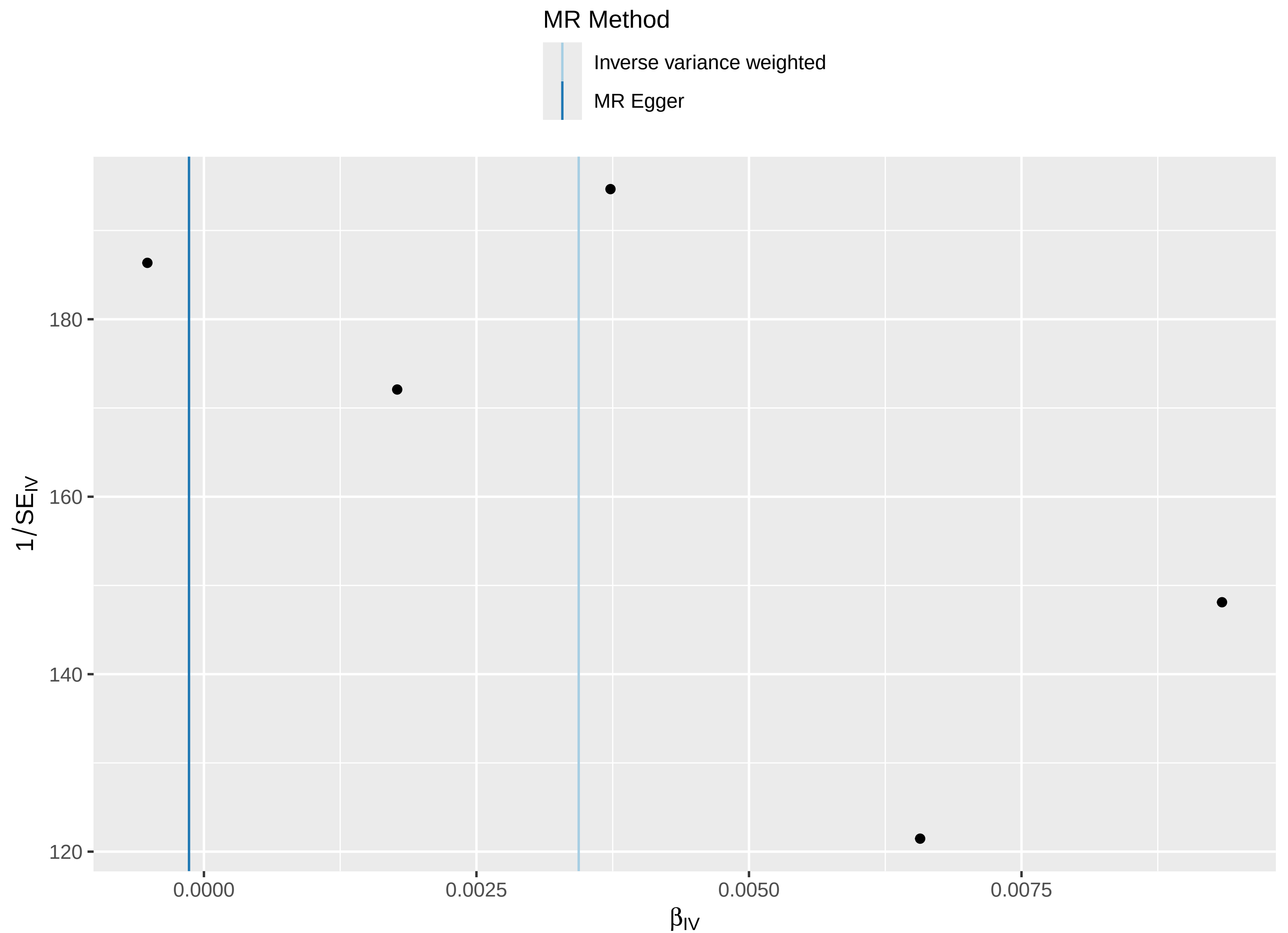

MR Method
Inverse variance weighted
MR Egger
1/SE_IV
180
160
140
120
0.0000
0.0025
0.0050
0.0075
β_IV

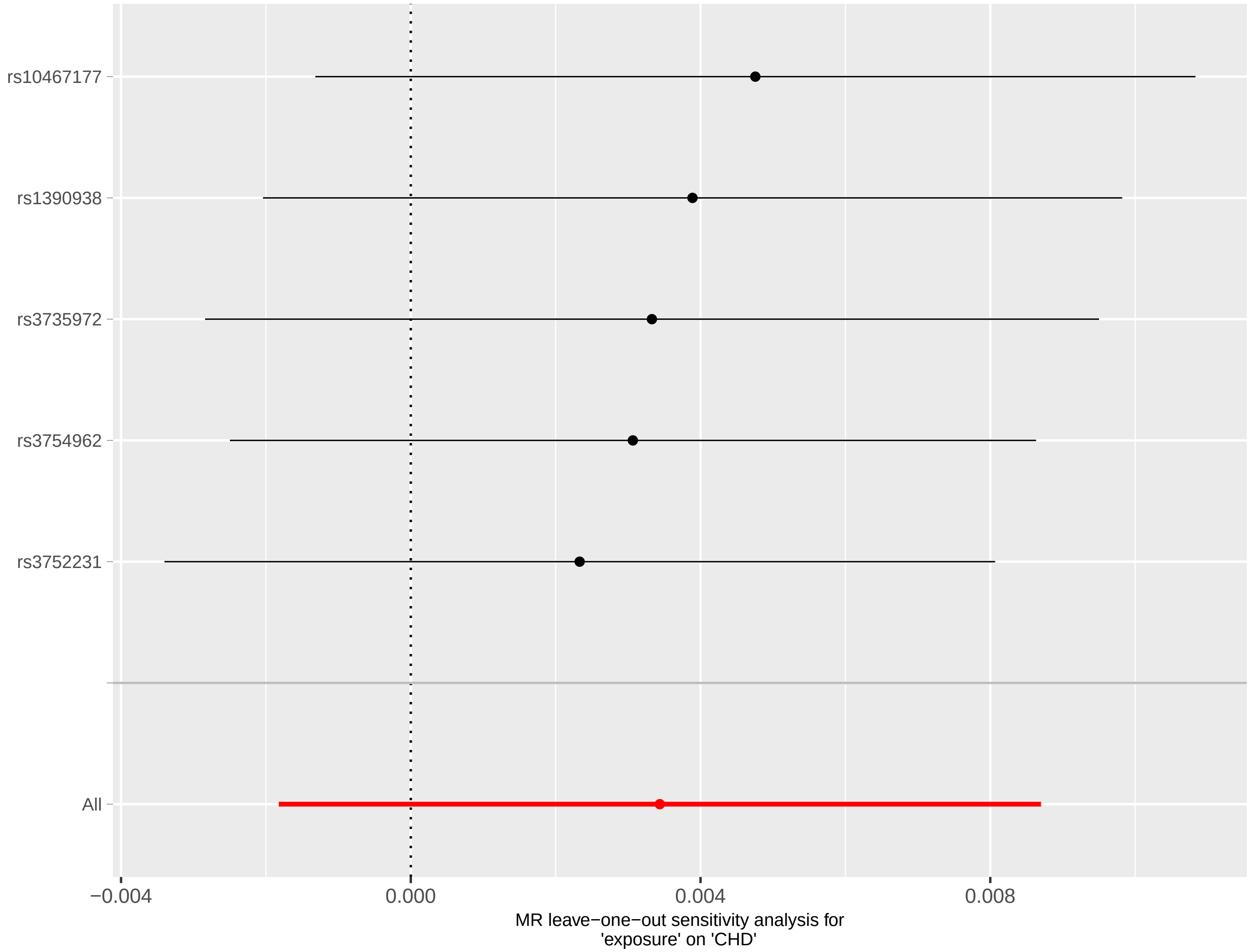

rs10467177
rs1390938
rs3735972
rs3754962
rs3752231
All
−0.004
0.000
0.004
0.008
MR leave−one−out sensitivity analysis for
'exposure' on 'CHD'

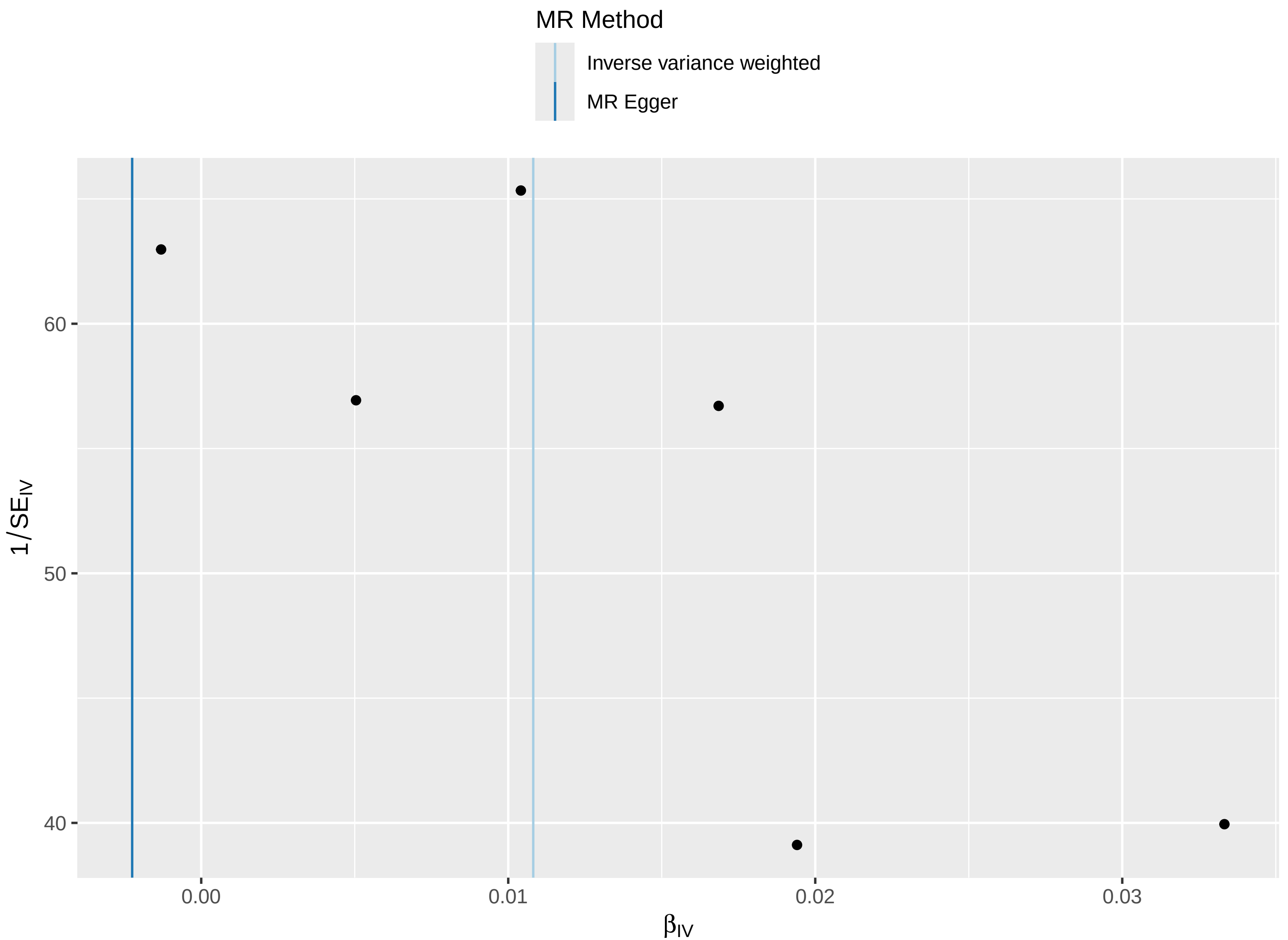
MR Method
Inverse variance weighted
MR Egger
60
50
40
1/SE$_{IV}$
0.00
0.01
0.02
0.03
β$_{IV}$

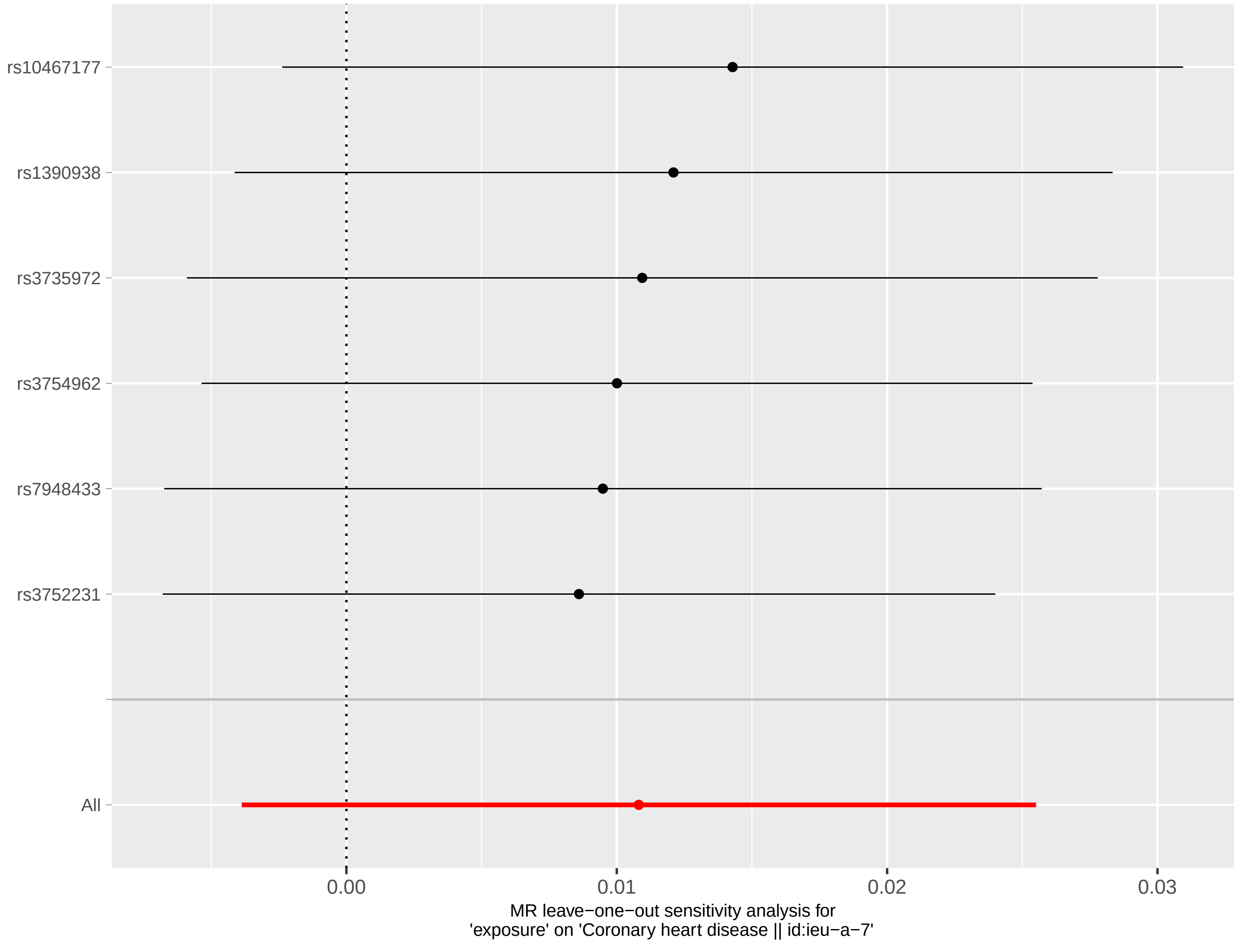
rs10467177
rs1390938
rs3735972
rs3754962
rs7948433
rs3752231
All
0.00
0.01
0.02
0.03
MR leave−one−out sensitivity analysis for
'exposure' on 'Coronary heart disease || id:ieu−a−7'

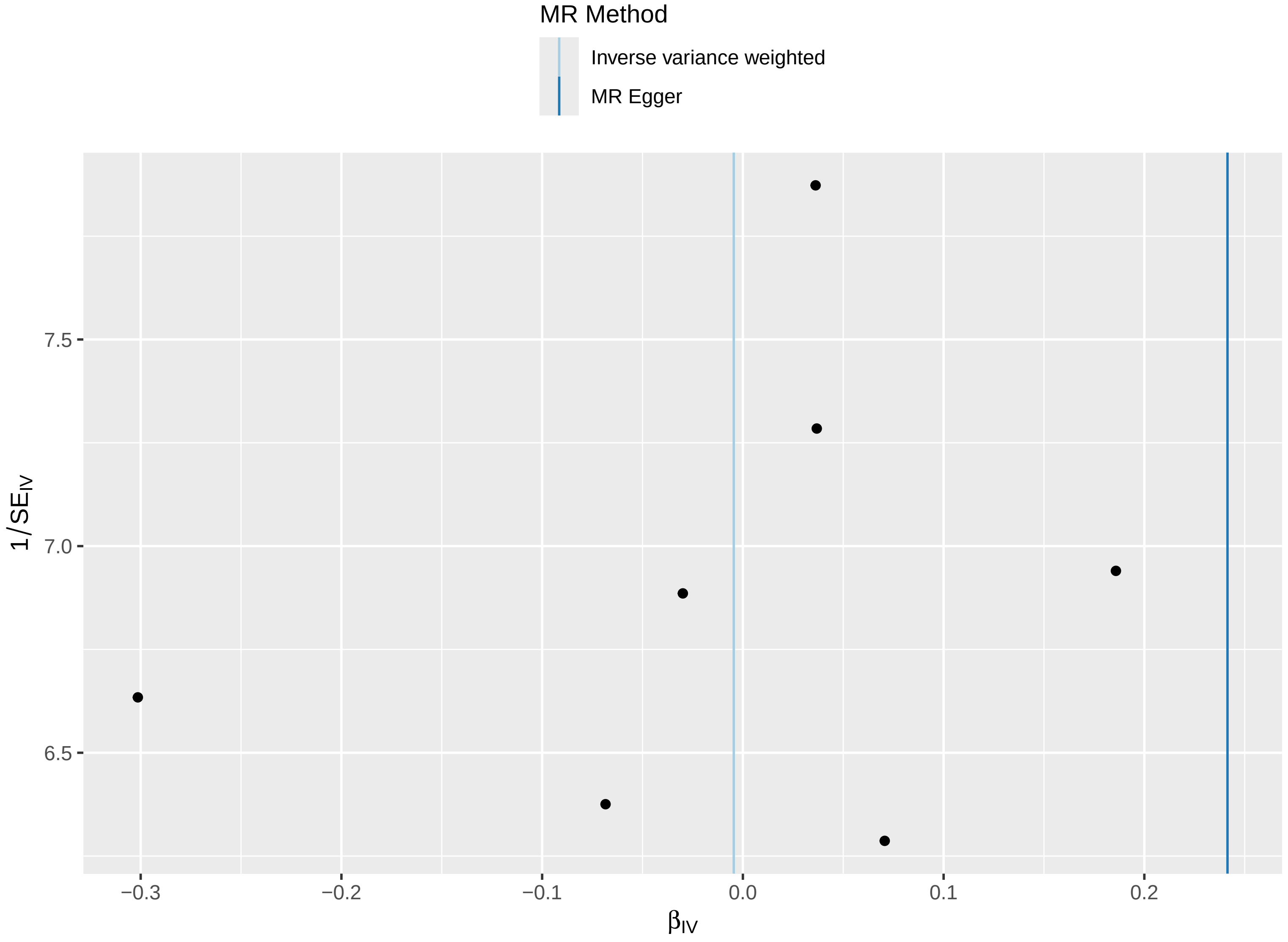
MR Method
Inverse variance weighted
MR Egger
7.5
7.0
6.5
1/SE_IV
−0.3
−0.2
−0.1
0.0
0.1
0.2
β_IV

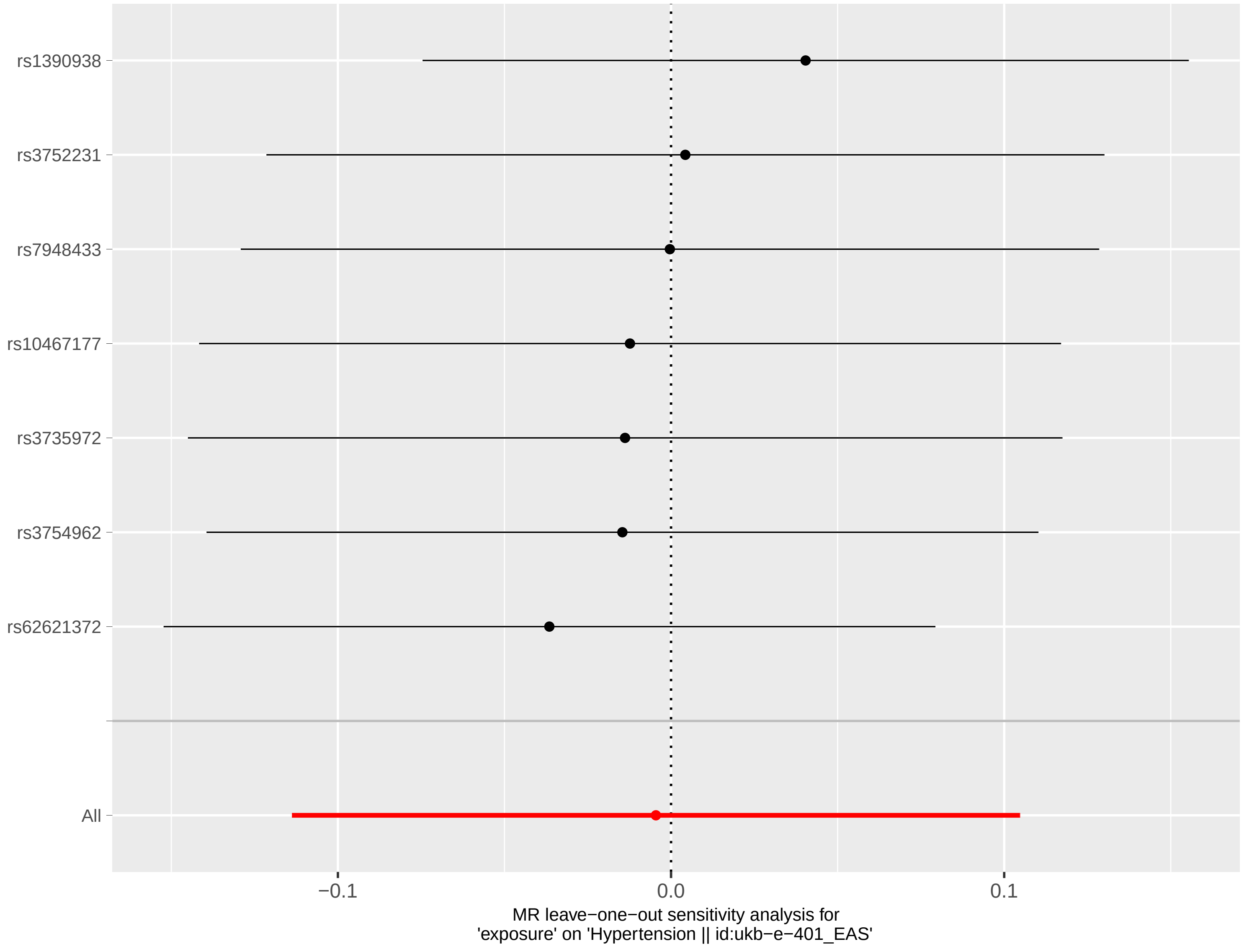
rs1390938
rs3752231
rs7948433
rs10467177
rs3735972
rs3754962
rs62621372
All
−0.1
0.0
0.1
MR leave−one−out sensitivity analysis for
'exposure' on 'Hypertension || id:ukb−e−401_EAS'

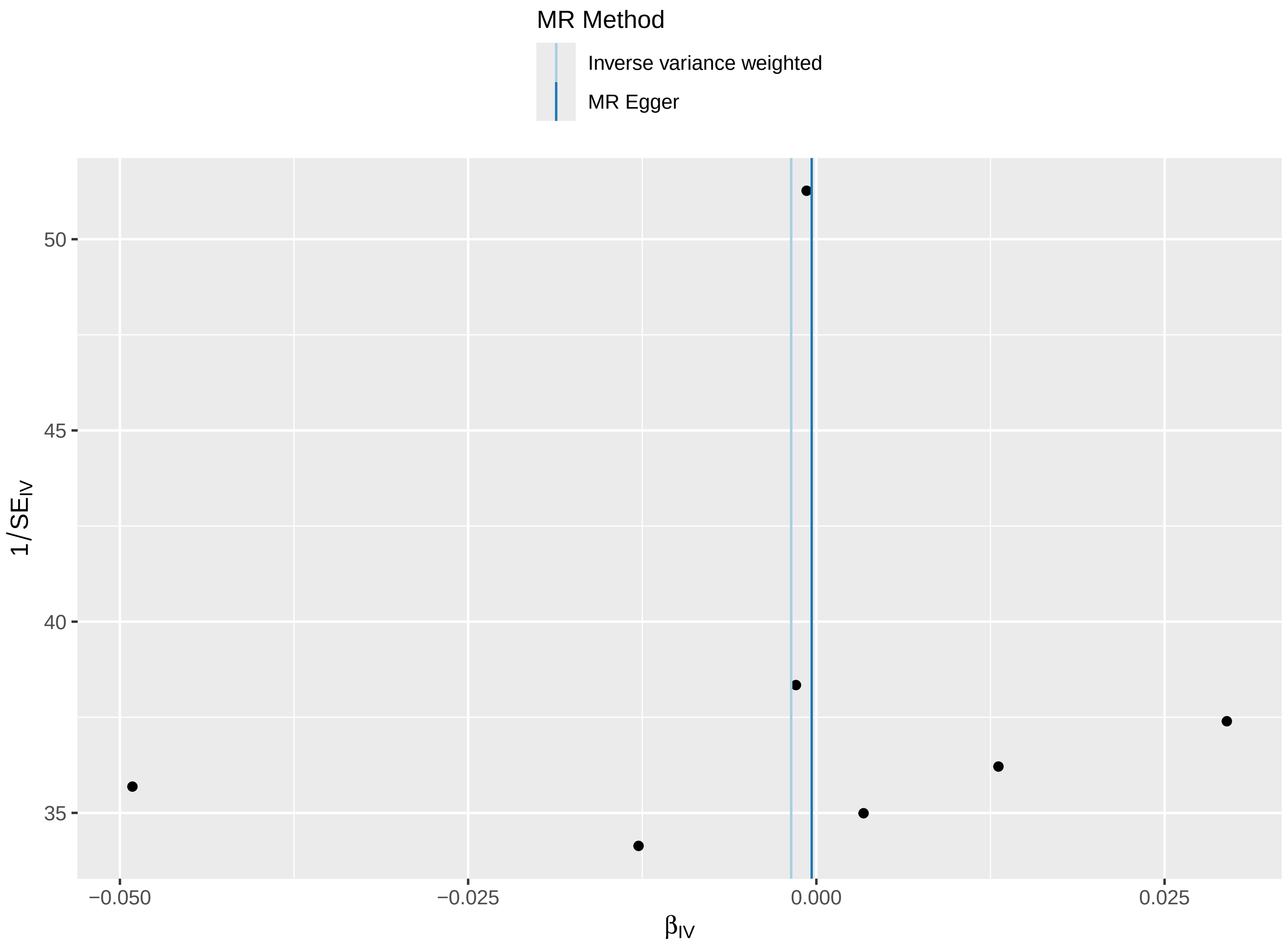
MR Method
Inverse variance weighted
MR Egger
50
45
40
35
1/SE_IV
−0.050
−0.025
0.000
0.025
β_IV

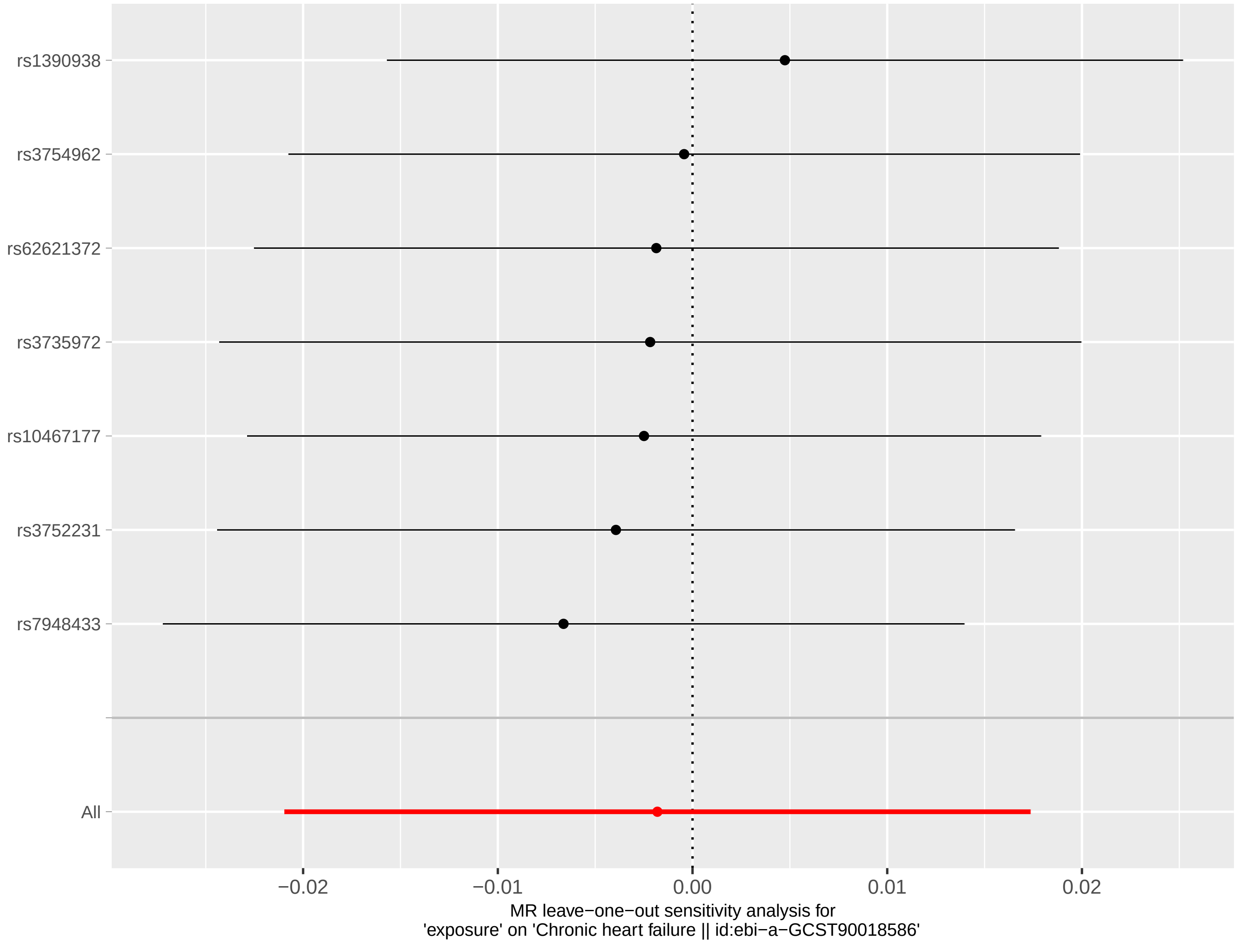
rs1390938
rs3754962
rs62621372
rs3735972
rs10467177
rs3752231
rs7948433
All
−0.02
−0.01
0.00
0.01
0.02
MR leave−one−out sensitivity analysis for
'exposure' on 'Chronic heart failure || id:ebi−a−GCST90018586'

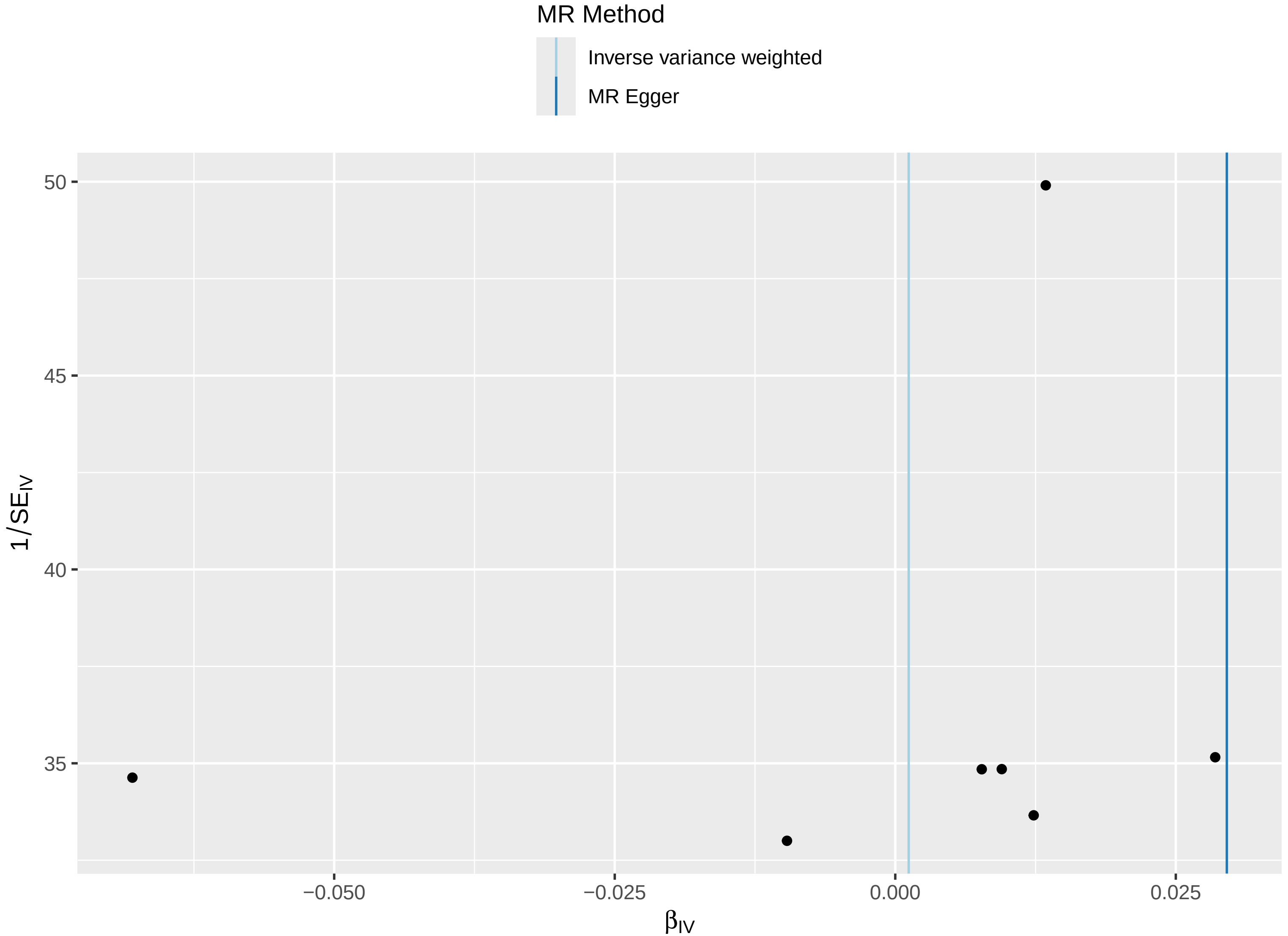

MR Method
Inverse variance weighted
MR Egger
50
45
40
35
1/SE_IV
−0.050
−0.025
0.000
0.025
β_IV

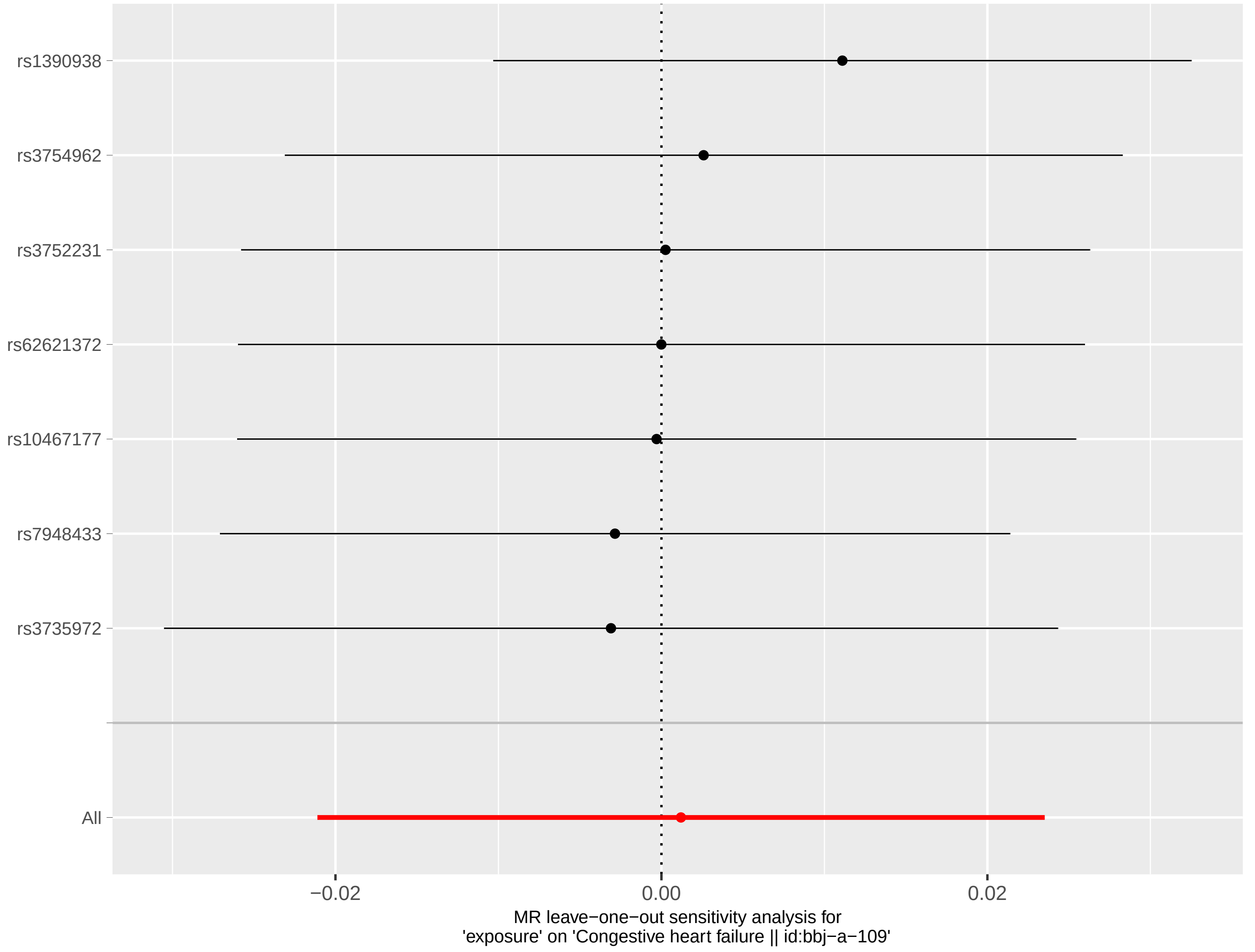
rs1390938
rs3754962
rs3752231
rs62621372
rs10467177
rs7948433
rs3735972
All
−0.02
0.00
0.02
MR leave−one−out sensitivity analysis for
'exposure' on 'Congestive heart failure || id:bbj−a−109'

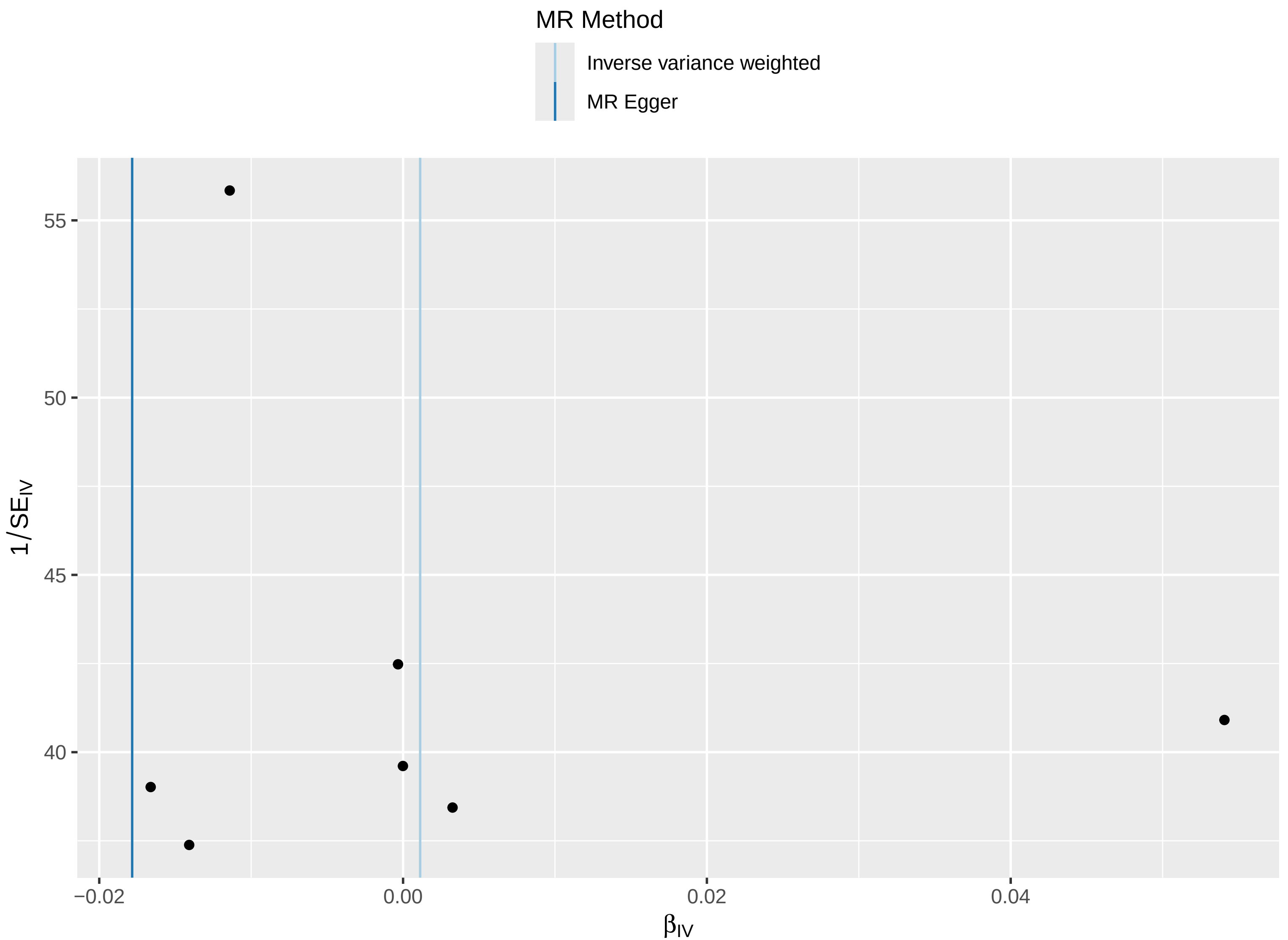
MR Method
Inverse variance weighted
MR Egger
55
50
45
40
1/SE_IV
−0.02
0.00
0.02
0.04
β_IV

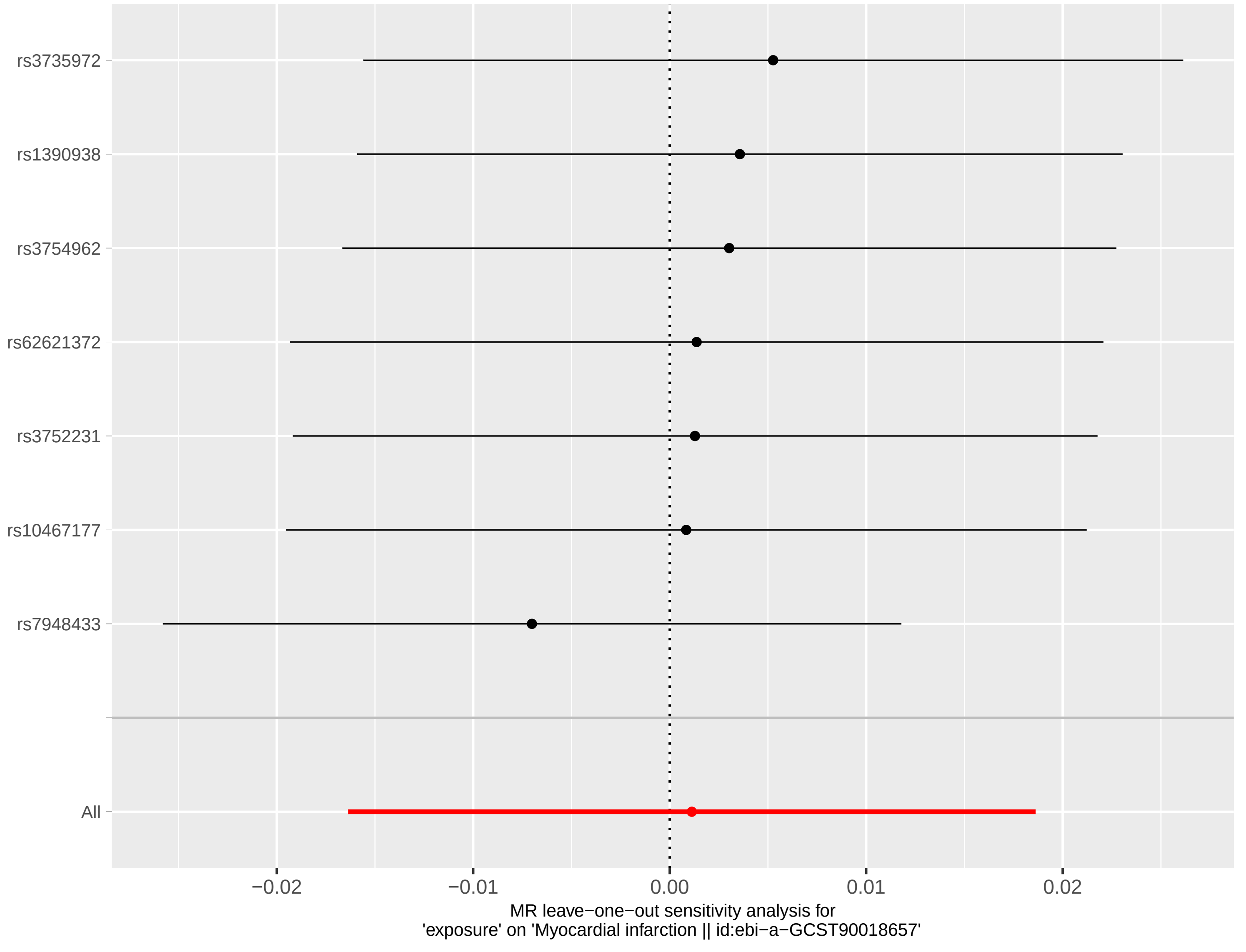

rs3735972
rs1390938
rs3754962
rs62621372
rs3752231
rs10467177
rs7948433
All
−0.02
−0.01
0.00
0.01
0.02
MR leave−one−out sensitivity analysis for
'exposure' on 'Myocardial infarction || id:ebi−a−GCST90018657'
