# Supplementary material for: Genetic liability to childhood Kawasaki disease and adult cardiovascular outcomes
Source: Front Cardiovasc Med. 2026 Jul 23;13:1848320. doi: 10.3389/fcvm.2026.1848320 (PMC13441699; doi:10.3389/fcvm.2026.1848320)
Supplement: Supplementary file 3 [file Datasheet3.pdf]

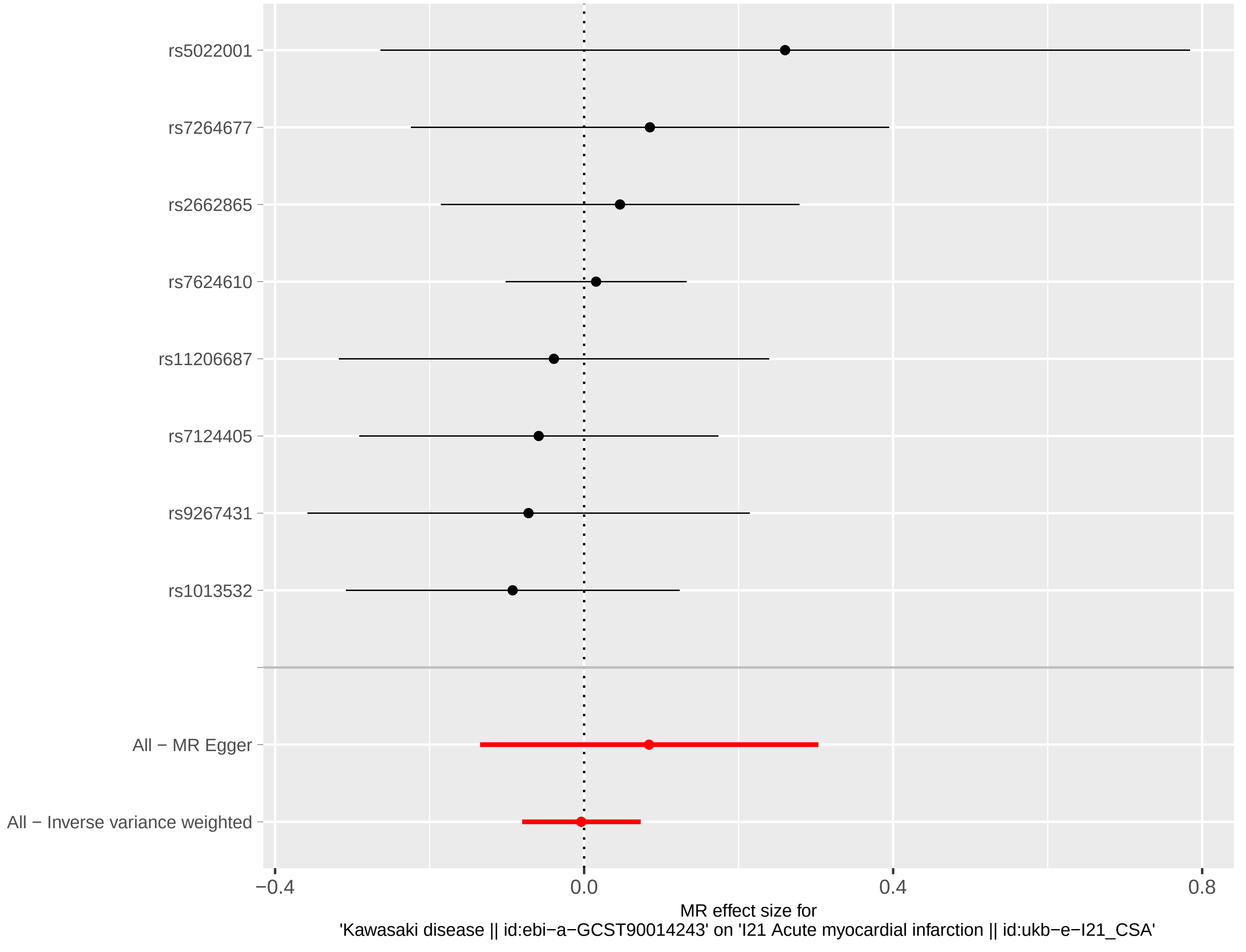
rs5022001
rs7264677
rs2662865
rs7624610
rs11206687
rs7124405
rs9267431
rs1013532
All − MR Egger
All − Inverse variance weighted
−0.4
0.0
0.4
0.8
MR effect size for
'Kawasaki disease || id:ebi−a−GCST90014243' on 'I21 Acute myocardial infarction || id:ukb−e−I21_CSA'

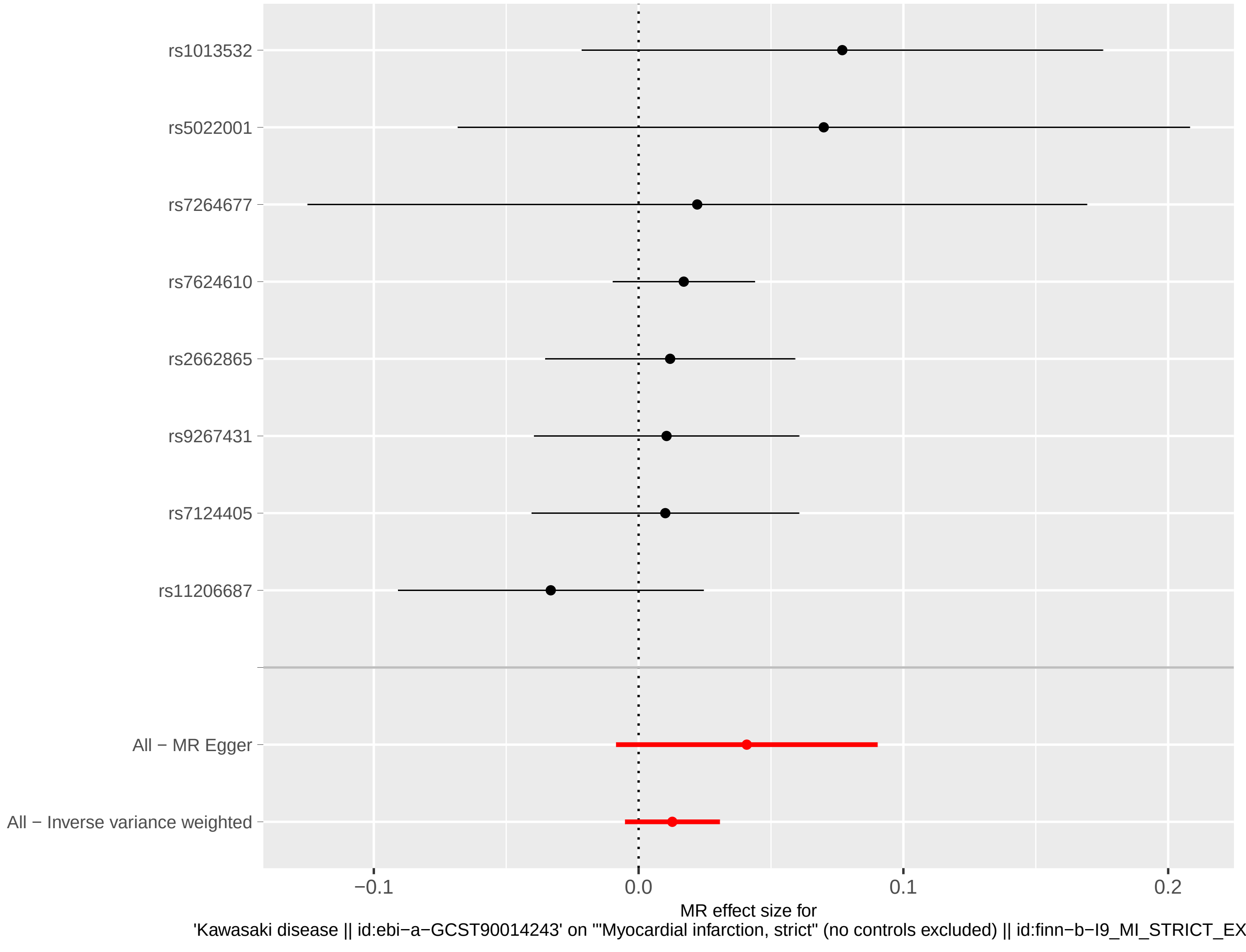
rs1013532
rs5022001
rs7264677
rs7624610
rs2662865
rs9267431
rs7124405
rs11206687
All − MR Egger
All − Inverse variance weighted
−0.1
0.0
0.1
0.2
MR effect size for
'Kawasaki disease || id:ebi−a−GCST90014243' on '"Myocardial infarction, strict" (no controls excluded) || id:finn−b−I9_MI_STRICT_EX

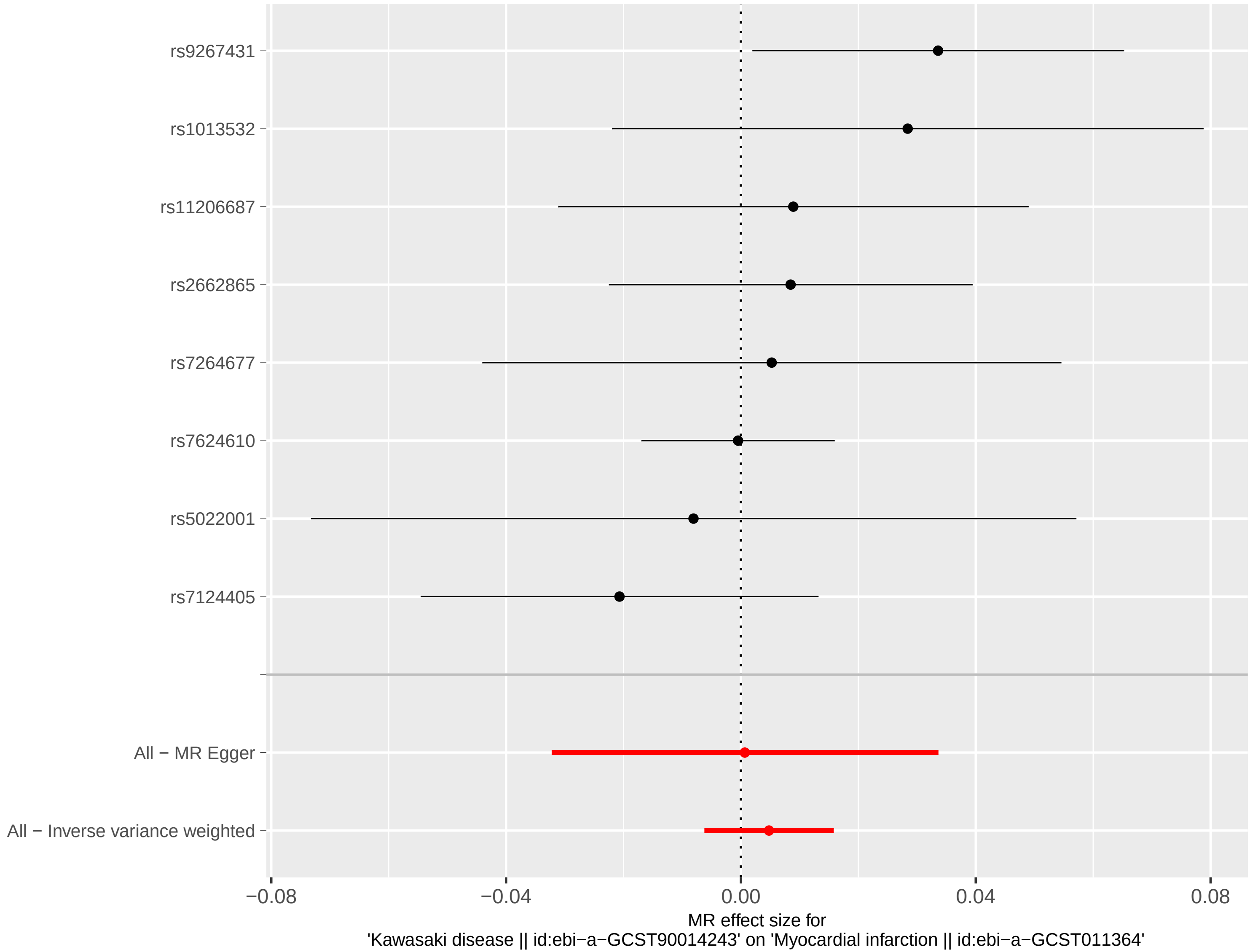

rs9267431
rs1013532
rs11206687
rs2662865
rs7264677
rs7624610
rs5022001
rs7124405
All − MR Egger
All − Inverse variance weighted
−0.08
−0.04
0.00
0.04
0.08
MR effect size for
'Kawasaki disease || id:ebi−a−GCST90014243' on 'Myocardial infarction || id:ebi−a−GCST011364'

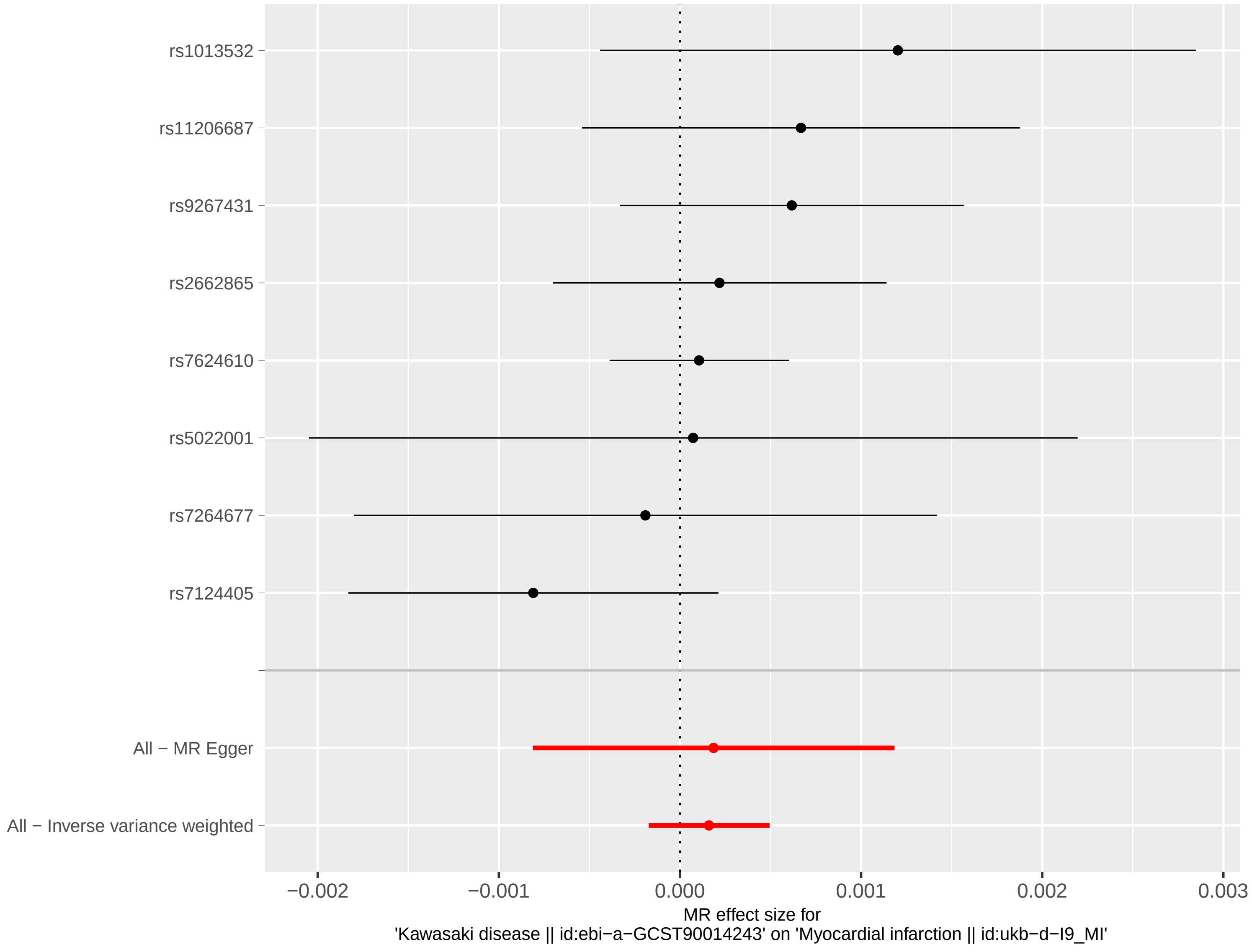

rs1013532
rs11206687
rs9267431
rs2662865
rs7624610
rs5022001
rs7264677
rs7124405
All − MR Egger
All − Inverse variance weighted
−0.002
−0.001
0.000
0.001
0.002
0.003
MR effect size for
'Kawasaki disease || id:ebi−a−GCST90014243' on 'Myocardial infarction || id:ukb−d−I9_MI'

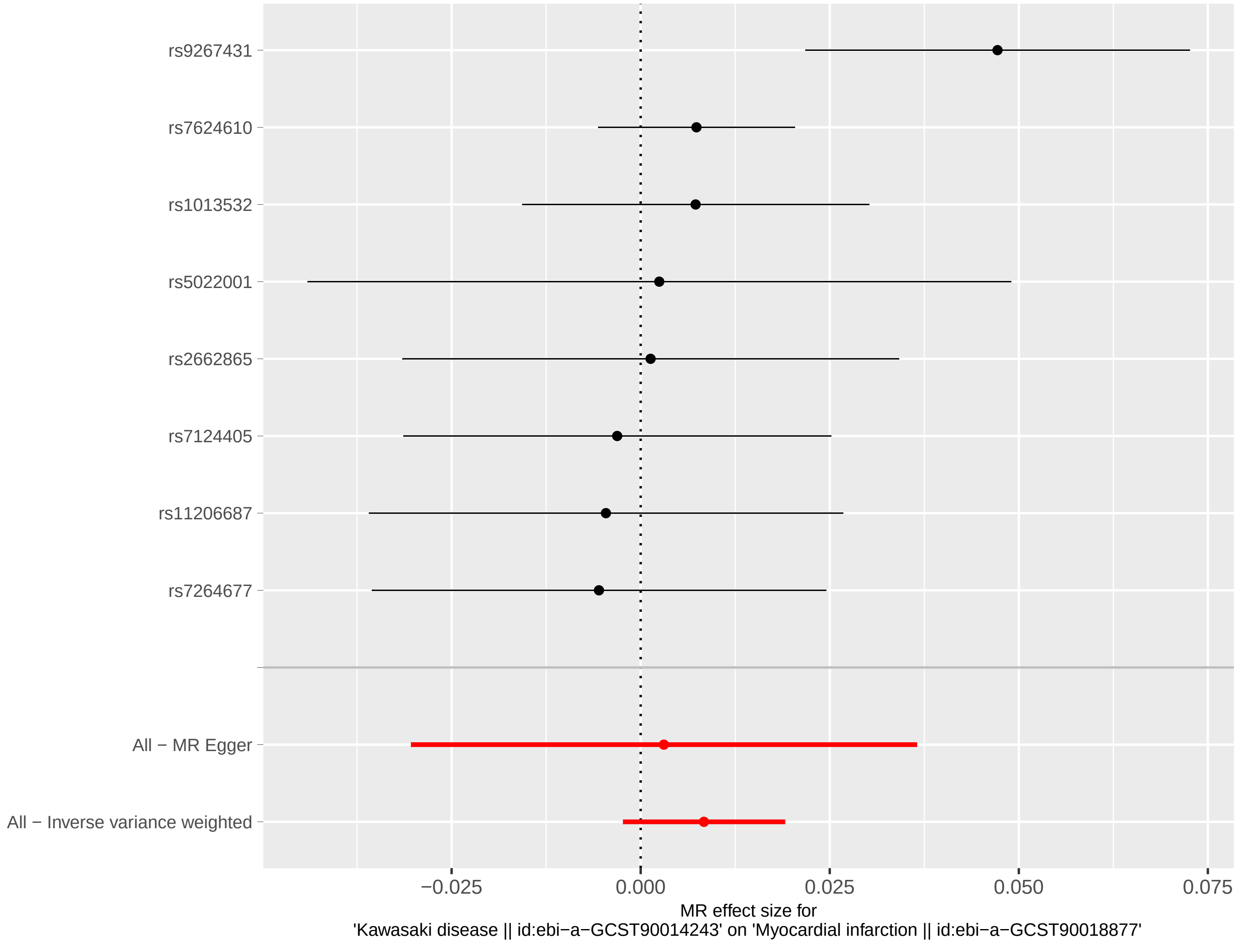

rs9267431
rs7624610
rs1013532
rs5022001
rs2662865
rs7124405
rs11206687
rs7264677
All − MR Egger
All − Inverse variance weighted
−0.025
0.000
0.025
0.050
0.075
MR effect size for
'Kawasaki disease || id:ebi−a−GCST90014243' on 'Myocardial infarction || id:ebi−a−GCST90018877'

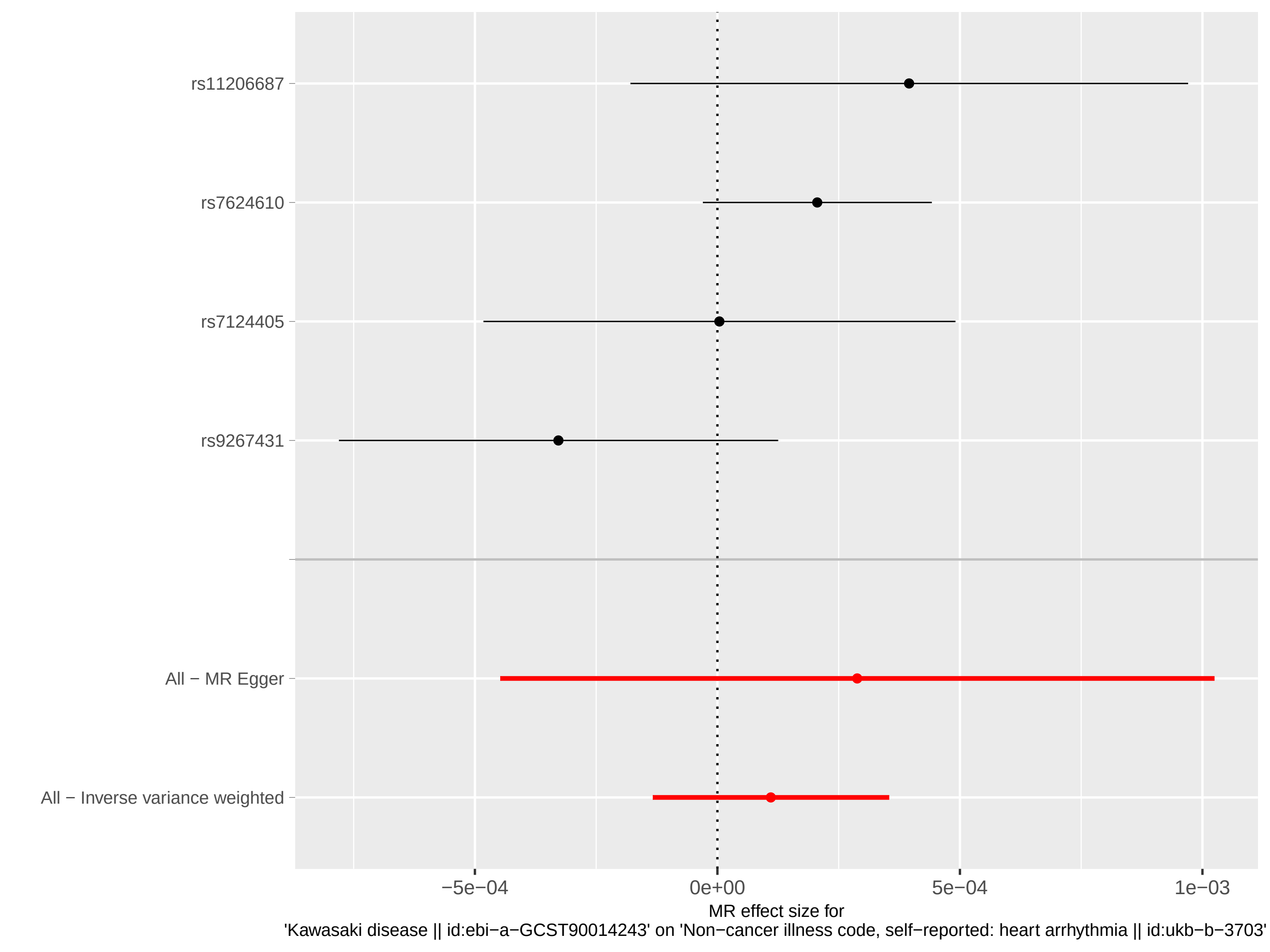
rs11206687
rs7624610
rs7124405
rs9267431
All − MR Egger
All − Inverse variance weighted
−5e−04
0e+00
5e−04
1e−03
MR effect size for
'Kawasaki disease || id:ebi−a−GCST90014243' on 'Non−cancer illness code, self−reported: heart arrhythmia || id:ukb−b−3703'

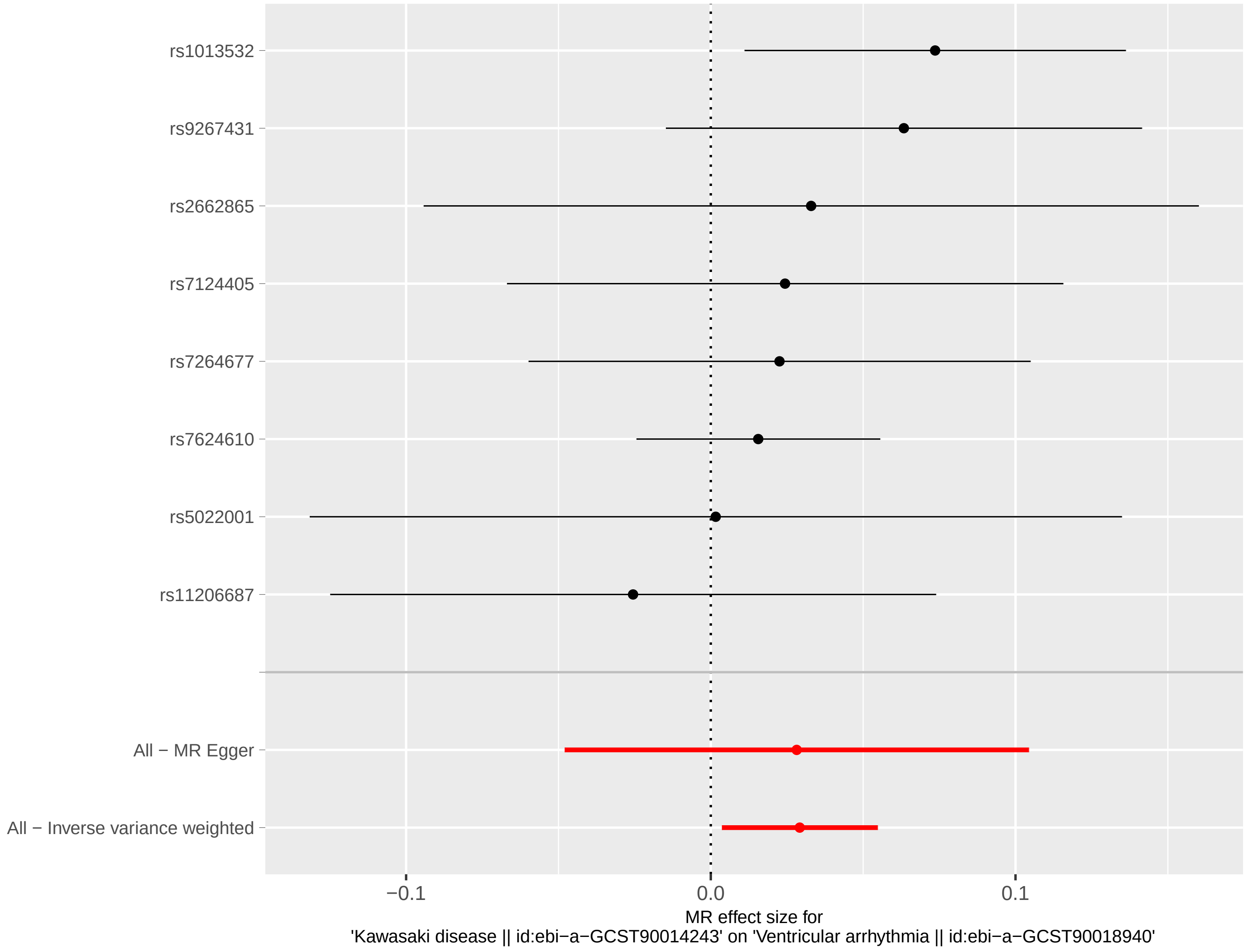

rs1013532
rs9267431
rs2662865
rs7124405
rs7264677
rs7624610
rs5022001
rs11206687
All − MR Egger
All − Inverse variance weighted
−0.1
0.0
0.1
MR effect size for
'Kawasaki disease || id:ebi−a−GCST90014243' on 'Ventricular arrhythmia || id:ebi−a−GCST90018940'

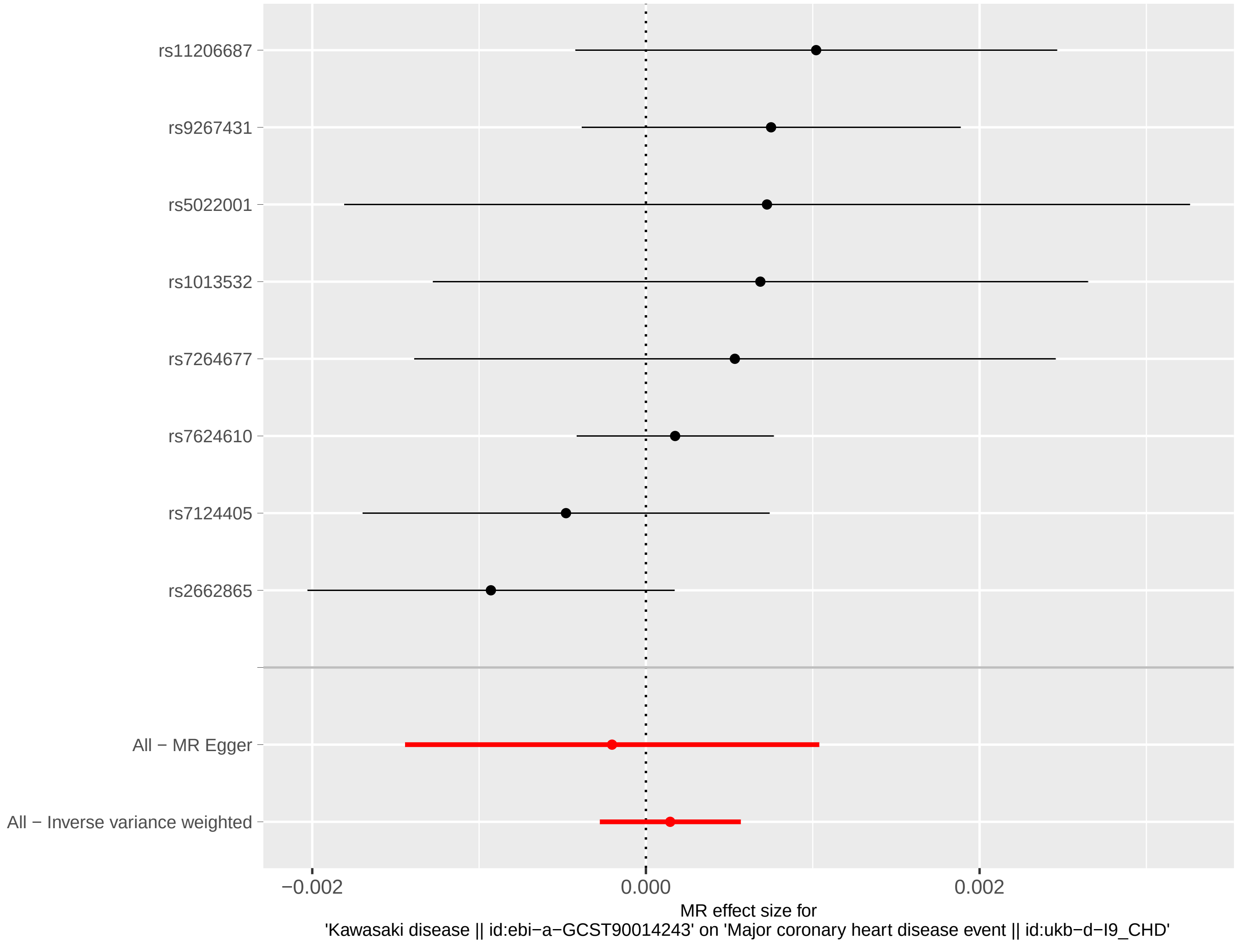

rs11206687
rs9267431
rs5022001
rs1013532
rs7264677
rs7624610
rs7124405
rs2662865
All − MR Egger
All − Inverse variance weighted
−0.002
0.000
0.002
MR effect size for
'Kawasaki disease || id:ebi−a−GCST90014243' on 'Major coronary heart disease event || id:ukb−d−I9_CHD'

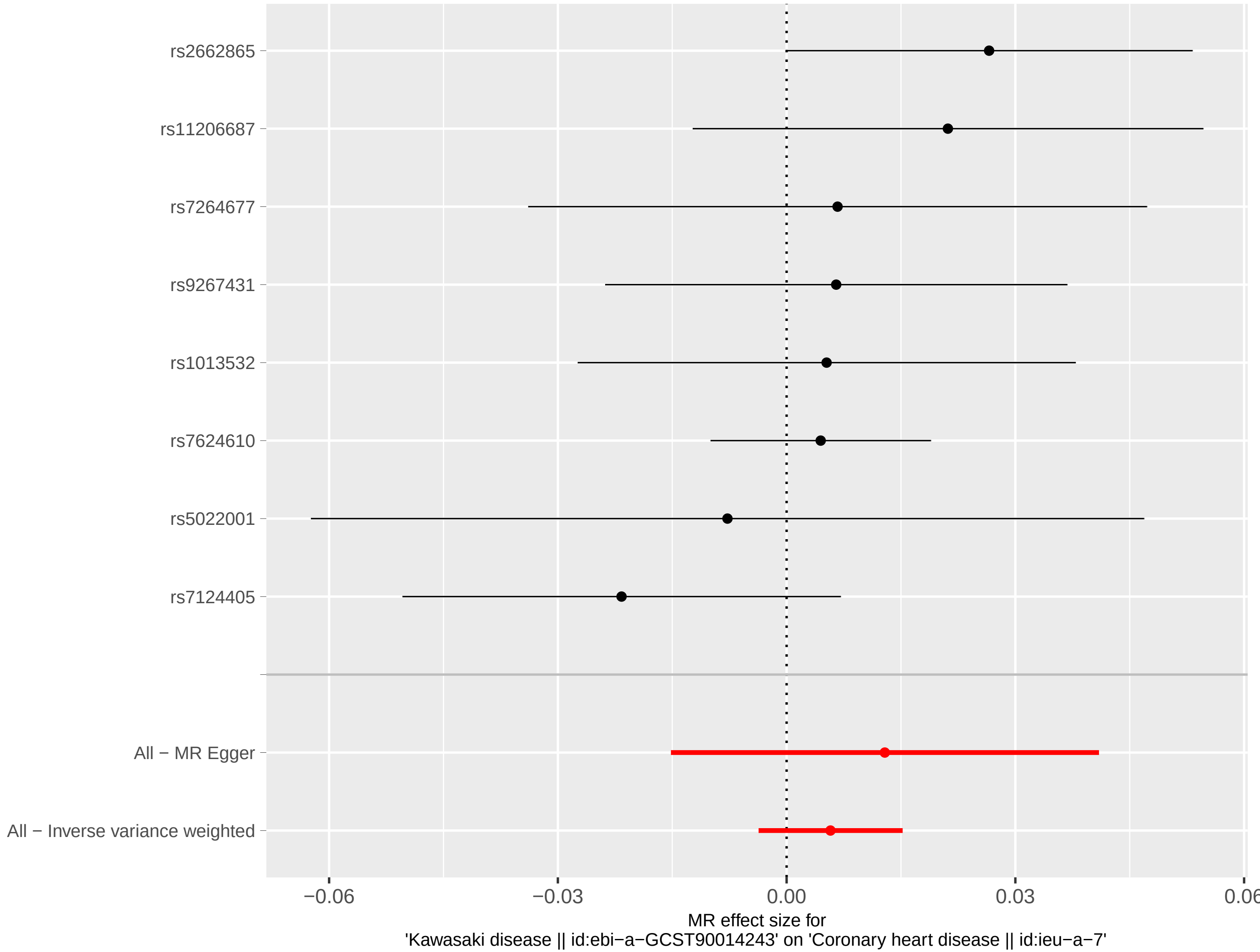
rs2662865
rs11206687
rs7264677
rs9267431
rs1013532
rs7624610
rs5022001
rs7124405
All − MR Egger
All − Inverse variance weighted
−0.06
−0.03
0.00
0.03
0.06
MR effect size for
'Kawasaki disease || id:ebi−a−GCST90014243' on 'Coronary heart disease || id:ieu−a−7'

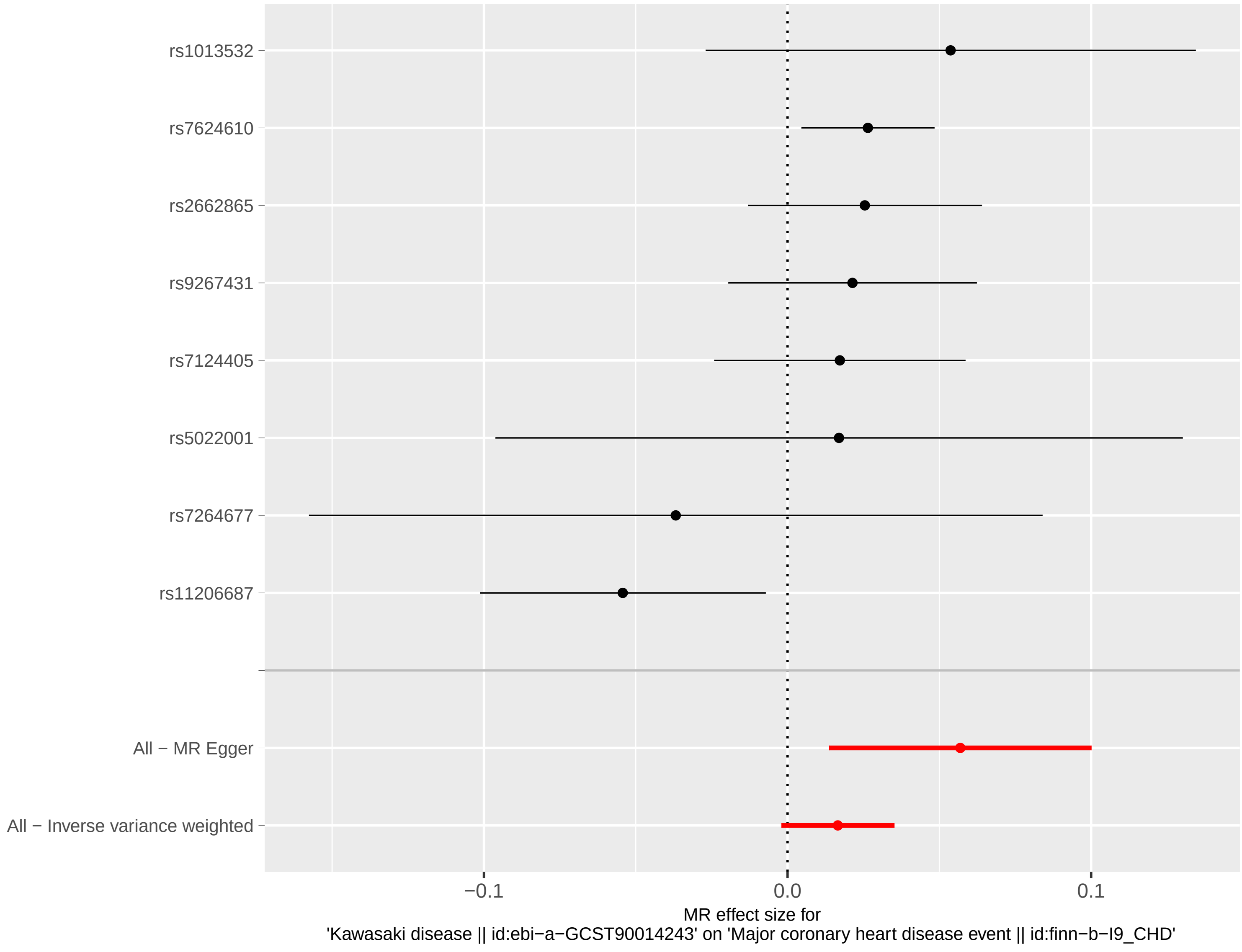

rs1013532
rs7624610
rs2662865
rs9267431
rs7124405
rs5022001
rs7264677
rs11206687
All − MR Egger
All − Inverse variance weighted
−0.1
0.0
0.1
MR effect size for
'Kawasaki disease || id:ebi−a−GCST90014243' on 'Major coronary heart disease event || id:finn−b−I9_CHD'

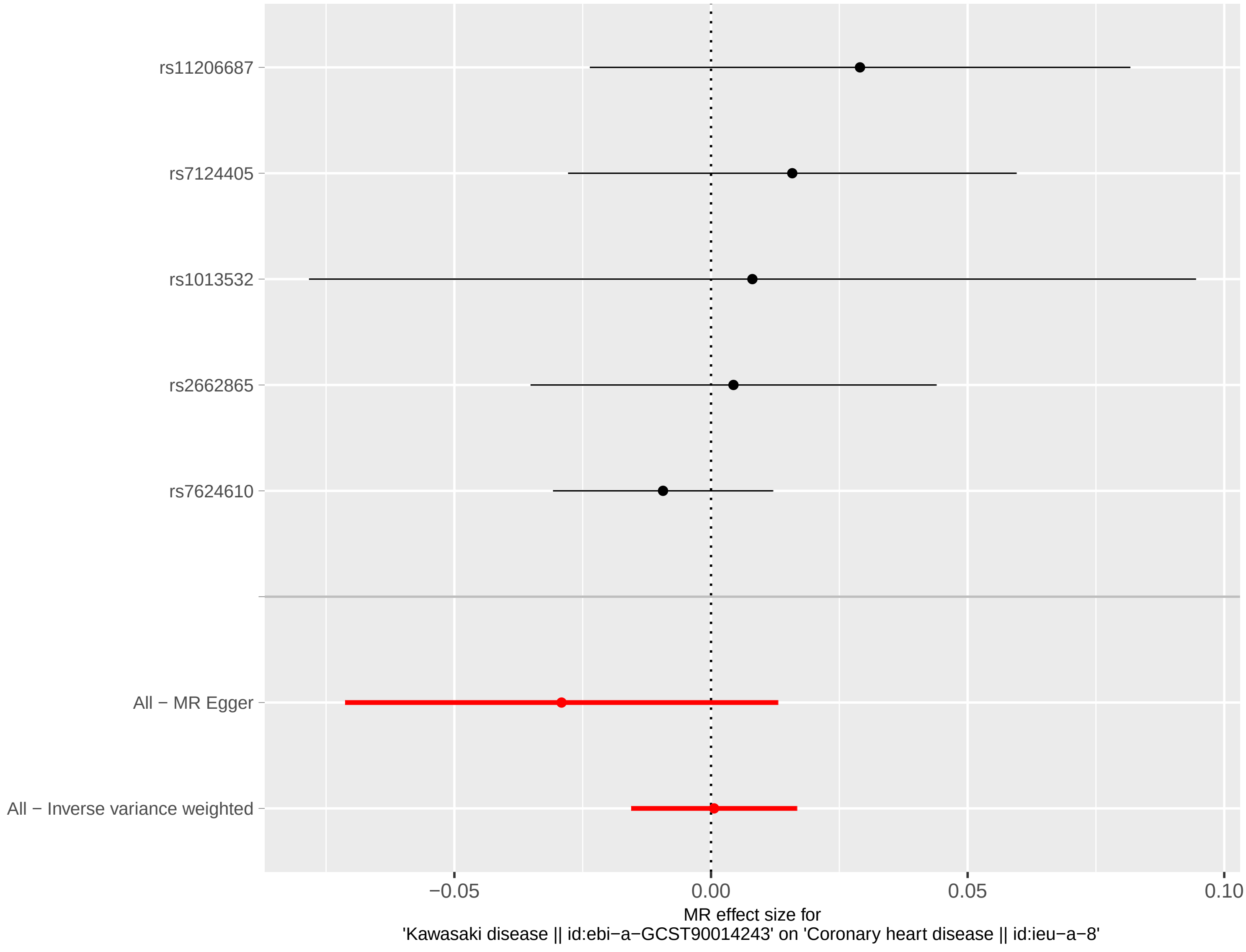
rs11206687
rs7124405
rs1013532
rs2662865
rs7624610
All − MR Egger
All − Inverse variance weighted
−0.05
0.00
0.05
0.10
MR effect size for
'Kawasaki disease || id:ebi−a−GCST90014243' on 'Coronary heart disease || id:ieu−a−8'

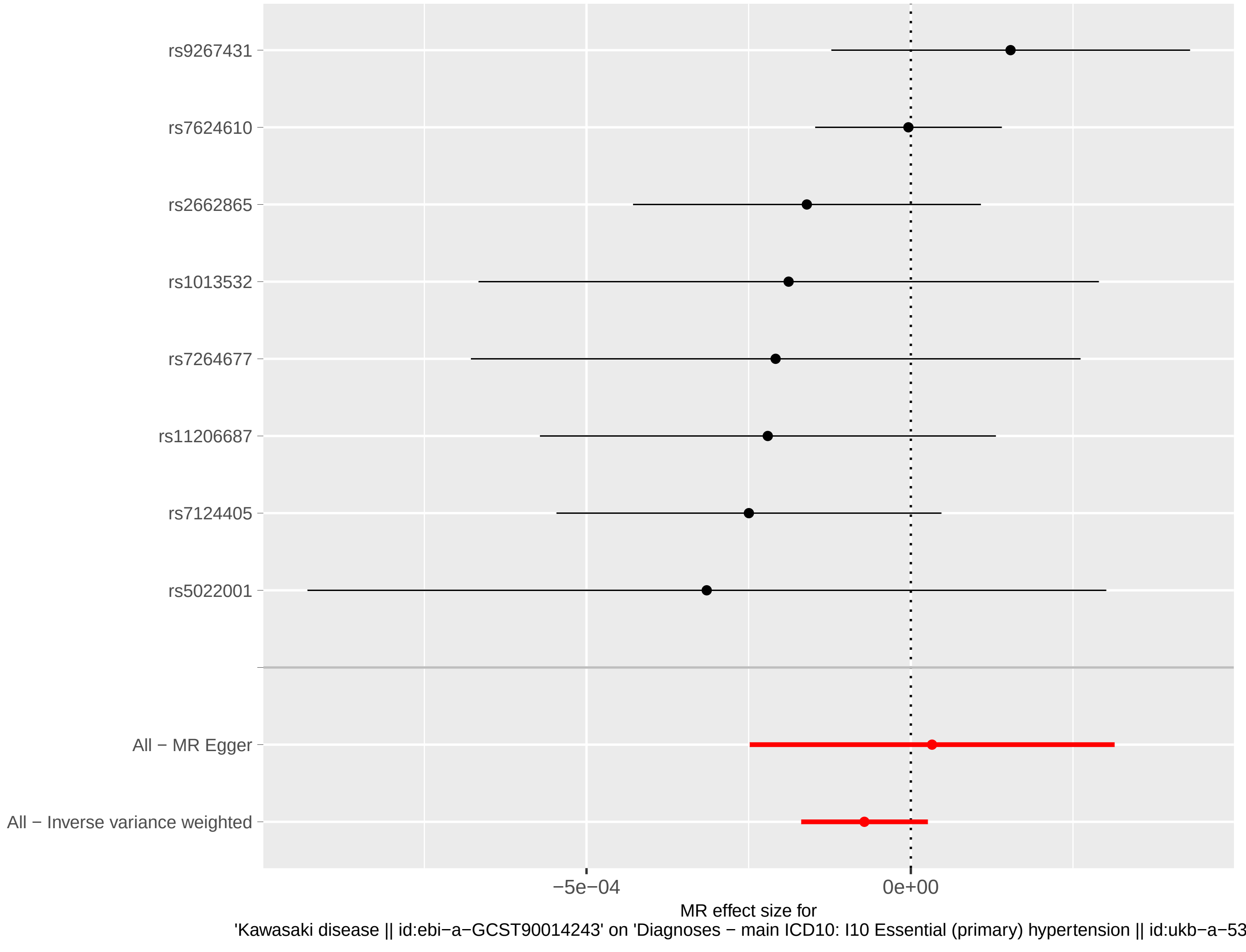

rs9267431
rs7624610
rs2662865
rs1013532
rs7264677
rs11206687
rs7124405
rs5022001
All − MR Egger
All − Inverse variance weighted
−5e−04
0e+00
MR effect size for
'Kawasaki disease || id:ebi−a−GCST90014243' on 'Diagnoses − main ICD10: I10 Essential (primary) hypertension || id:ukb−a−53

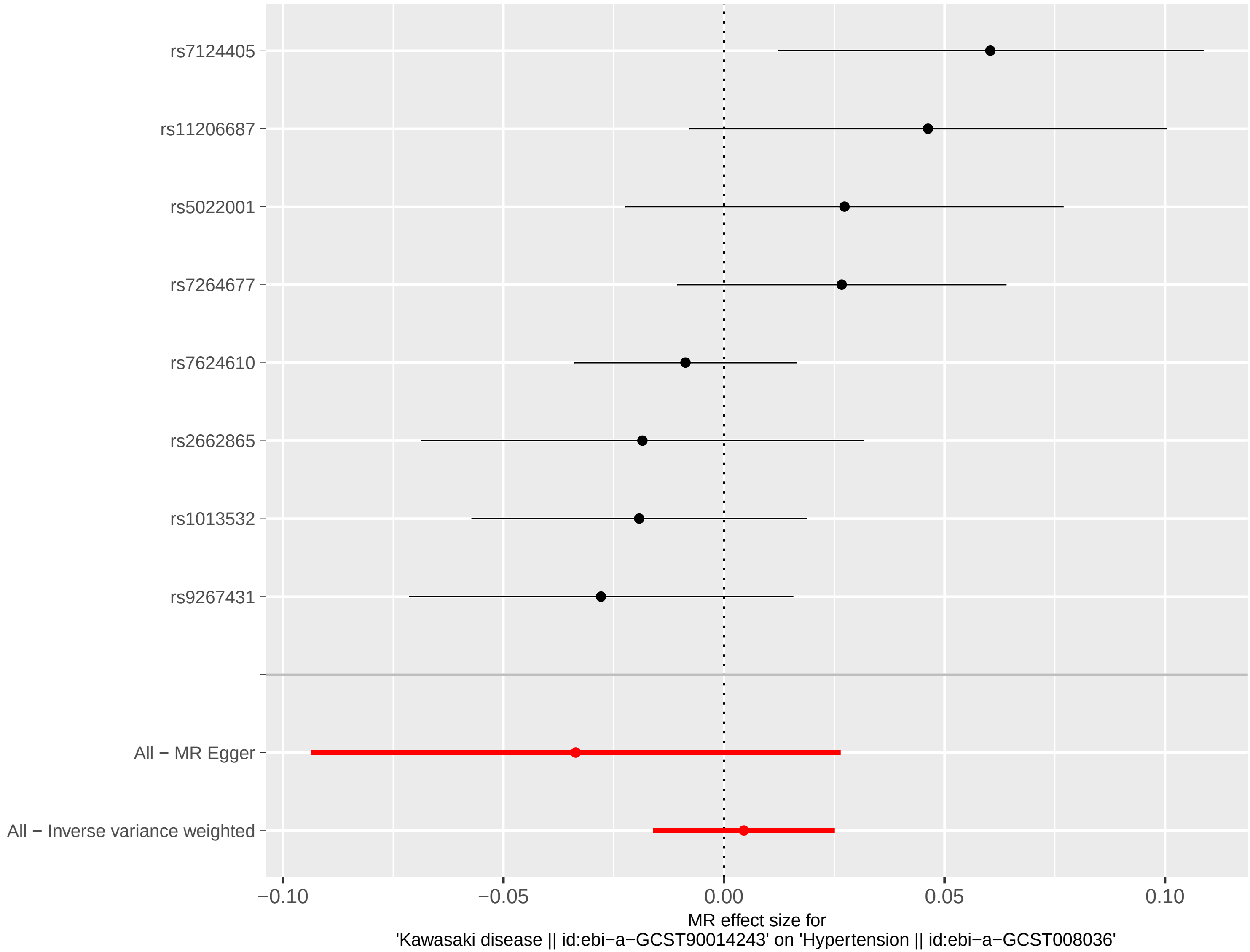
rs7124405
rs11206687
rs5022001
rs7264677
rs7624610
rs2662865
rs1013532
rs9267431
All − MR Egger
All − Inverse variance weighted
−0.10
−0.05
0.00
0.05
0.10
MR effect size for
'Kawasaki disease || id:ebi−a−GCST90014243' on 'Hypertension || id:ebi−a−GCST008036'

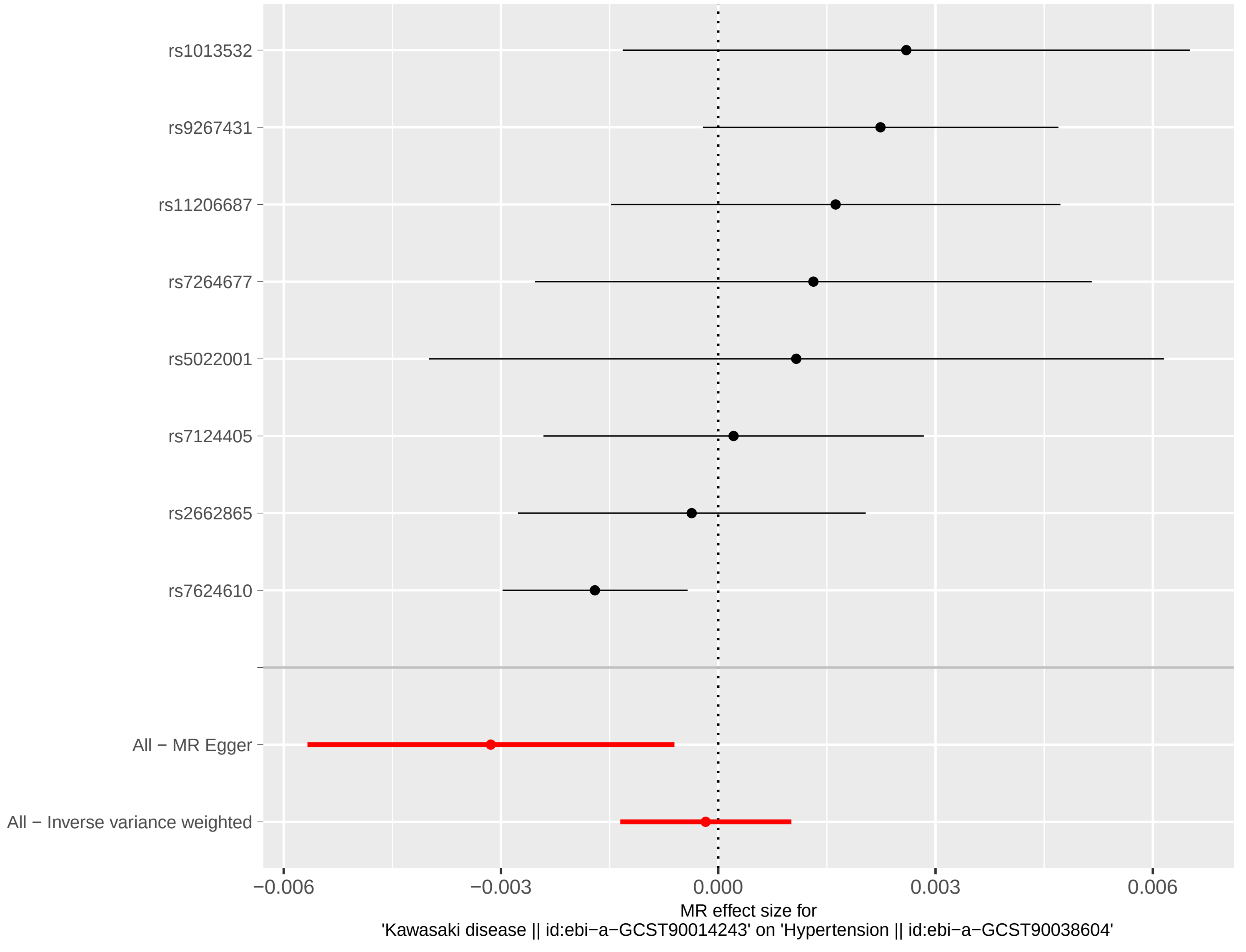

rs1013532
rs9267431
rs11206687
rs7264677
rs5022001
rs7124405
rs2662865
rs7624610
All − MR Egger
All − Inverse variance weighted
−0.006
−0.003
0.000
0.003
0.006
MR effect size for
'Kawasaki disease || id:ebi−a−GCST90014243' on 'Hypertension || id:ebi−a−GCST90038604'

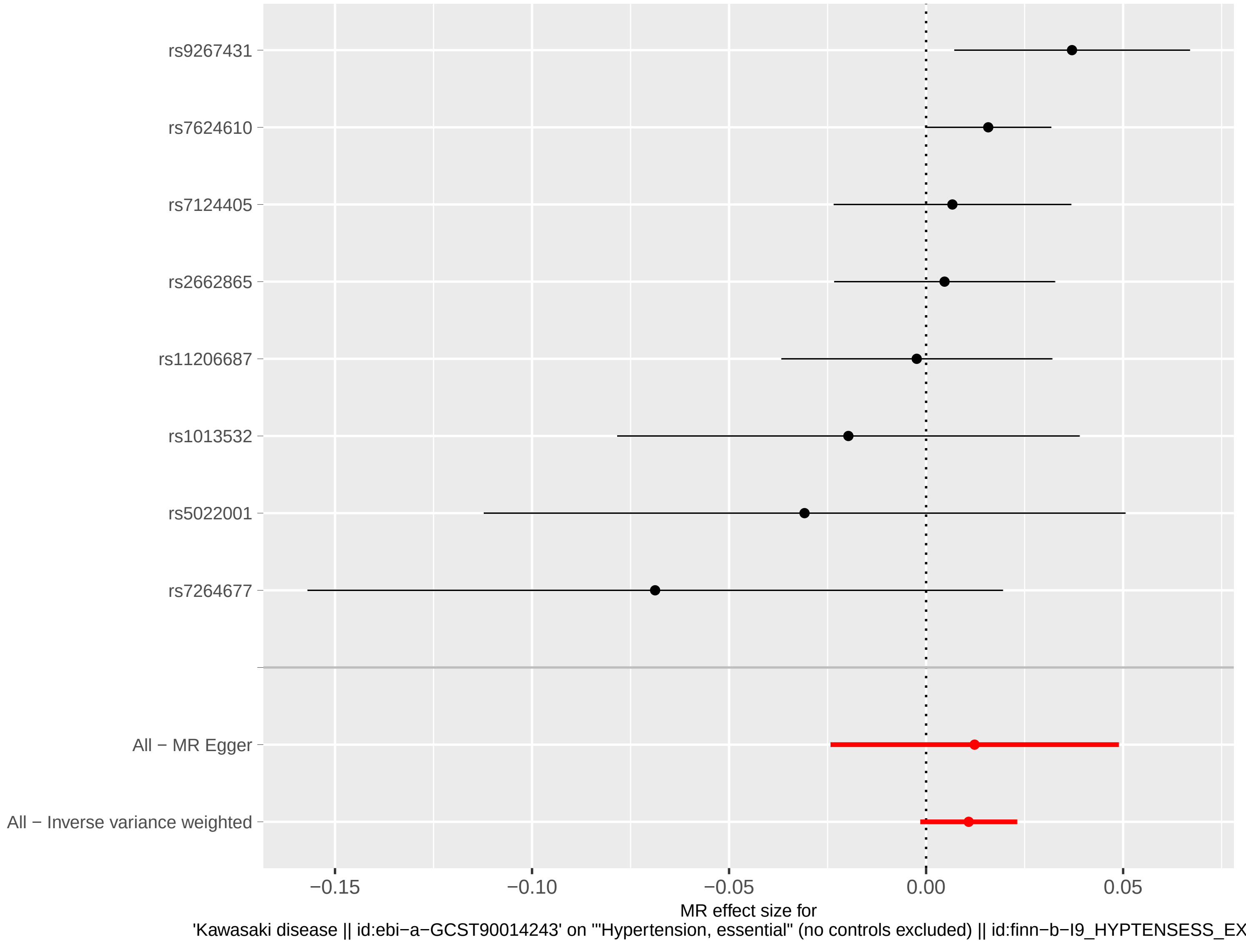
rs9267431
rs7624610
rs7124405
rs2662865
rs11206687
rs1013532
rs5022001
rs7264677
All − MR Egger
All − Inverse variance weighted
−0.15
−0.10
−0.05
0.00
0.05
MR effect size for
'Kawasaki disease || id:ebi−a−GCST90014243' on '"Hypertension, essential" (no controls excluded) || id:finn−b−I9_HYPTENSESS_EX

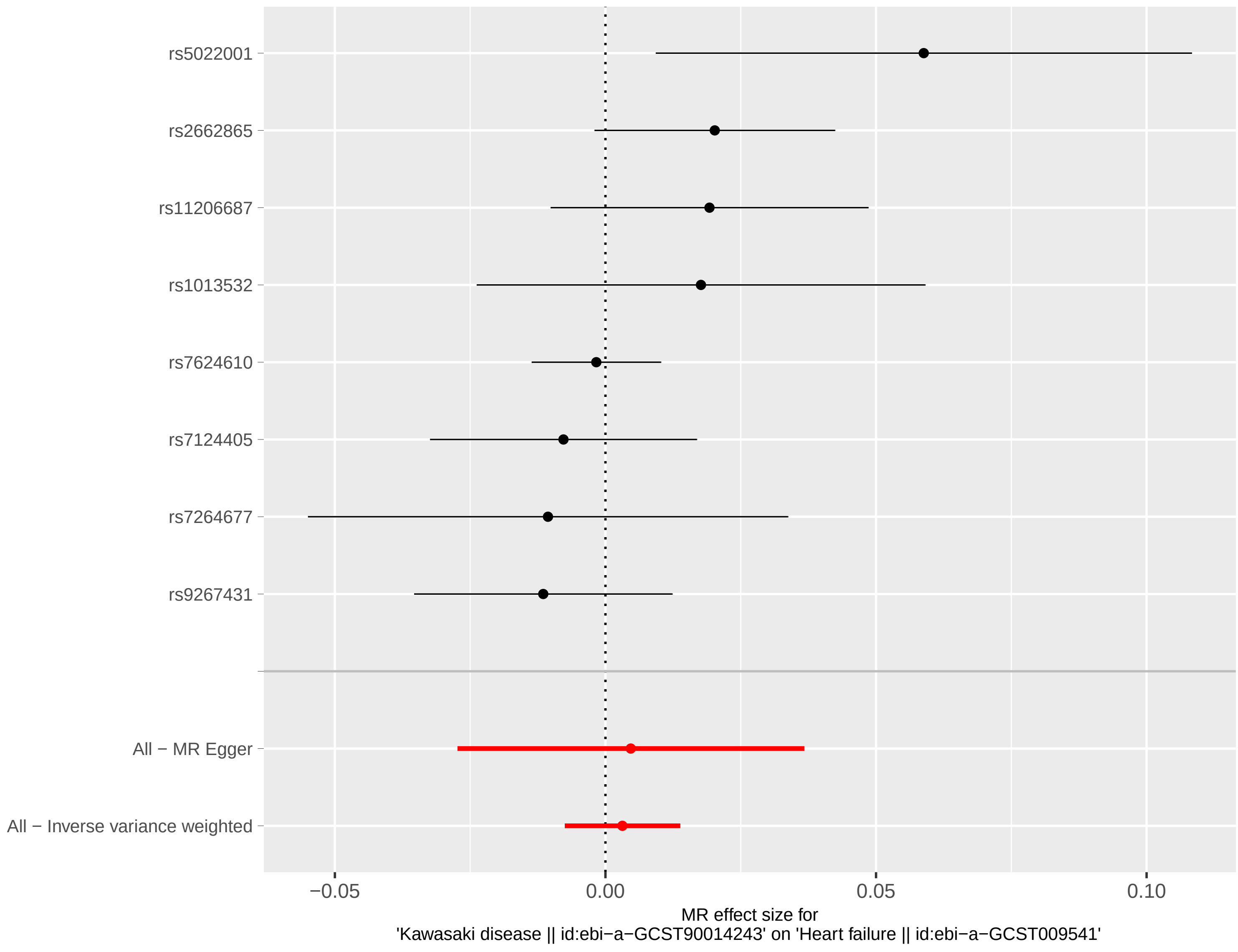

rs5022001
rs2662865
rs11206687
rs1013532
rs7624610
rs7124405
rs7264677
rs9267431
All − MR Egger
All − Inverse variance weighted
−0.05
0.00
0.05
0.10
MR effect size for
'Kawasaki disease || id:ebi−a−GCST90014243' on 'Heart failure || id:ebi−a−GCST009541'

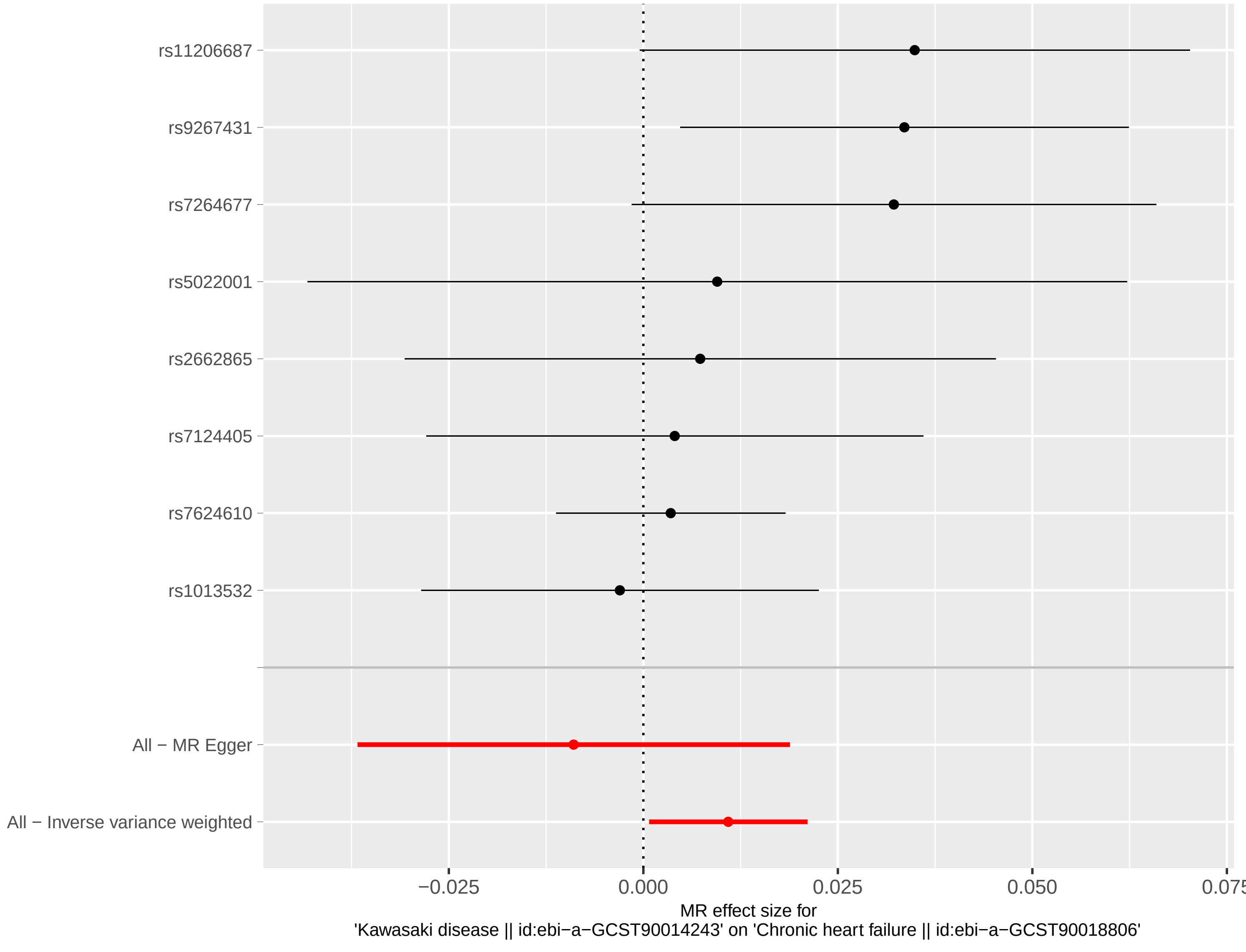

rs11206687
rs9267431
rs7264677
rs5022001
rs2662865
rs7124405
rs7624610
rs1013532
All − MR Egger
All − Inverse variance weighted
−0.025
0.000
0.025
0.050
MR effect size for
'Kawasaki disease || id:ebi−a−GCST90014243' on 'Chronic heart failure || id:ebi−a−GCST90018806'

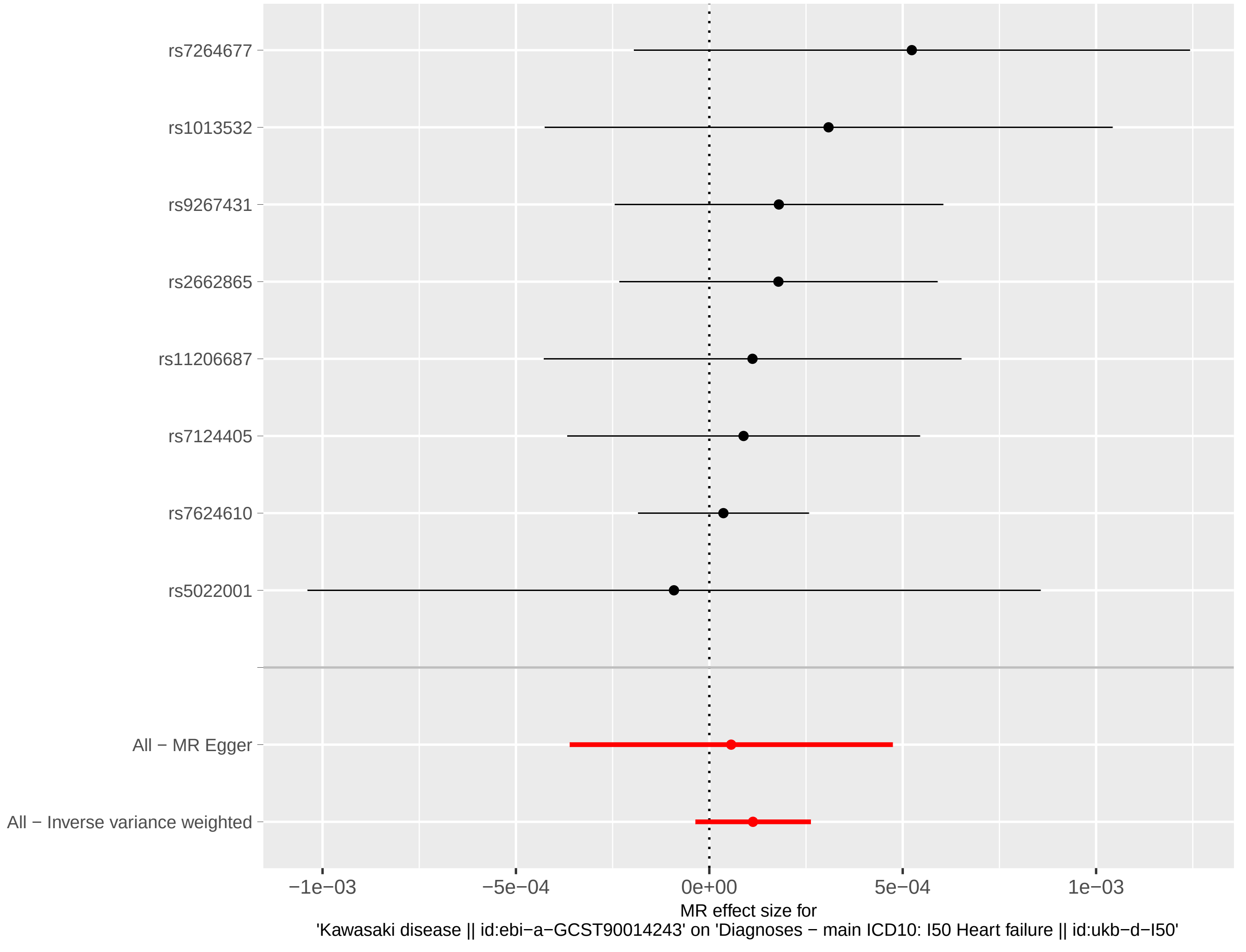
rs7264677
rs1013532
rs9267431
rs2662865
rs11206687
rs7124405
rs7624610
rs5022001
All − MR Egger
All − Inverse variance weighted
−1e−03
−5e−04
0e+00
5e−04
1e−03
MR effect size for
'Kawasaki disease || id:ebi−a−GCST90014243' on 'Diagnoses − main ICD10: I50 Heart failure || id:ukb−d−I50'

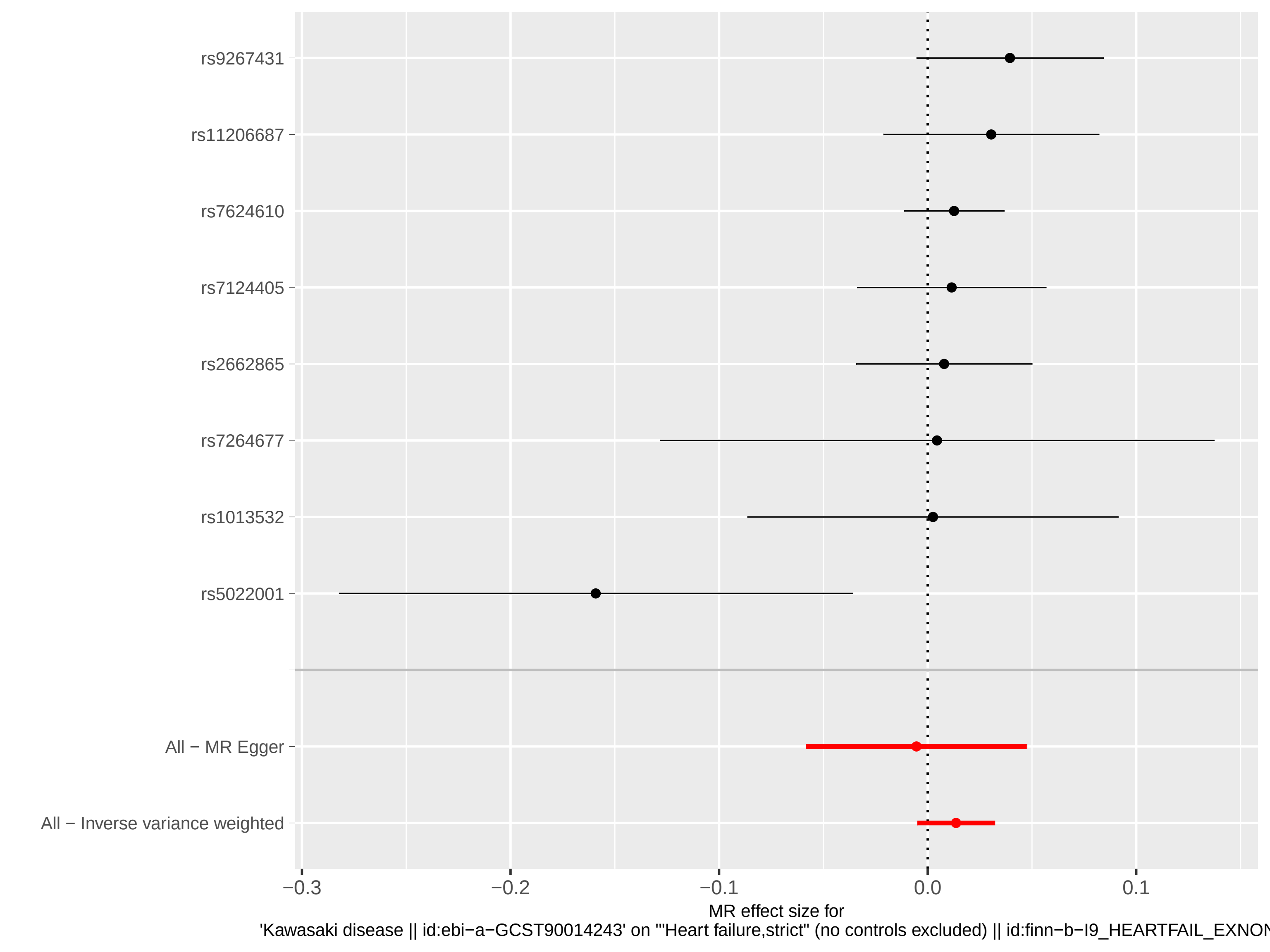

rs9267431
rs11206687
rs7624610
rs7124405
rs2662865
rs7264677
rs1013532
rs5022001
All − MR Egger
All − Inverse variance weighted
−0.3
−0.2
−0.1
0.0
0.1
MR effect size for
'Kawasaki disease || id:ebi−a−GCST90014243' on '"Heart failure,strict" (no controls excluded) || id:finn−b−I9_HEARTFAIL_EXNON

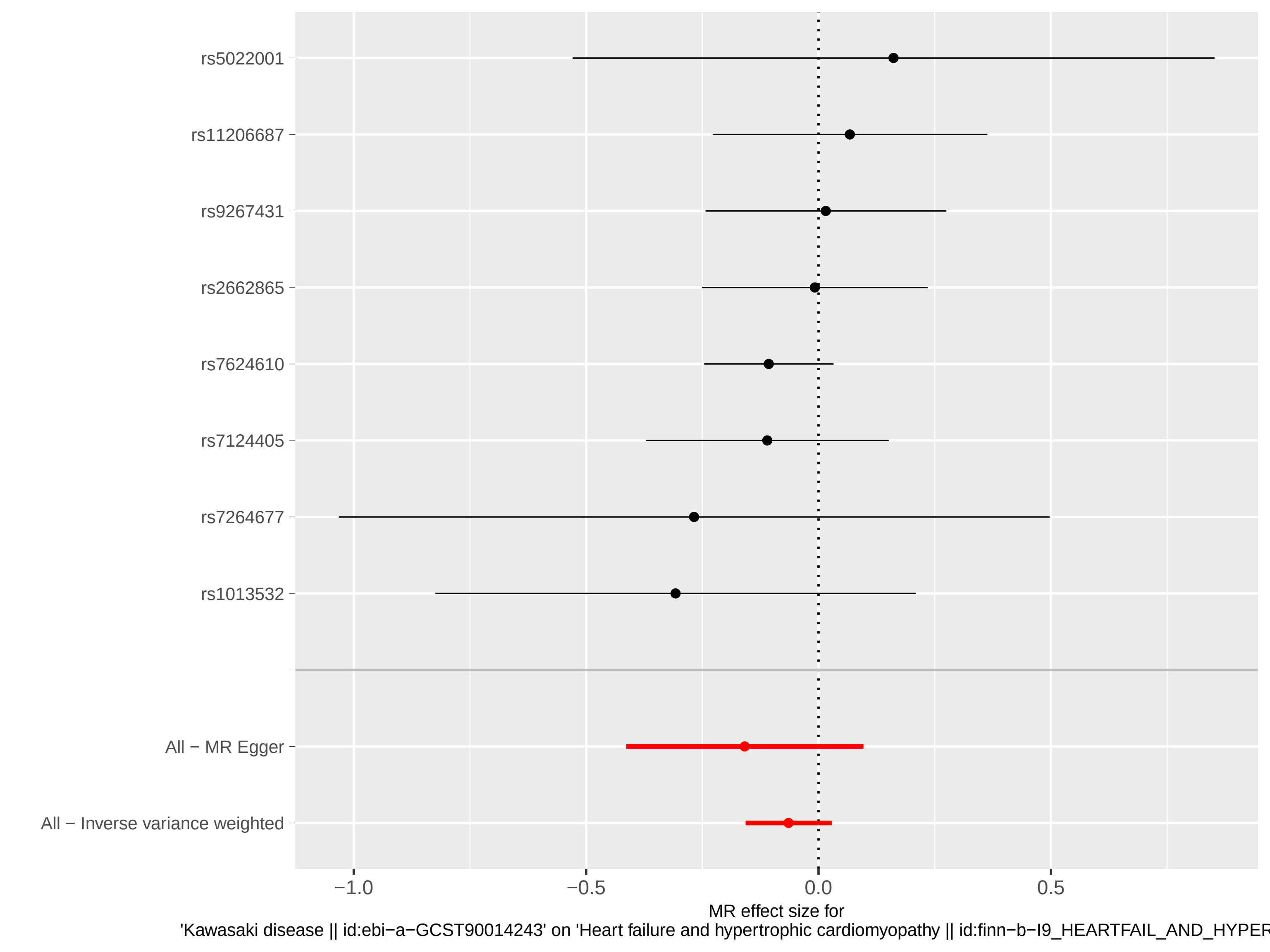

rs5022001
rs11206687
rs9267431
rs2662865
rs7624610
rs7124405
rs7264677
rs1013532
All − MR Egger
All − Inverse variance weighted
−1.0
−0.5
0.0
0.5
MR effect size for
'Kawasaki disease || id:ebi−a−GCST90014243' on 'Heart failure and hypertrophic cardiomyopathy || id:finn−b−I9_HEARTFAIL_AND_HYPER

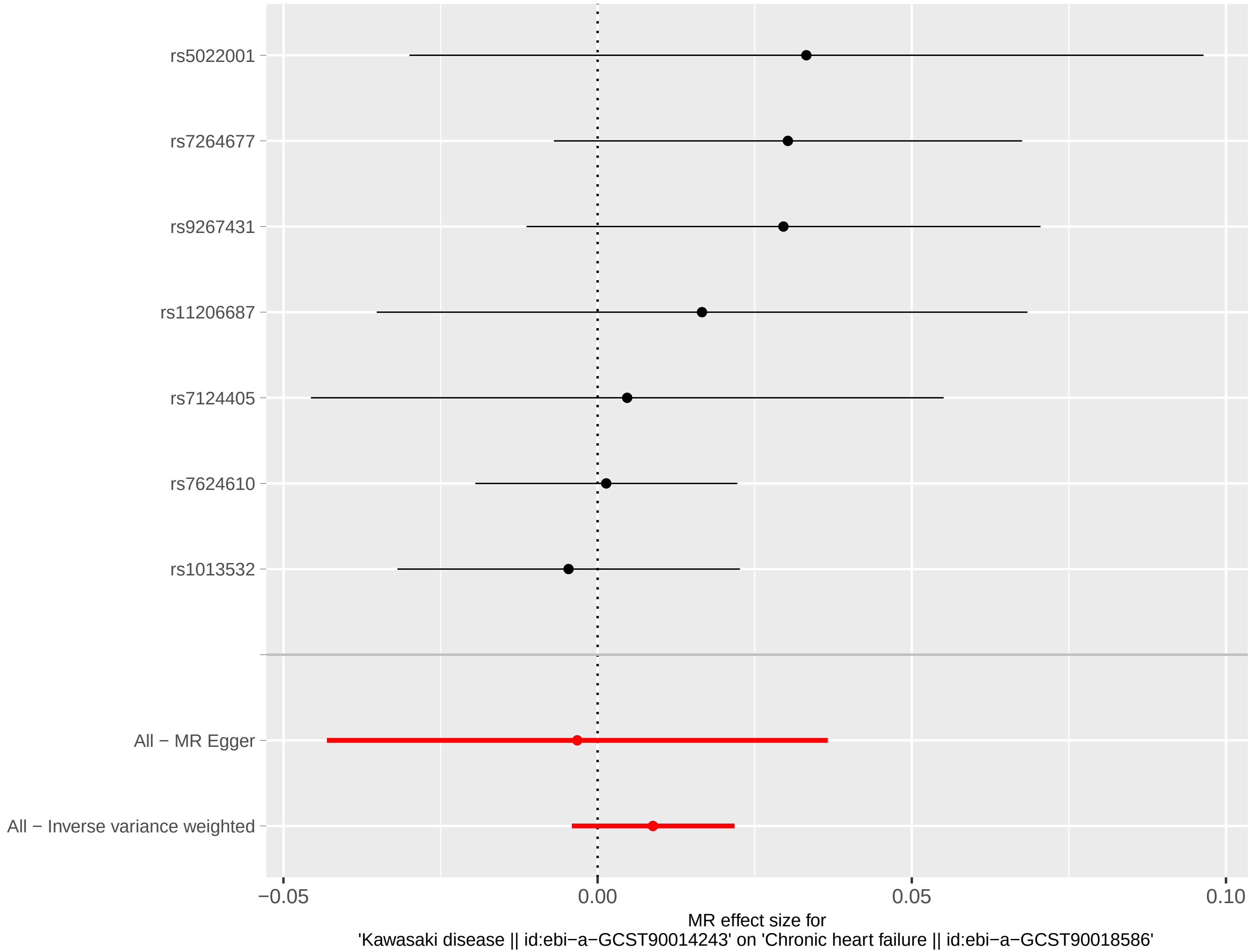
rs5022001
rs7264677
rs9267431
rs11206687
rs7124405
rs7624610
rs1013532
All − MR Egger
All − Inverse variance weighted
−0.05
0.00
0.05
0.10
MR effect size for
'Kawasaki disease || id:ebi−a−GCST90014243' on 'Chronic heart failure || id:ebi−a−GCST90018586'

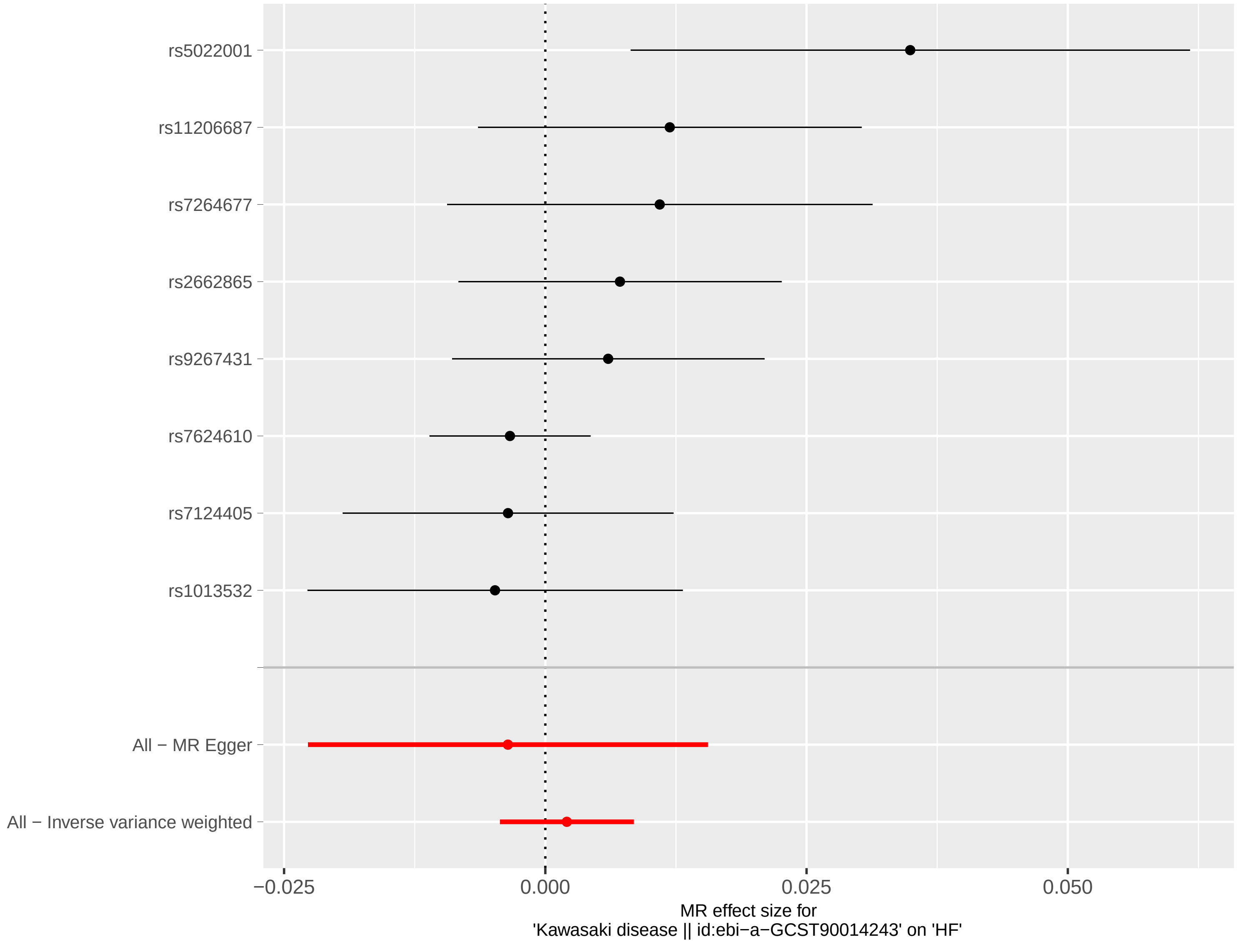

rs5022001
rs11206687
rs7264677
rs2662865
rs9267431
rs7624610
rs7124405
rs1013532
All − MR Egger
All − Inverse variance weighted
−0.025
0.000
0.025
0.050
MR effect size for
'Kawasaki disease || id:ebi−a−GCST90014243' on 'HF'

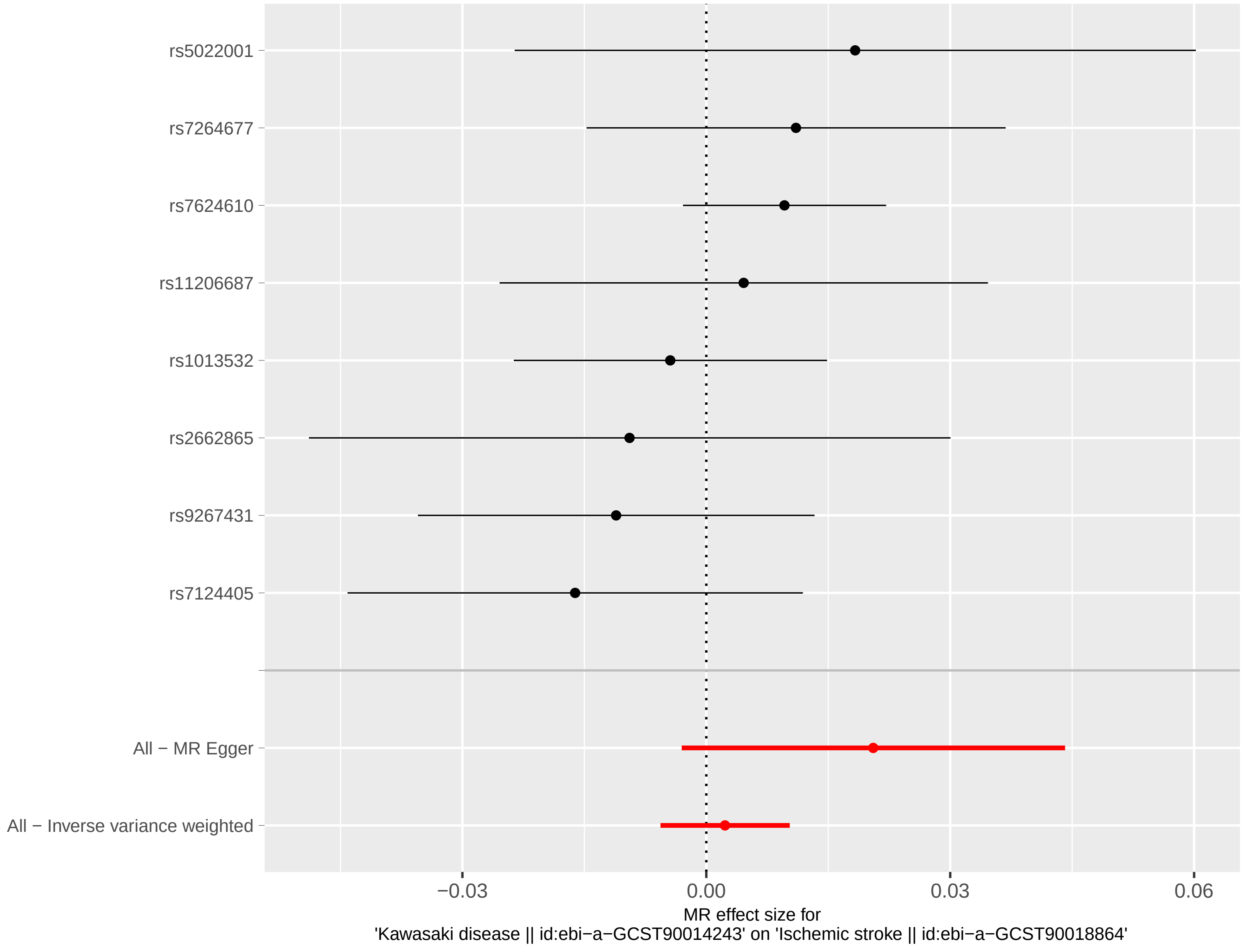
rs5022001
rs7264677
rs7624610
rs11206687
rs1013532
rs2662865
rs9267431
rs7124405
All − MR Egger
All − Inverse variance weighted
−0.03
0.00
0.03
0.06
MR effect size for
'Kawasaki disease || id:ebi−a−GCST90014243' on 'Ischemic stroke || id:ebi−a−GCST90018864'

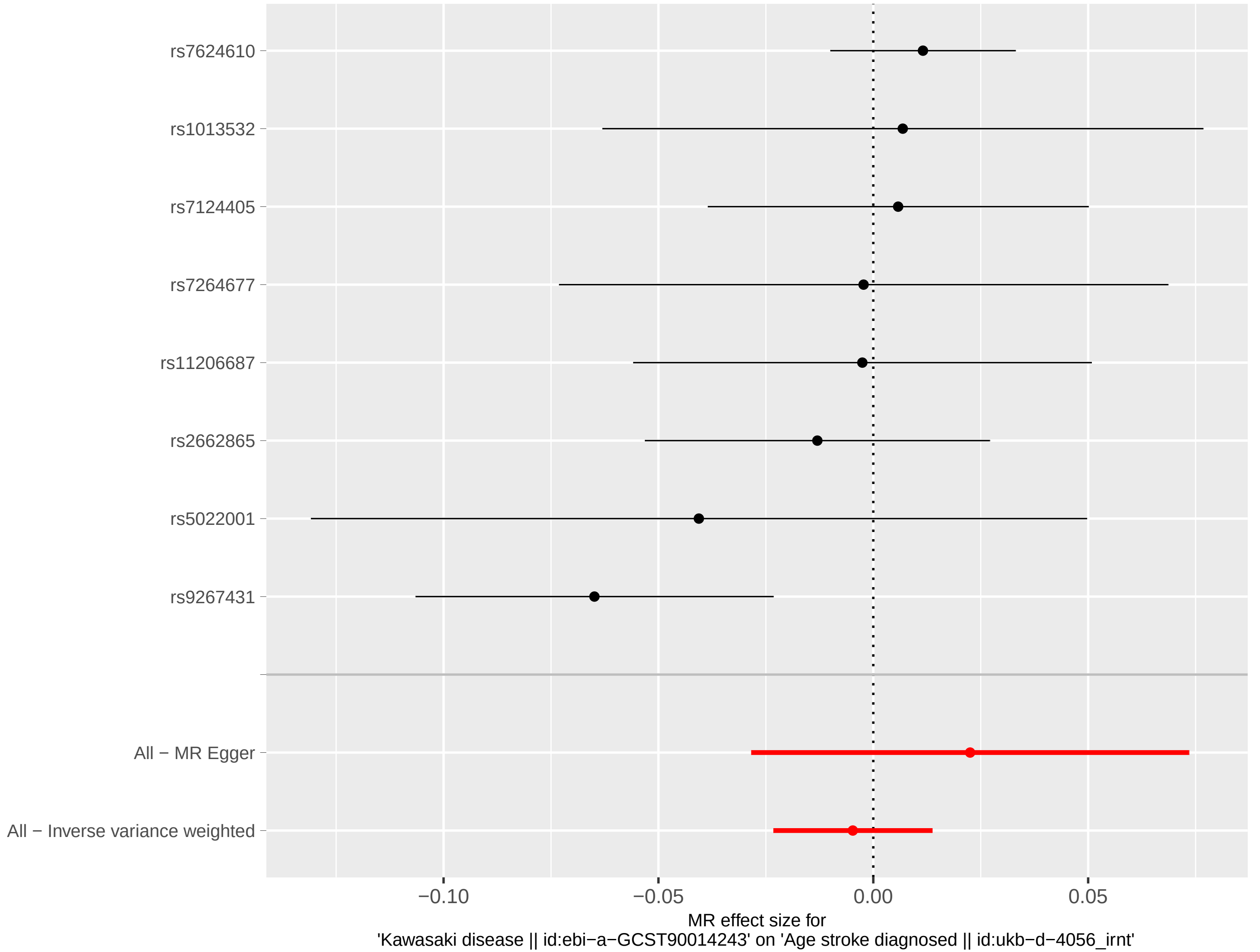
rs7624610
rs1013532
rs7124405
rs7264677
rs11206687
rs2662865
rs5022001
rs9267431
All − MR Egger
All − Inverse variance weighted
−0.10
−0.05
0.00
0.05
MR effect size for
'Kawasaki disease || id:ebi−a−GCST90014243' on 'Age stroke diagnosed || id:ukb−d−4056_irnt'
