# Supplementary material for: Genetic liability to childhood Kawasaki disease and adult cardiovascular outcomes
Source: Front Cardiovasc Med. 2026 Jul 23;13:1848320. doi: 10.3389/fcvm.2026.1848320 (PMC13441699; doi:10.3389/fcvm.2026.1848320)
Supplement: Supplementary file 4 [file Datasheet4.pdf]

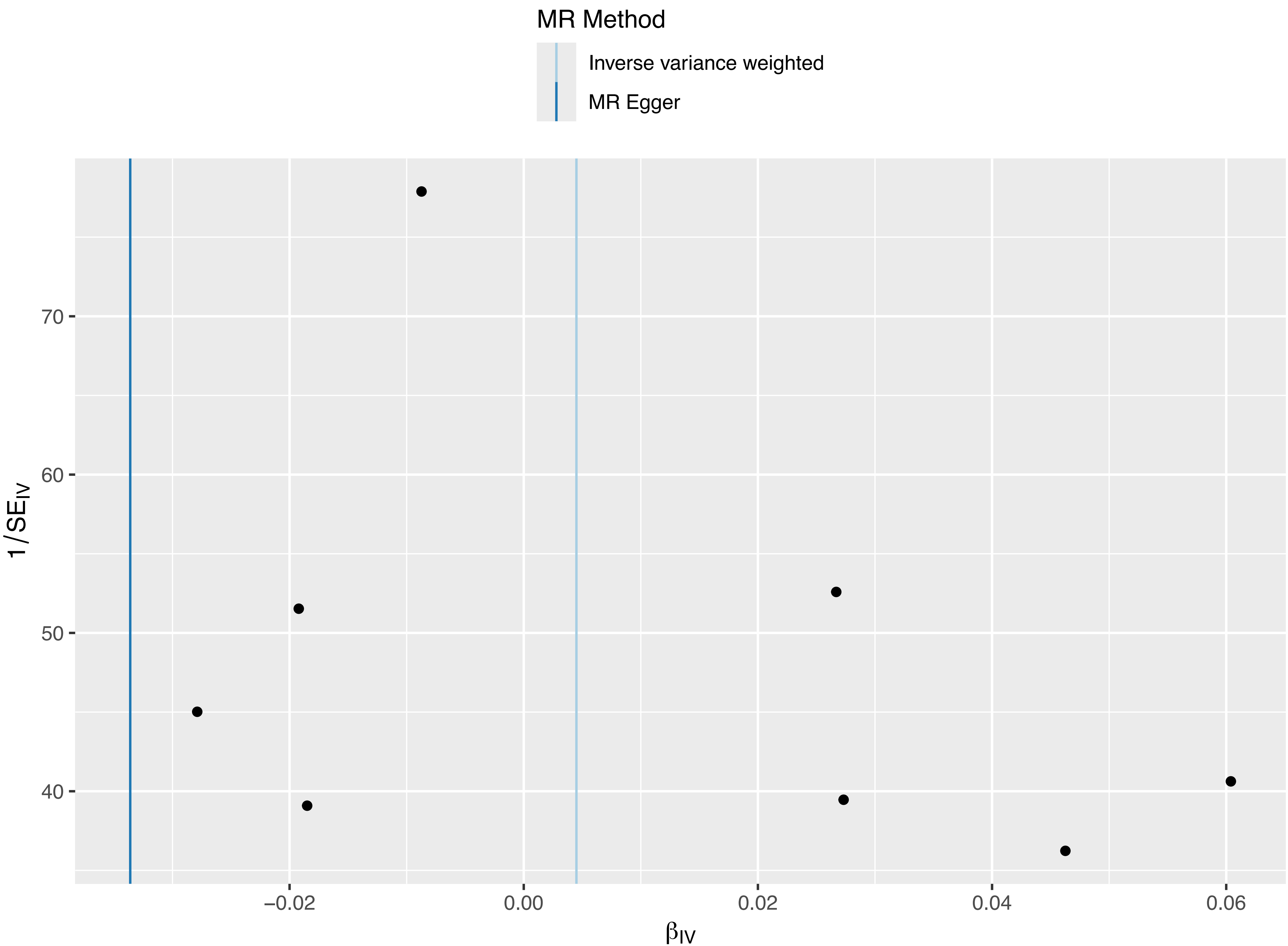

MR Method
Inverse variance weighted
MR Egger
70
60
50
40
1/SE_IV
−0.02
0.00
0.02
0.04
0.06
β_IV

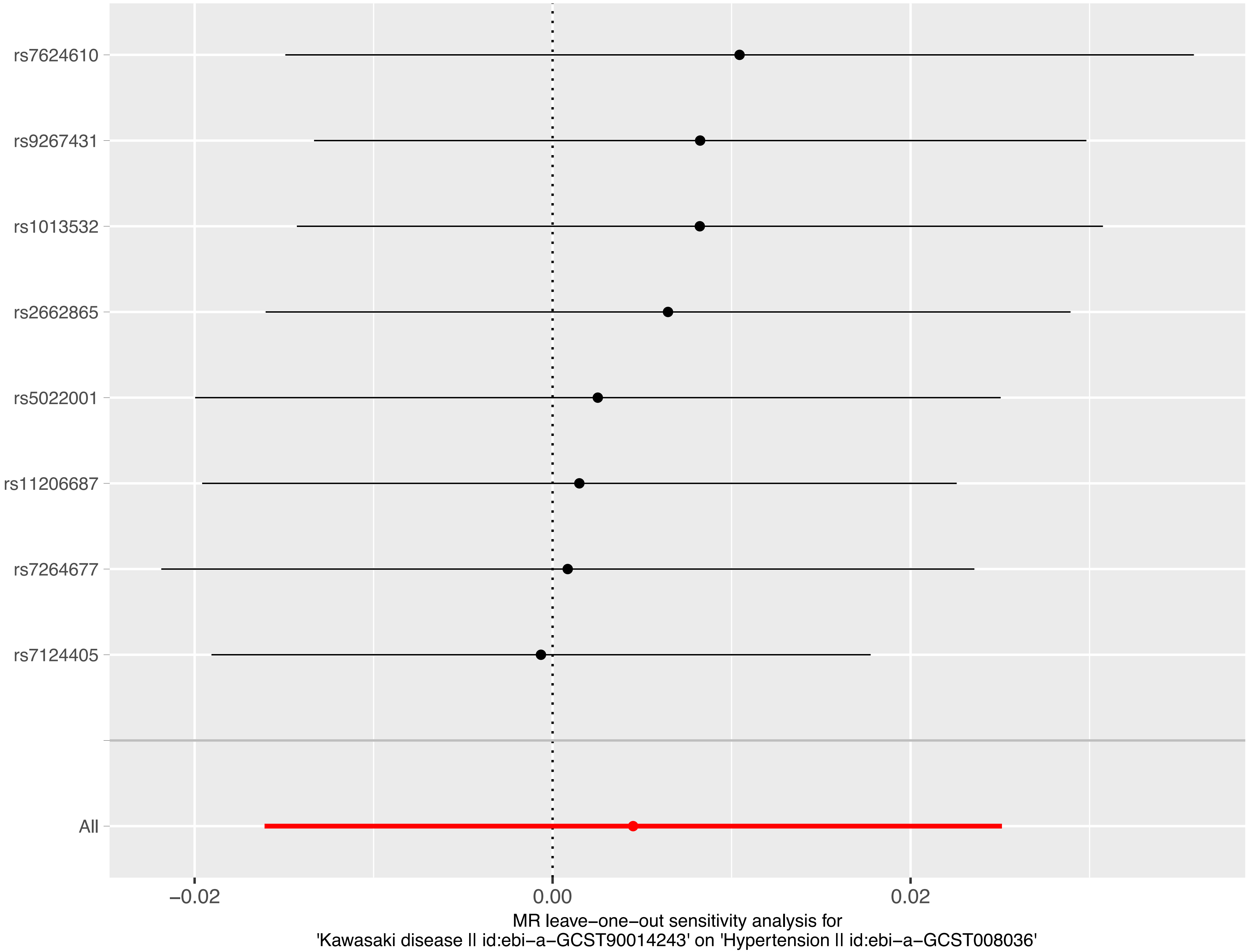

rs7624610
rs9267431
rs1013532
rs2662865
rs5022001
rs11206687
rs7264677
rs7124405
All
−0.02
0.00
0.02
MR leave−one−out sensitivity analysis for
'Kawasaki disease || id:ebi−a−GCST90014243' on 'Hypertension || id:ebi−a−GCST008036'

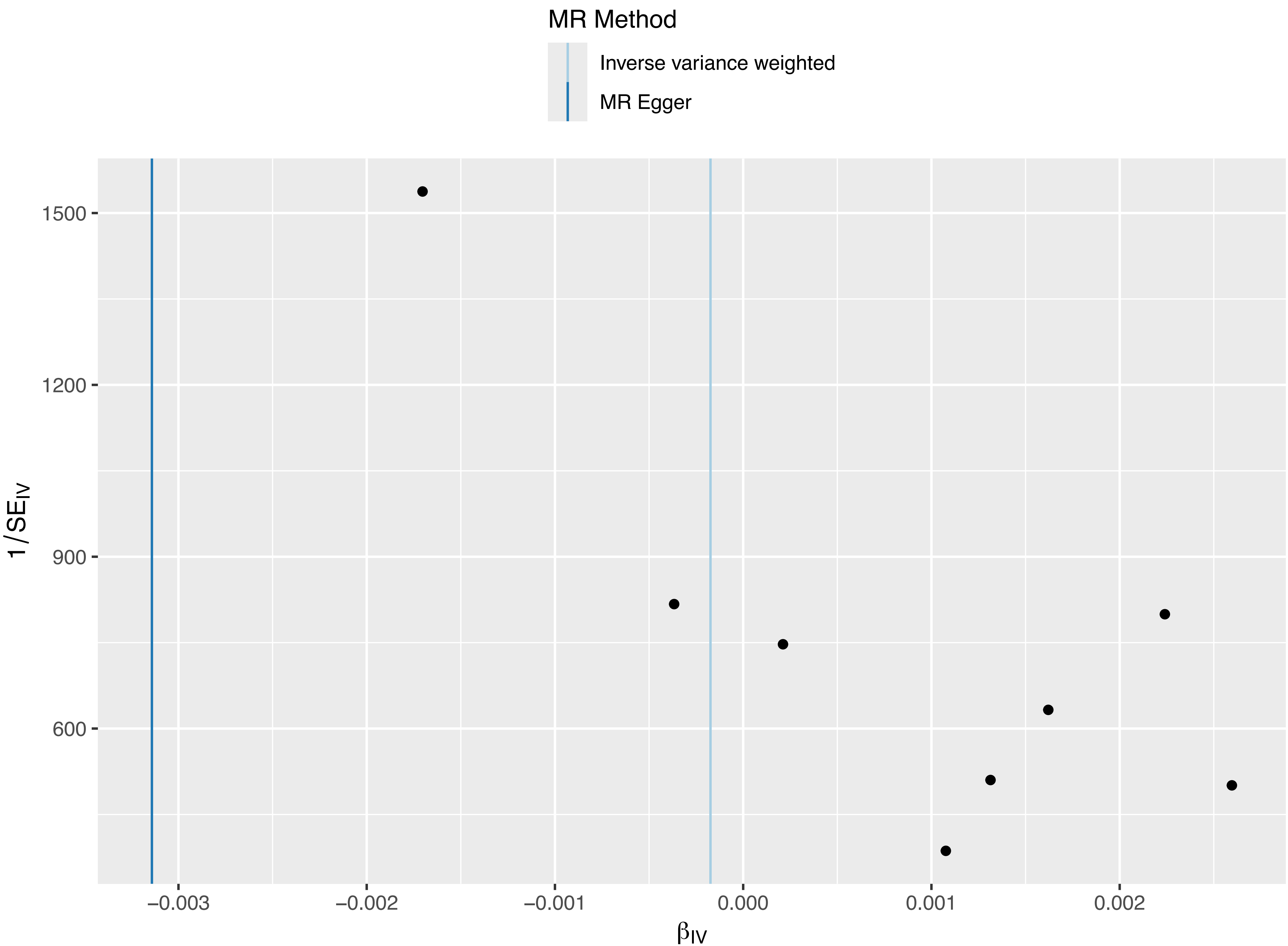

MR Method
Inverse variance weighted
MR Egger
1500
1200
900
600
−0.003
−0.002
−0.001
0.000
0.001
0.002
$1/SE_{IV}$
β_{IV}

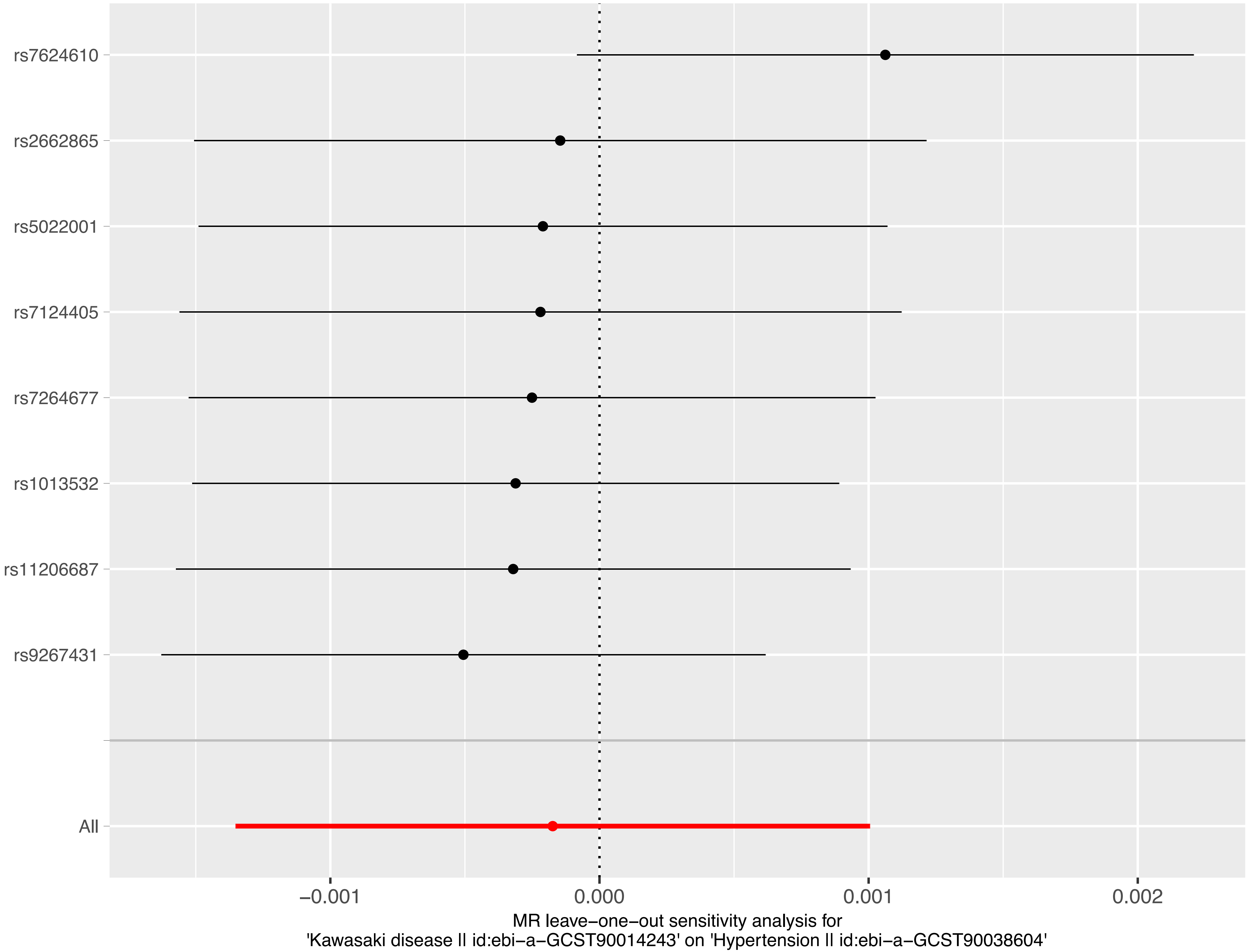
rs7624610
rs2662865
rs5022001
rs7124405
rs7264677
rs1013532
rs11206687
rs9267431
All
−0.001
0.000
0.001
0.002
MR leave−one−out sensitivity analysis for
'Kawasaki disease || id:ebi−a−GCST90014243' on 'Hypertension || id:ebi−a−GCST90038604'

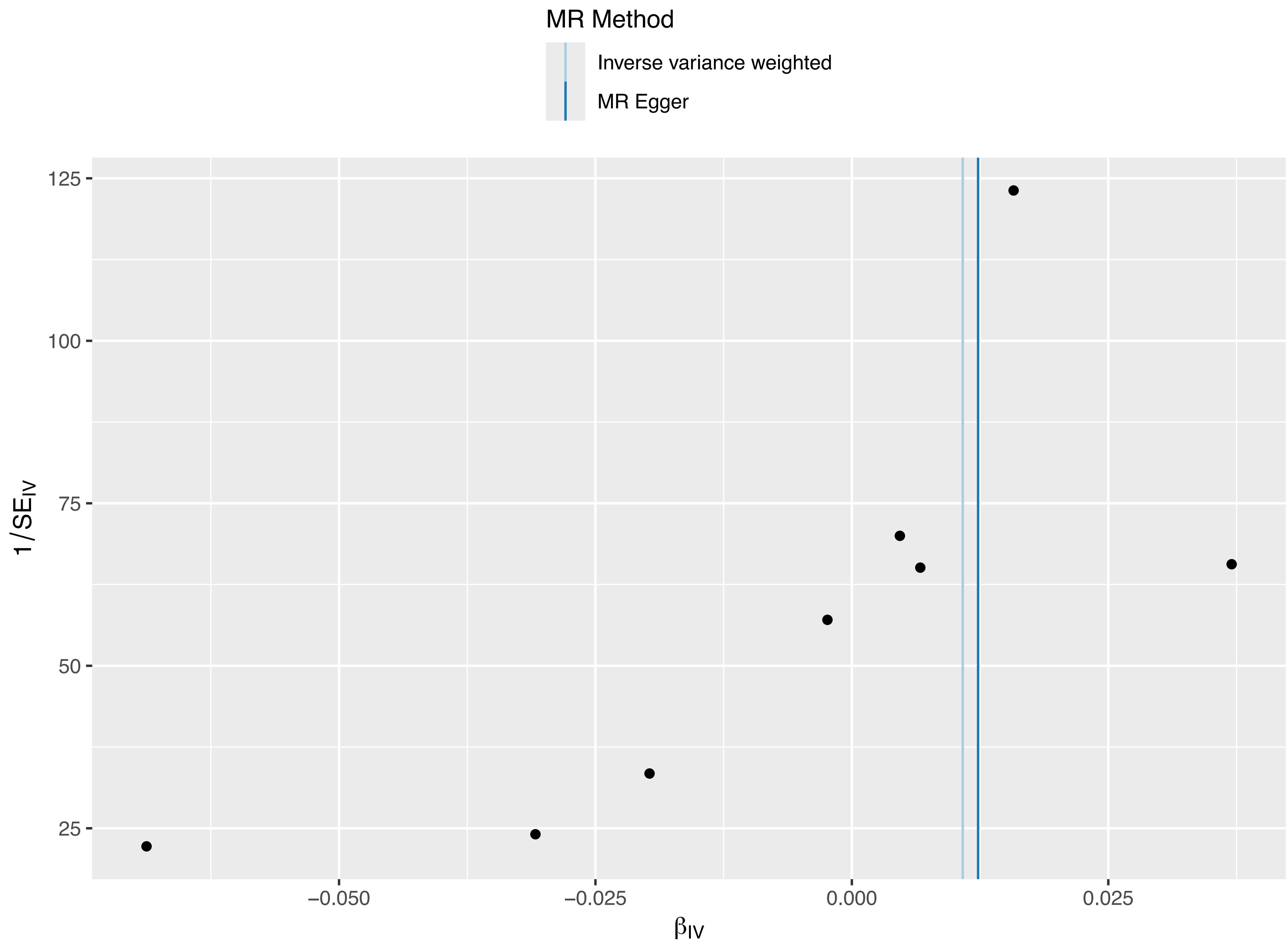
MR Method
Inverse variance weighted
MR Egger
125
100
75
50
25
1/SE_IV
−0.050
−0.025
0.000
0.025
β_IV

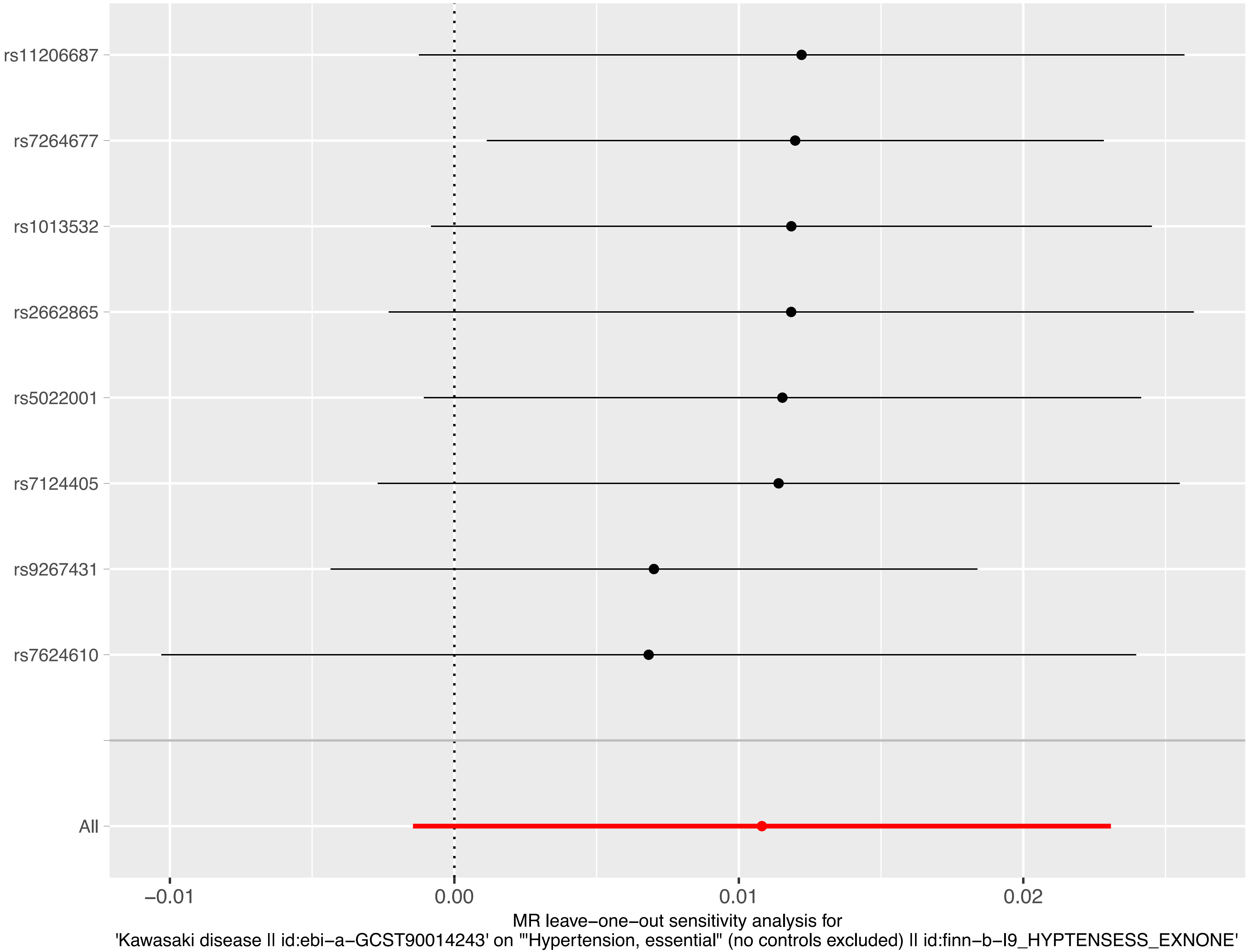
rs11206687
rs7264677
rs1013532
rs2662865
rs5022001
rs7124405
rs9267431
rs7624610
All
−0.01
0.00
0.01
0.02
MR leave−one−out sensitivity analysis for
'Kawasaki disease || id:ebi−a−GCST90014243' on '"Hypertension, essential" (no controls excluded) || id:finn−b−I9_HYPTENSESS_EXNONE'

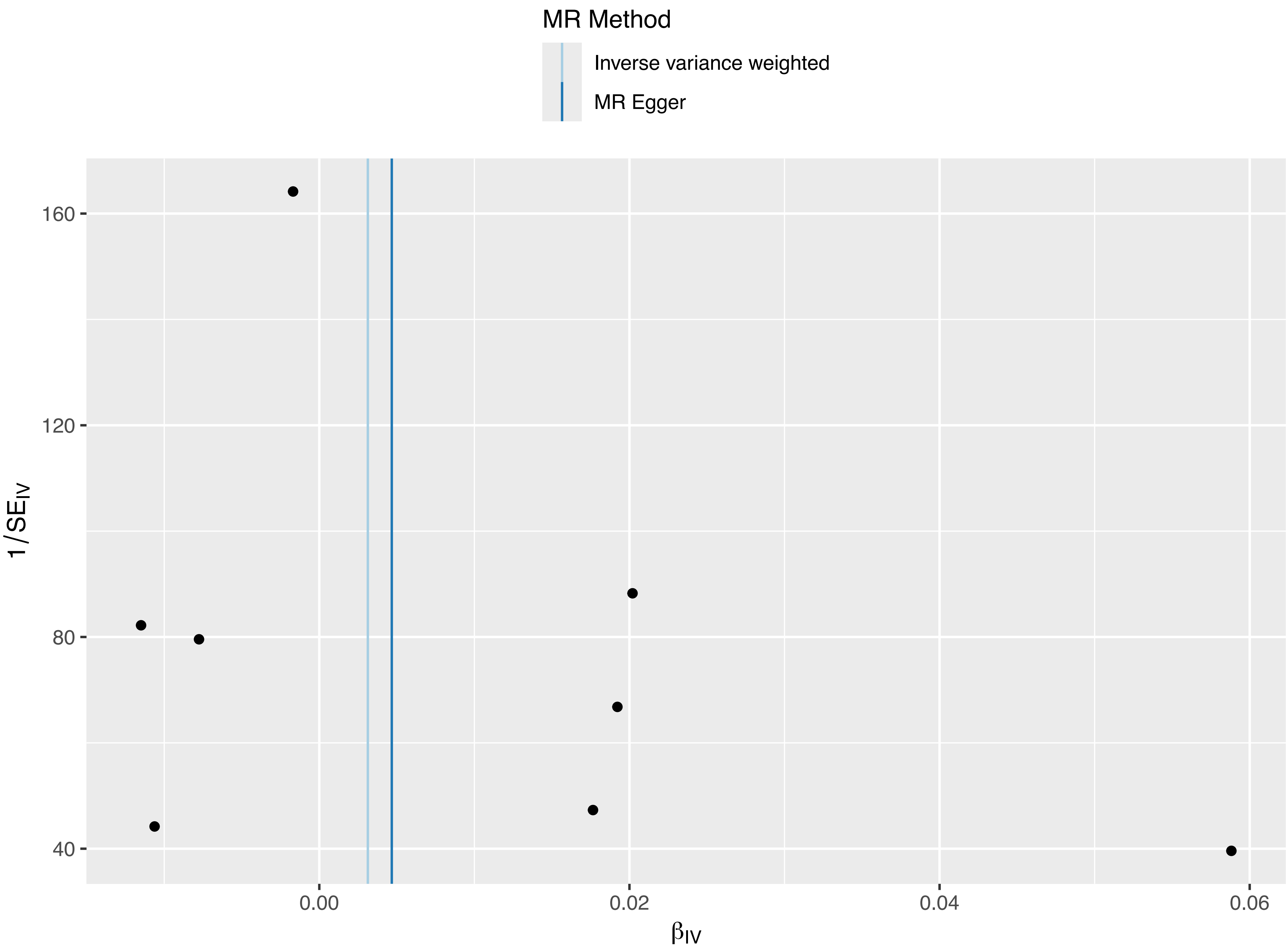
MR Method
Inverse variance weighted
MR Egger
160
120
80
40
$1/SE_{IV}$
0.00
0.02
0.04
0.06
β_{IV}

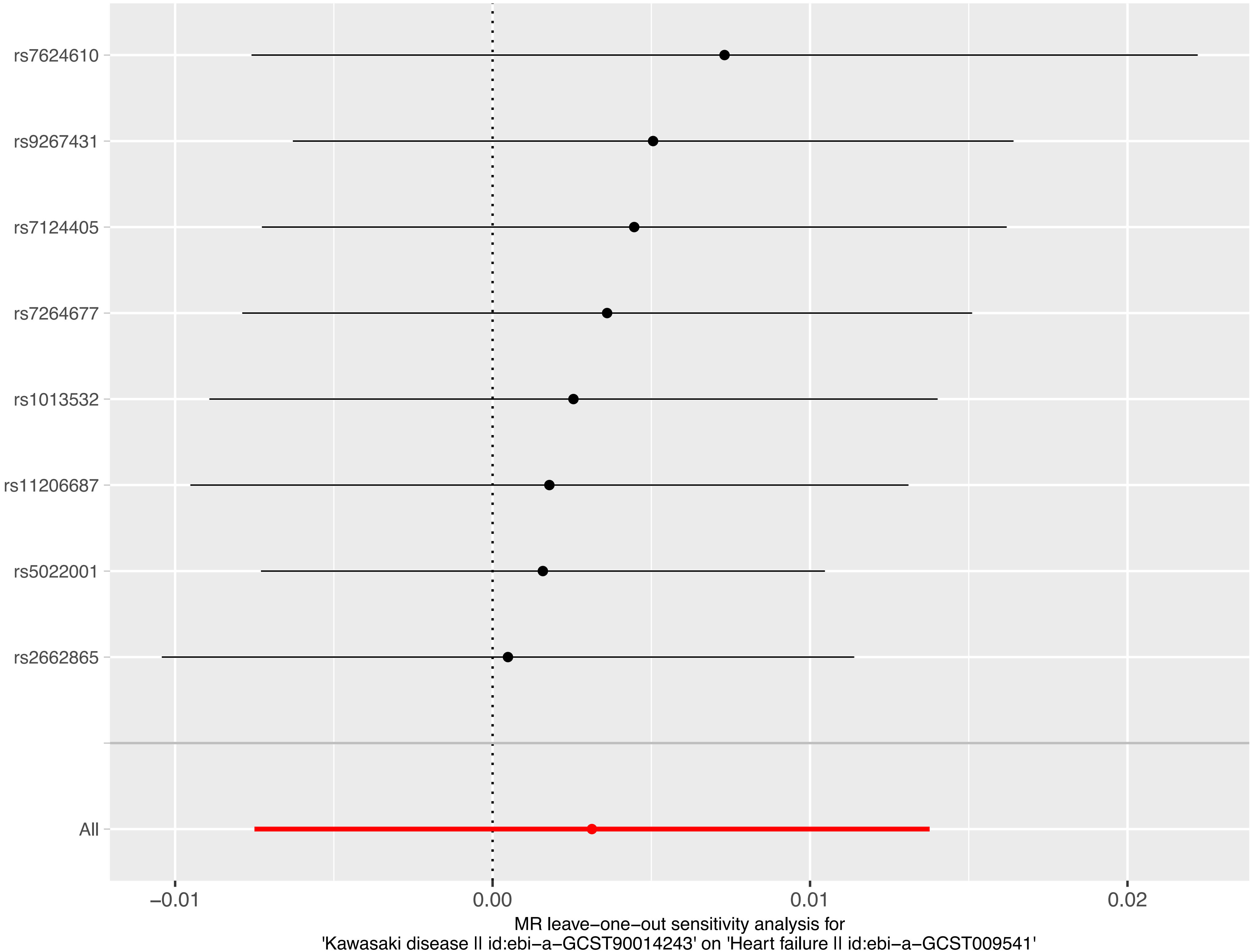
rs7624610
rs9267431
rs7124405
rs7264677
rs1013532
rs11206687
rs5022001
rs2662865
All
−0.01
0.00
0.01
0.02
MR leave−one−out sensitivity analysis for
'Kawasaki disease || id:ebi−a−GCST90014243' on 'Heart failure || id:ebi−a−GCST009541'

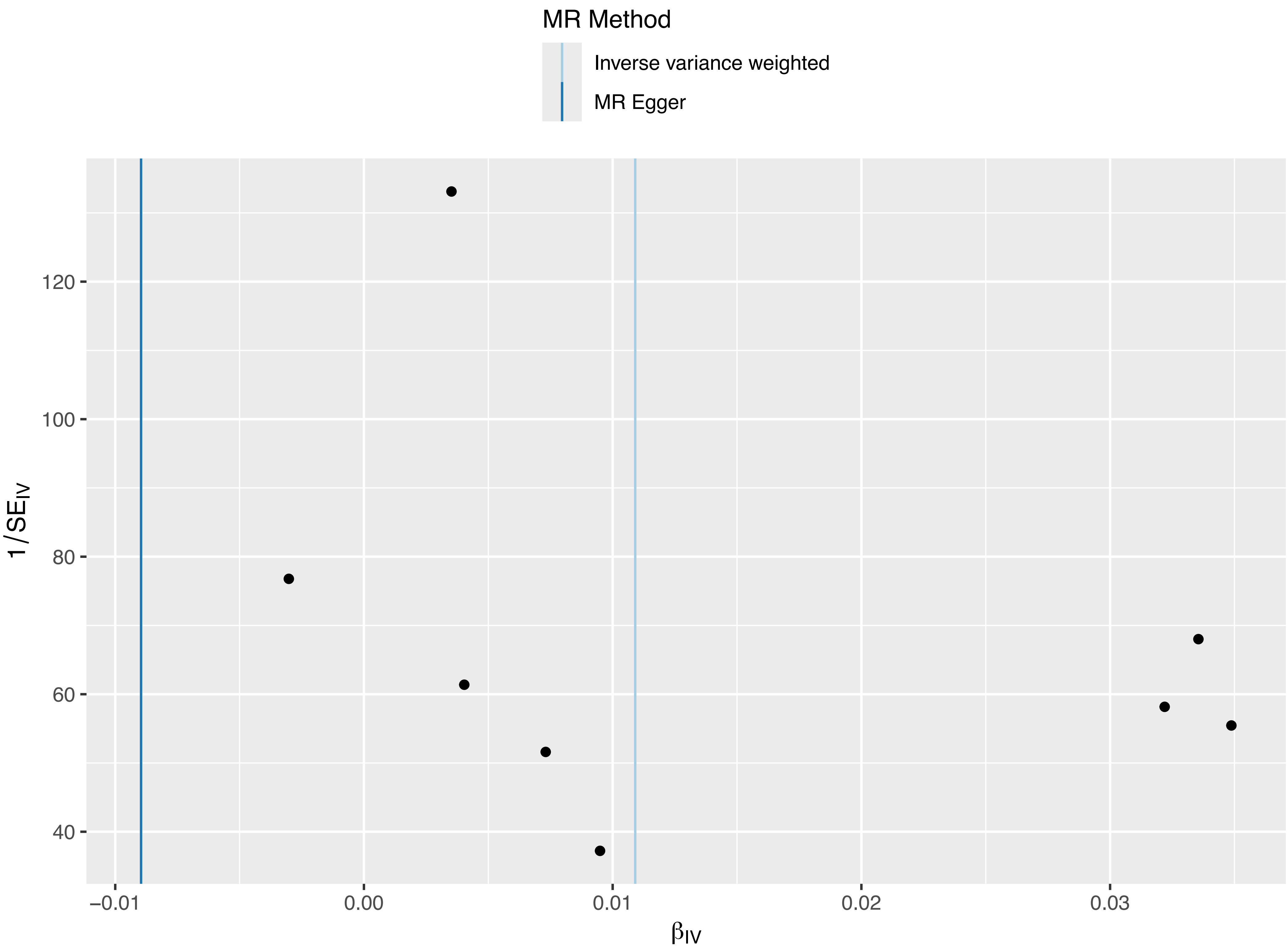

MR Method
Inverse variance weighted
MR Egger
120
100
80
60
40
1/SE$_{IV}$
−0.01
0.00
0.01
0.02
0.03
β$_{IV}$

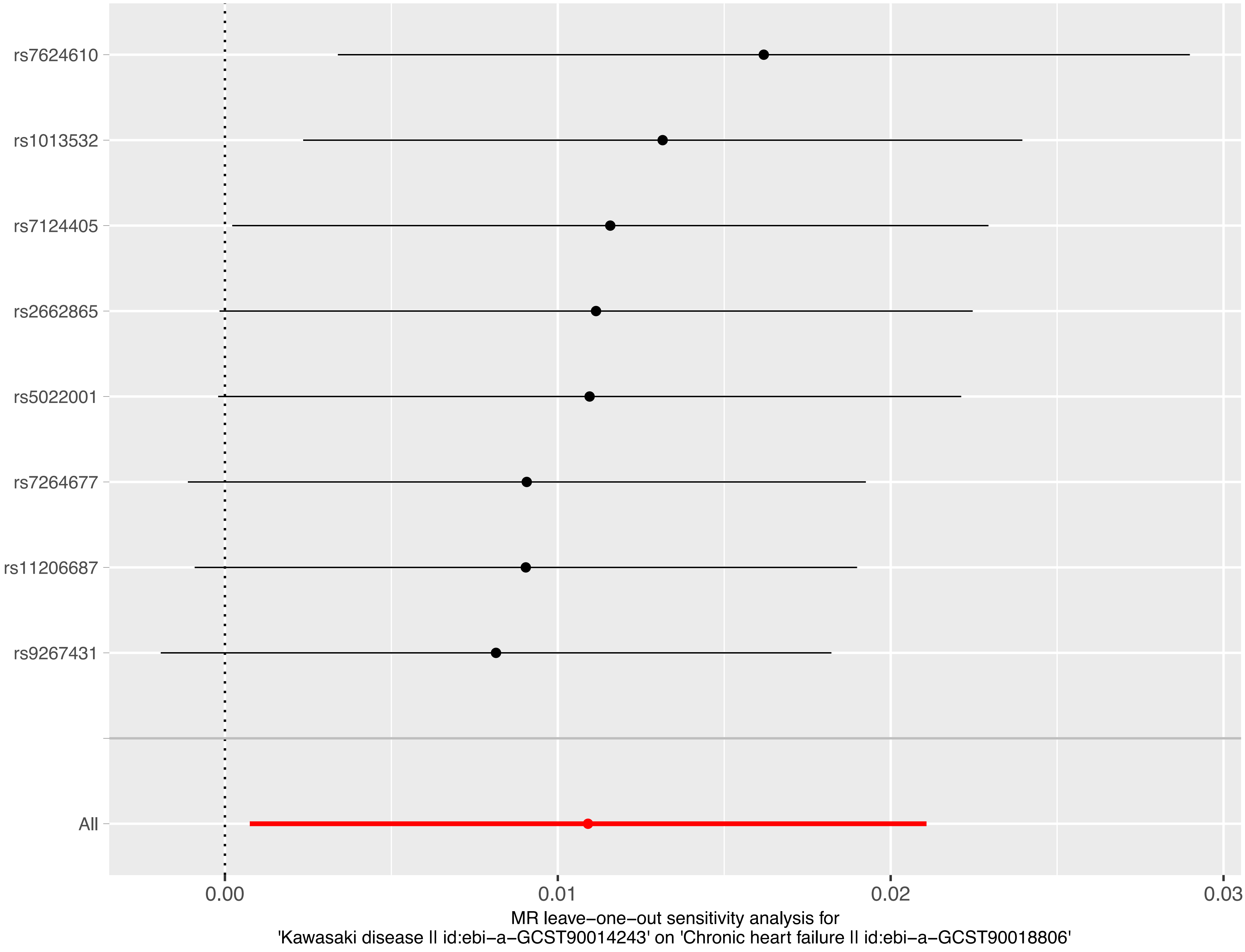
rs7624610
rs1013532
rs7124405
rs2662865
rs5022001
rs7264677
rs11206687
rs9267431
All
0.00
0.01
0.02
0.03
MR leave−one−out sensitivity analysis for
'Kawasaki disease || id:ebi−a−GCST90014243' on 'Chronic heart failure || id:ebi−a−GCST90018806'

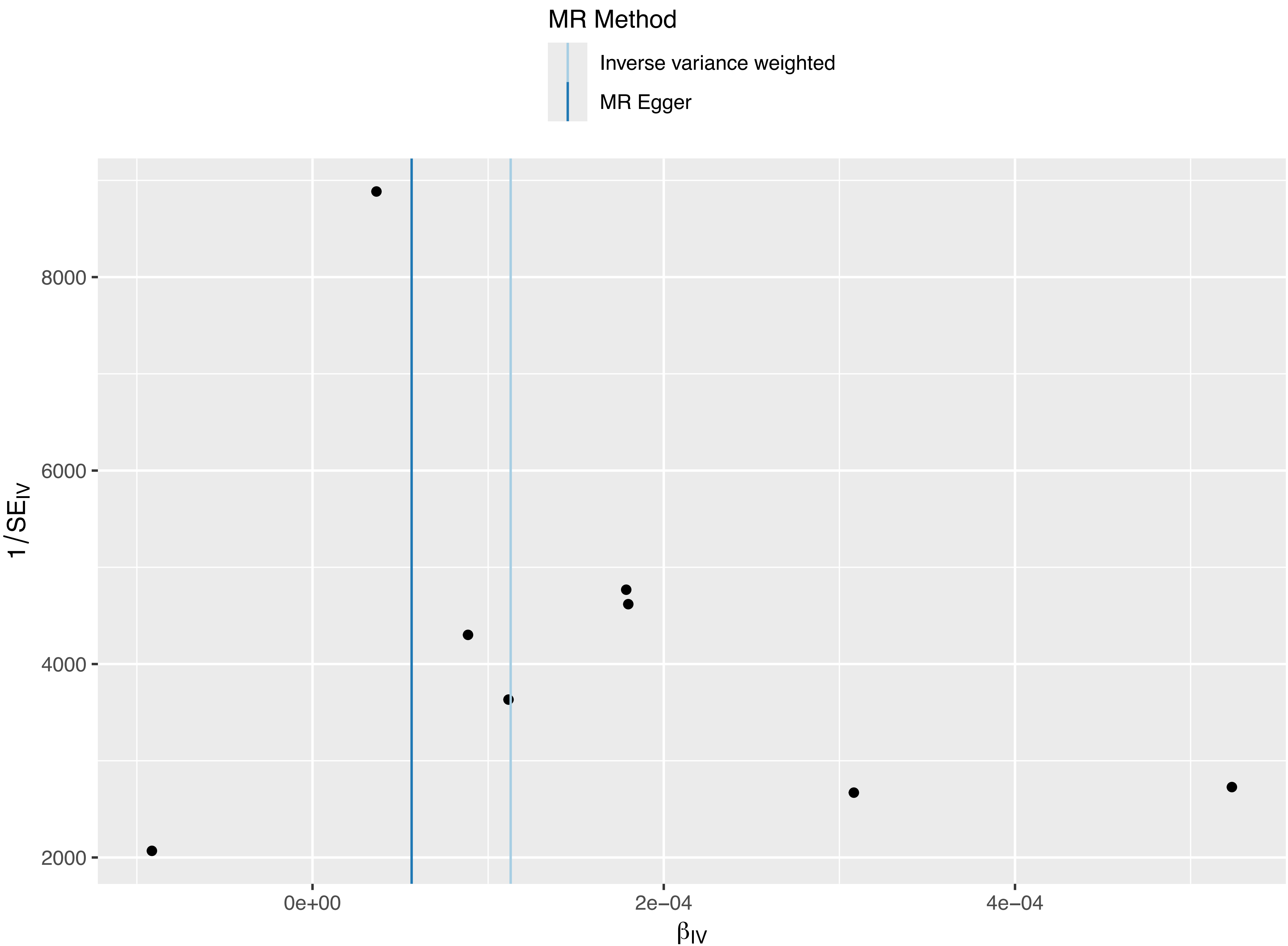
MR Method
Inverse variance weighted
MR Egger
8000
6000
4000
2000
1/SE$_{IV}$
0e+00
2e−04
4e−04
β_{IV}

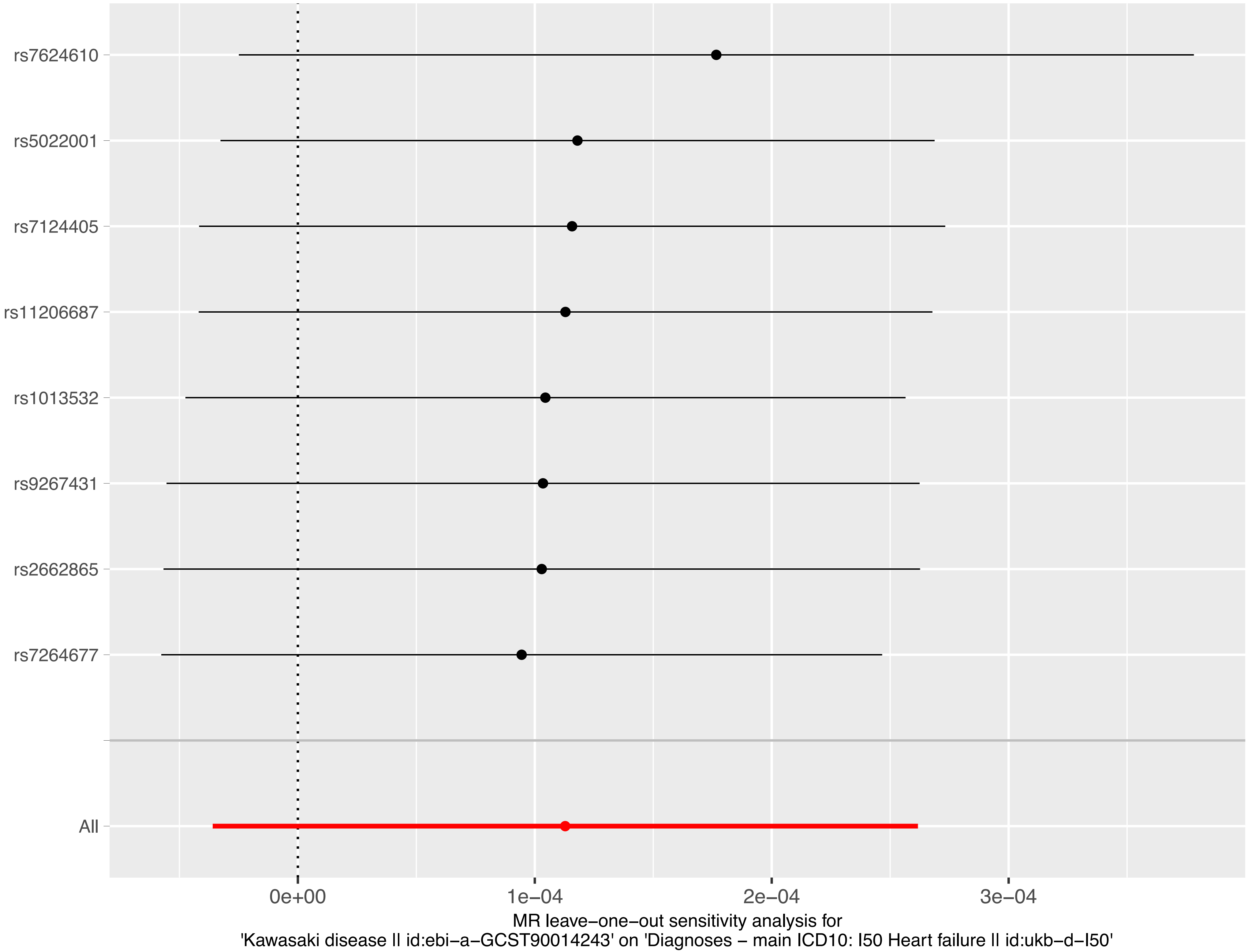
rs7624610
rs5022001
rs7124405
rs11206687
rs1013532
rs9267431
rs2662865
rs7264677
All
0e+00
1e−04
2e−04
3e−04
MR leave−one−out sensitivity analysis for
'Kawasaki disease || id:ebi−a−GCST90014243' on 'Diagnoses − main ICD10: I50 Heart failure || id:ukb−d−I50'

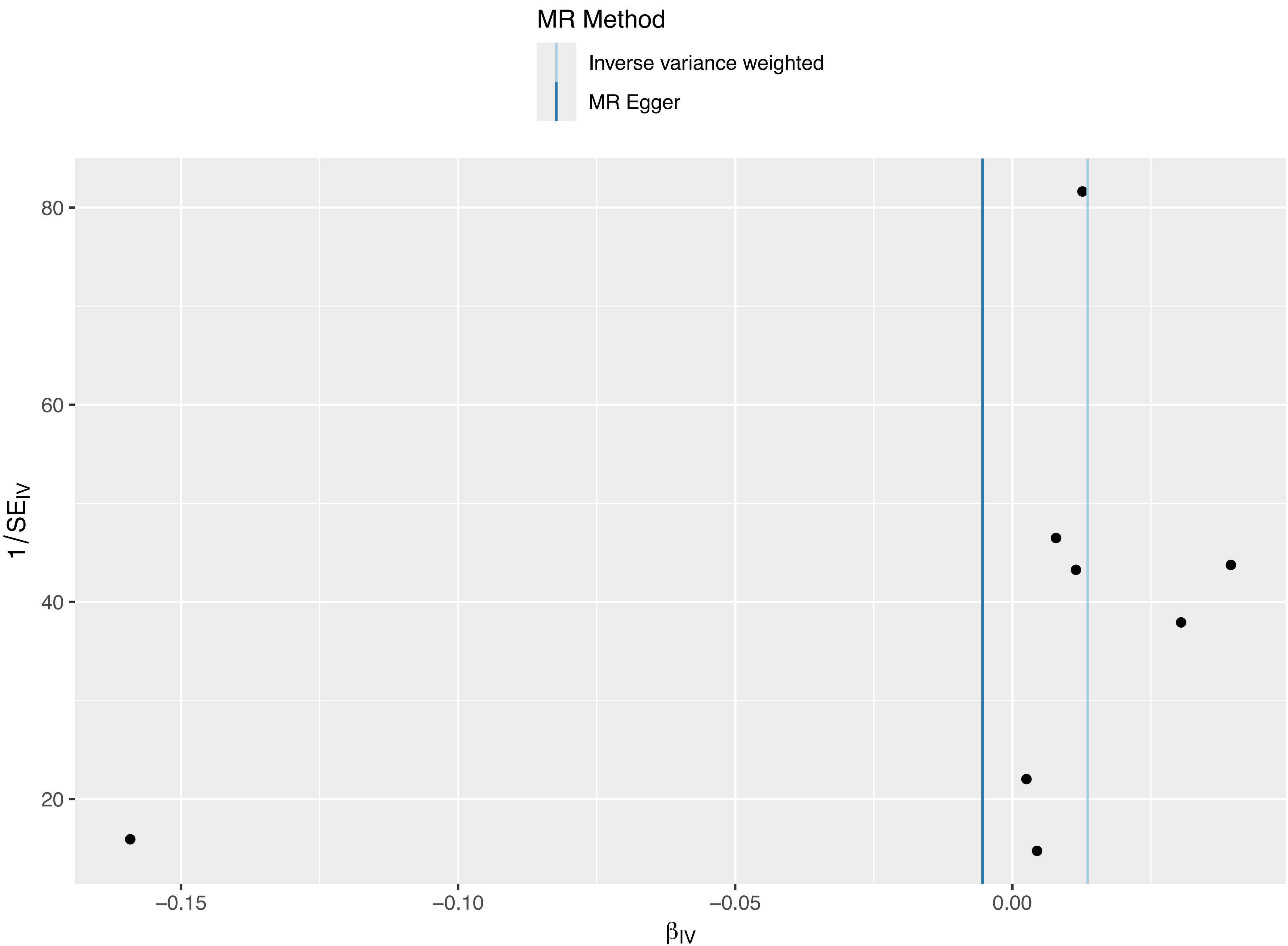
MR Method
Inverse variance weighted
MR Egger
80
60
40
20
$1/SE_{IV}$
−0.15
−0.10
−0.05
0.00
β_{IV}

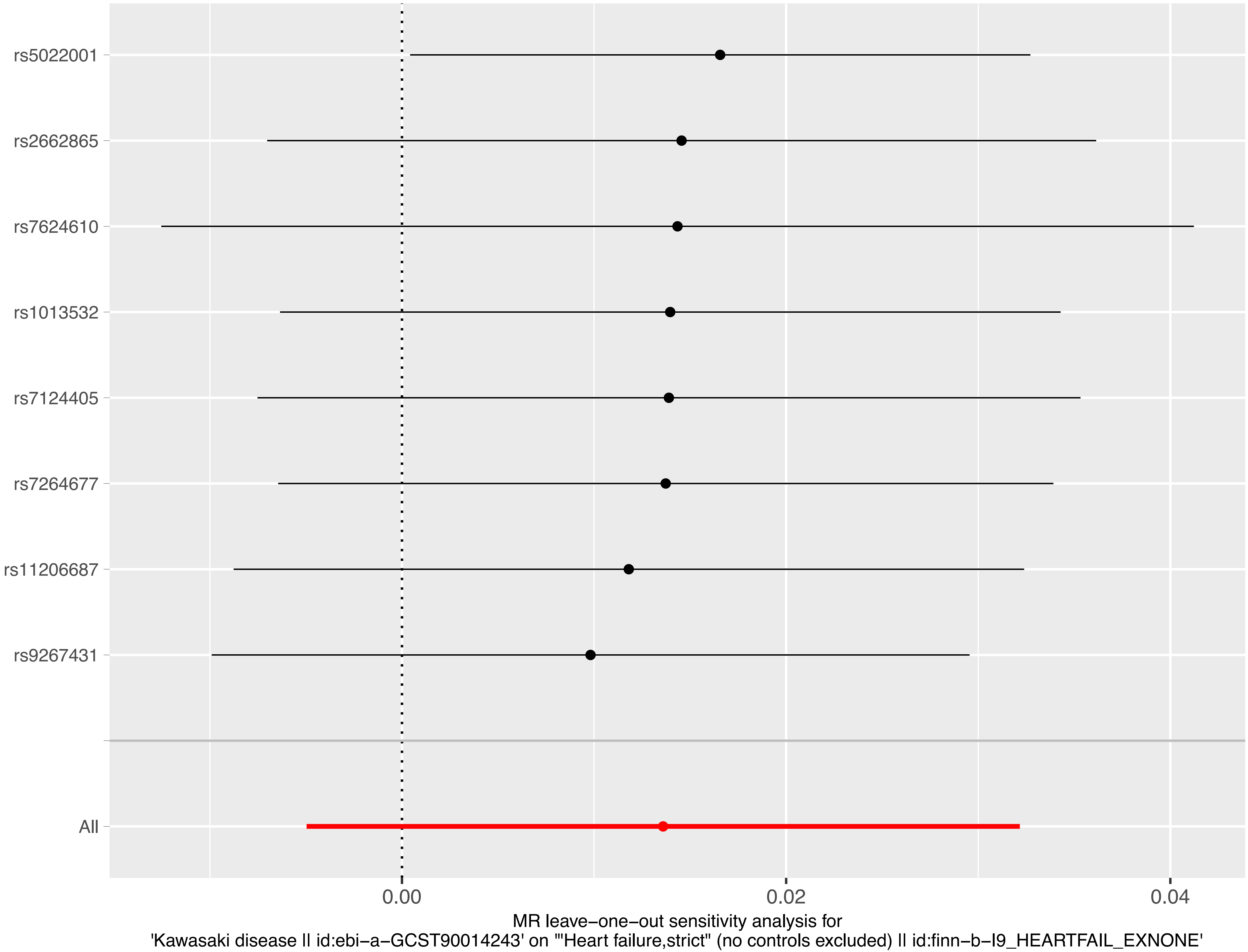

rs5022001
rs2662865
rs7624610
rs1013532
rs7124405
rs7264677
rs11206687
rs9267431
All
0.00
0.02
0.04
MR leave−one−out sensitivity analysis for
'Kawasaki disease || id:ebi−a−GCST90014243' on '"Heart failure,strict" (no controls excluded) || id:finn−b−I9_HEARTFAIL_EXNONE'

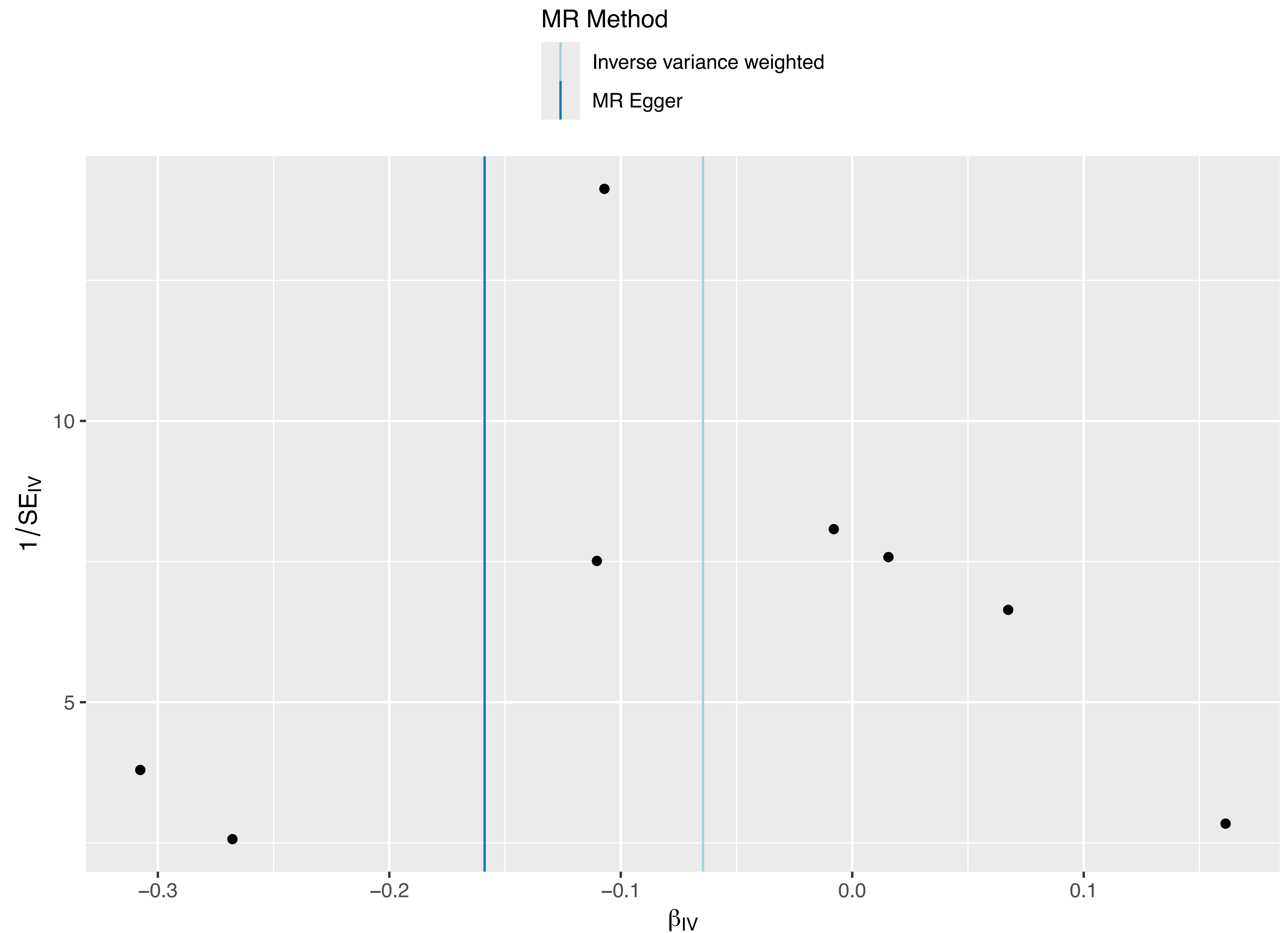

MR Method
Inverse variance weighted
MR Egger
10
5
1/SE$_{IV}$
−0.3
−0.2
−0.1
0.0
0.1
β$_{IV}$

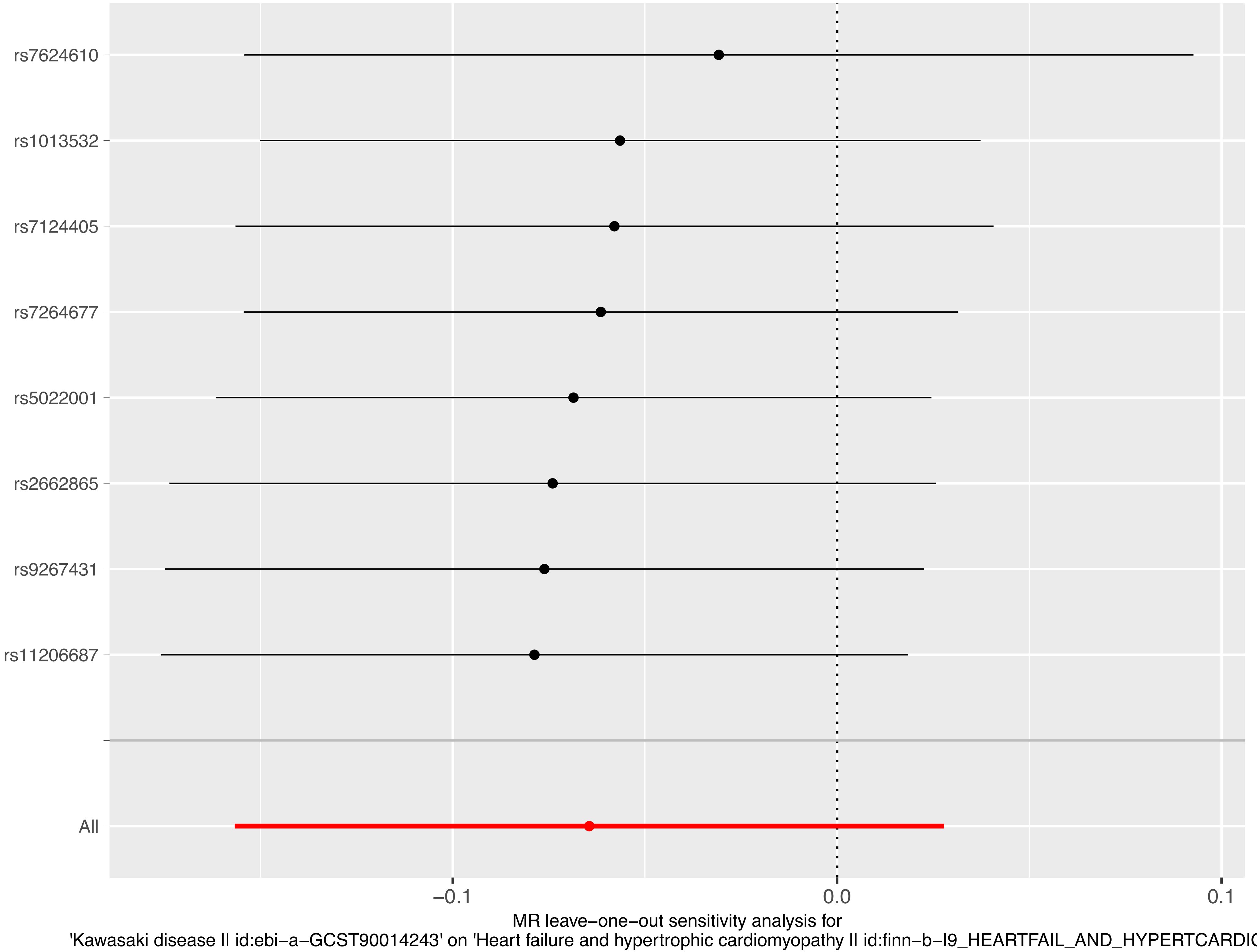

rs7624610
rs1013532
rs7124405
rs7264677
rs5022001
rs2662865
rs9267431
rs11206687
All
−0.1
0.0
0.1
MR leave−one−out sensitivity analysis for
'Kawasaki disease || id:ebi−a−GCST90014243' on 'Heart failure and hypertrophic cardiomyopathy || id:finn−b−I9_HEARTFAIL_AND_HYPERTCARDI(

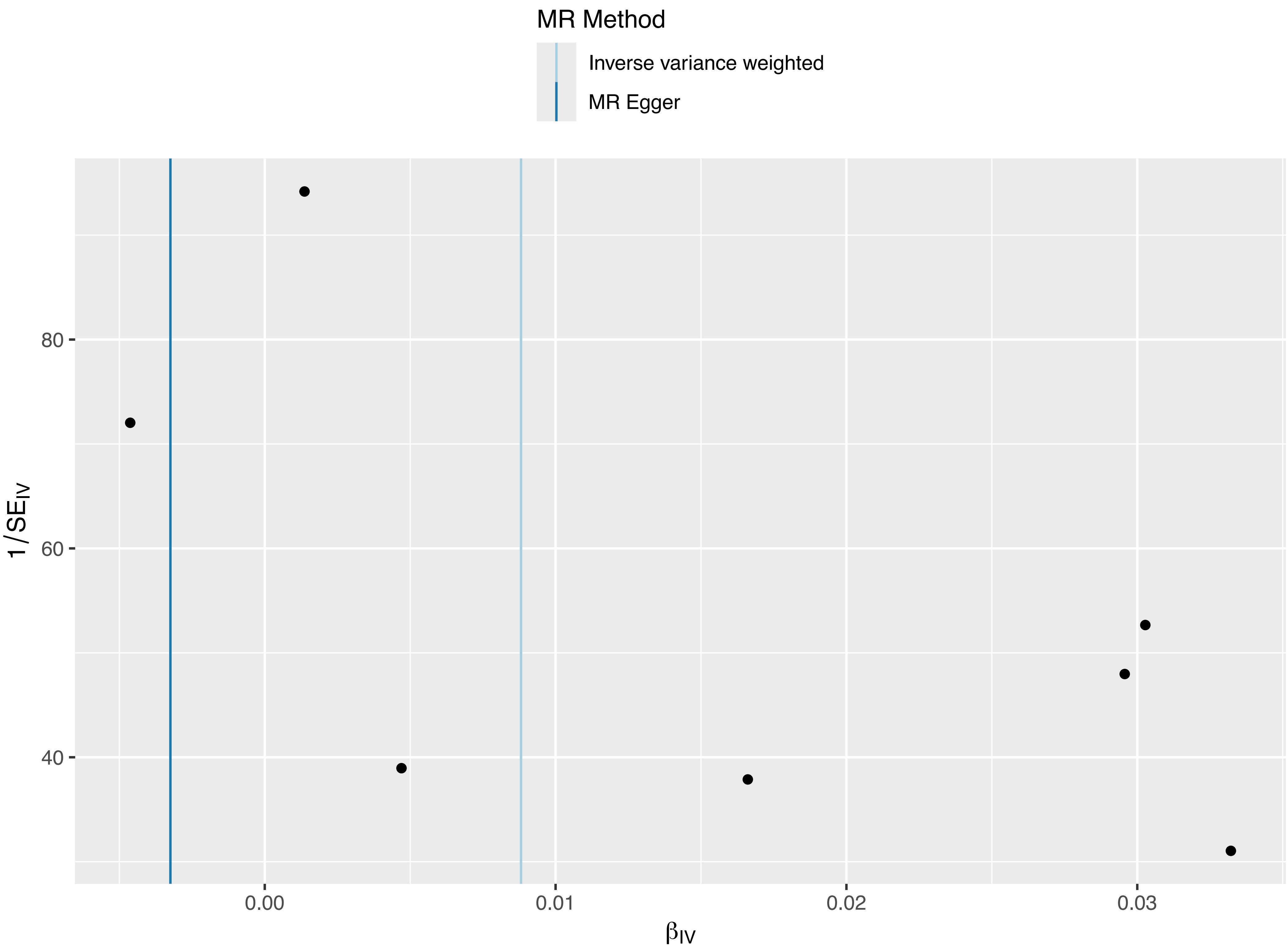

MR Method
Inverse variance weighted
MR Egger
80
60
40
1/SE_IV
0.00
0.01
0.02
0.03
β_IV

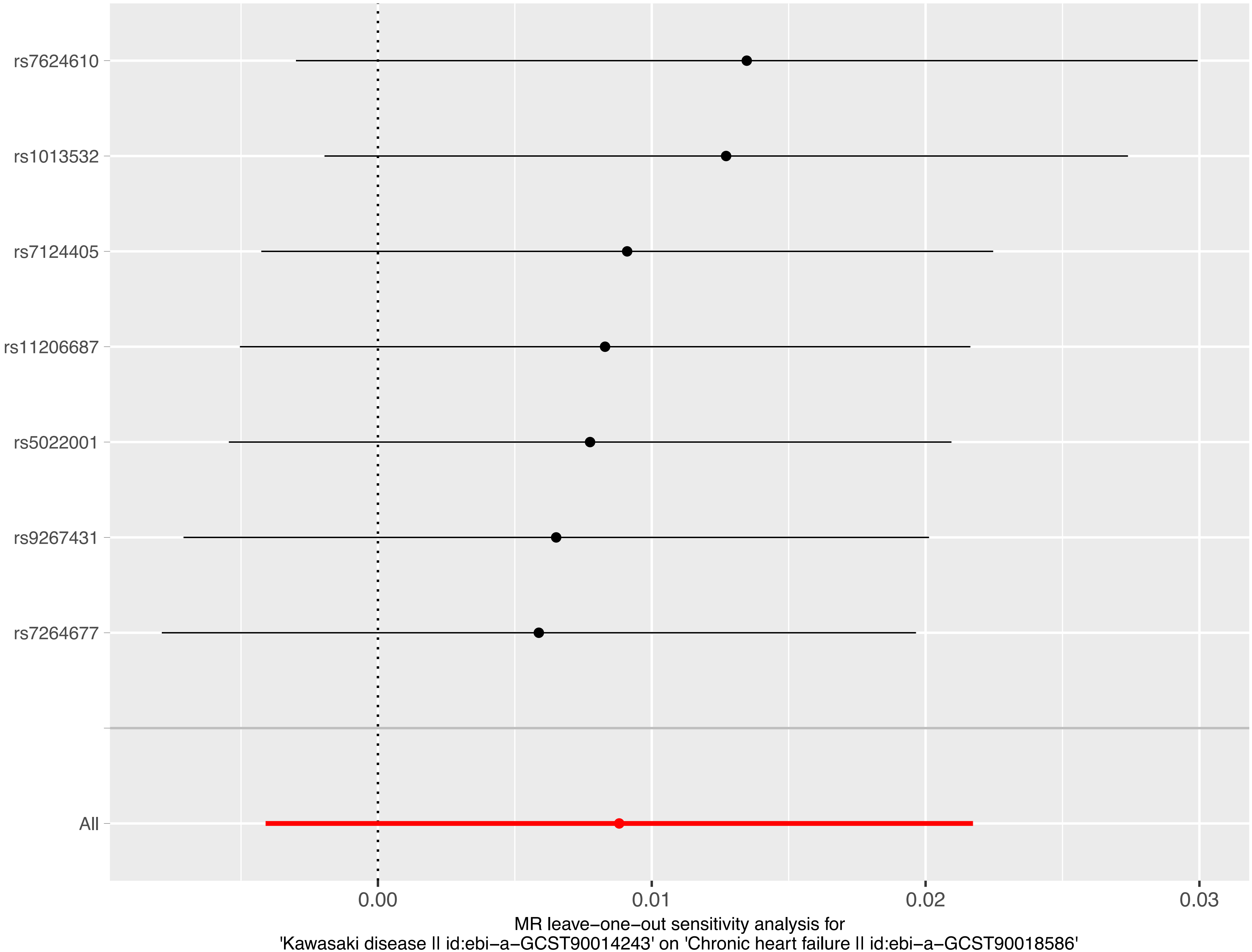

rs7624610
rs1013532
rs7124405
rs11206687
rs5022001
rs9267431
rs7264677
All
0.00
0.01
0.02
0.03
MR leave−one−out sensitivity analysis for
'Kawasaki disease || id:ebi−a−GCST90014243' on 'Chronic heart failure || id:ebi−a−GCST90018586'

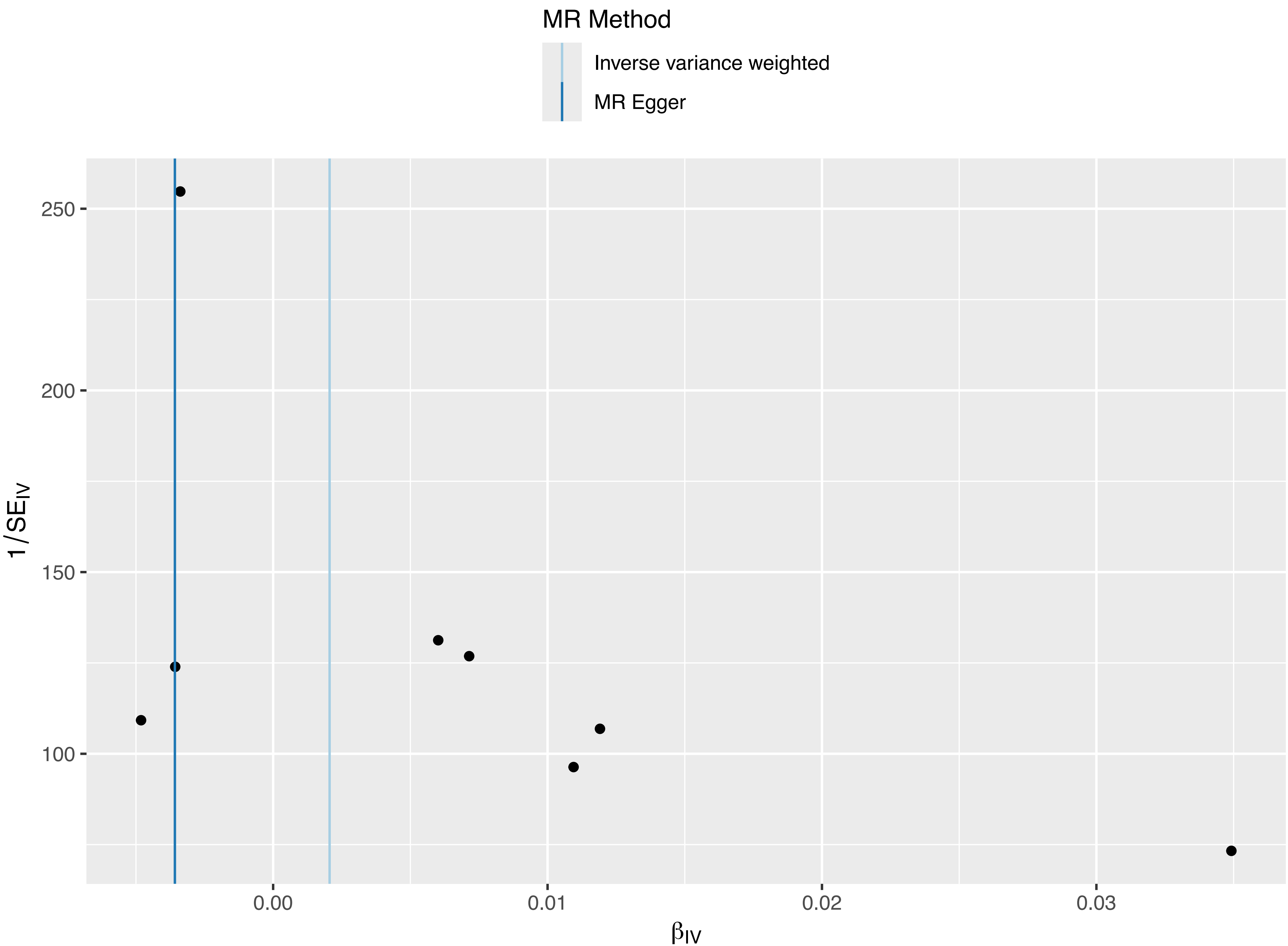

MR Method
Inverse variance weighted
MR Egger
250
200
150
100
1/SE$_{IV}$
0.00
0.01
0.02
0.03
β$_{IV}$

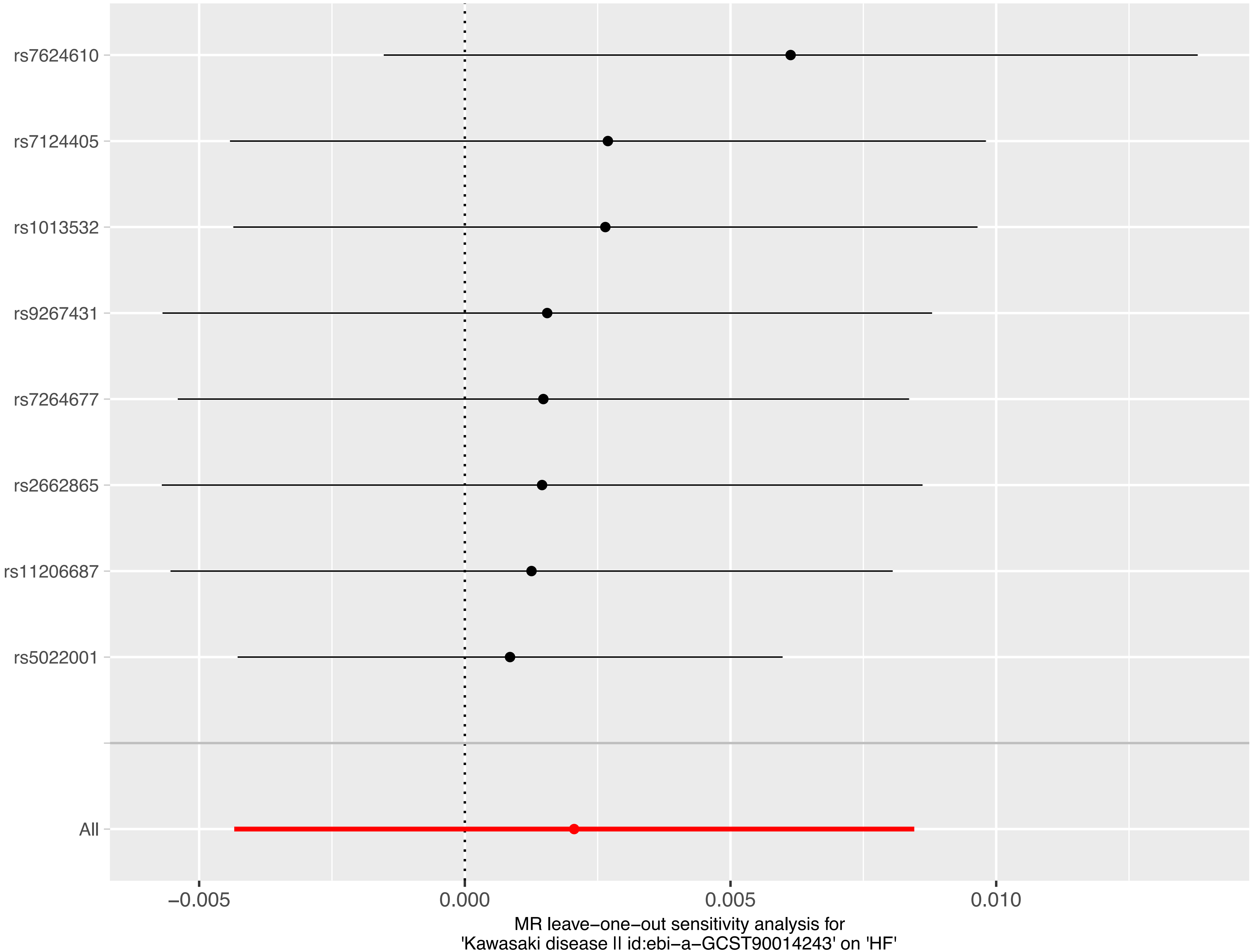
rs7624610
rs7124405
rs1013532
rs9267431
rs7264677
rs2662865
rs11206687
rs5022001
All
−0.005
0.000
0.005
0.010
MR leave−one−out sensitivity analysis for
'Kawasaki disease II id:ebi−a−GCST90014243' on 'HF'

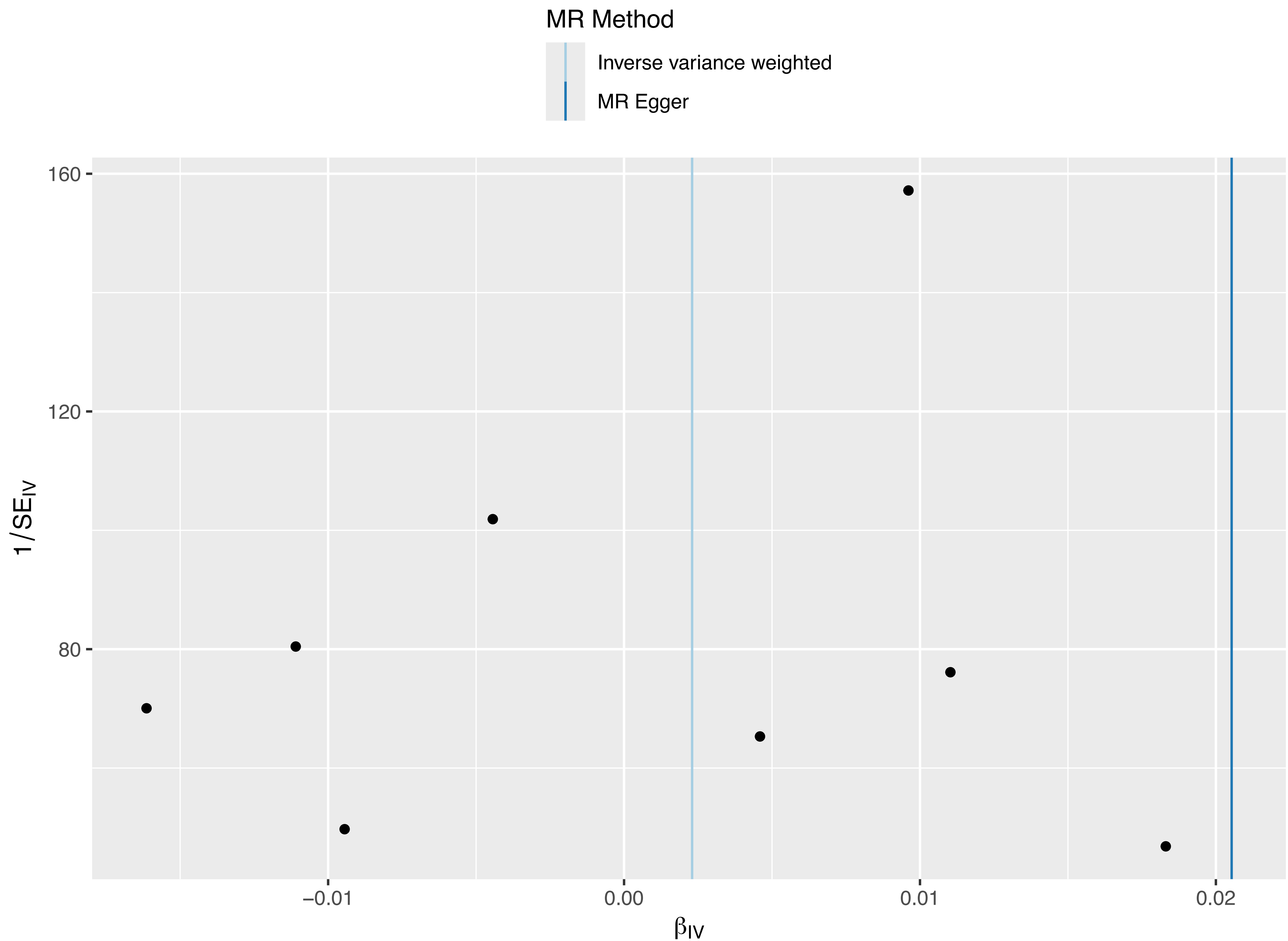
MR Method
Inverse variance weighted
MR Egger
160
120
80
1/SE$_{IV}$
−0.01
0.00
0.01
0.02
β$_{IV}$

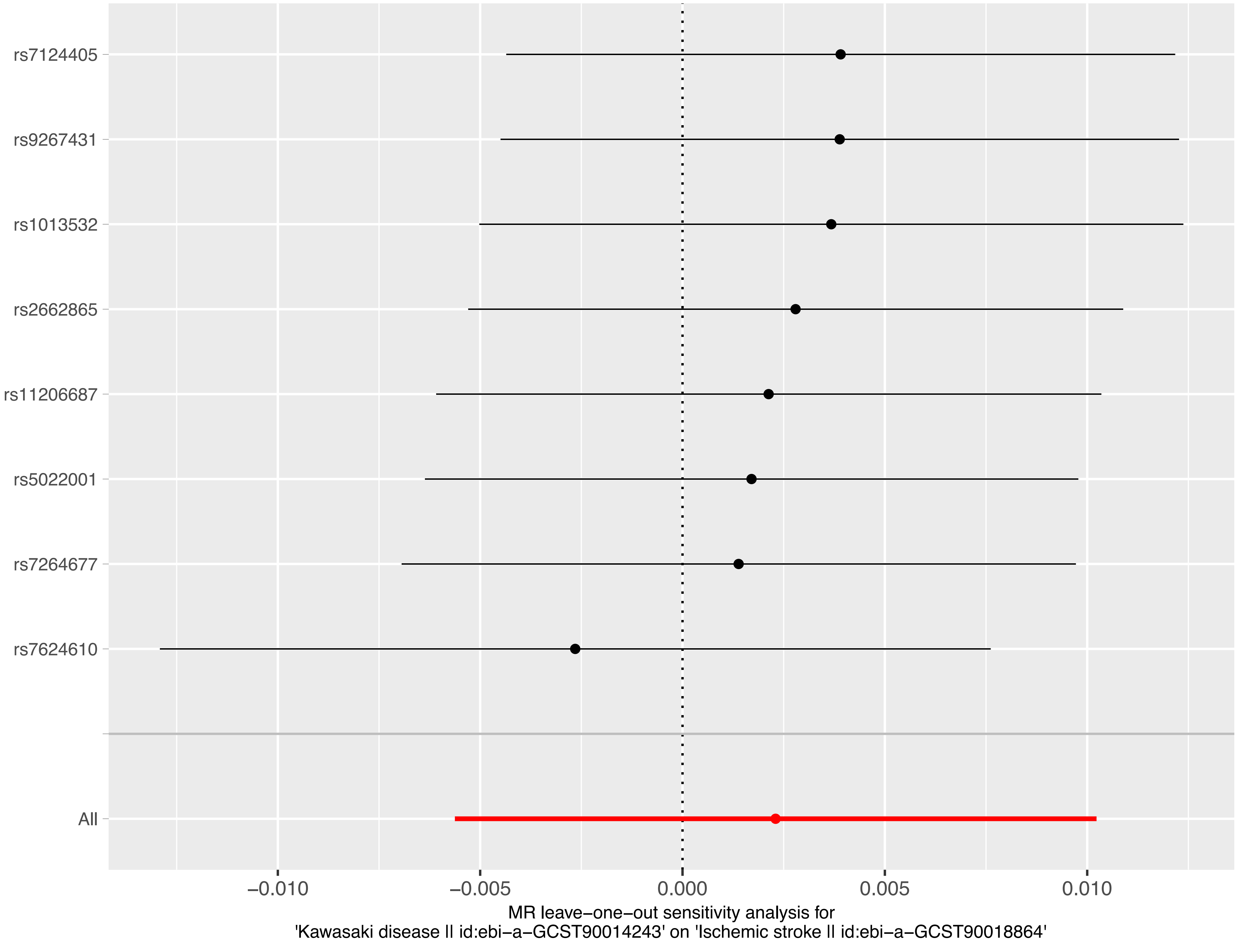
rs7124405
rs9267431
rs1013532
rs2662865
rs11206687
rs5022001
rs7264677
rs7624610
All
−0.010
−0.005
0.000
0.005
0.010
MR leave−one−out sensitivity analysis for
'Kawasaki disease || id:ebi−a−GCST90014243' on 'Ischemic stroke || id:ebi−a−GCST90018864'

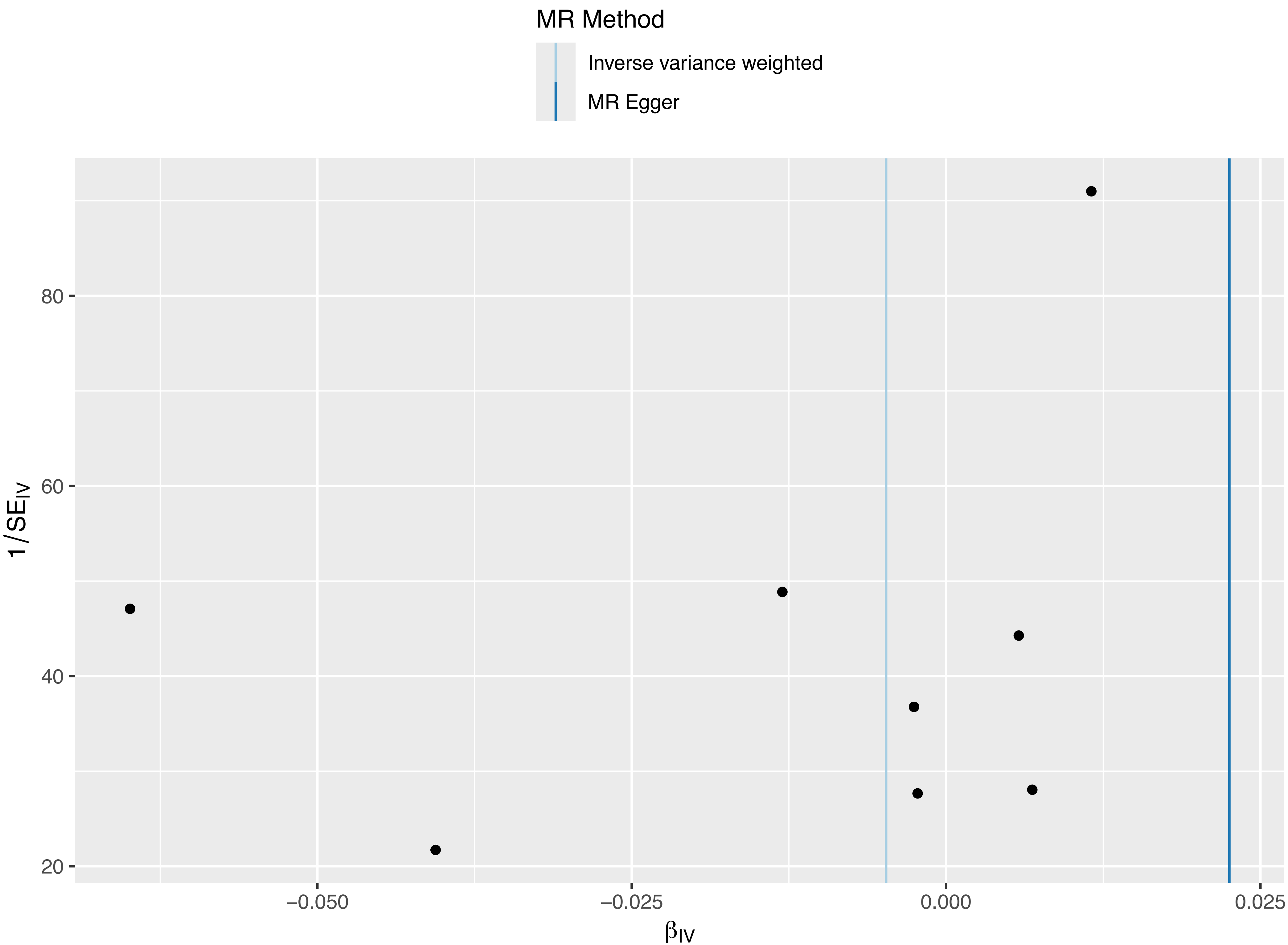

MR Method
Inverse variance weighted
MR Egger
80
60
40
20
1/SE$_{IV}$
−0.050
−0.025
0.000
0.025
β$_{IV}$

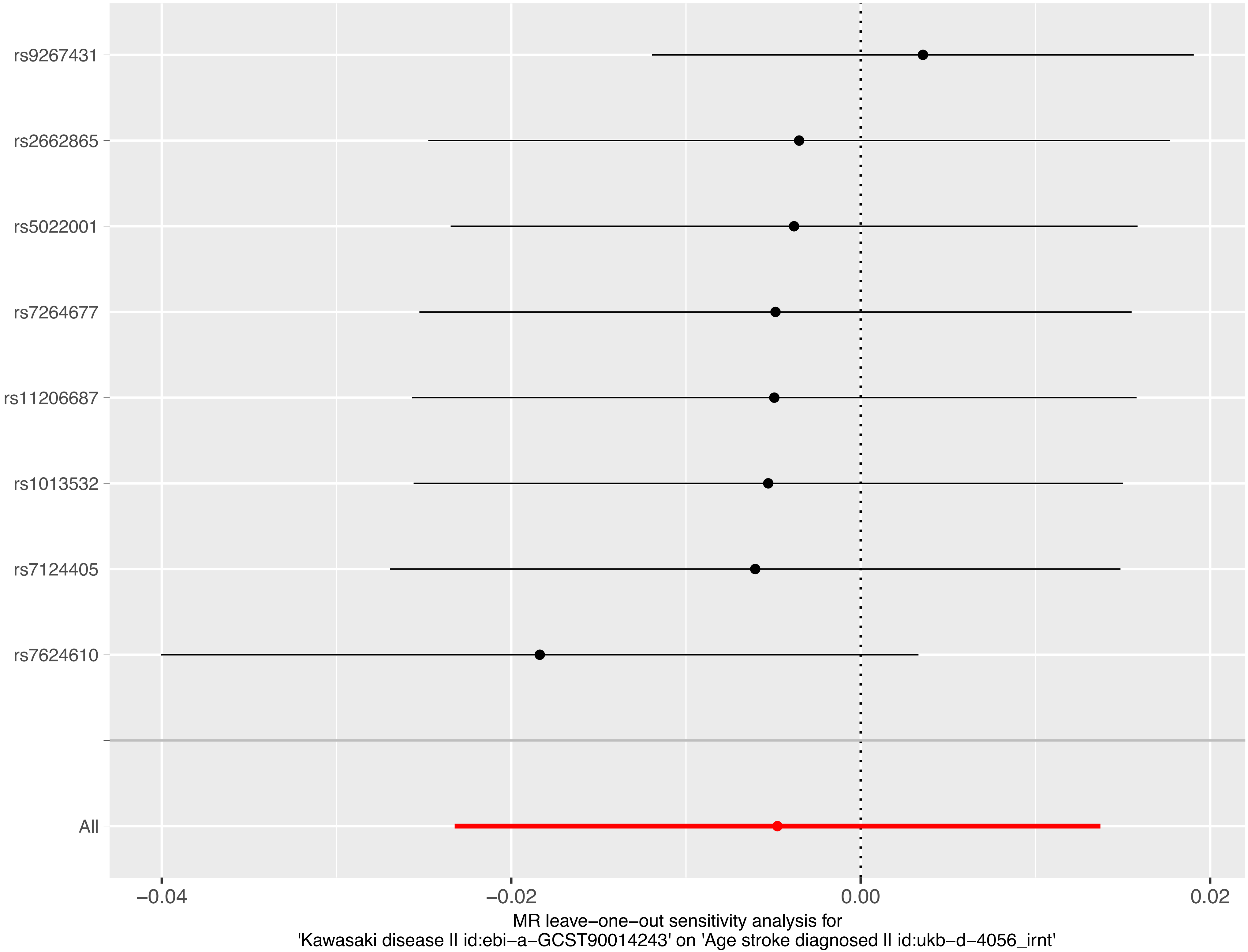
rs9267431
rs2662865
rs5022001
rs7264677
rs11206687
rs1013532
rs7124405
rs7624610
All
−0.04
−0.02
0.00
0.02
MR leave−one−out sensitivity analysis for
'Kawasaki disease || id:ebi−a−GCST90014243' on 'Age stroke diagnosed || id:ukb−d−4056_irnt'

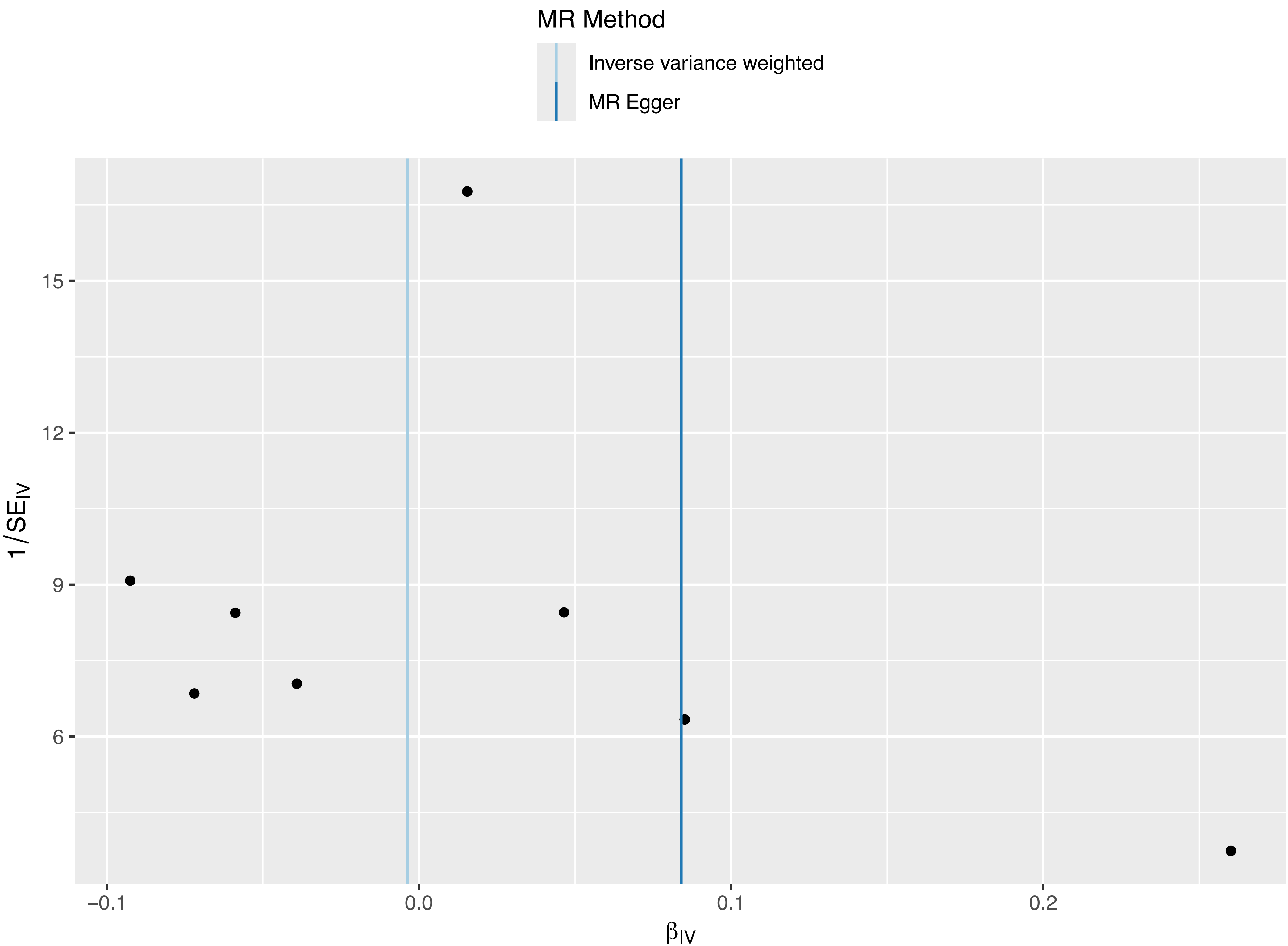

MR Method
Inverse variance weighted
MR Egger
15
12
9
6
1/SE_IV
−0.1
0.0
0.1
0.2
β_IV

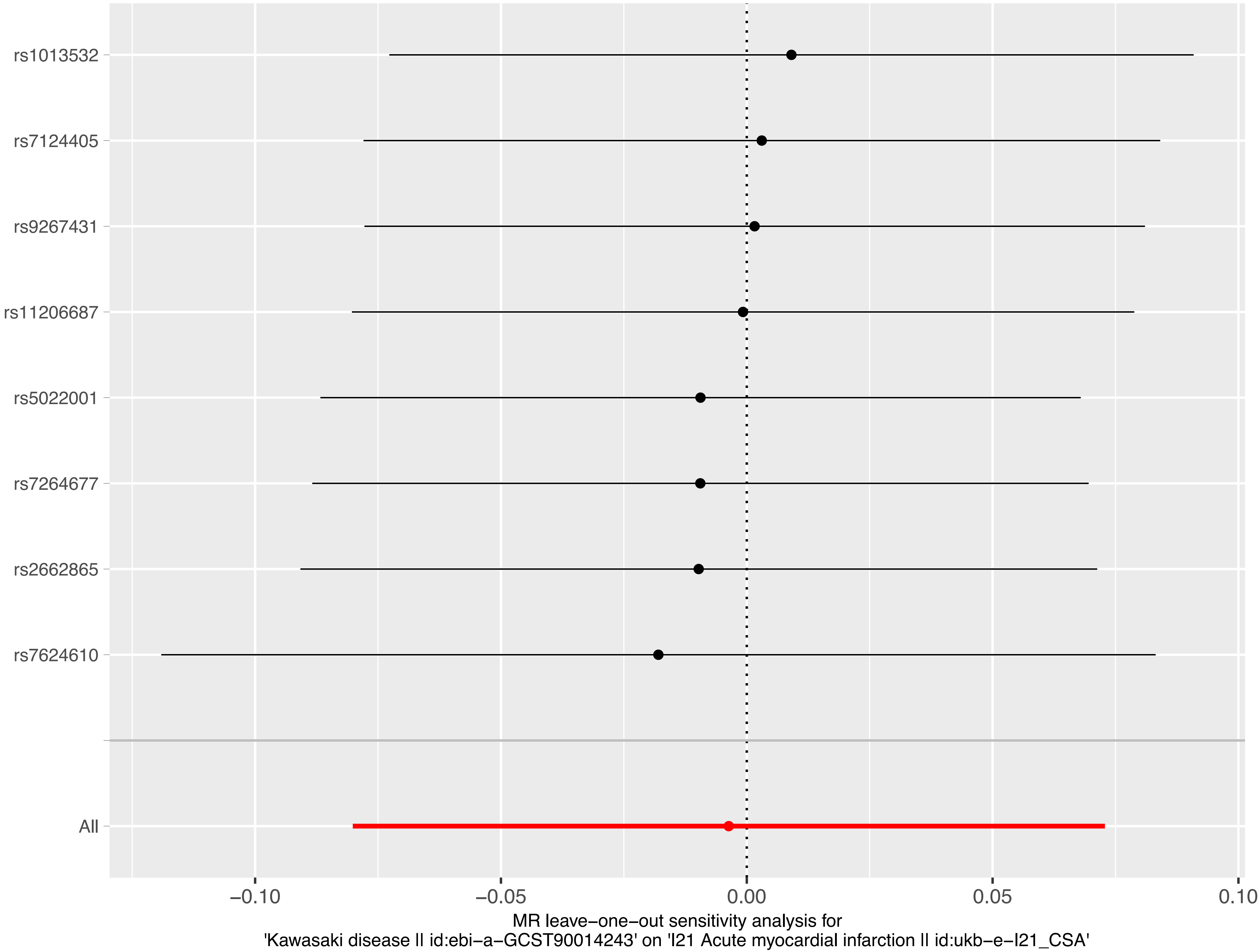
rs1013532
rs7124405
rs9267431
rs11206687
rs5022001
rs7264677
rs2662865
rs7624610
All
−0.10
−0.05
0.00
0.05
0.10
MR leave−one−out sensitivity analysis for
'Kawasaki disease || id:ebi−a−GCST90014243' on 'I21 Acute myocardial infarction || id:ukb−e−I21_CSA'

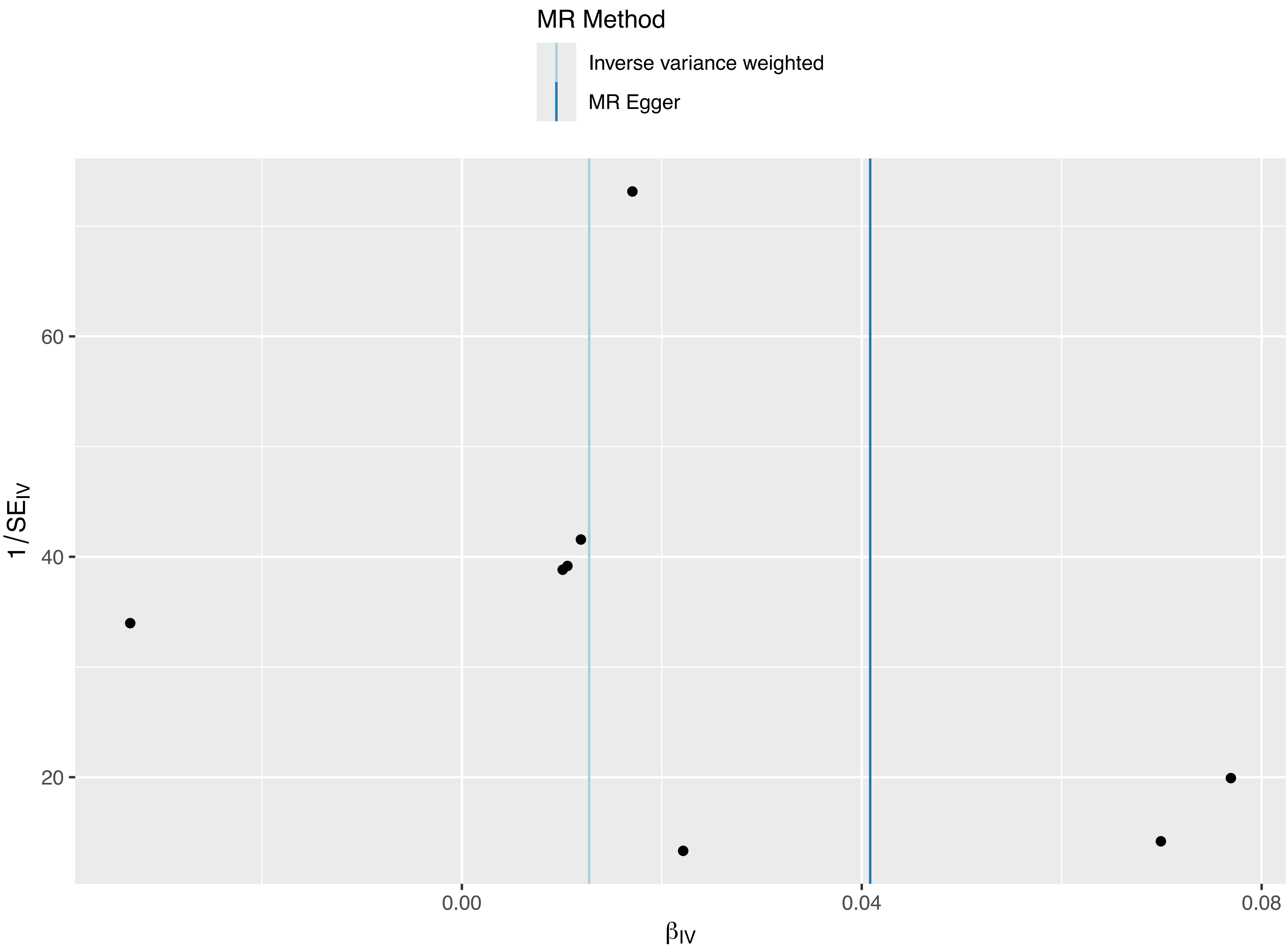
MR Method
Inverse variance weighted
MR Egger
60
40
20
1/SE_IV
0.00
0.04
0.08
β_IV

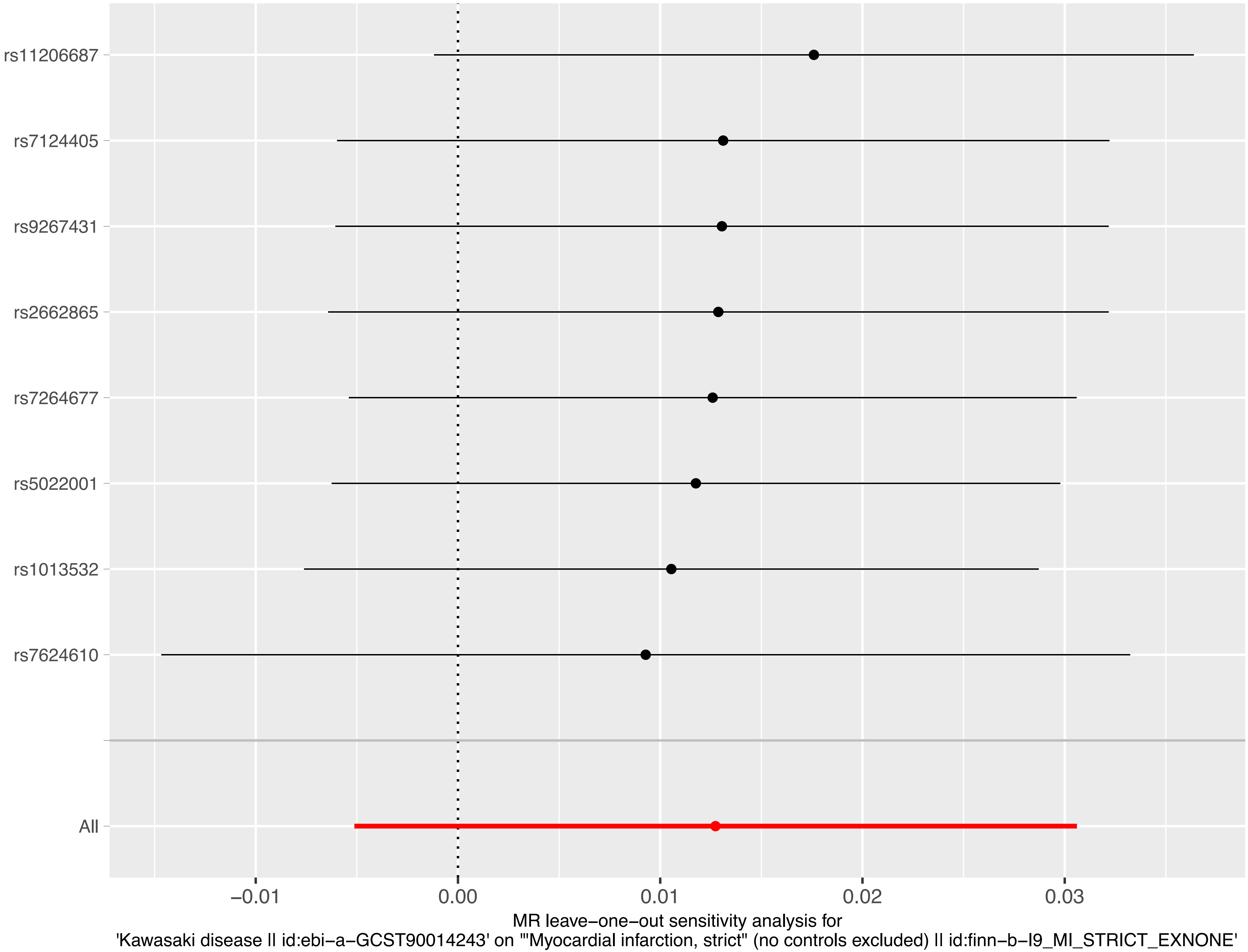

rs11206687
rs7124405
rs9267431
rs2662865
rs7264677
rs5022001
rs1013532
rs7624610
All
−0.01
0.00
0.01
0.02
0.03
MR leave−one−out sensitivity analysis for
'Kawasaki disease || id:ebi−a−GCST90014243' on '"Myocardial infarction, strict" (no controls excluded) || id:finn−b−I9_MI_STRICT_EXNONE'

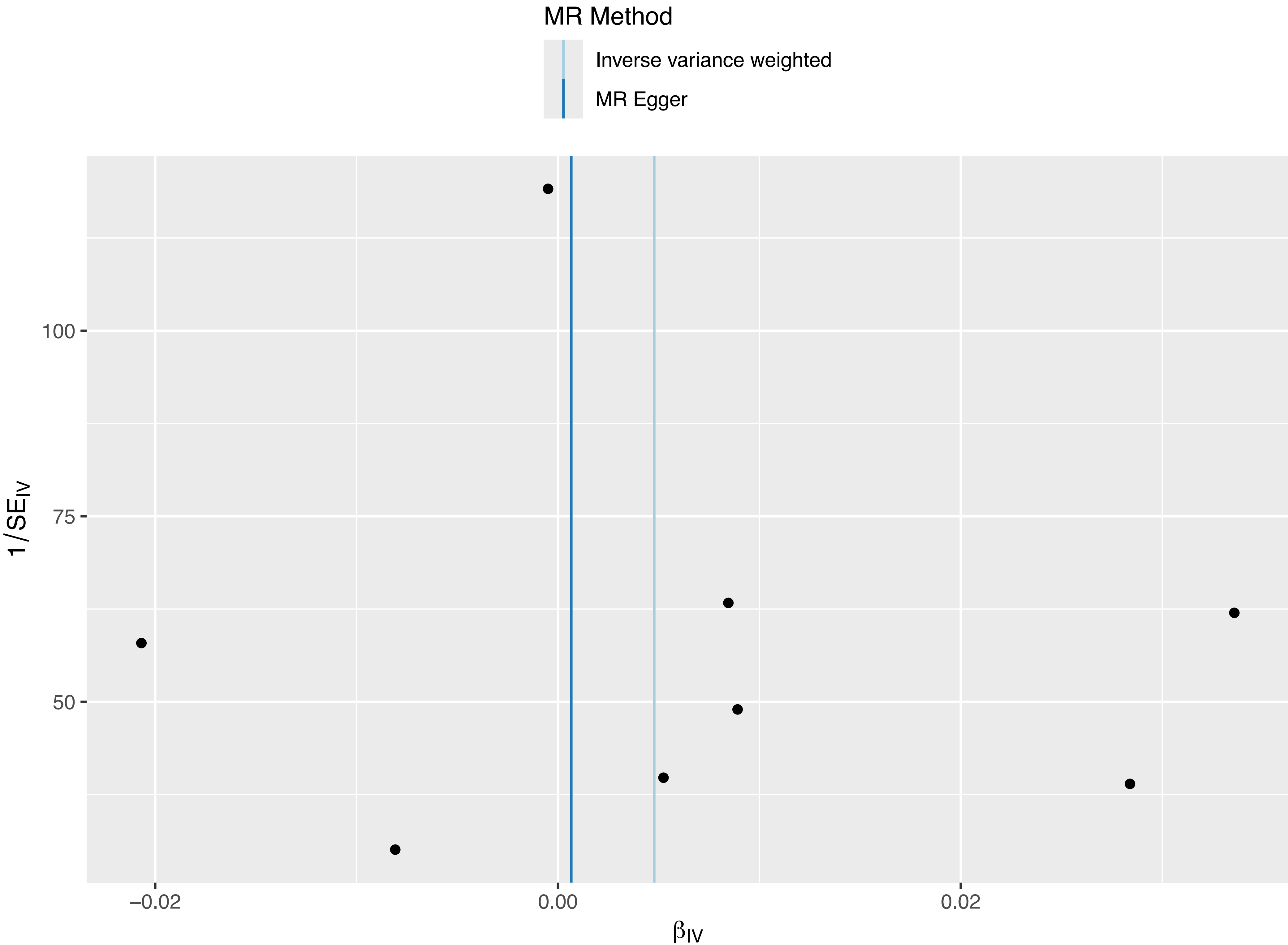

MR Method
Inverse variance weighted
MR Egger
100
75
50
1/SE_IV
−0.02
0.00
0.02
β_IV

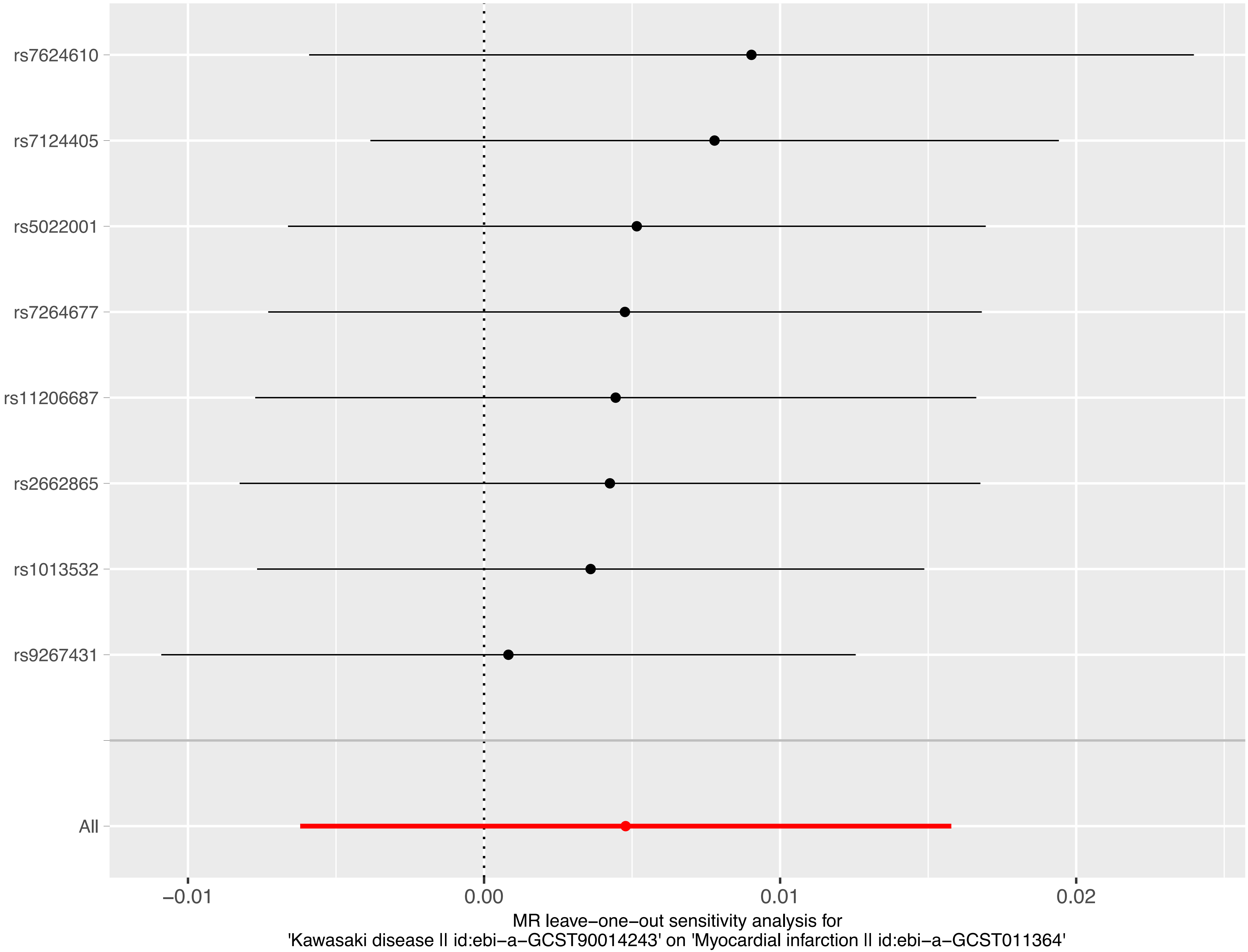

rs7624610
rs7124405
rs5022001
rs7264677
rs11206687
rs2662865
rs1013532
rs9267431
All
−0.01
0.00
0.01
0.02
MR leave−one−out sensitivity analysis for
'Kawasaki disease || id:ebi−a−GCST90014243' on 'Myocardial infarction || id:ebi−a−GCST011364'

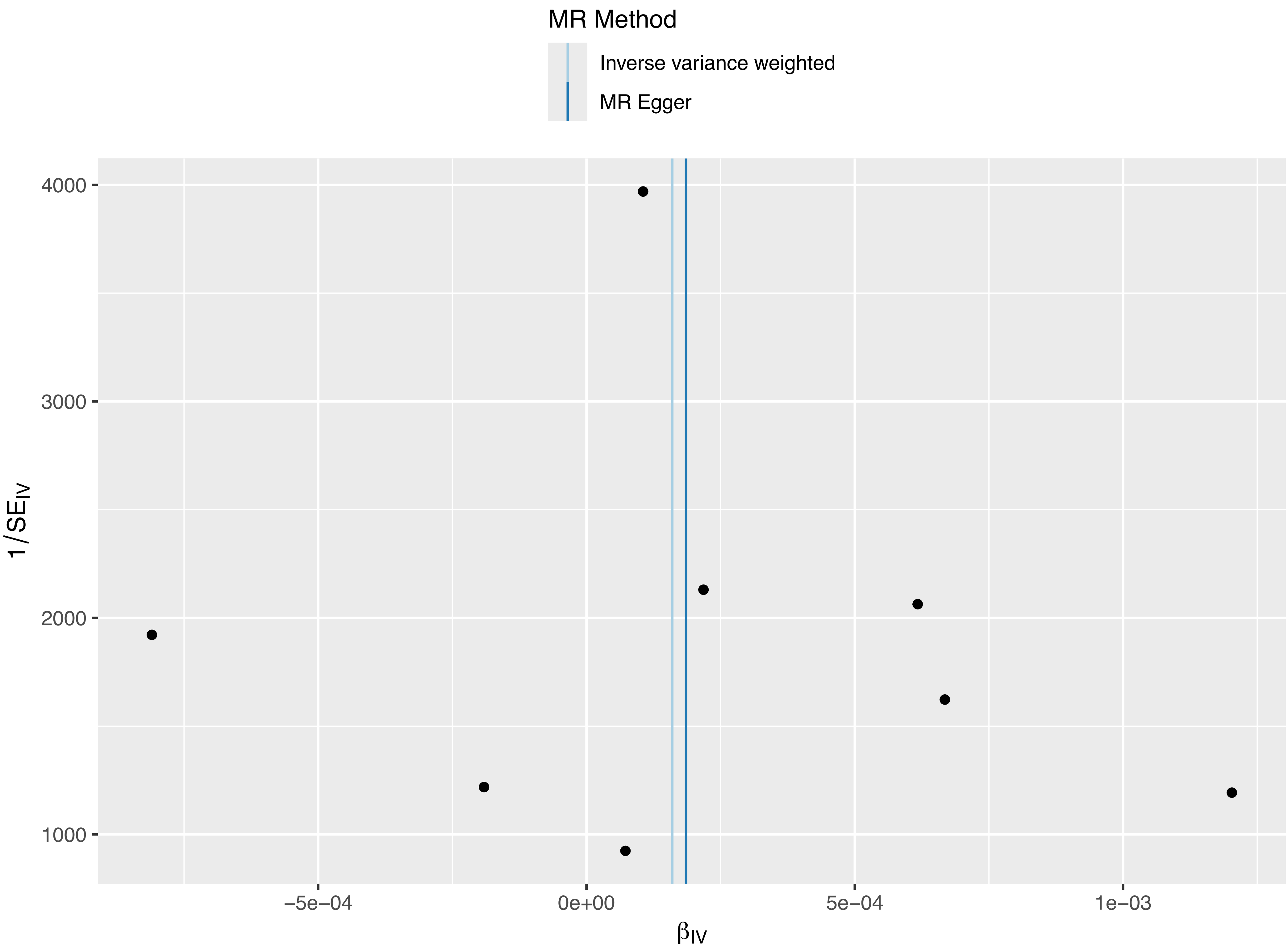

MR Method
Inverse variance weighted
MR Egger
4000
3000
2000
1000
1/SE_IV
−5e−04
0e+00
5e−04
1e−03
β_IV

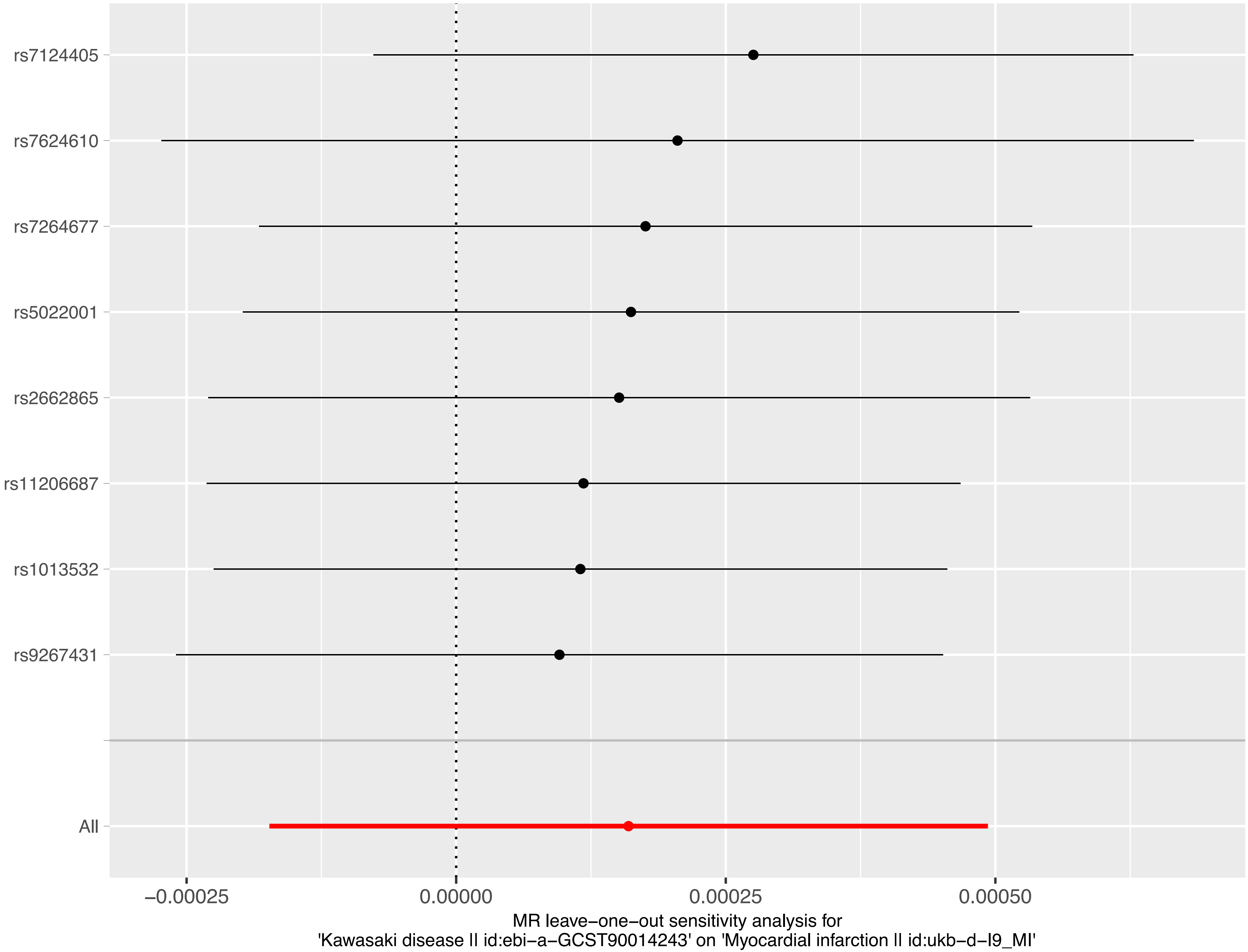
rs7124405
rs7624610
rs7264677
rs5022001
rs2662865
rs11206687
rs1013532
rs9267431
All
−0.00025
0.00000
0.00025
0.00050
MR leave−one−out sensitivity analysis for
'Kawasaki disease || id:ebi−a−GCST90014243' on 'Myocardial infarction || id:ukb−d−I9_MI'

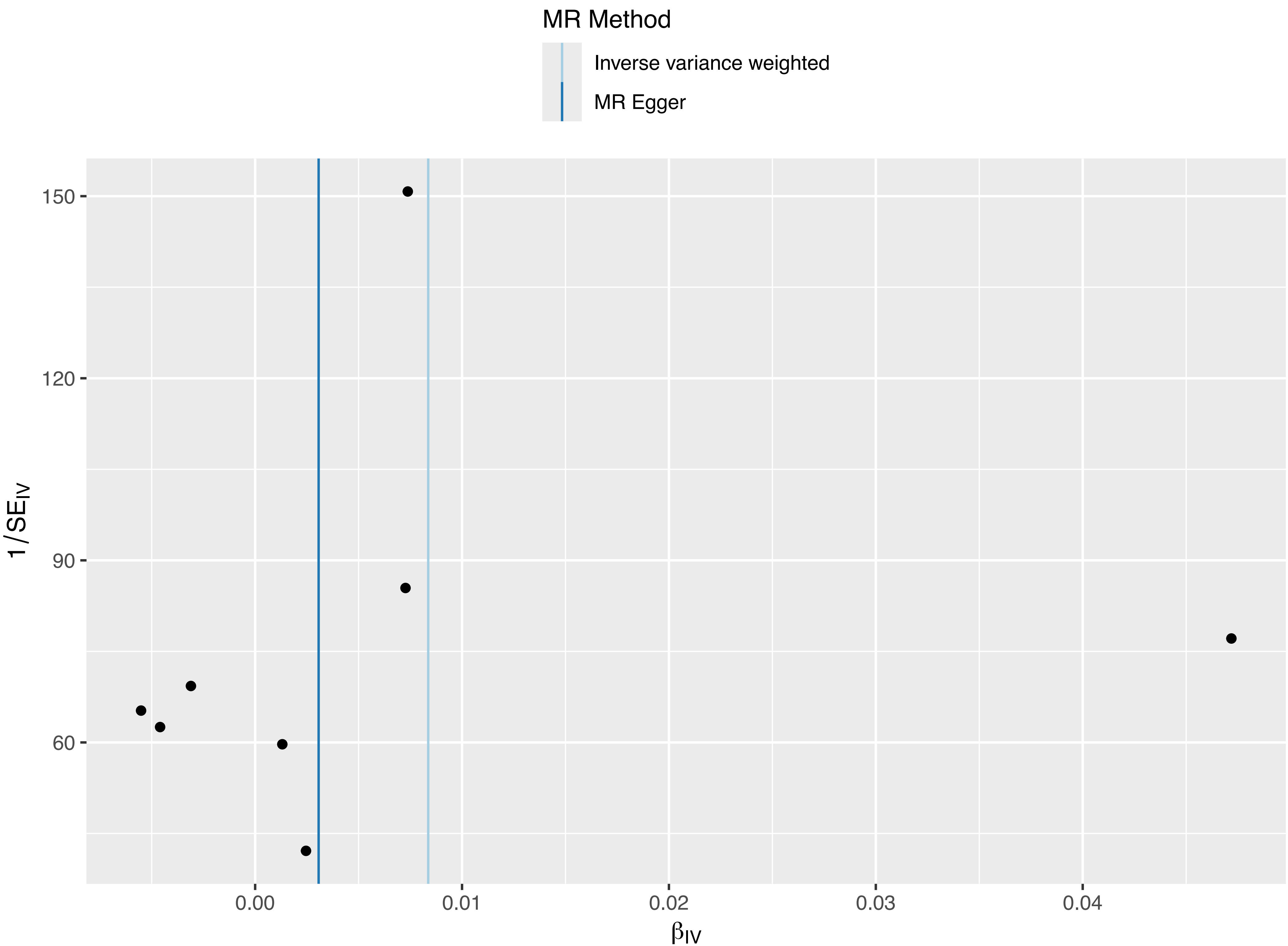
MR Method
Inverse variance weighted
MR Egger
150
120
90
60
0.00
0.01
0.02
0.03
0.04
$1/SE_{IV}$
β_{IV}

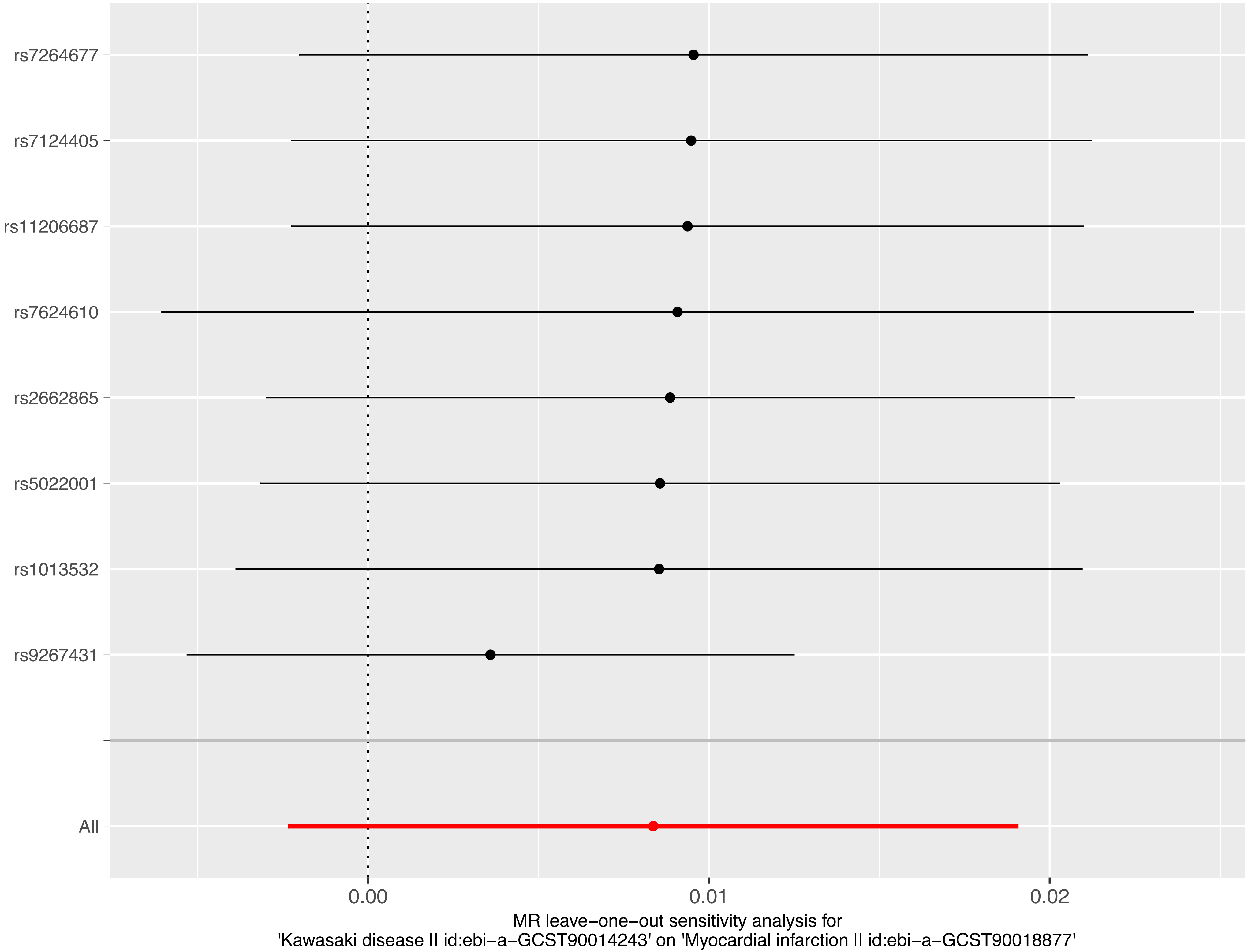
rs7264677
rs7124405
rs11206687
rs7624610
rs2662865
rs5022001
rs1013532
rs9267431
All
0.00
0.01
0.02
MR leave−one−out sensitivity analysis for
'Kawasaki disease || id:ebi−a−GCST90014243' on 'Myocardial infarction || id:ebi−a−GCST90018877'

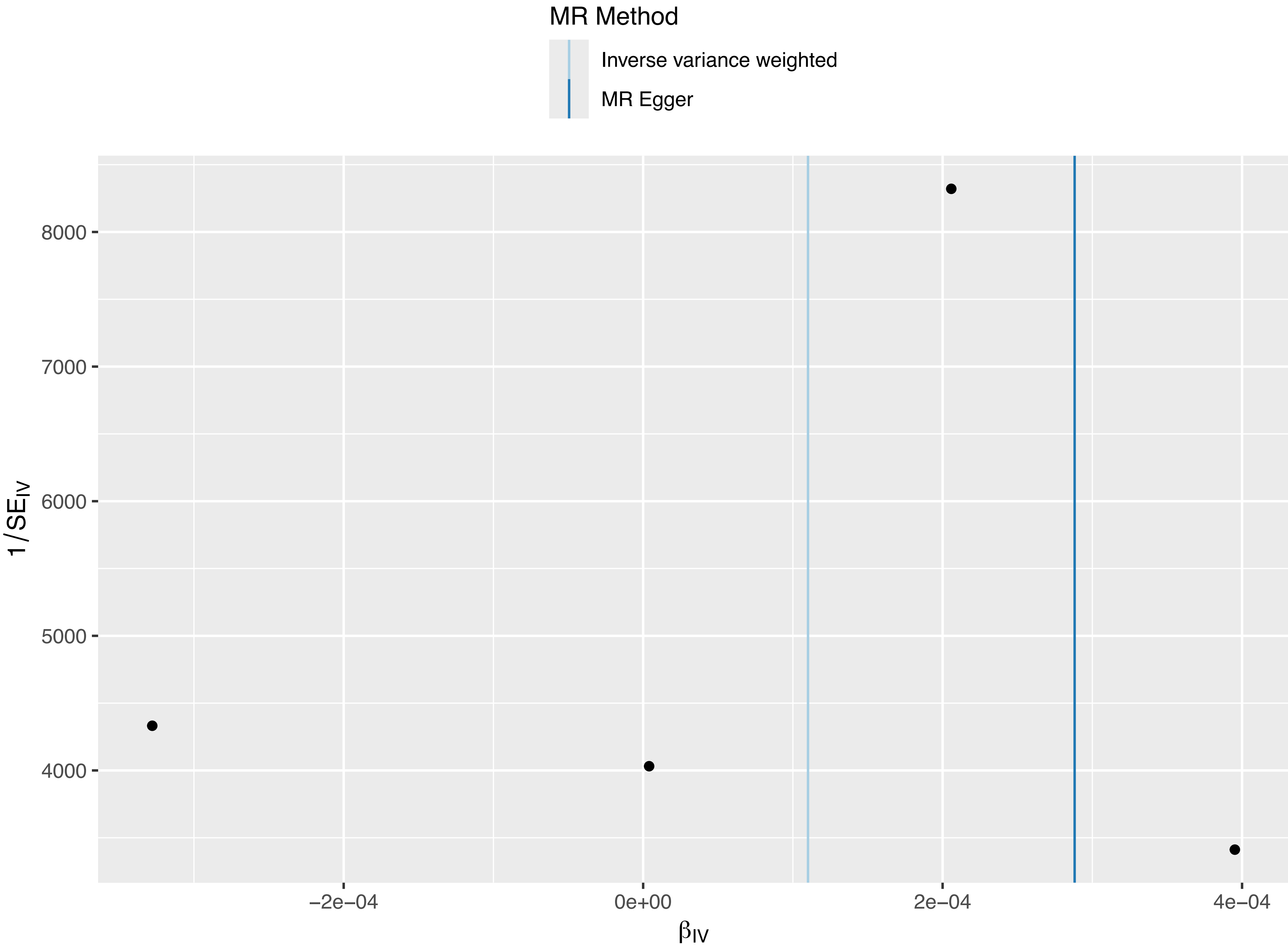

MR Method
Inverse variance weighted
MR Egger
8000
7000
6000
5000
4000
1/SE_IV
−2e−04
0e+00
2e−04
4e−04
β_IV

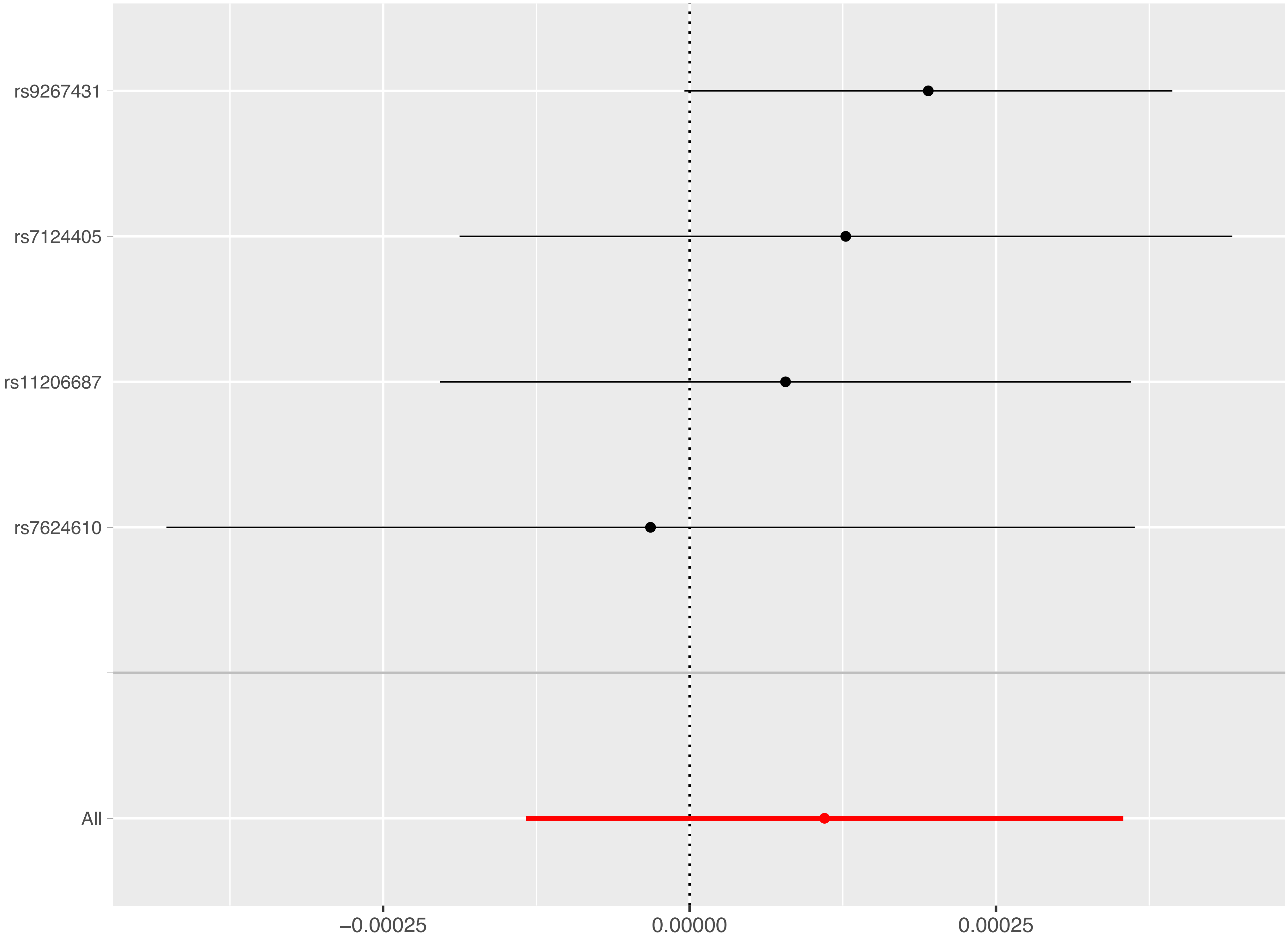

MR leave−one−out sensitivity analysis for
'Kawasaki disease || id:ebi−a−GCST90014243' on 'Non−cancer illness code, self−reported: heart arrhythmia || id:ukb−b−3703'

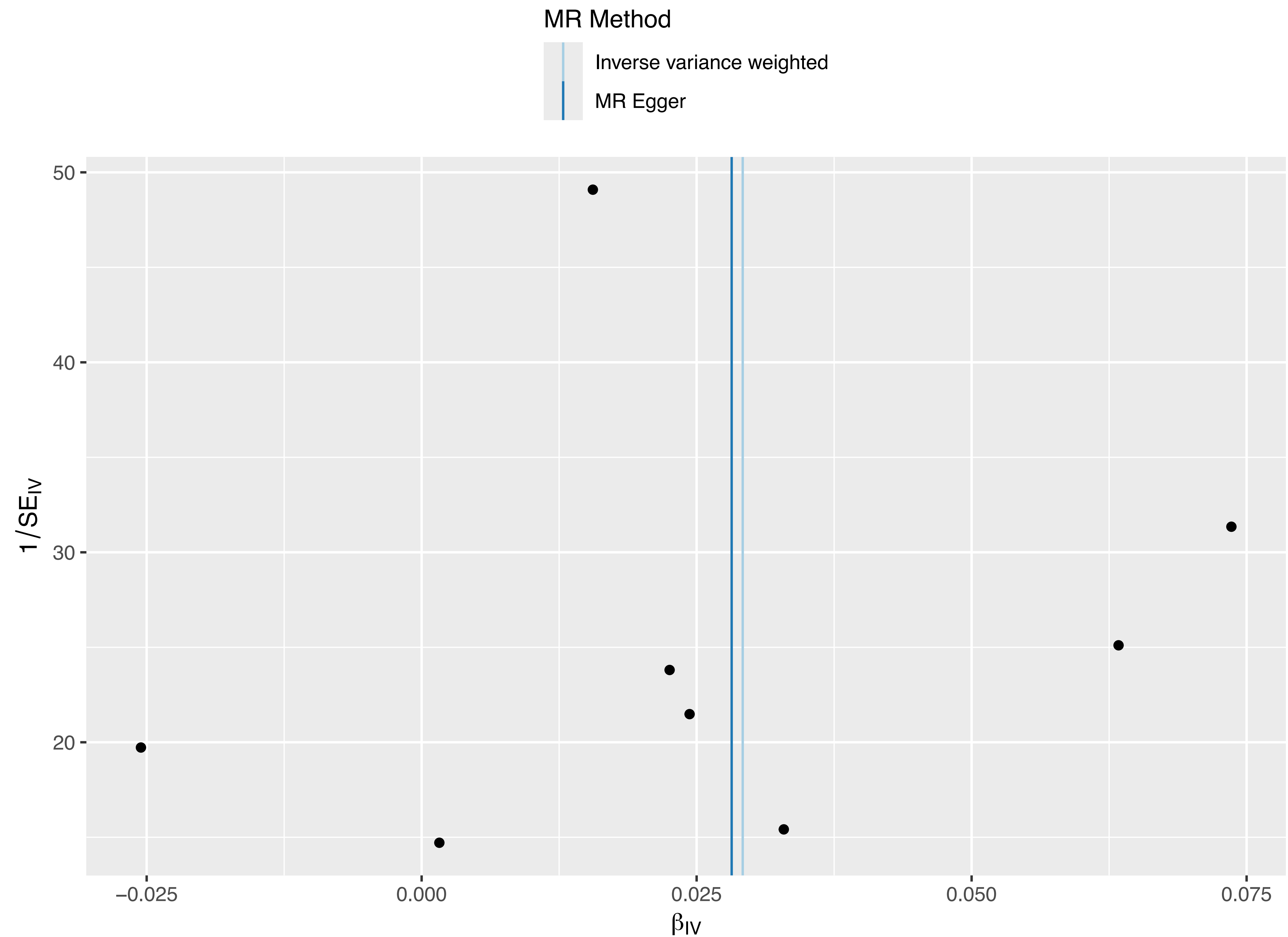
MR Method
Inverse variance weighted
MR Egger
1/SE$_{IV}$
50
40
30
20
−0.025
0.000
0.025
0.050
0.075
β$_{IV}$

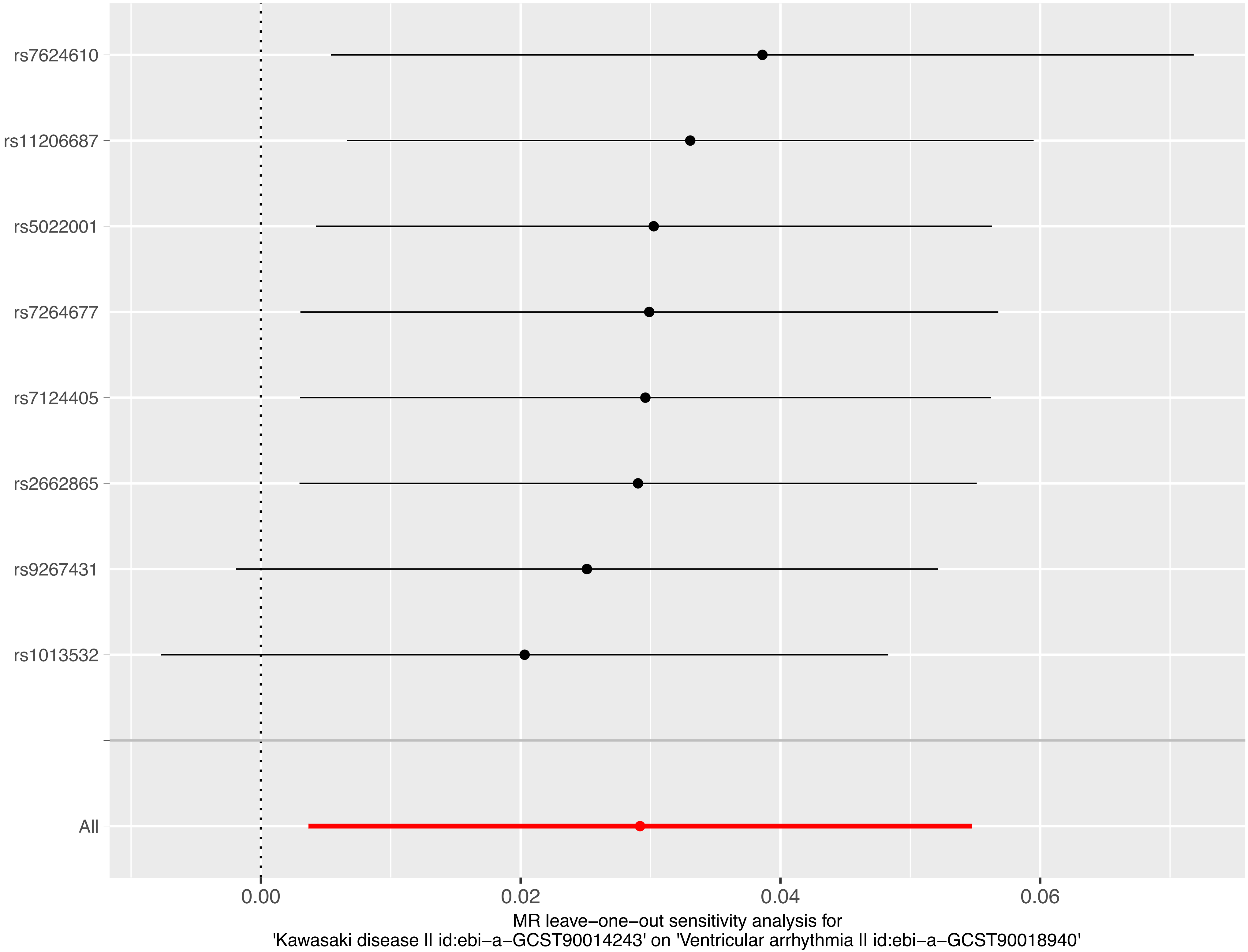

rs7624610
rs11206687
rs5022001
rs7264677
rs7124405
rs2662865
rs9267431
rs1013532
All
0.00
0.02
0.04
0.06
MR leave−one−out sensitivity analysis for
'Kawasaki disease || id:ebi−a−GCST90014243' on 'Ventricular arrhythmia || id:ebi−a−GCST90018940'

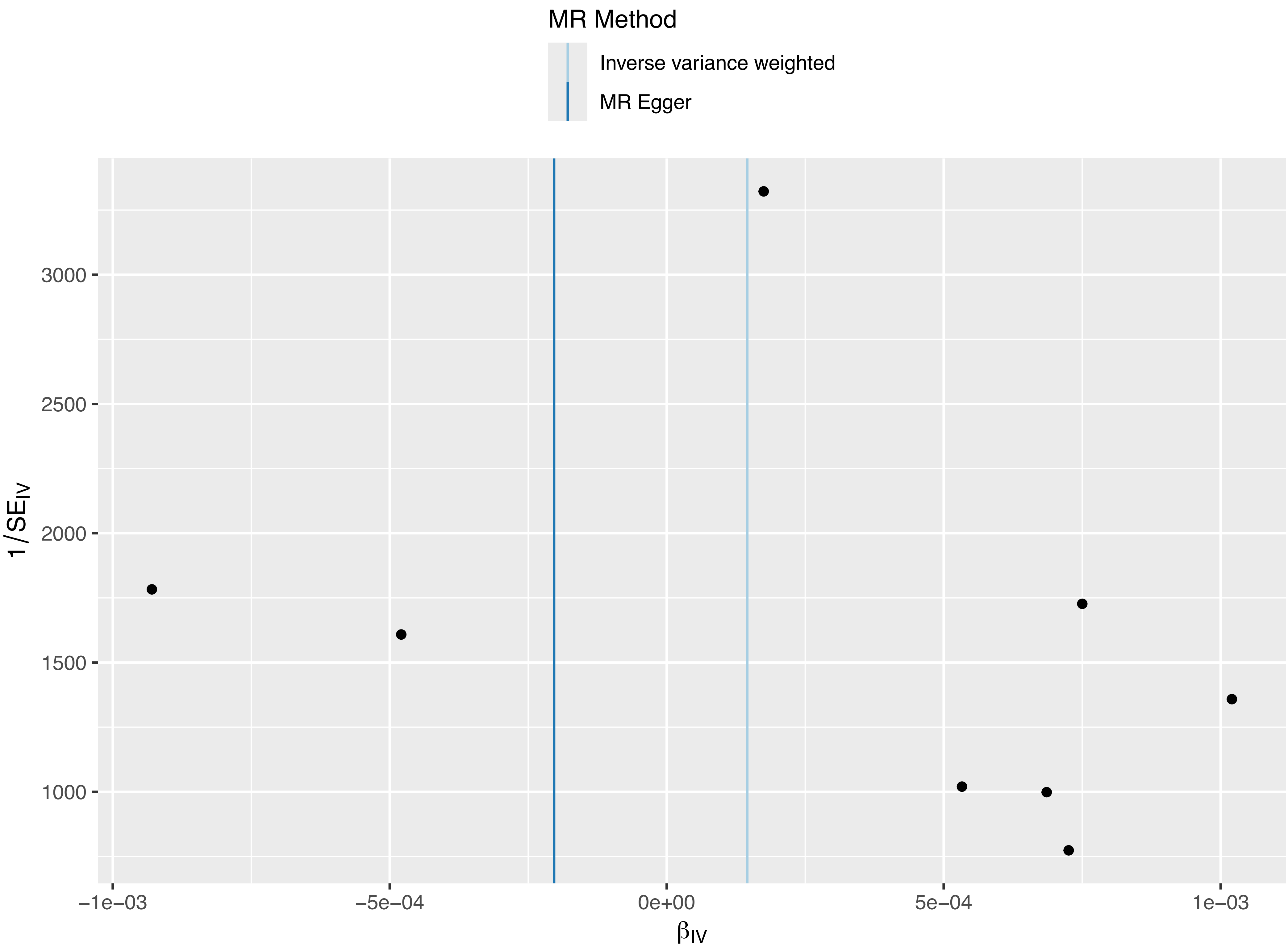

MR Method
Inverse variance weighted
MR Egger
1/SE_IV
3000
2500
2000
1500
1000
−1e−03
−5e−04
0e+00
5e−04
1e−03
β_IV

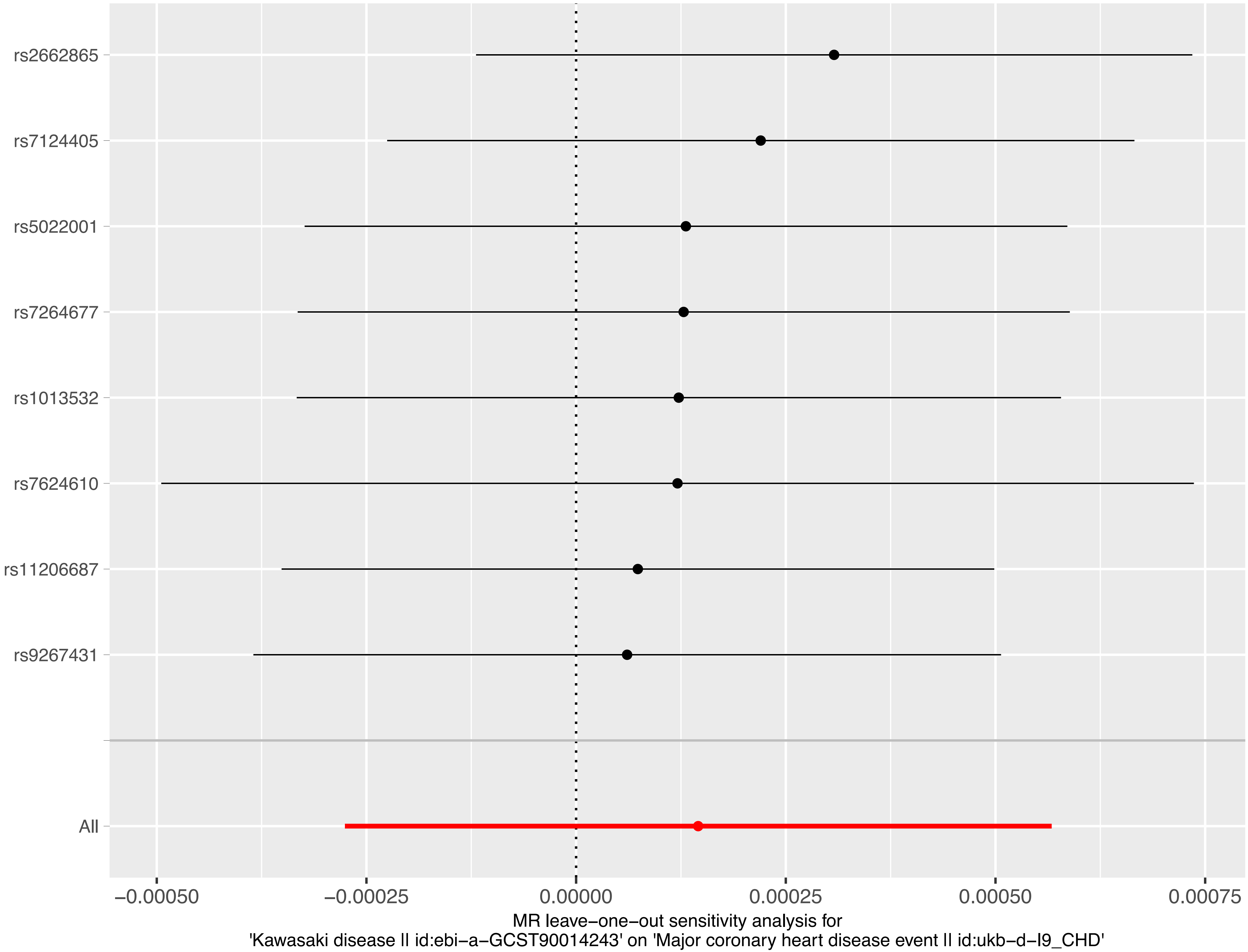
rs2662865
rs7124405
rs5022001
rs7264677
rs1013532
rs7624610
rs11206687
rs9267431
All
−0.00050
−0.00025
0.00000
0.00025
0.00050
0.00075
MR leave−one−out sensitivity analysis for
'Kawasaki disease || id:ebi−a−GCST90014243' on 'Major coronary heart disease event || id:ukb−d−I9_CHD'

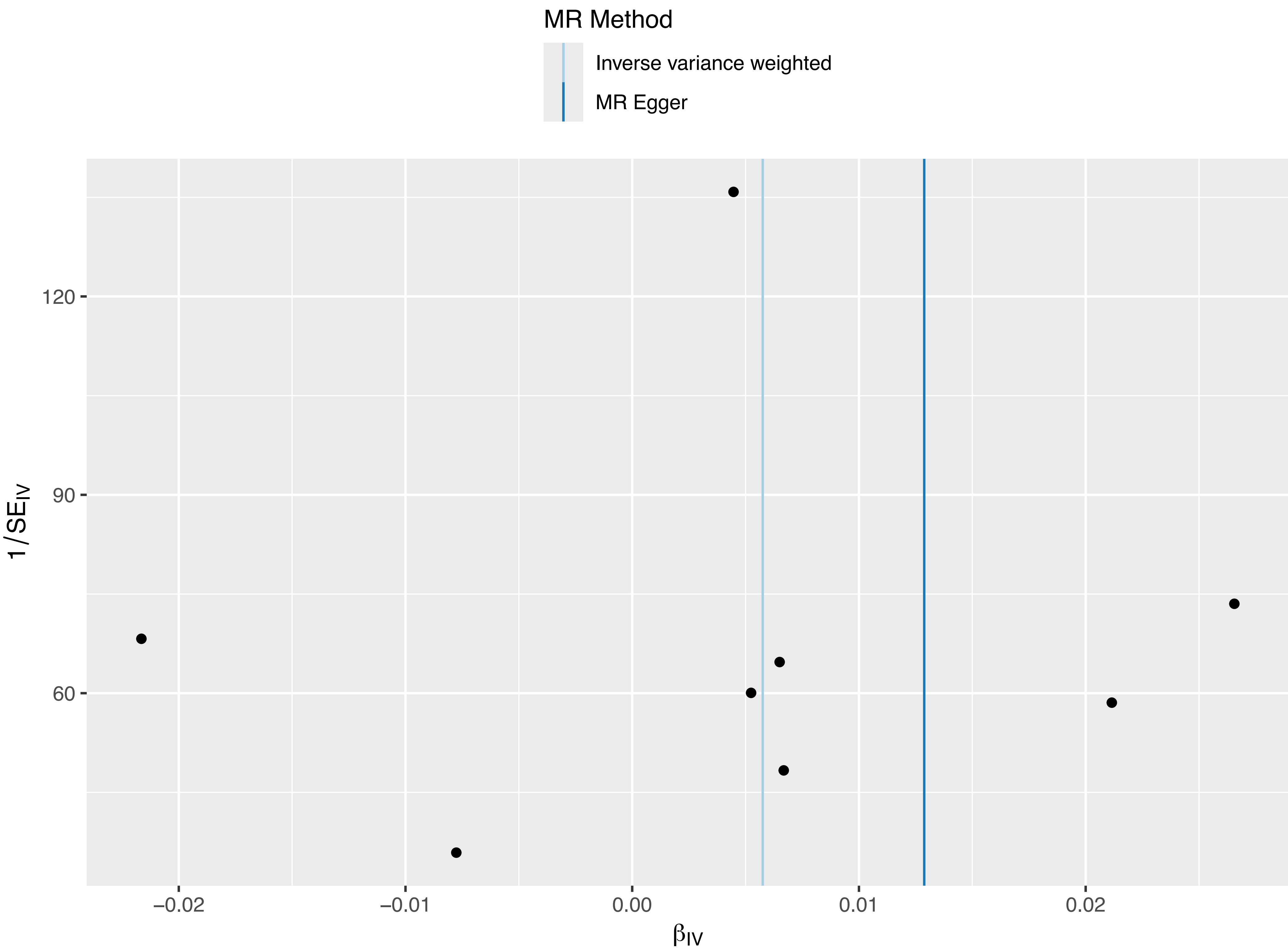

MR Method
Inverse variance weighted
MR Egger
120
90
60
$1/SE_{IV}$
−0.02
−0.01
0.00
0.01
0.02
β_{IV}

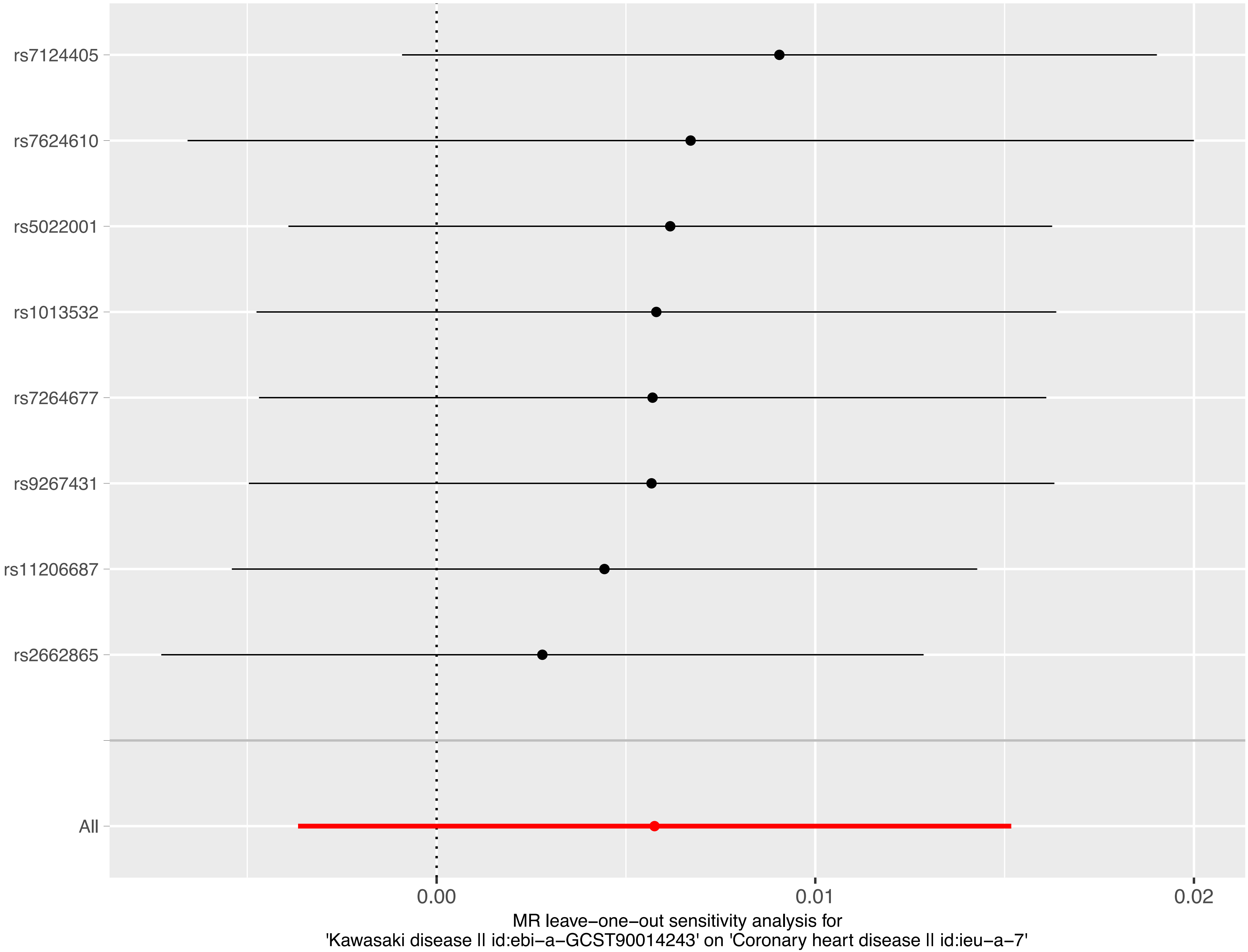
rs7124405
rs7624610
rs5022001
rs1013532
rs7264677
rs9267431
rs11206687
rs2662865
All
0.00
0.01
0.02
MR leave−one−out sensitivity analysis for
'Kawasaki disease || id:ebi−a−GCST90014243' on 'Coronary heart disease || id:ieu−a−7'

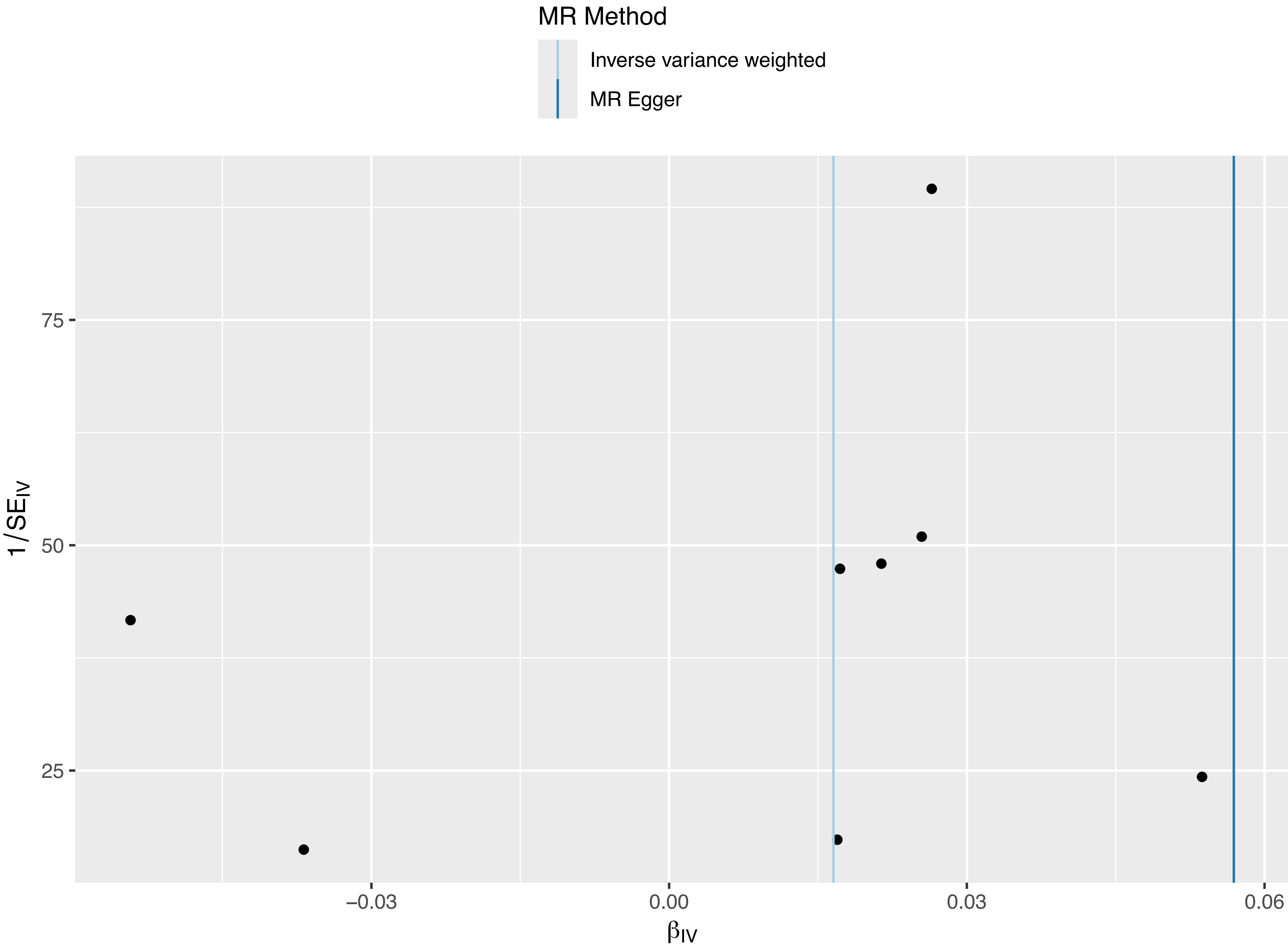
MR Method
Inverse variance weighted
MR Egger
75
50
25
1/SE_IV
−0.03
0.00
0.03
0.06
β_IV

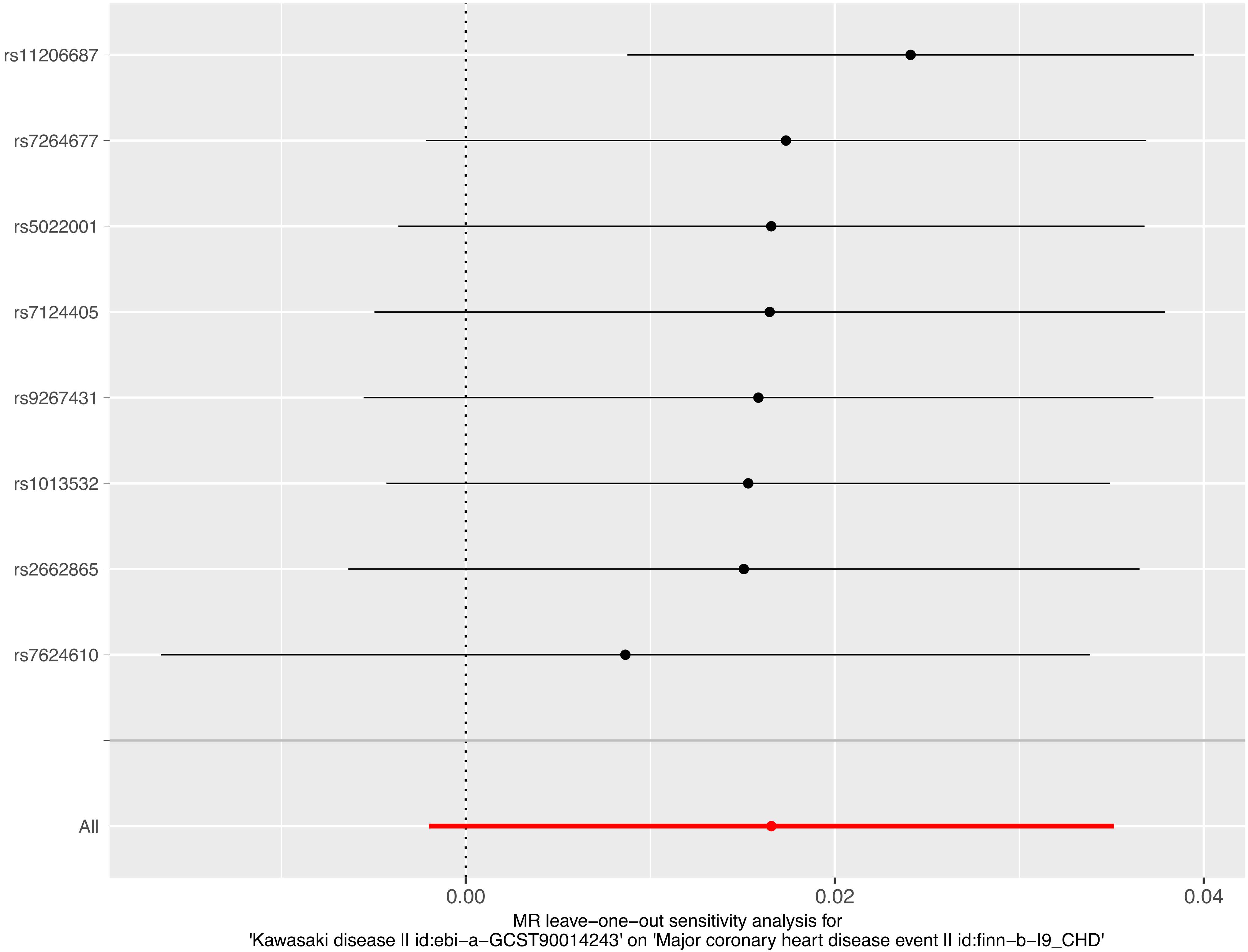
rs11206687
rs7264677
rs5022001
rs7124405
rs9267431
rs1013532
rs2662865
rs7624610
All
0.00
0.02
0.04
MR leave−one−out sensitivity analysis for
'Kawasaki disease || id:ebi−a−GCST90014243' on 'Major coronary heart disease event || id:finn−b−I9_CHD'

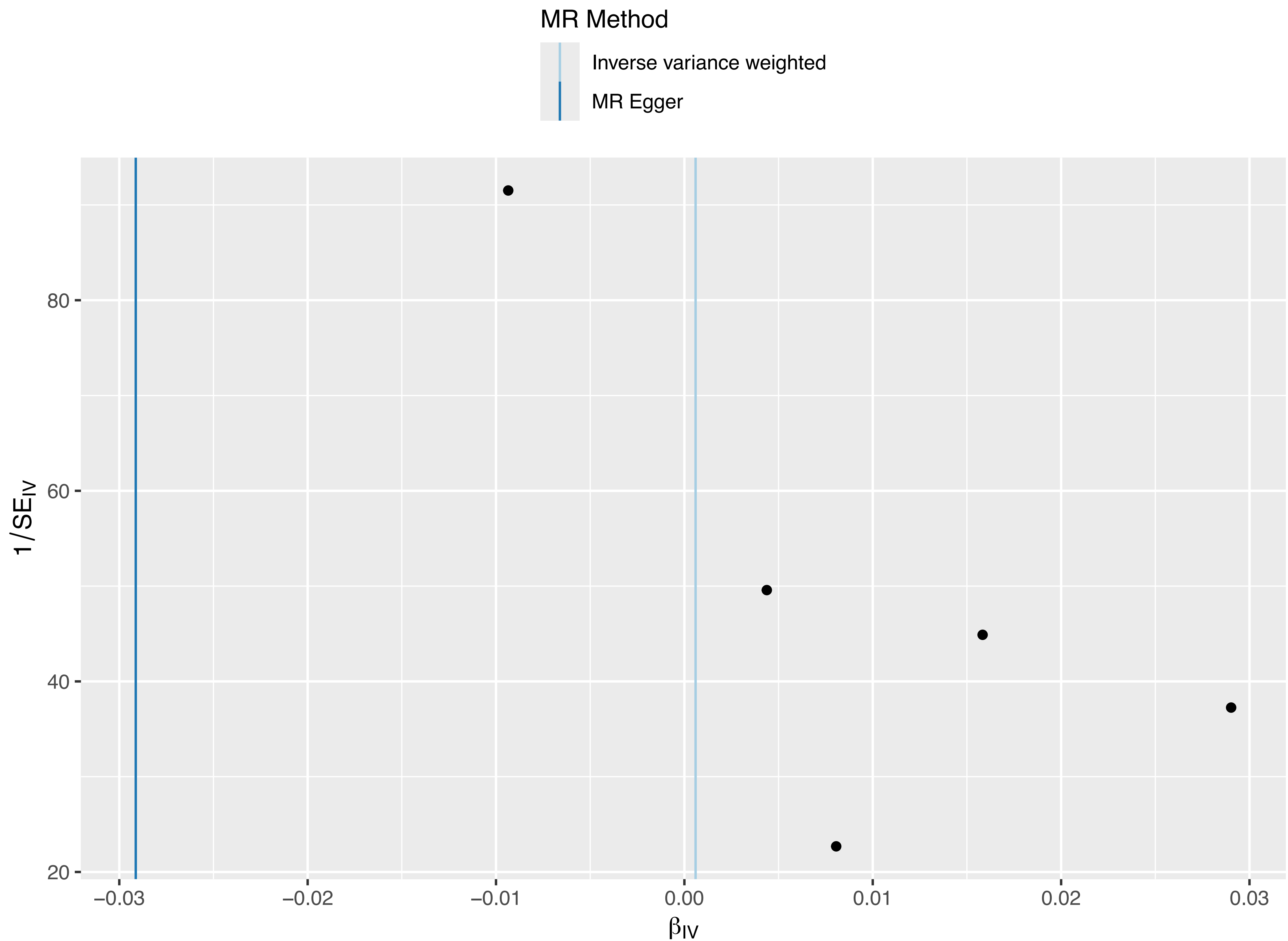

MR Method
Inverse variance weighted
MR Egger
80
60
40
20
1/SE$_{IV}$
−0.03
−0.02
−0.01
0.00
0.01
0.02
0.03
β_{IV}

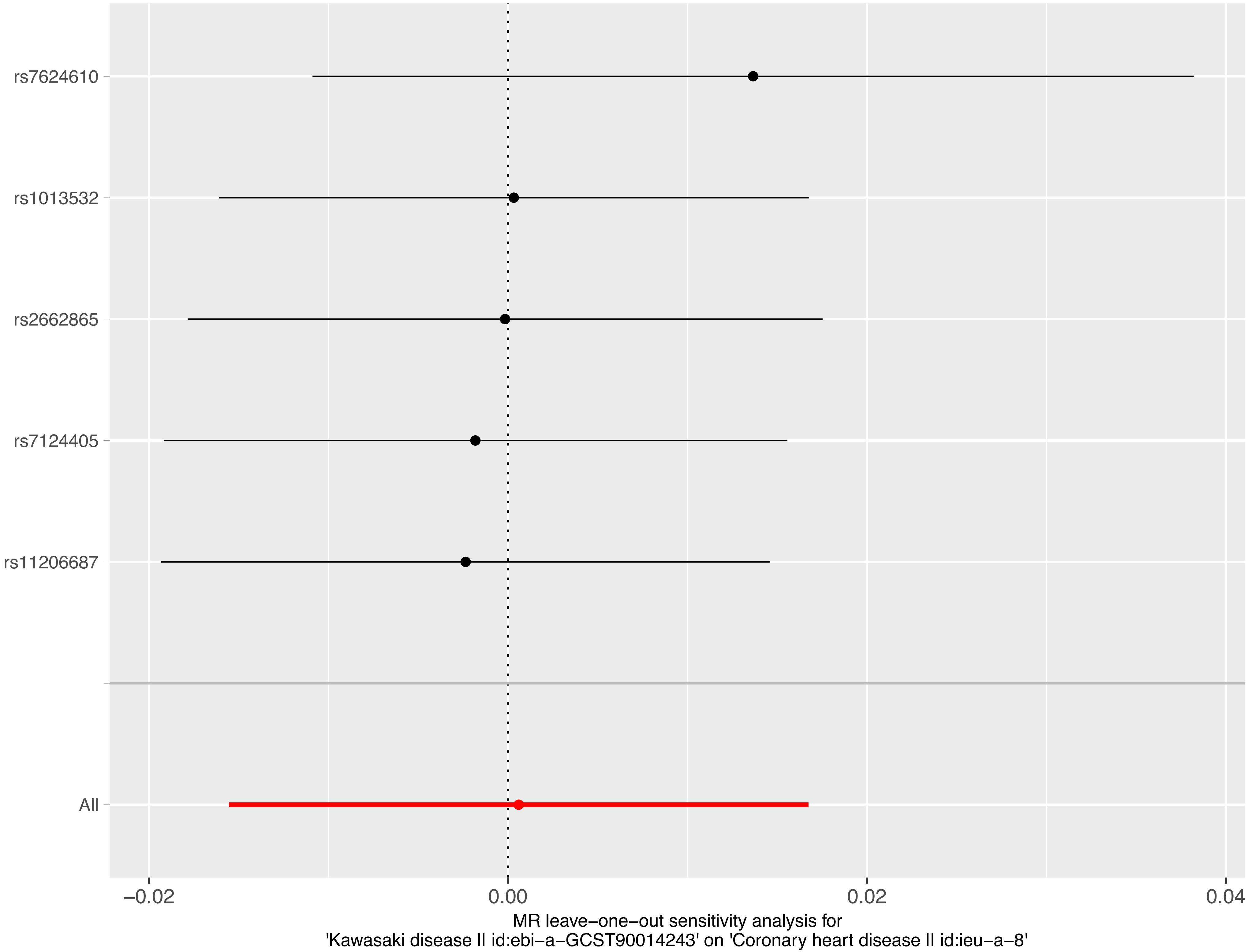
rs7624610
rs1013532
rs2662865
rs7124405
rs11206687
All
−0.02
0.00
0.02
0.04
MR leave−one−out sensitivity analysis for
'Kawasaki disease || id:ebi−a−GCST90014243' on 'Coronary heart disease || id:ieu−a−8'

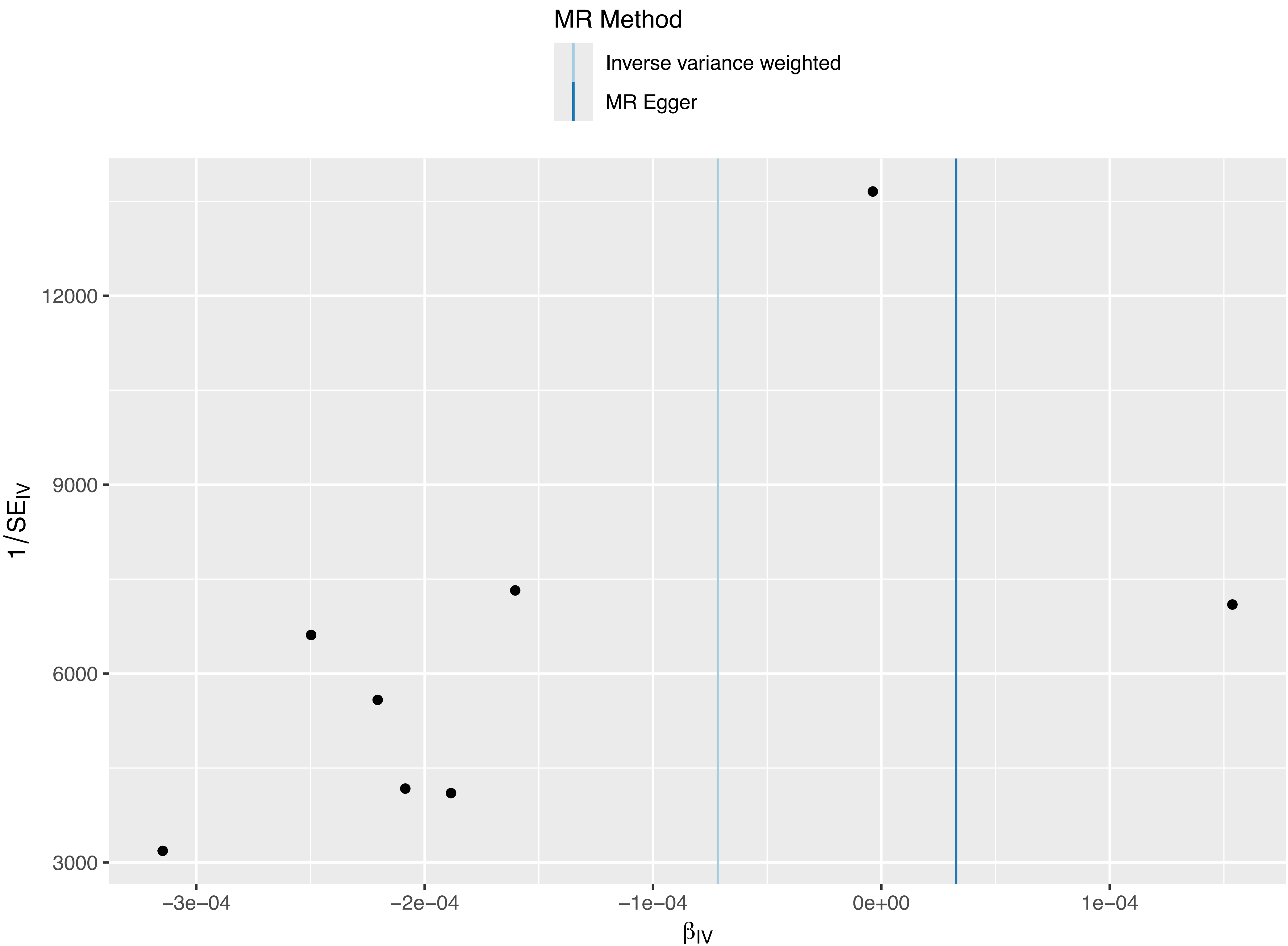

MR Method
Inverse variance weighted
MR Egger
12000
9000
6000
3000
$1/SE_{IV}$
−3e−04
−2e−04
−1e−04
0e+00
1e−04
β_{IV}

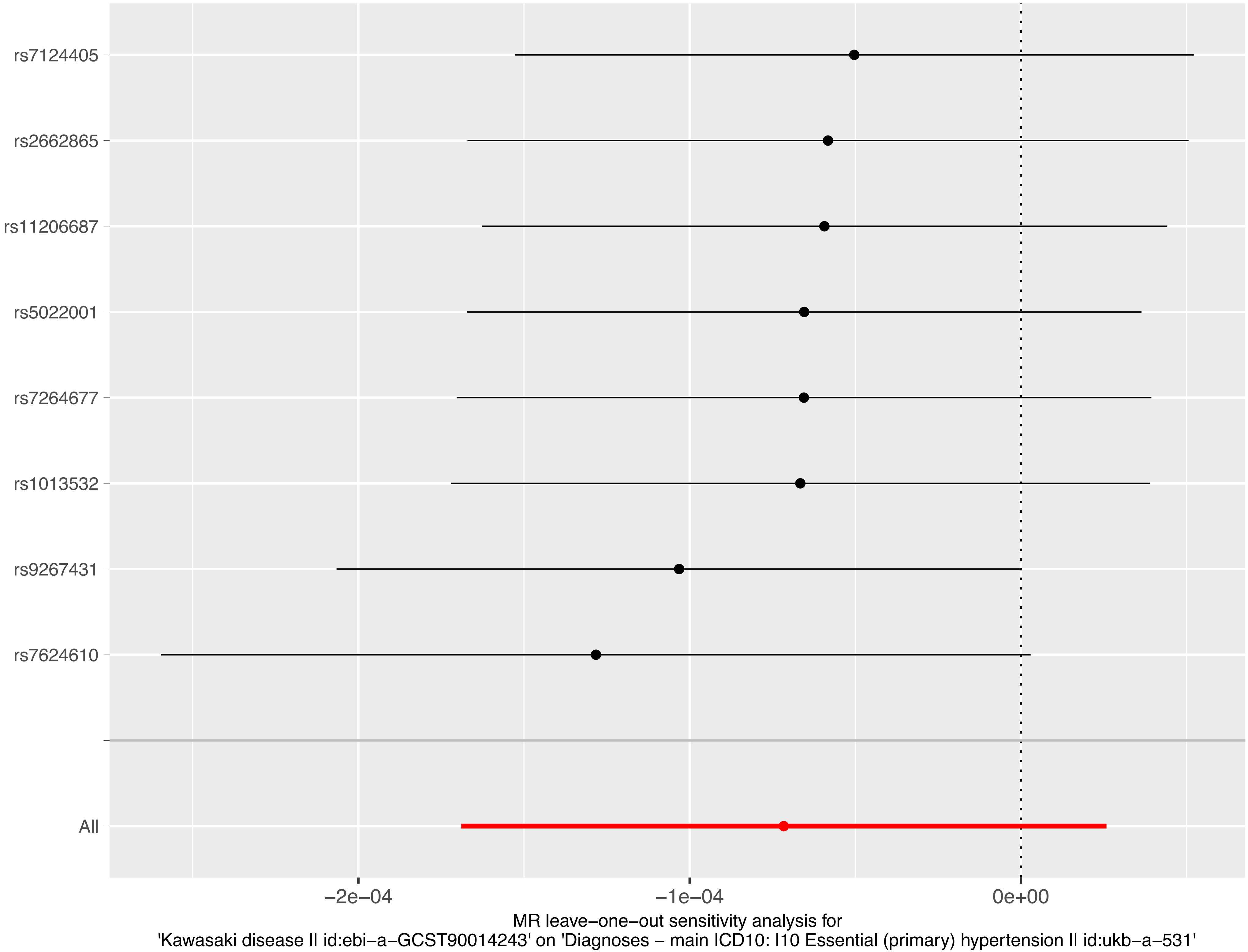
rs7124405
rs2662865
rs11206687
rs5022001
rs7264677
rs1013532
rs9267431
rs7624610
All
−2e−04
−1e−04
0e+00
MR leave−one−out sensitivity analysis for
'Kawasaki disease || id:ebi−a−GCST90014243' on 'Diagnoses − main ICD10: I10 Essential (primary) hypertension || id:ukb−a−531'
