# Supplementary material for: Genetic liability to childhood Kawasaki disease and adult cardiovascular outcomes
Source: Front Cardiovasc Med. 2026 Jul 23;13:1848320. doi: 10.3389/fcvm.2026.1848320 (PMC13441699; doi:10.3389/fcvm.2026.1848320)
Supplement: Supplementary file 5 [file Datasheet5.pdf]

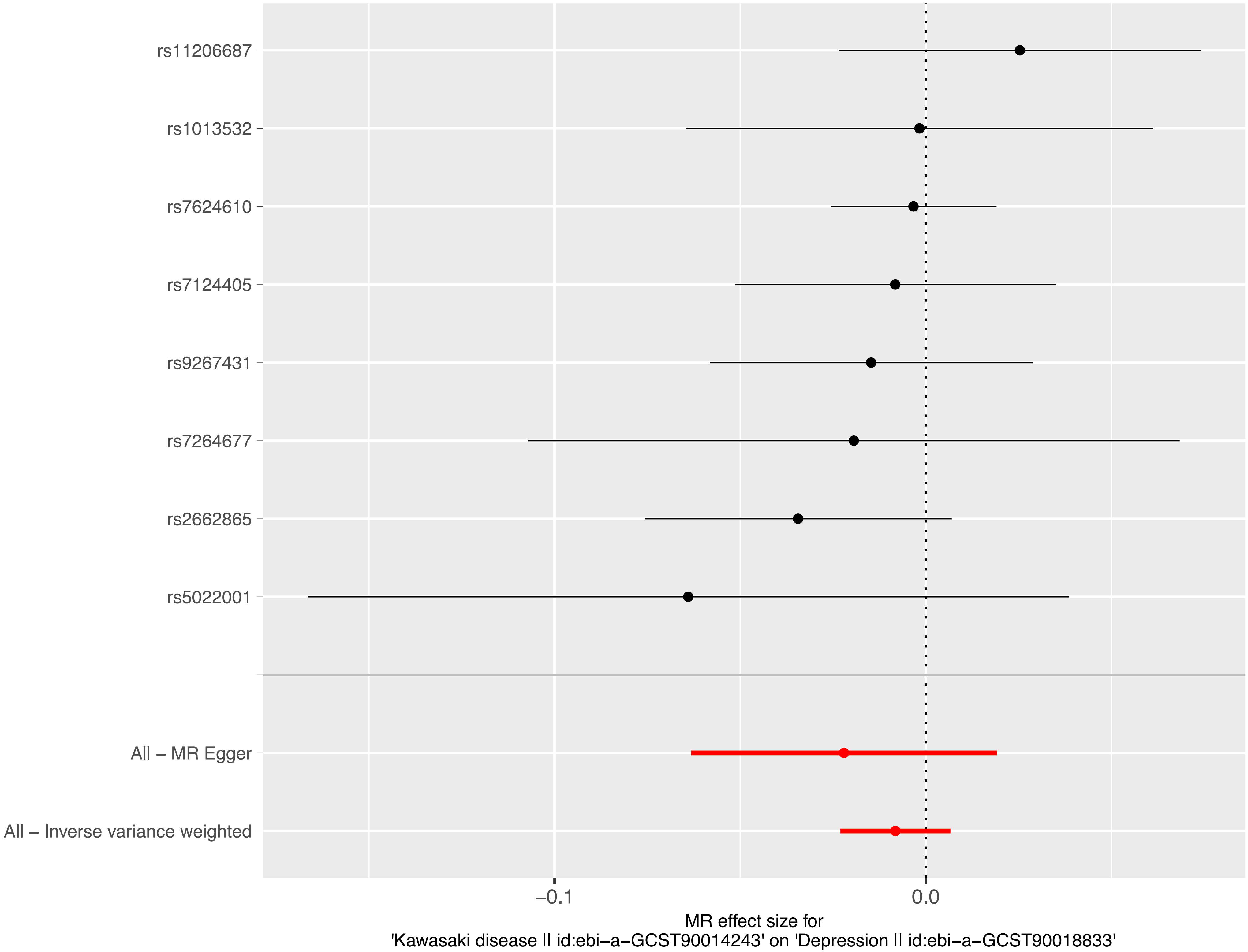
rs11206687
rs1013532
rs7624610
rs7124405
rs9267431
rs7264677
rs2662865
rs5022001
All − MR Egger
All − Inverse variance weighted
−0.1
0.0
MR effect size for
'Kawasaki disease || id:ebi−a−GCST90014243' on 'Depression || id:ebi−a−GCST90018833'

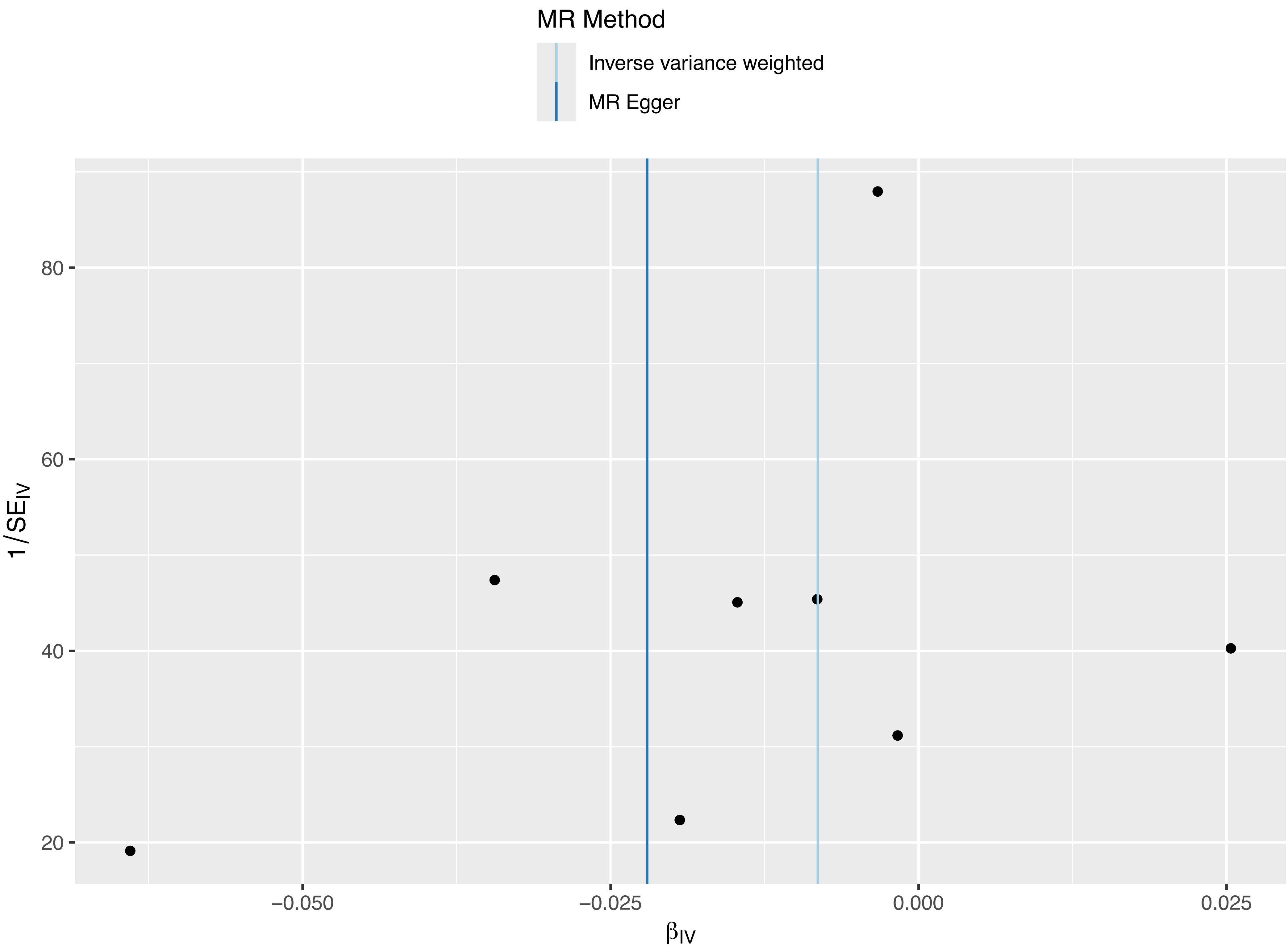

MR Method
Inverse variance weighted
MR Egger
80
60
40
20
1/SE_IV
−0.050
−0.025
0.000
0.025
β_IV

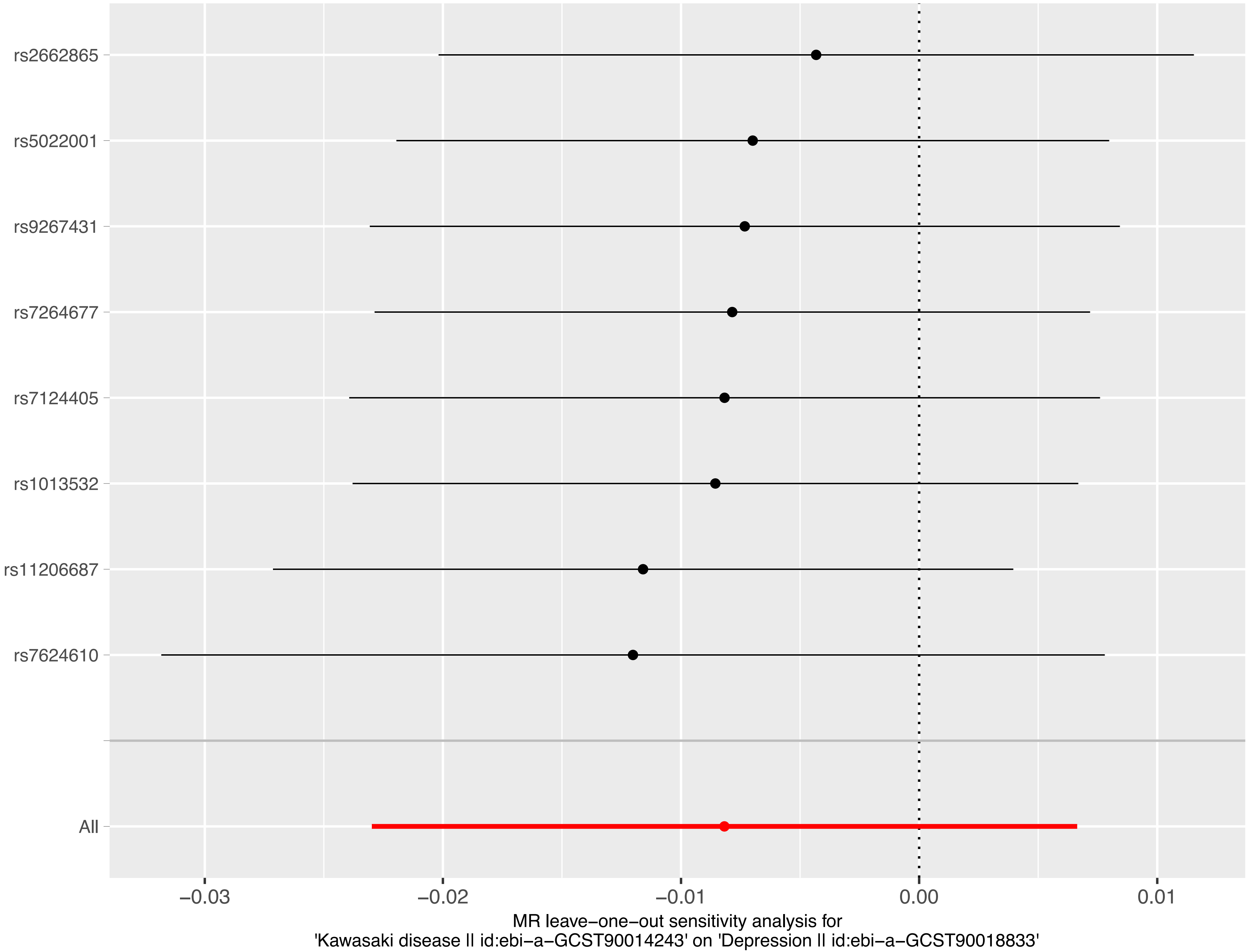
rs2662865
rs5022001
rs9267431
rs7264677
rs7124405
rs1013532
rs11206687
rs7624610
All
−0.03
−0.02
−0.01
0.00
0.01
MR leave−one−out sensitivity analysis for
'Kawasaki disease || id:ebi−a−GCST90014243' on 'Depression || id:ebi−a−GCST90018833'

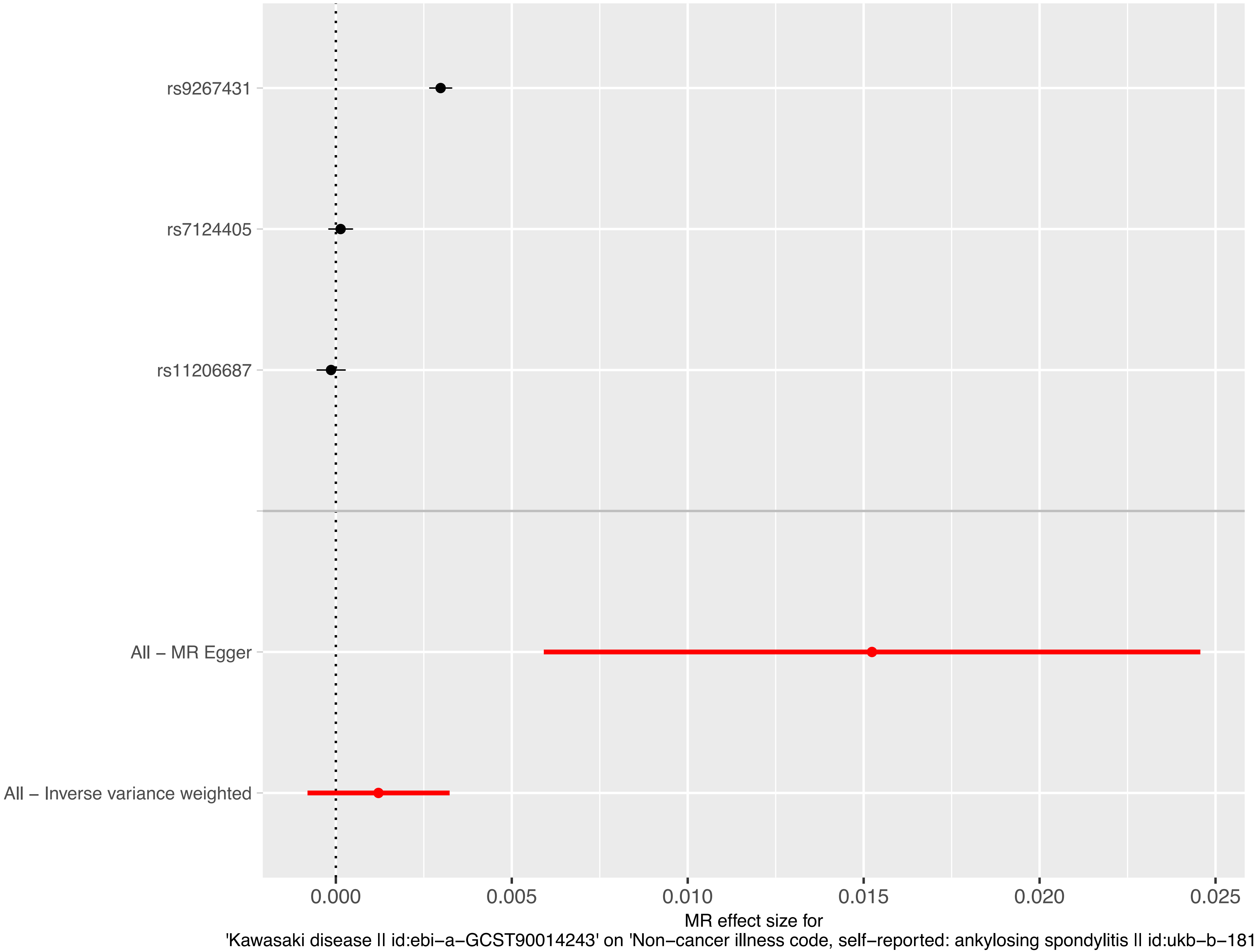

rs9267431
rs7124405
rs11206687
All − MR Egger
All − Inverse variance weighted
0.000
0.005
0.010
0.015
0.020
0.025
MR effect size for
'Kawasaki disease || id:ebi−a−GCST90014243' on 'Non−cancer illness code, self−reported: ankylosing spondylitis || id:ukb−b−181

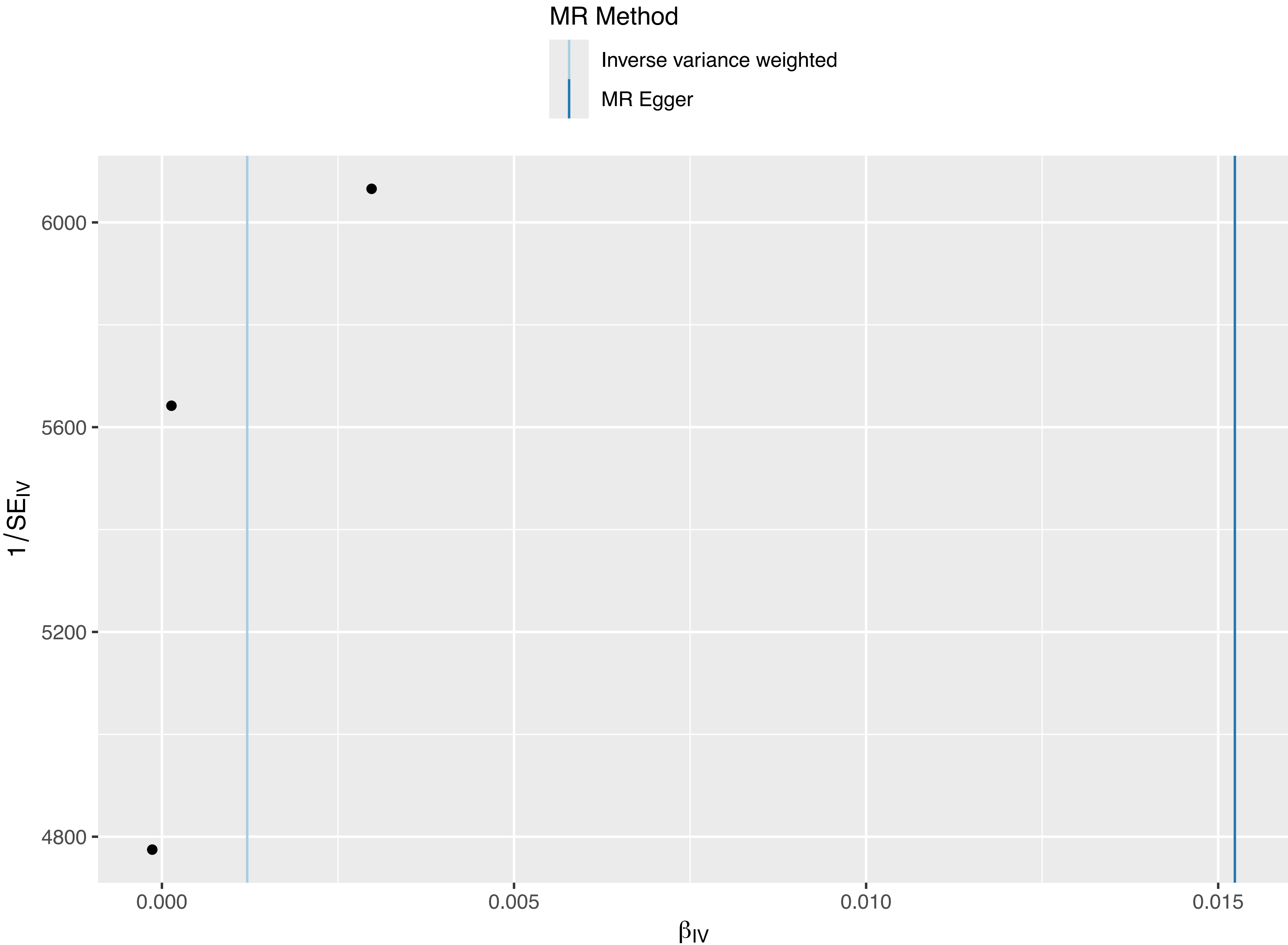
MR Method
Inverse variance weighted
MR Egger
6000
5600
5200
4800
$1/SE_{IV}$
0.000
0.005
0.010
0.015
β_{IV}

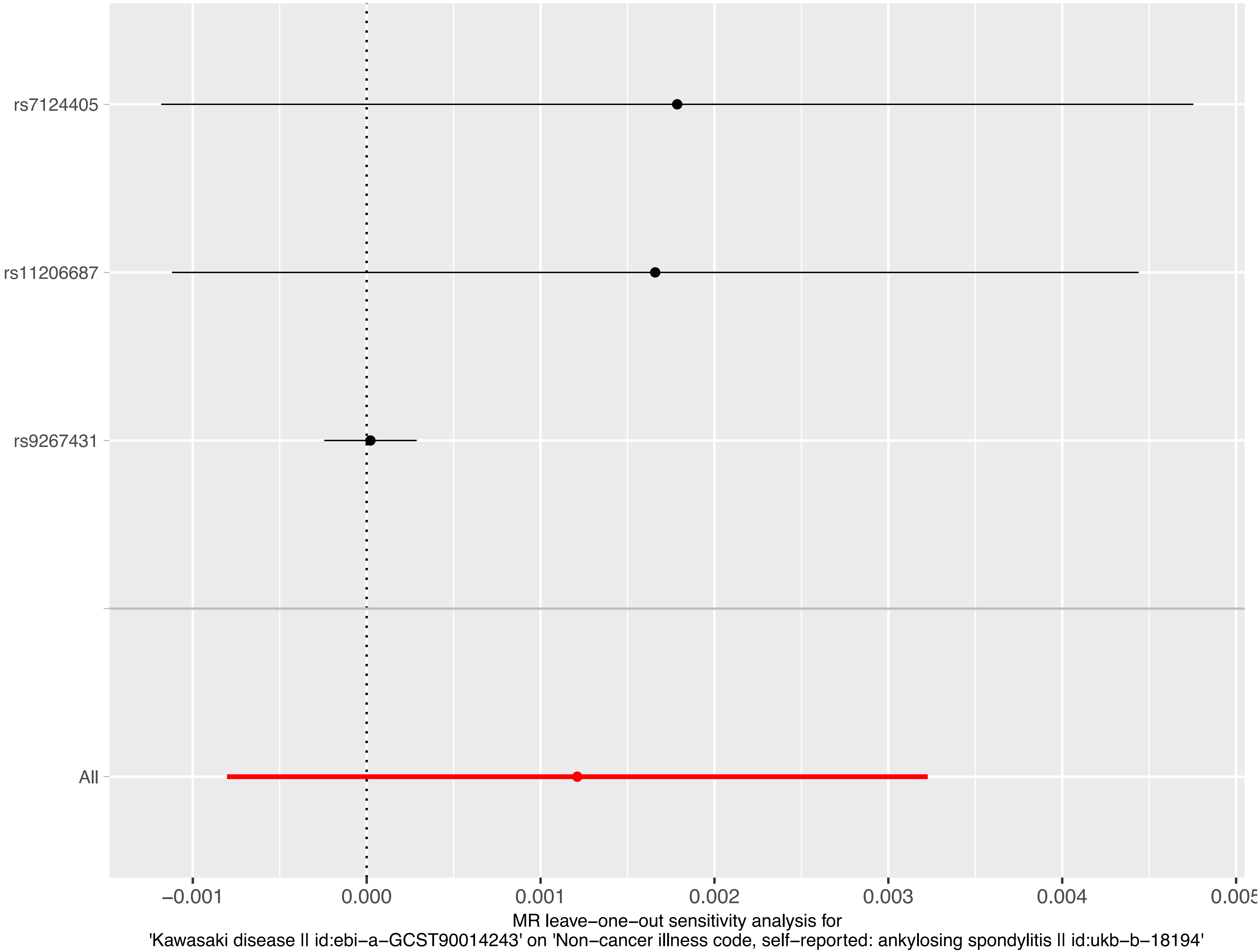
rs7124405
rs11206687
rs9267431
All
−0.001
0.000
0.001
0.002
0.003
0.004
0.005
MR leave−one−out sensitivity analysis for
'Kawasaki disease || id:ebi−a−GCST90014243' on 'Non−cancer illness code, self−reported: ankylosing spondylitis || id:ukb−b−18194'

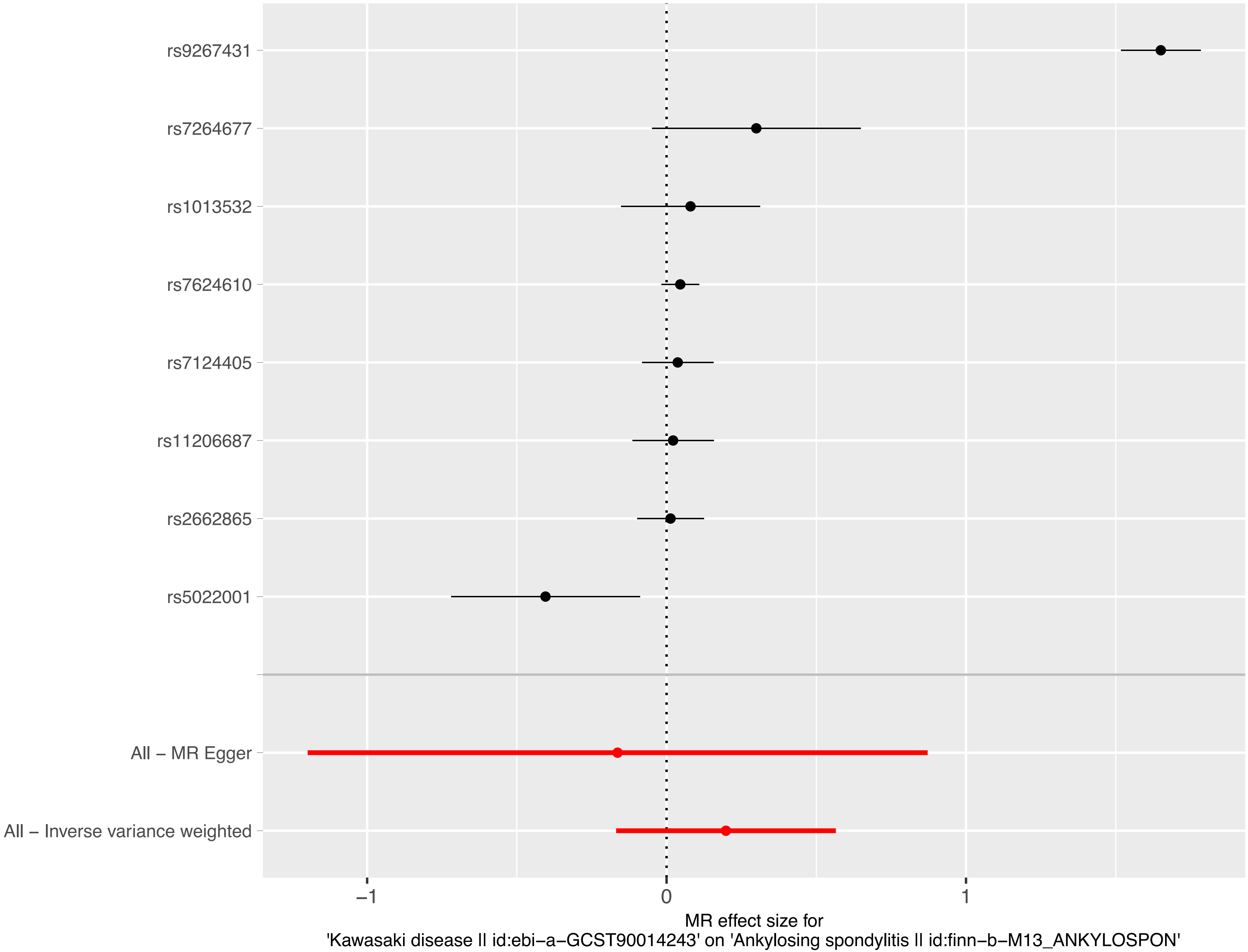

rs9267431
rs7264677
rs1013532
rs7624610
rs7124405
rs11206687
rs2662865
rs5022001
All – MR Egger
All – Inverse variance weighted
−1
0
1
MR effect size for
'Kawasaki disease || id:ebi−a−GCST90014243' on 'Ankylosing spondylitis || id:finn−b−M13_ANKYLOSPON'

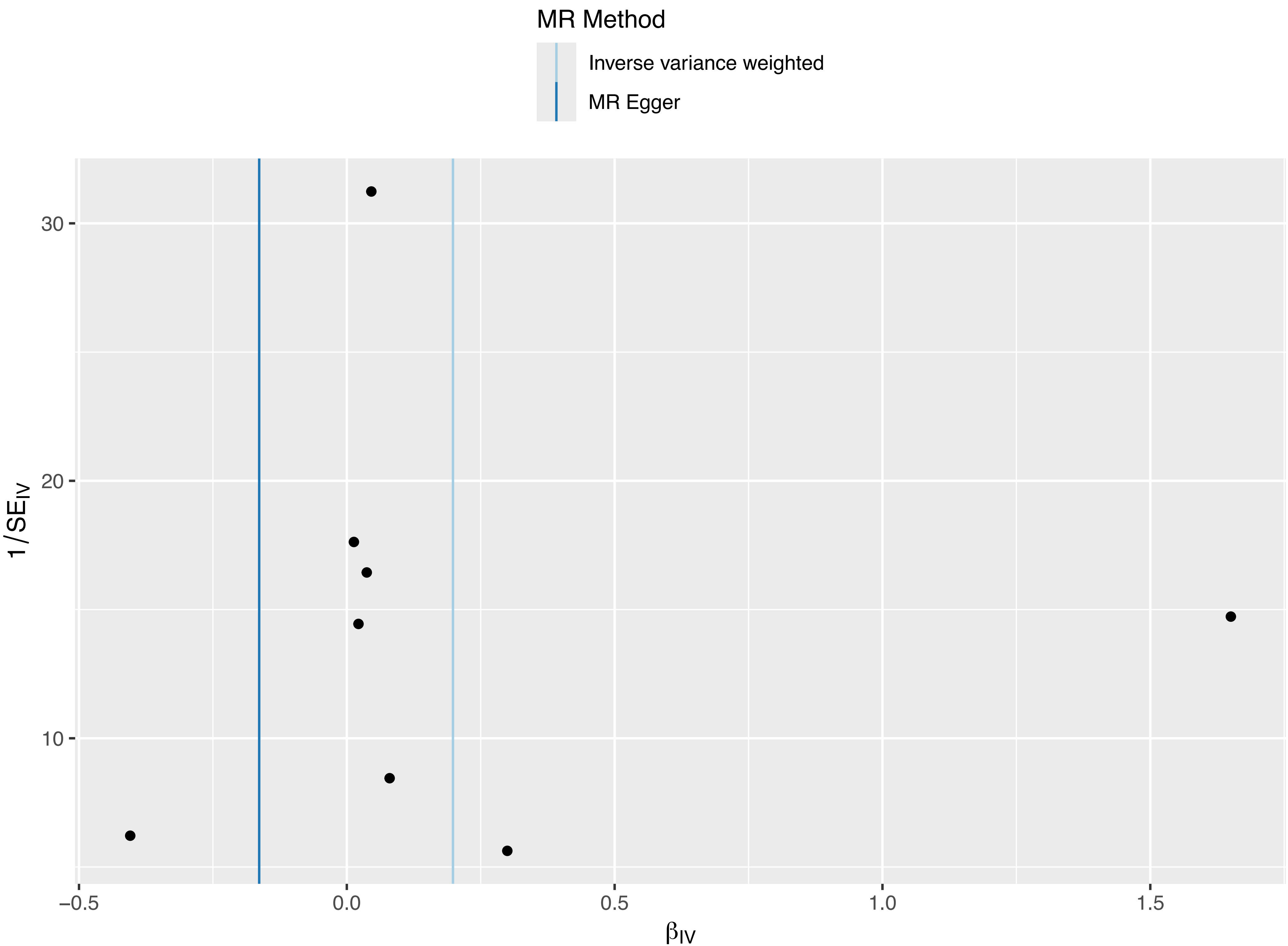
MR Method
Inverse variance weighted
MR Egger
30
20
10
1/SE$_{IV}$
−0.5
0.0
0.5
1.0
1.5
β$_{IV}$

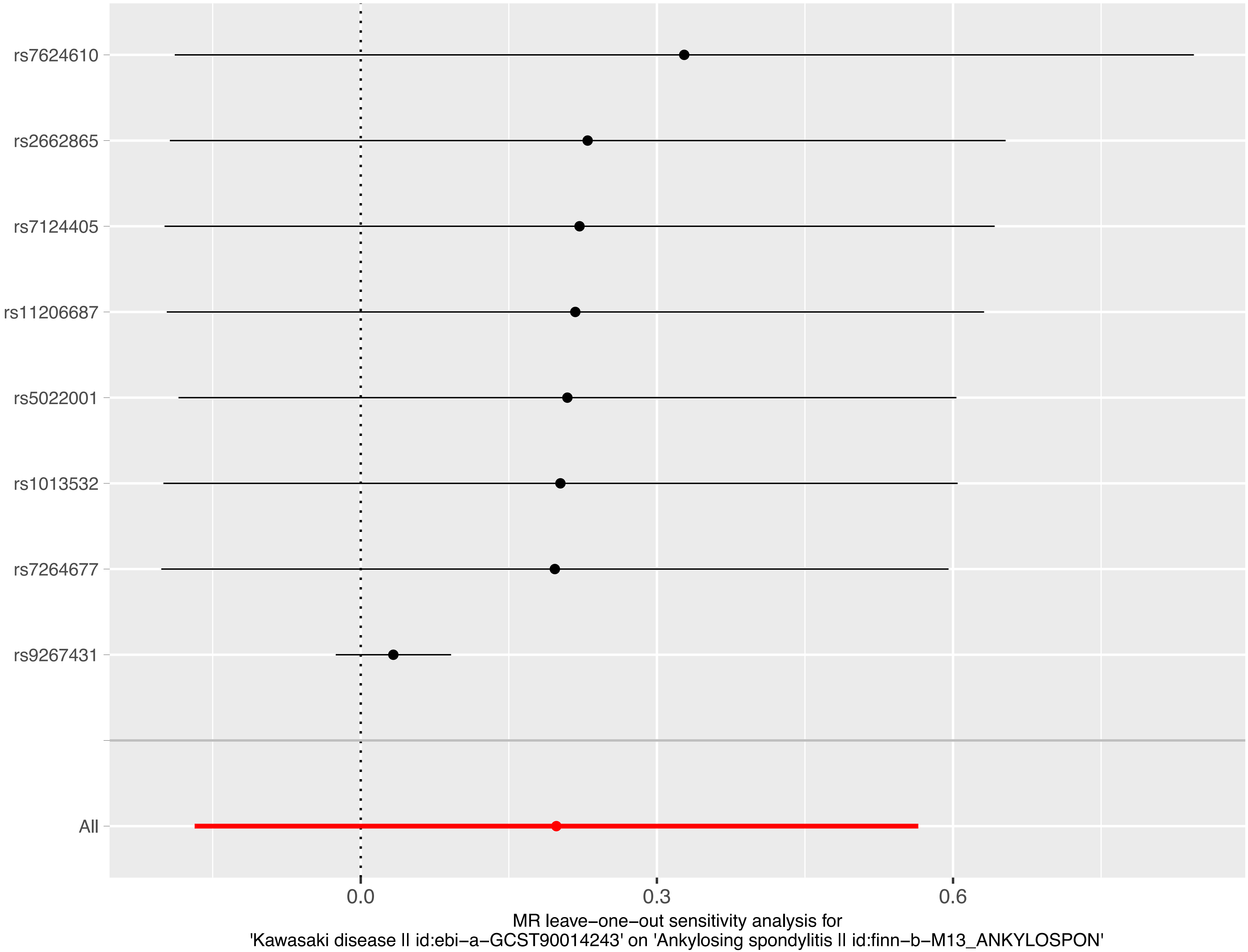
rs7624610
rs2662865
rs7124405
rs11206687
rs5022001
rs1013532
rs7264677
rs9267431
All
0.0
0.3
0.6
MR leave−one−out sensitivity analysis for
'Kawasaki disease || id:ebi−a−GCST90014243' on 'Ankylosing spondylitis || id:finn−b−M13_ANKYLOSPON'

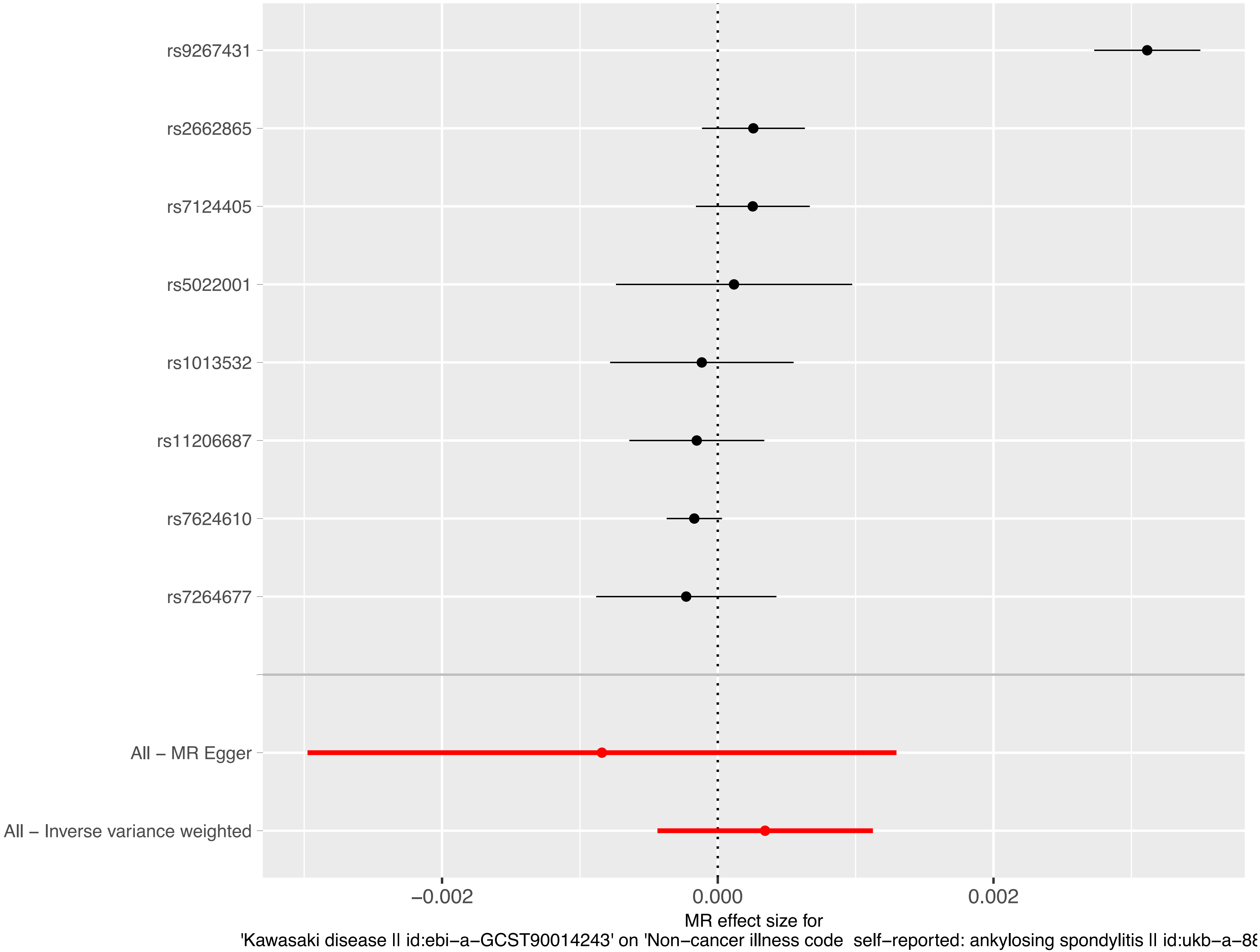
rs9267431
rs2662865
rs7124405
rs5022001
rs1013532
rs11206687
rs7624610
rs7264677
All – MR Egger
All – Inverse variance weighted
−0.002
0.000
0.002
MR effect size for
'Kawasaki disease || id:ebi−a−GCST90014243' on 'Non−cancer illness code self−reported: ankylosing spondylitis || id:ukb−a−8

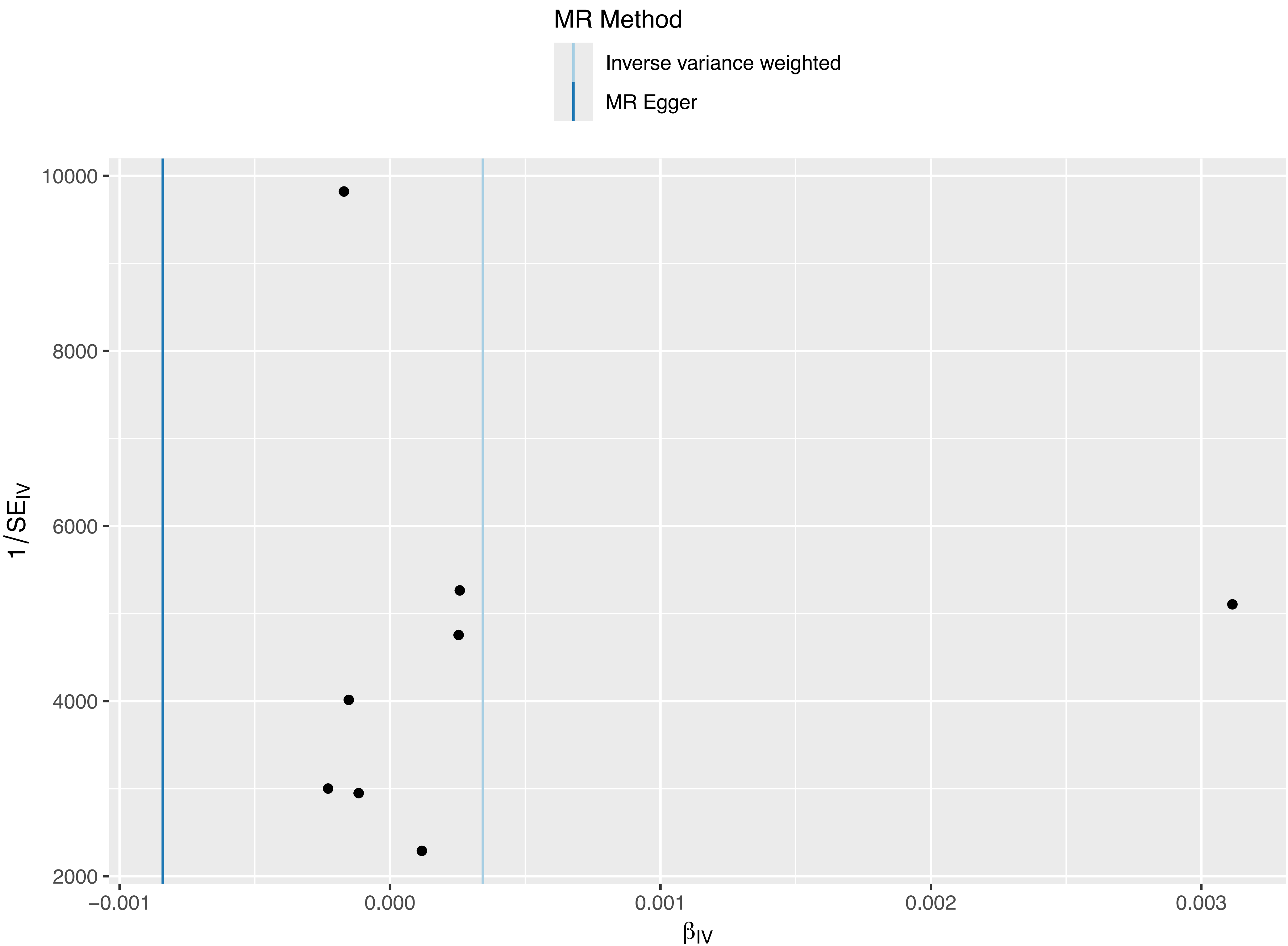
MR Method
Inverse variance weighted
MR Egger
10000
8000
6000
4000
2000
1/SE_IV
−0.001
0.000
0.001
0.002
0.003
β_IV

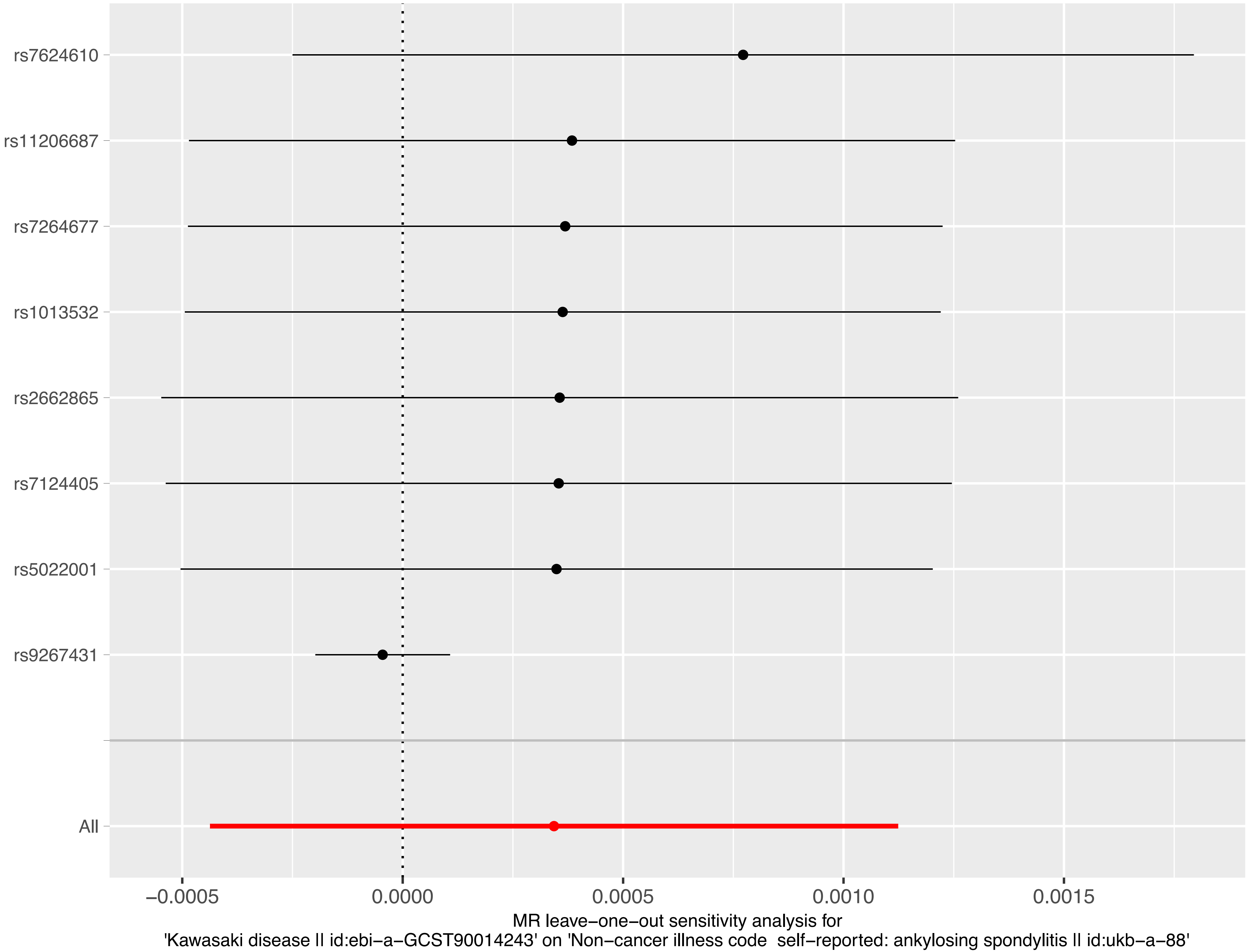
rs7624610
rs11206687
rs7264677
rs1013532
rs2662865
rs7124405
rs5022001
rs9267431
All
−0.0005
0.0000
0.0005
0.0010
0.0015
MR leave−one−out sensitivity analysis for
'Kawasaki disease || id:ebi−a−GCST90014243' on 'Non−cancer illness code self−reported: ankylosing spondylitis || id:ukb−a−88'

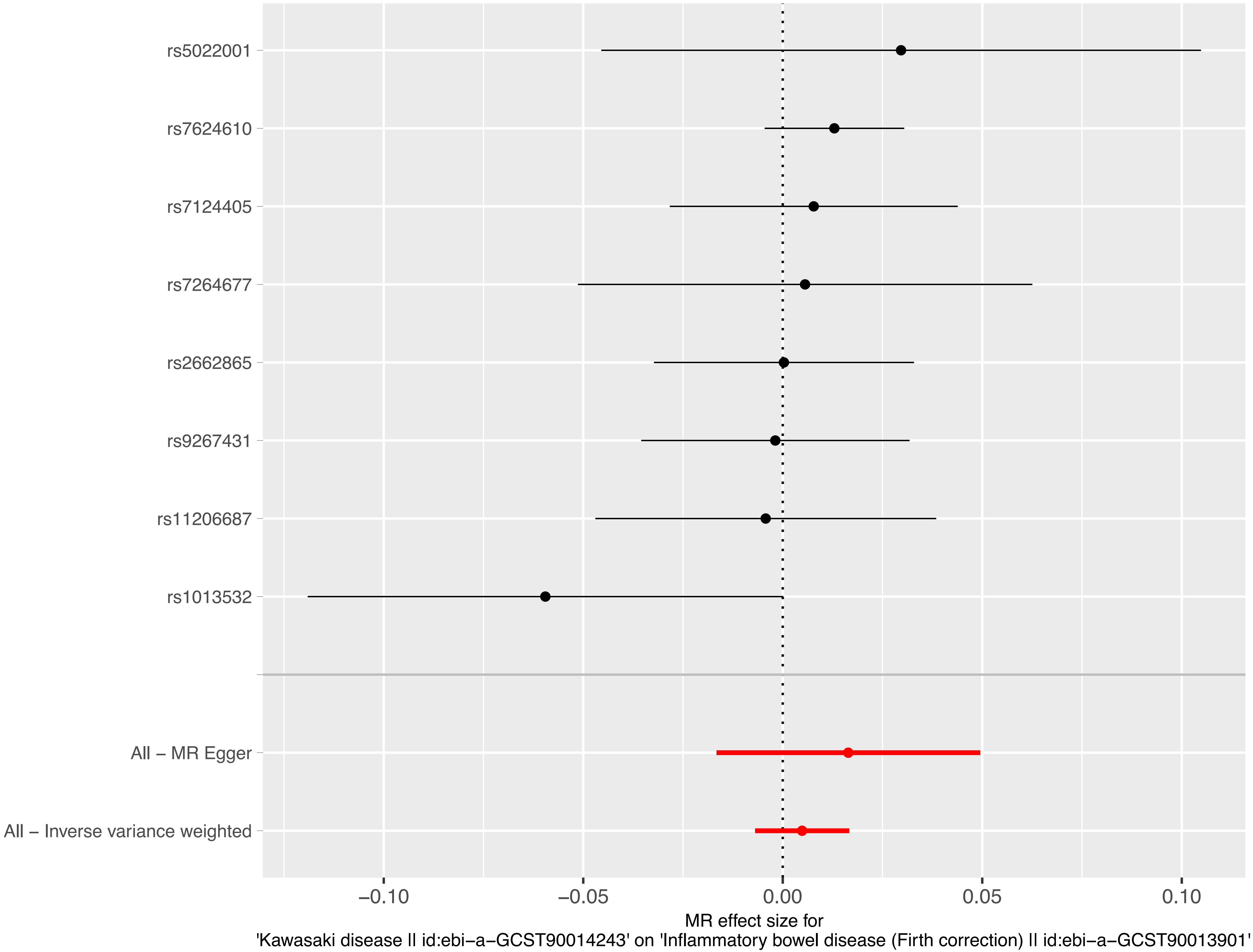
rs5022001
rs7624610
rs7124405
rs7264677
rs2662865
rs9267431
rs11206687
rs1013532
All – MR Egger
All – Inverse variance weighted
−0.10
−0.05
0.00
0.05
0.10
MR effect size for
'Kawasaki disease || id:ebi−a−GCST90014243' on 'Inflammatory bowel disease (Firth correction) || id:ebi−a−GCST90013901'

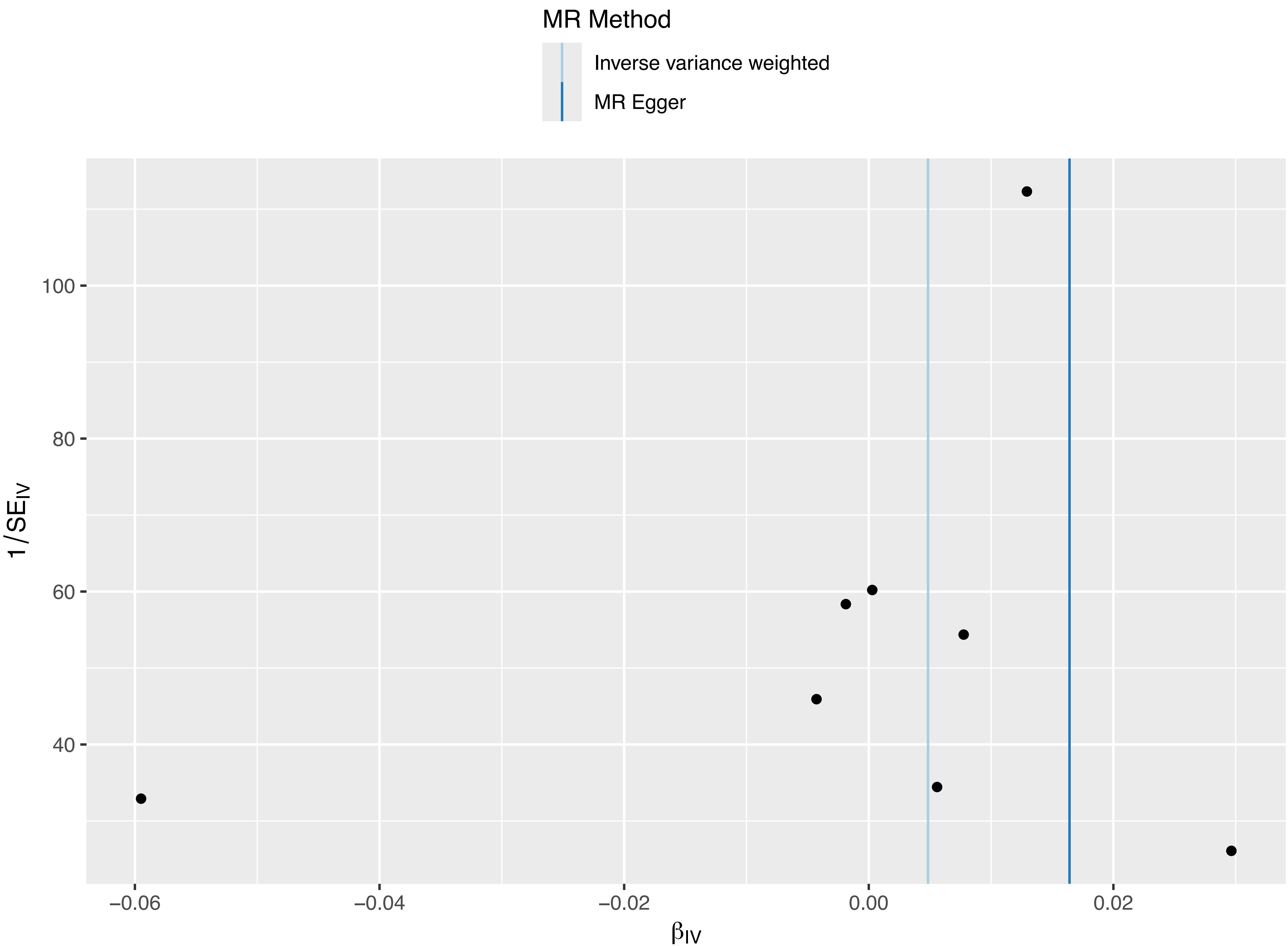
MR Method
Inverse variance weighted
MR Egger
100
80
60
40
1/SE_IV
−0.06
−0.04
−0.02
0.00
0.02
β_IV

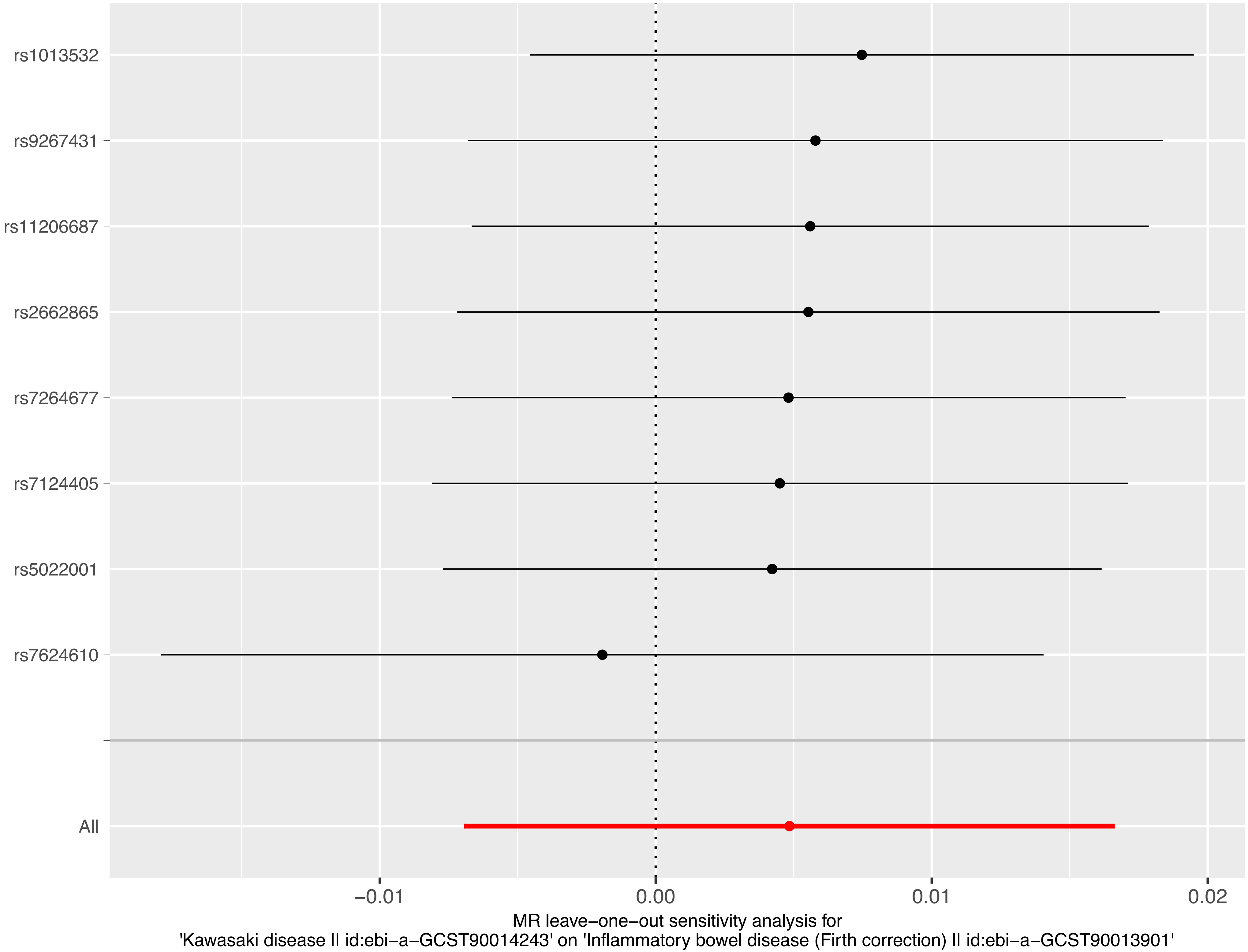
rs1013532
rs9267431
rs11206687
rs2662865
rs7264677
rs7124405
rs5022001
rs7624610
All
−0.01
0.00
0.01
0.02
MR leave−one−out sensitivity analysis for
'Kawasaki disease || id:ebi−a−GCST90014243' on 'Inflammatory bowel disease (Firth correction) || id:ebi−a−GCST90013901'

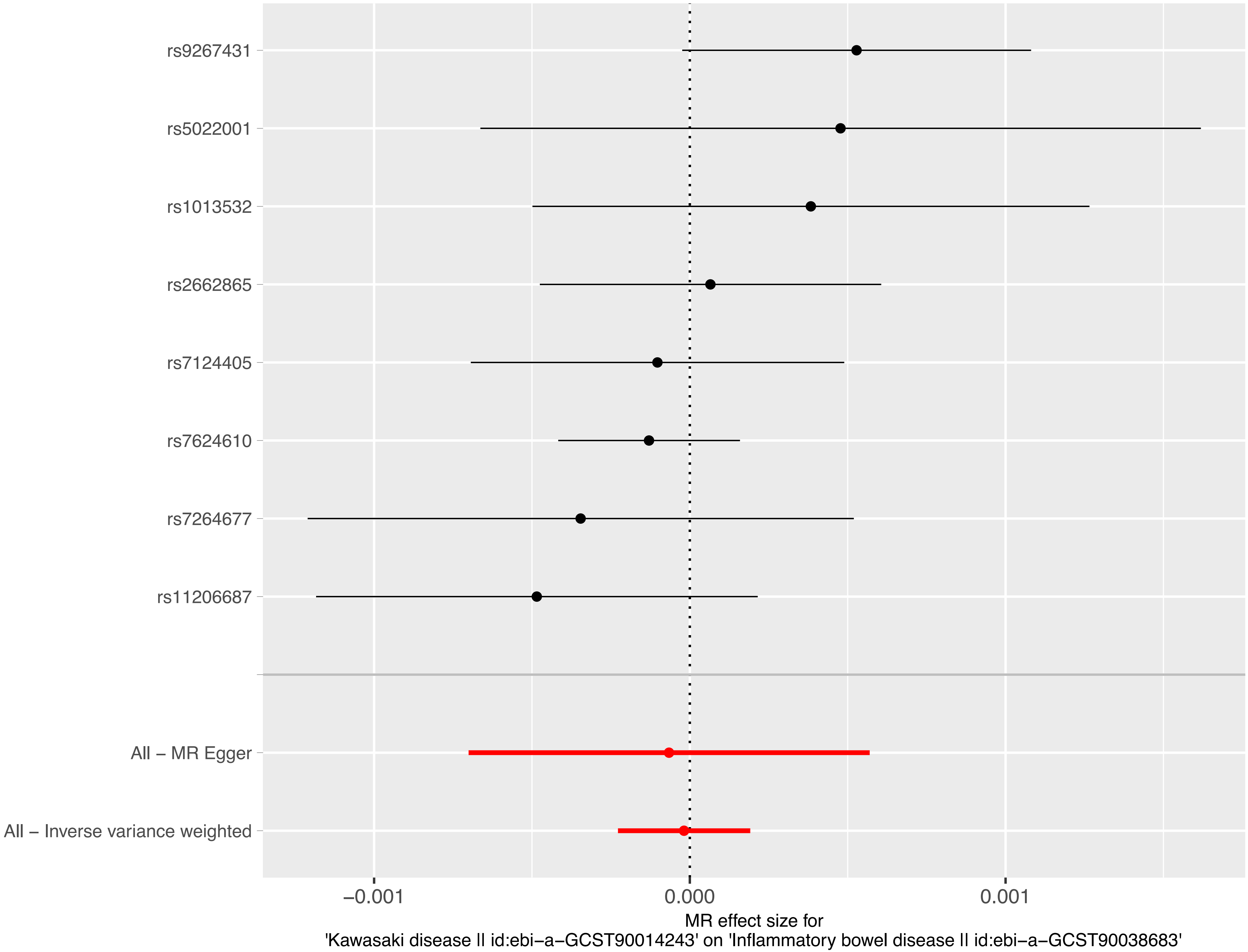
rs9267431
rs5022001
rs1013532
rs2662865
rs7124405
rs7624610
rs7264677
rs11206687
All – MR Egger
All – Inverse variance weighted
−0.001
0.000
0.001
MR effect size for
'Kawasaki disease || id:ebi−a−GCST90014243' on 'Inflammatory bowel disease || id:ebi−a−GCST90038683'

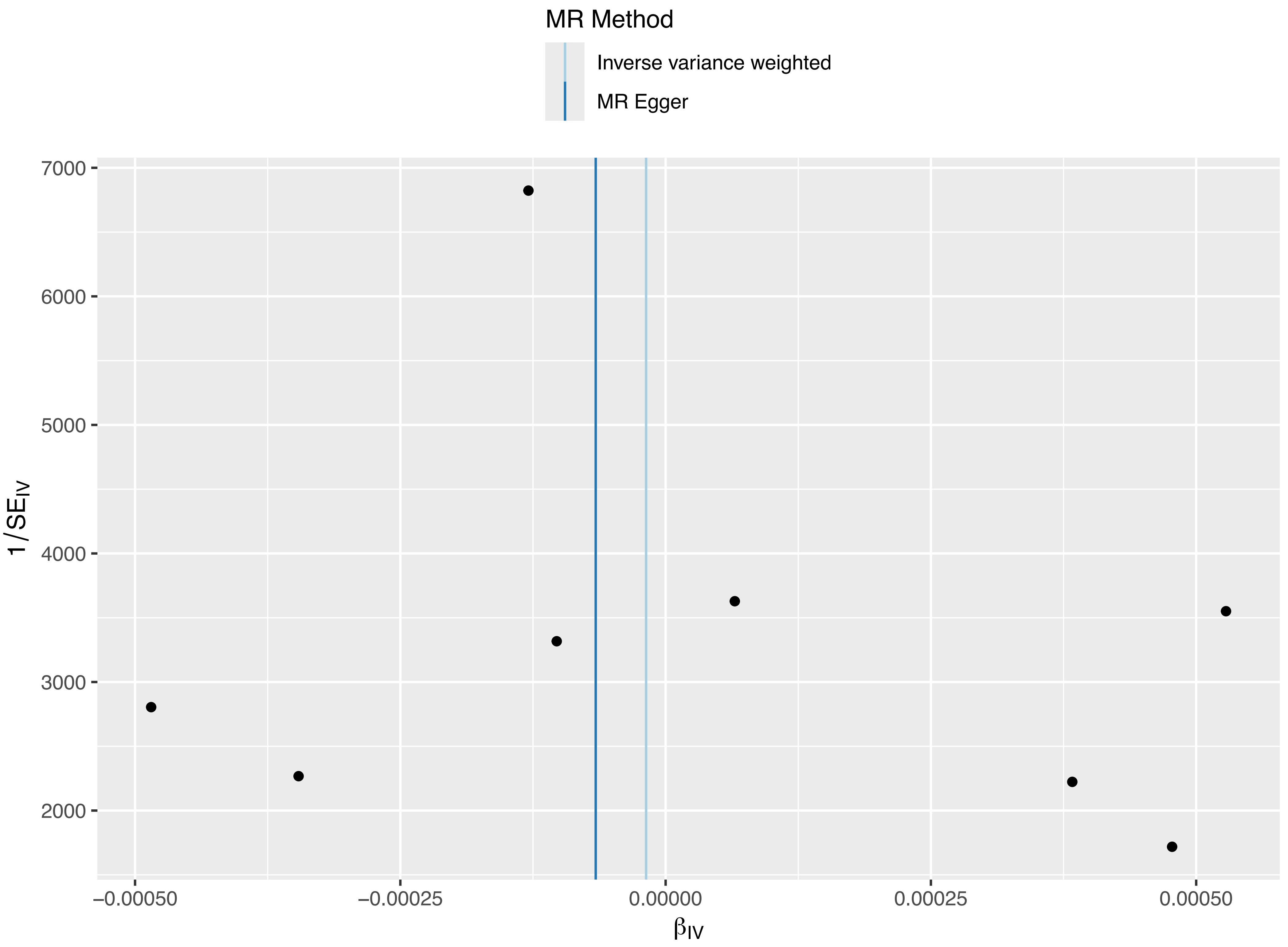

MR Method
Inverse variance weighted
MR Egger
7000
6000
5000
4000
3000
2000
1/SE_IV
−0.00050
−0.00025
0.00000
0.00025
0.00050
β_IV

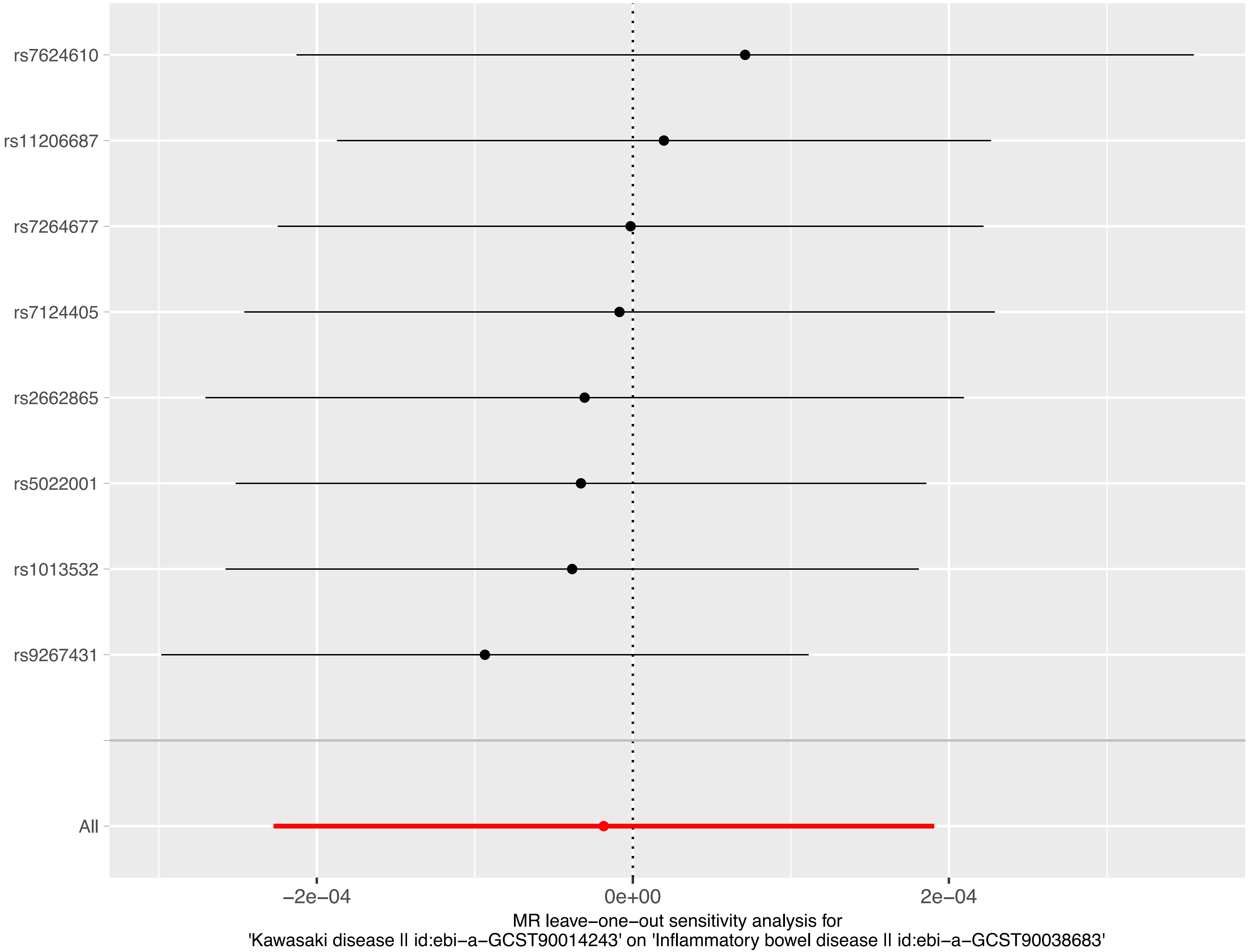
rs7624610
rs11206687
rs7264677
rs7124405
rs2662865
rs5022001
rs1013532
rs9267431
All
−2e−04
0e+00
2e−04
MR leave−one−out sensitivity analysis for
'Kawasaki disease || id:ebi−a−GCST90014243' on 'Inflammatory bowel disease || id:ebi−a−GCST90038683'

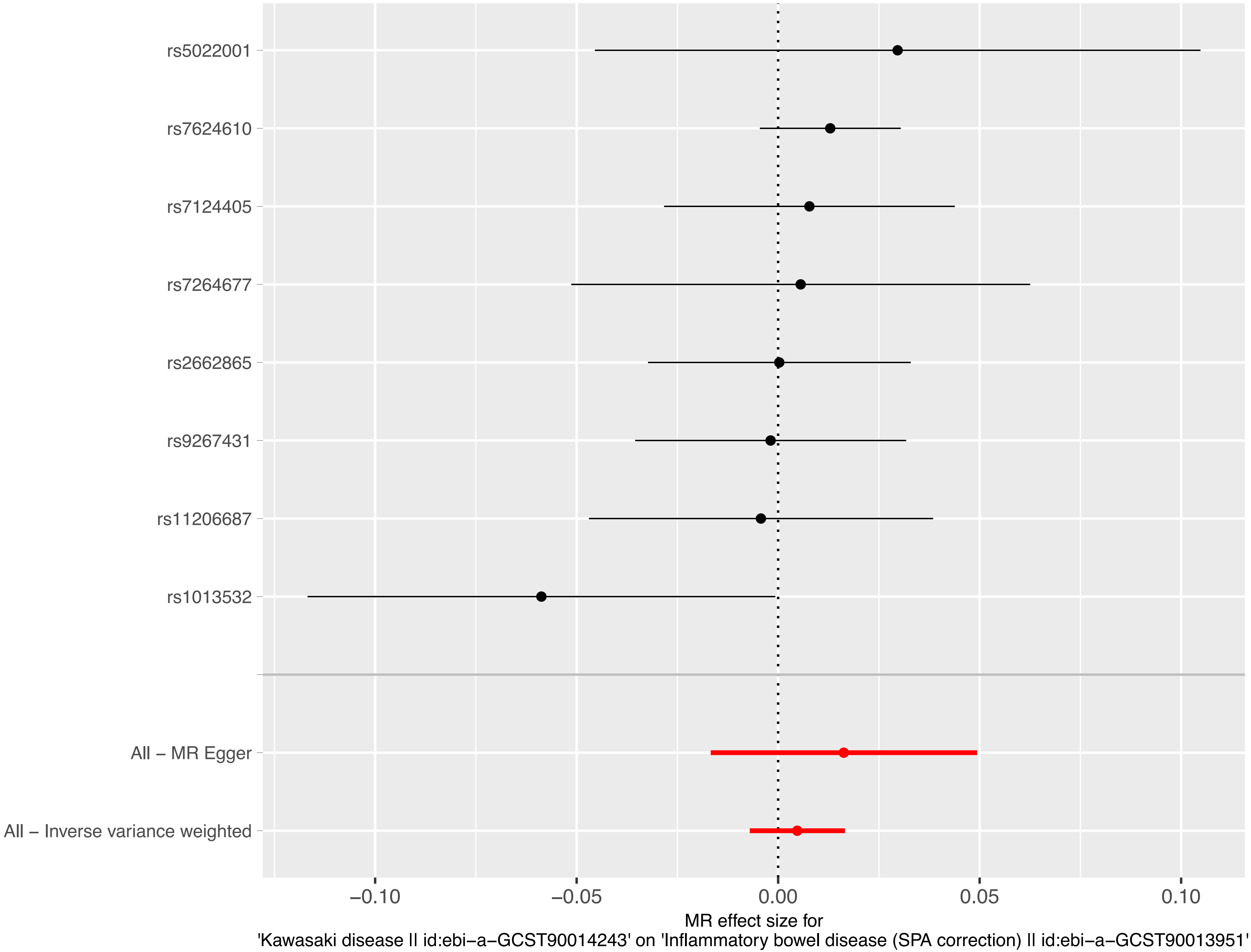
rs5022001
rs7624610
rs7124405
rs7264677
rs2662865
rs9267431
rs11206687
rs1013532
All – MR Egger
All – Inverse variance weighted
−0.10
−0.05
0.00
0.05
0.10
MR effect size for
'Kawasaki disease || id:ebi−a−GCST90014243' on 'Inflammatory bowel disease (SPA correction) || id:ebi−a−GCST90013951'

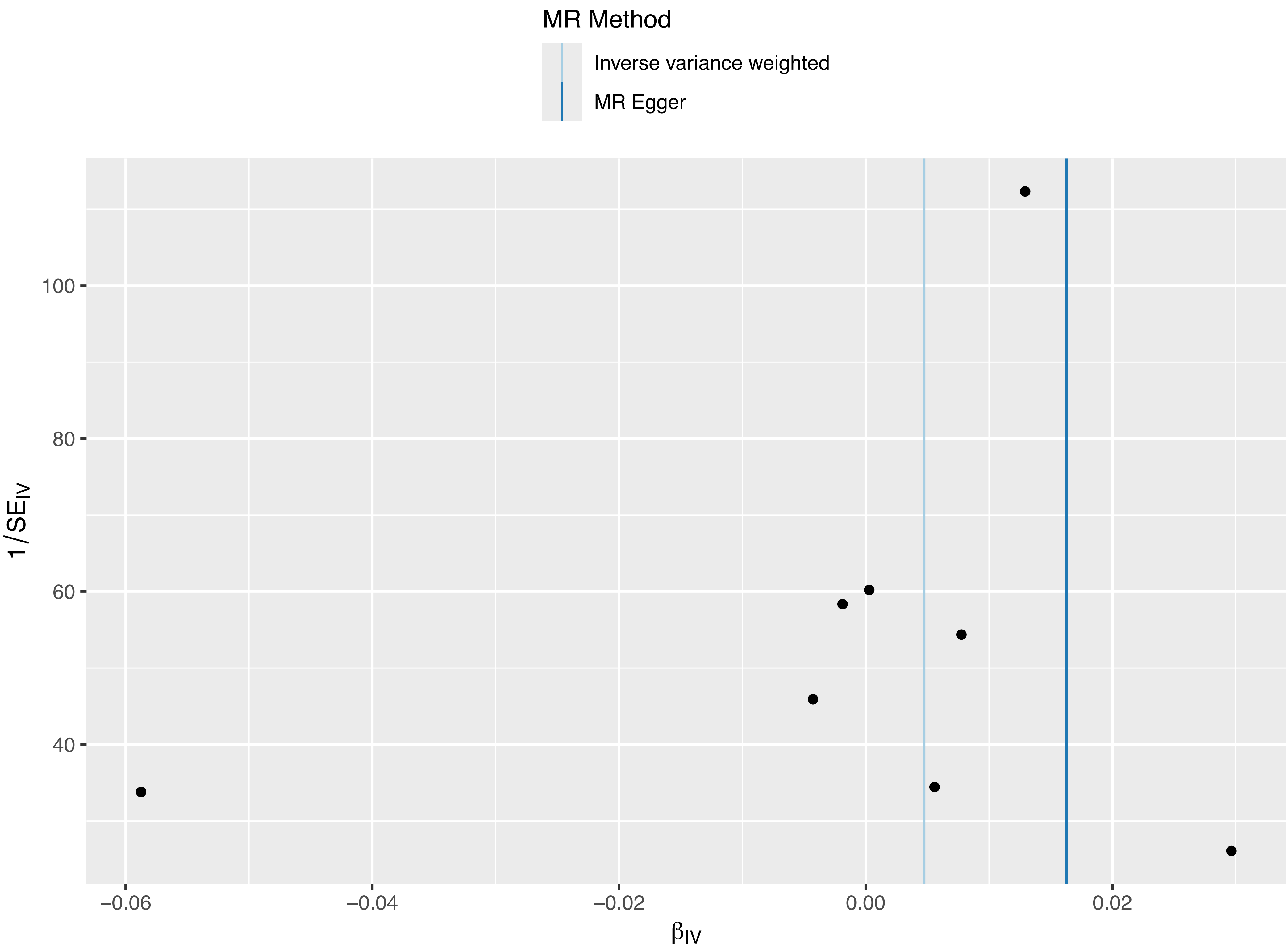
MR Method
Inverse variance weighted
MR Egger
1/SE$_{IV}$
β_{IV}
100
80
60
40
−0.06
−0.04
−0.02
0.00
0.02

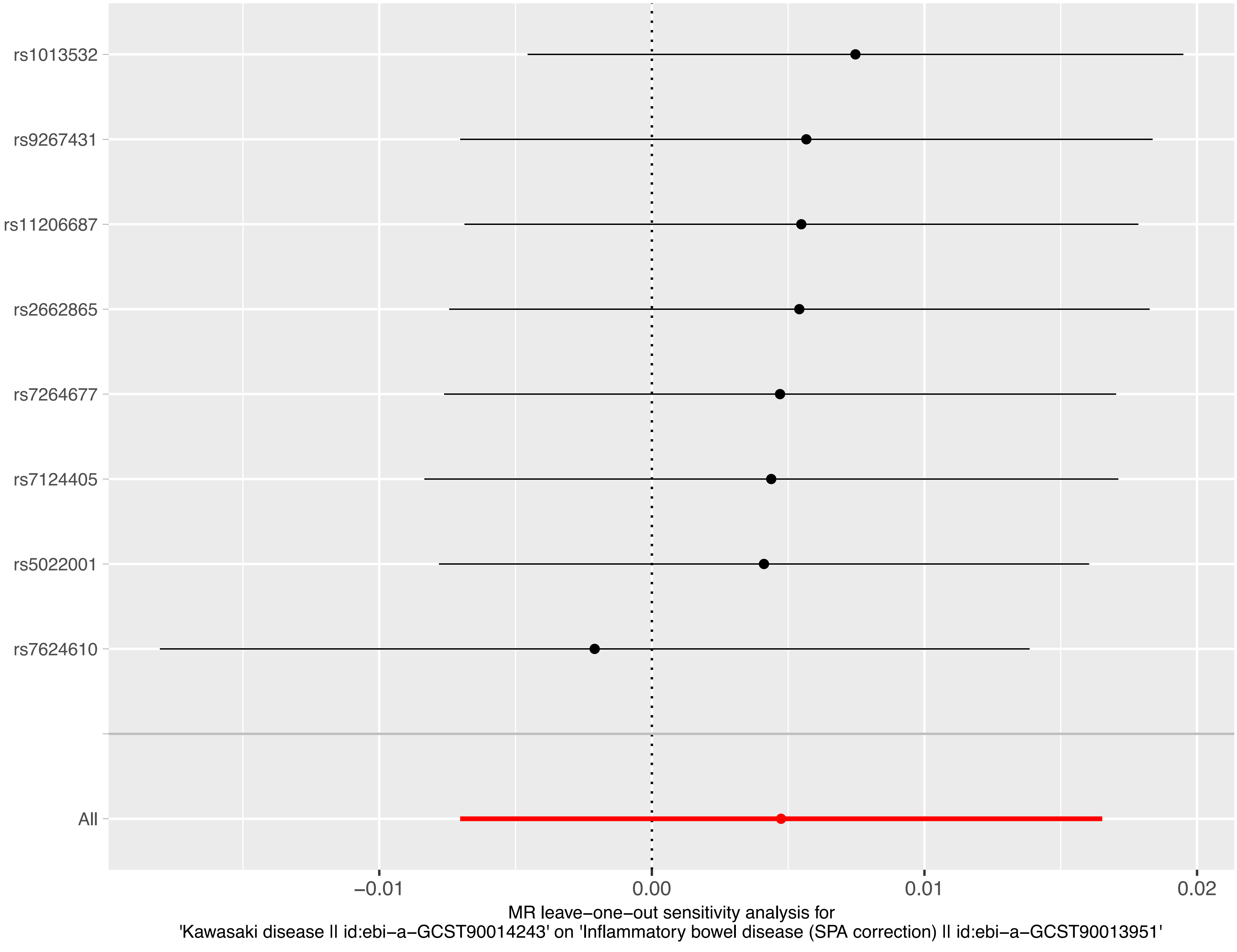
rs1013532
rs9267431
rs11206687
rs2662865
rs7264677
rs7124405
rs5022001
rs7624610
All
−0.01
0.00
0.01
0.02
MR leave−one−out sensitivity analysis for
'Kawasaki disease || id:ebi−a−GCST90014243' on 'Inflammatory bowel disease (SPA correction) || id:ebi−a−GCST90013951'

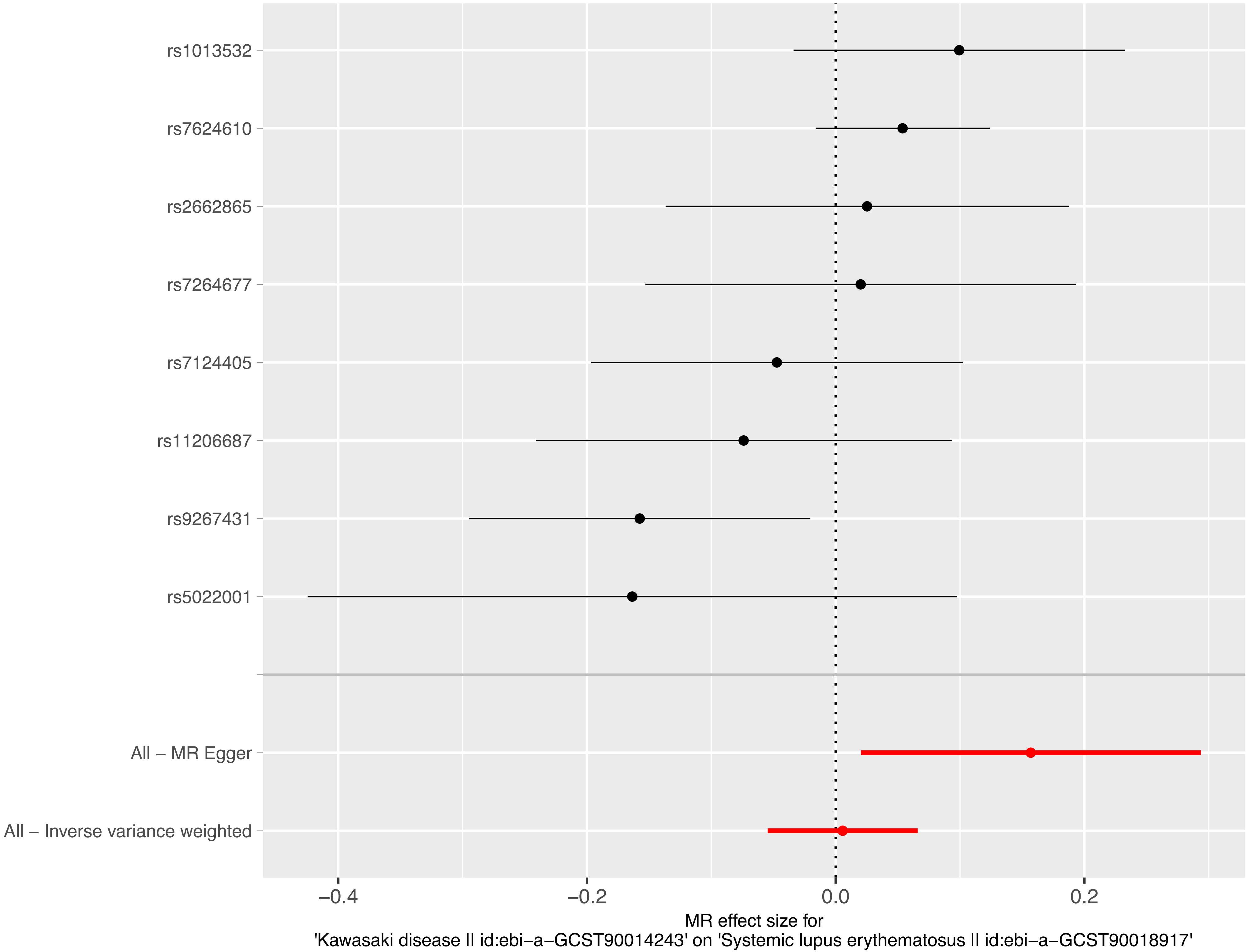

rs1013532
rs7624610
rs2662865
rs7264677
rs7124405
rs11206687
rs9267431
rs5022001
All – MR Egger
All – Inverse variance weighted
−0.4
−0.2
0.0
0.2
MR effect size for
'Kawasaki disease || id:ebi–a–GCST90014243' on 'Systemic lupus erythematosus || id:ebi–a–GCST90018917'

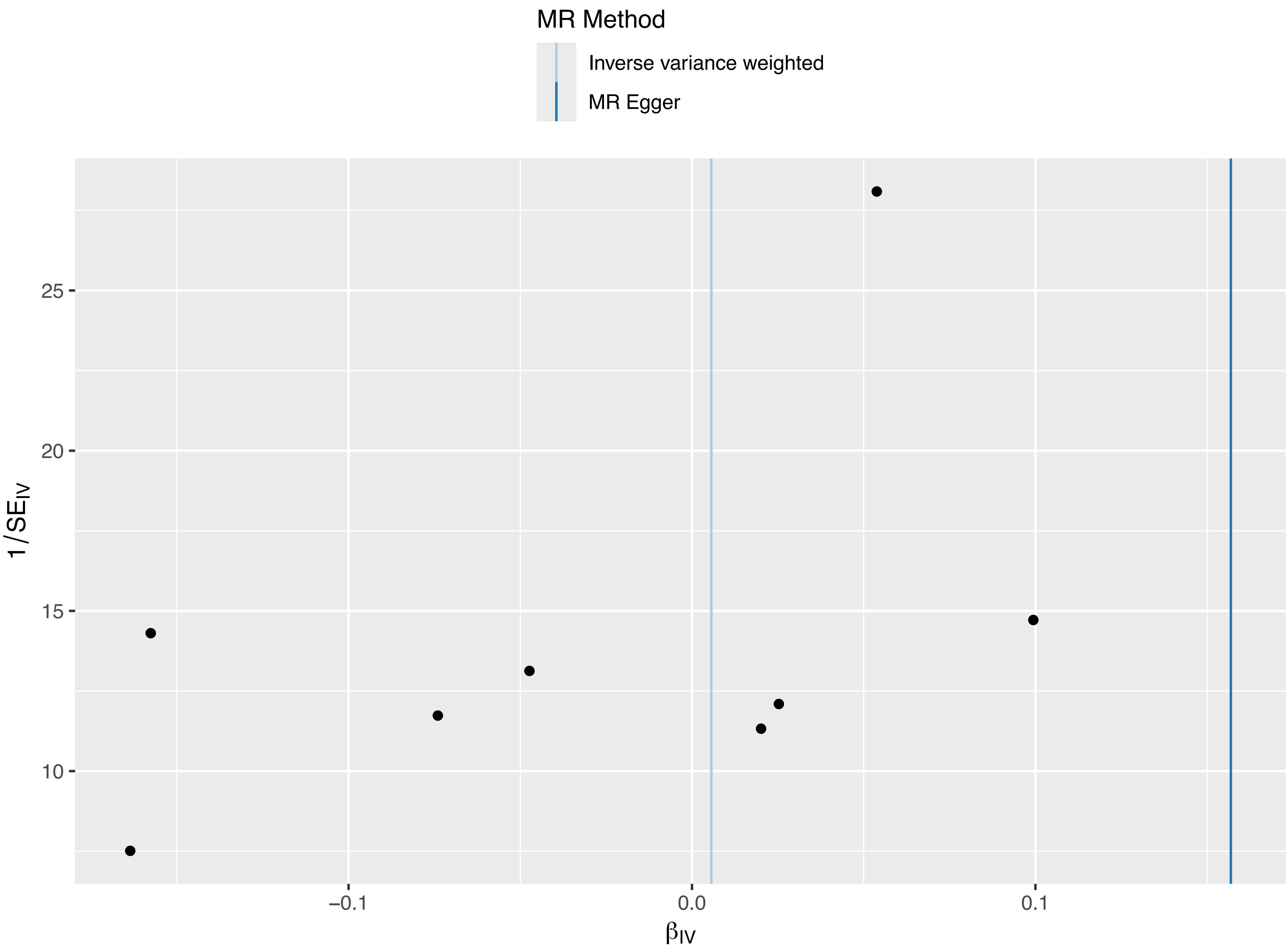
MR Method
Inverse variance weighted
MR Egger
25
20
15
10
1/SE$_{IV}$
−0.1
0.0
0.1
β_{IV}

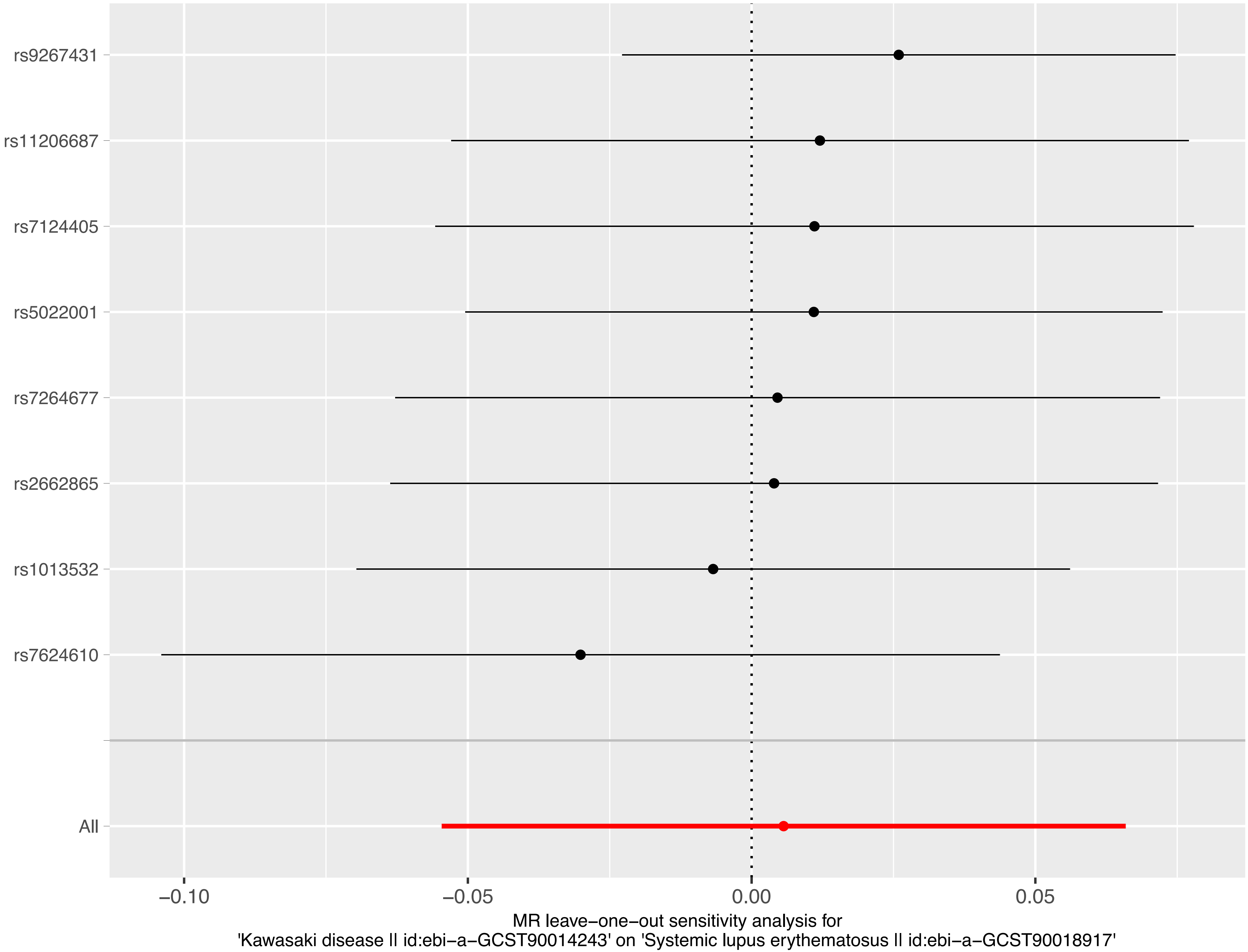

rs9267431
rs11206687
rs7124405
rs5022001
rs7264677
rs2662865
rs1013532
rs7624610
All
−0.10
−0.05
0.00
0.05
MR leave−one−out sensitivity analysis for
'Kawasaki disease || id:ebi−a−GCST90014243' on 'Systemic lupus erythematosus || id:ebi−a−GCST90018917'

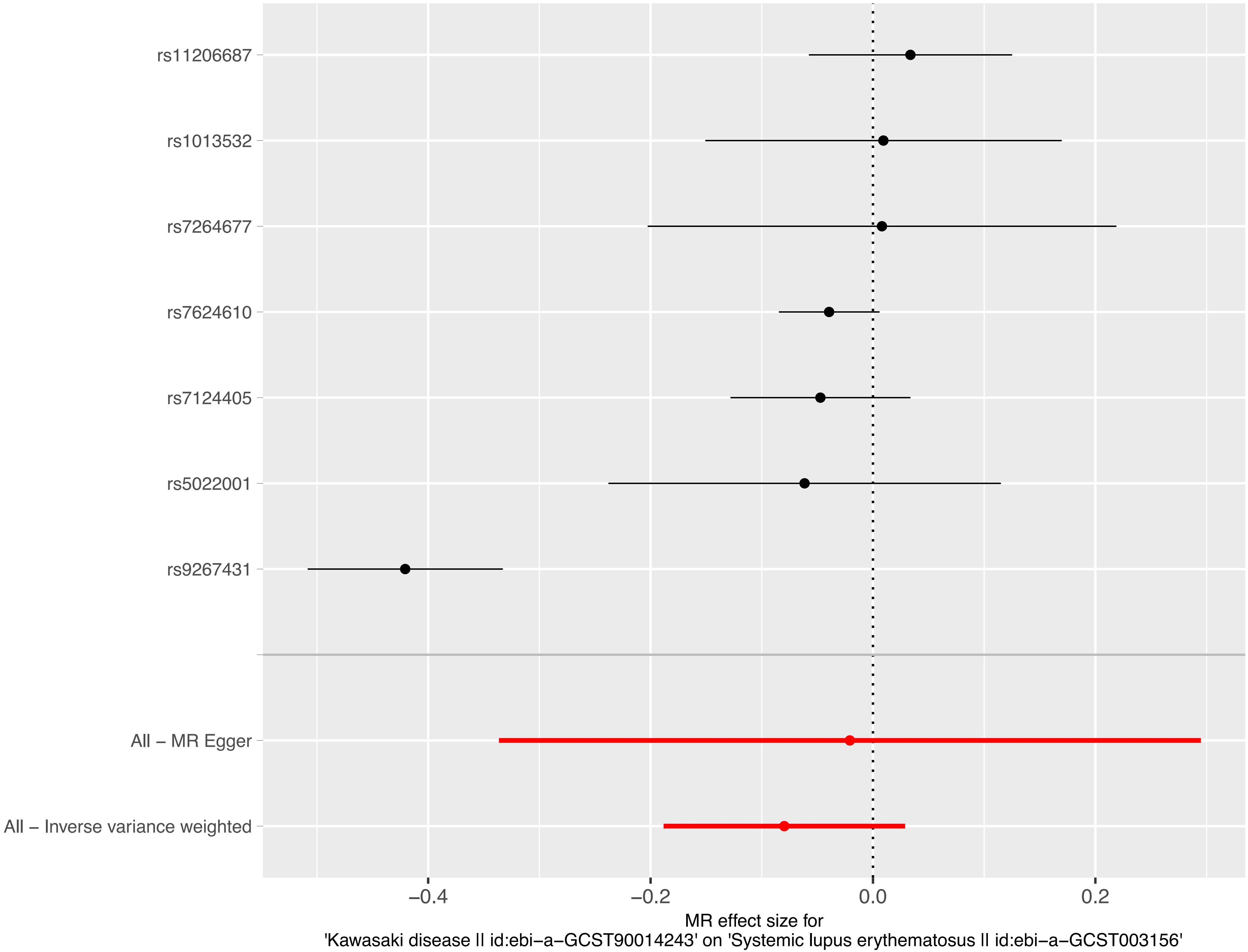
rs11206687
rs1013532
rs7264677
rs7624610
rs7124405
rs5022001
rs9267431
All – MR Egger
All – Inverse variance weighted
−0.4
−0.2
0.0
0.2
MR effect size for
'Kawasaki disease || id:ebi−a−GCST90014243' on 'Systemic lupus erythematosus || id:ebi−a−GCST003156'

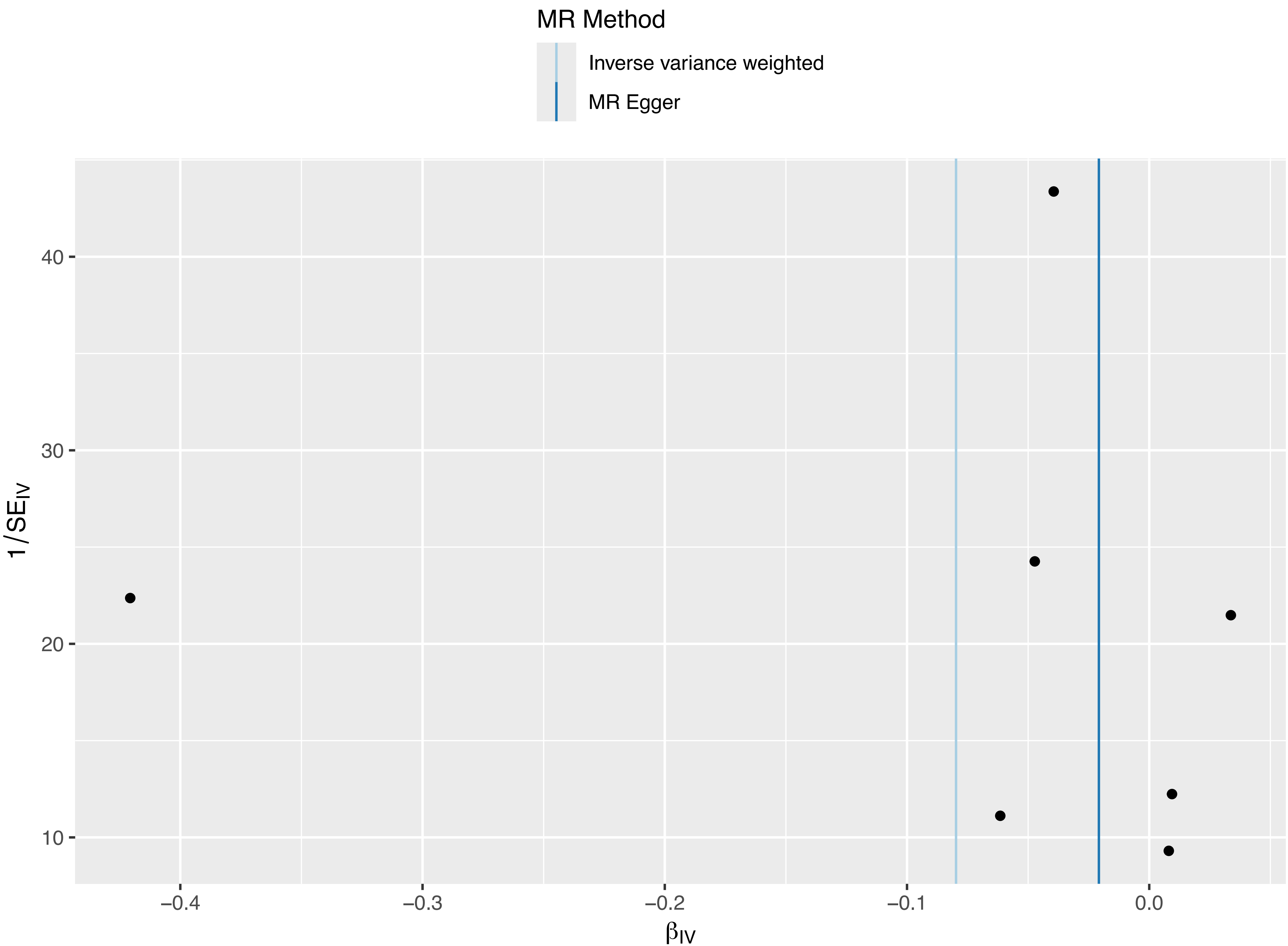

MR Method
Inverse variance weighted
MR Egger
40
30
20
10
1/SE_IV
−0.4
−0.3
−0.2
−0.1
0.0
β_IV

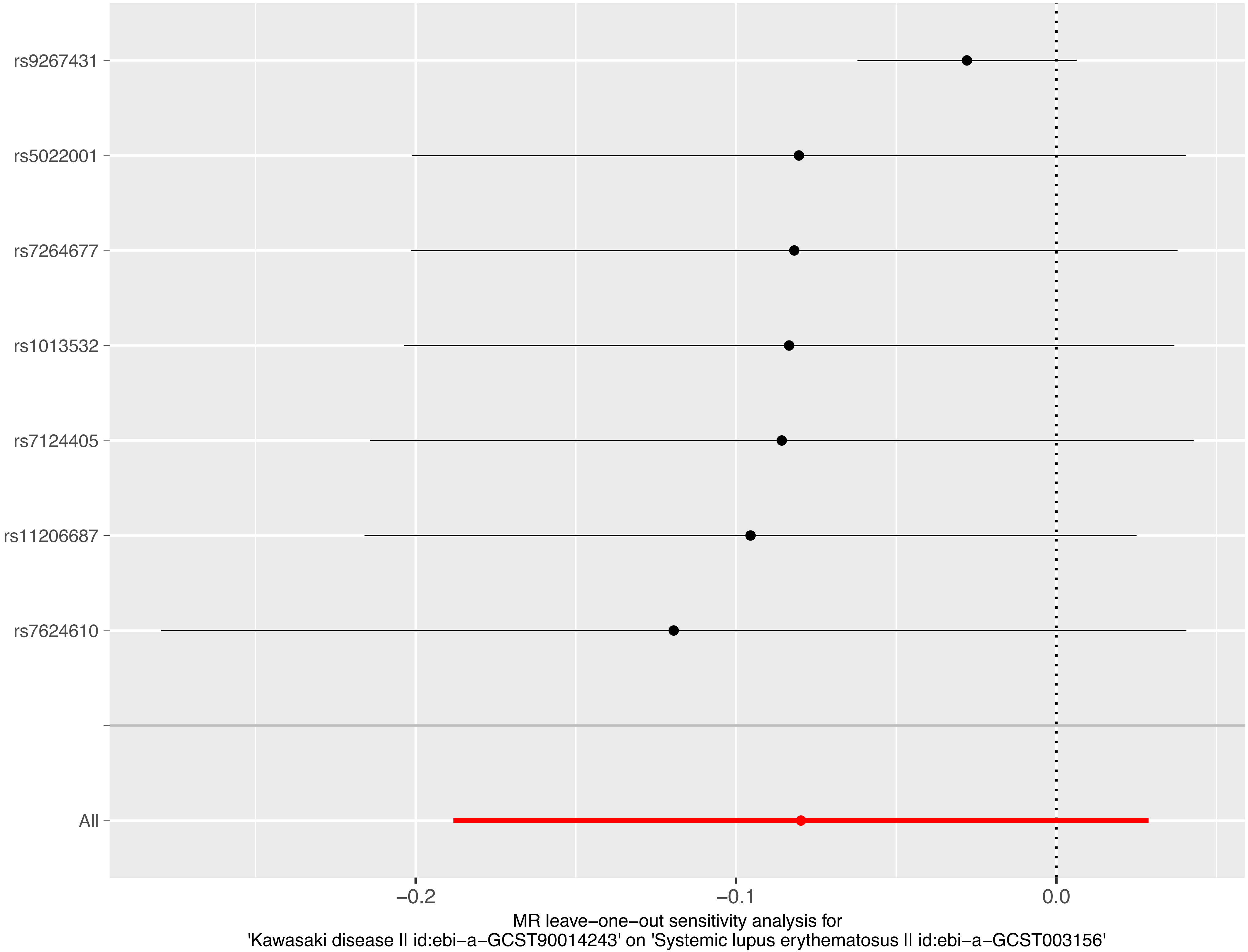

rs9267431
rs5022001
rs7264677
rs1013532
rs7124405
rs11206687
rs7624610
All
−0.2
−0.1
0.0
MR leave−one−out sensitivity analysis for
'Kawasaki disease || id:ebi−a−GCST90014243' on 'Systemic lupus erythematosus || id:ebi−a−GCST003156'

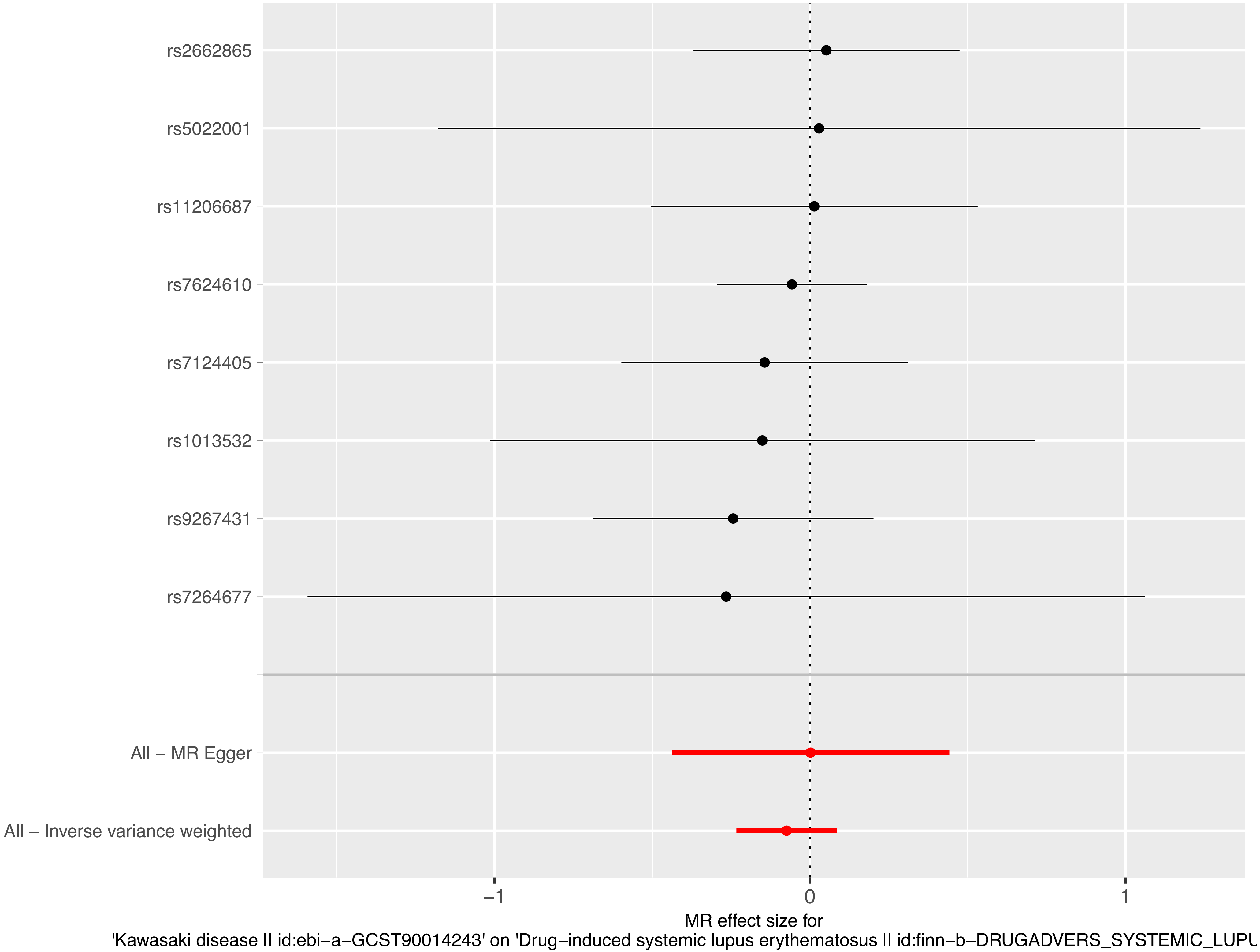
rs2662865
rs5022001
rs11206687
rs7624610
rs7124405
rs1013532
rs9267431
rs7264677
All – MR Egger
All – Inverse variance weighted
−1
0
1
MR effect size for
'Kawasaki disease || id:ebi−a−GCST90014243' on 'Drug−induced systemic lupus erythematosus || id:finn−b−DRUGADVERS_SYSTEMIC_LUPU

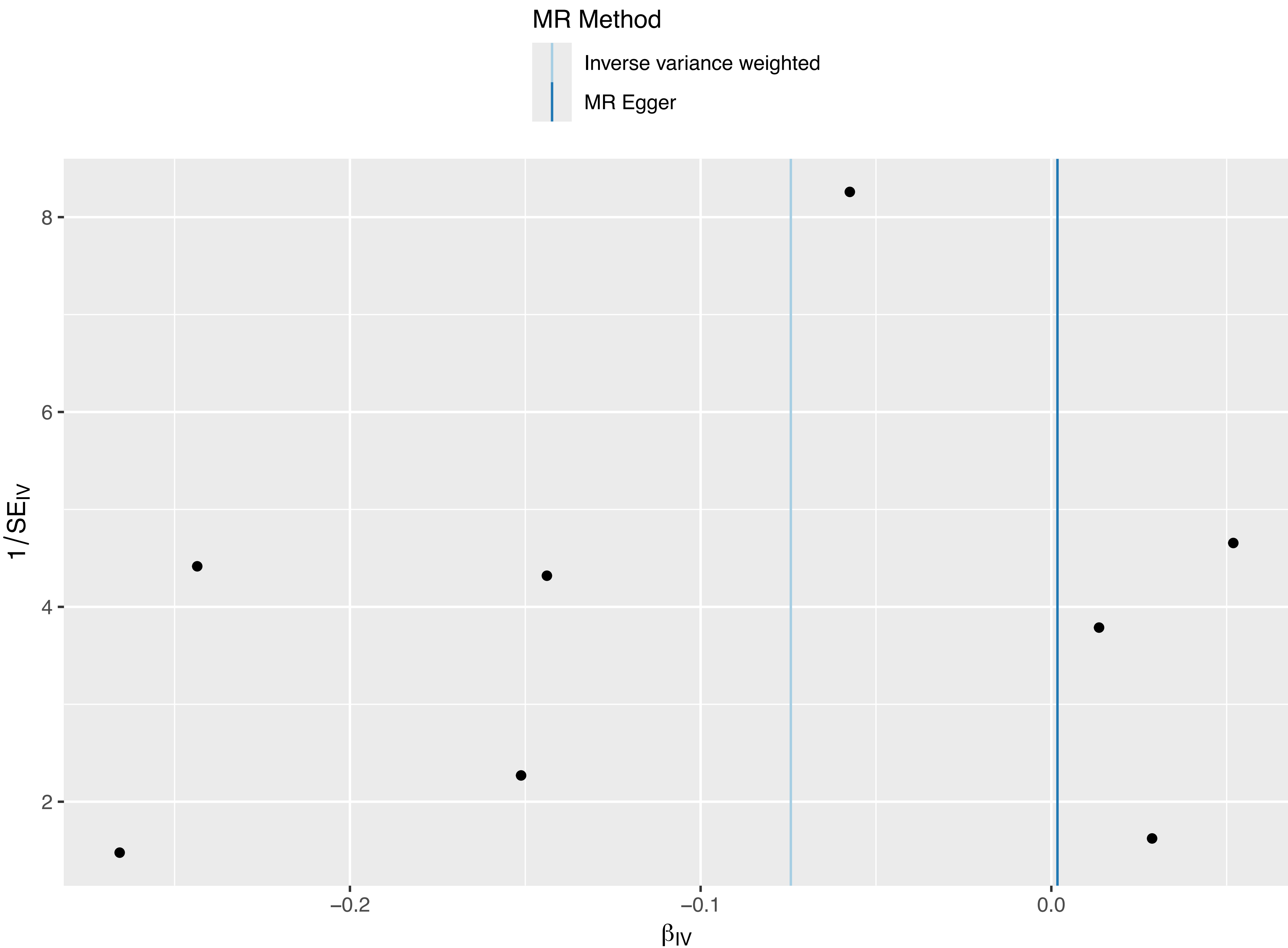
MR Method
Inverse variance weighted
MR Egger
1/SE_IV
β_IV
8
6
4
2
−0.2
−0.1
0.0

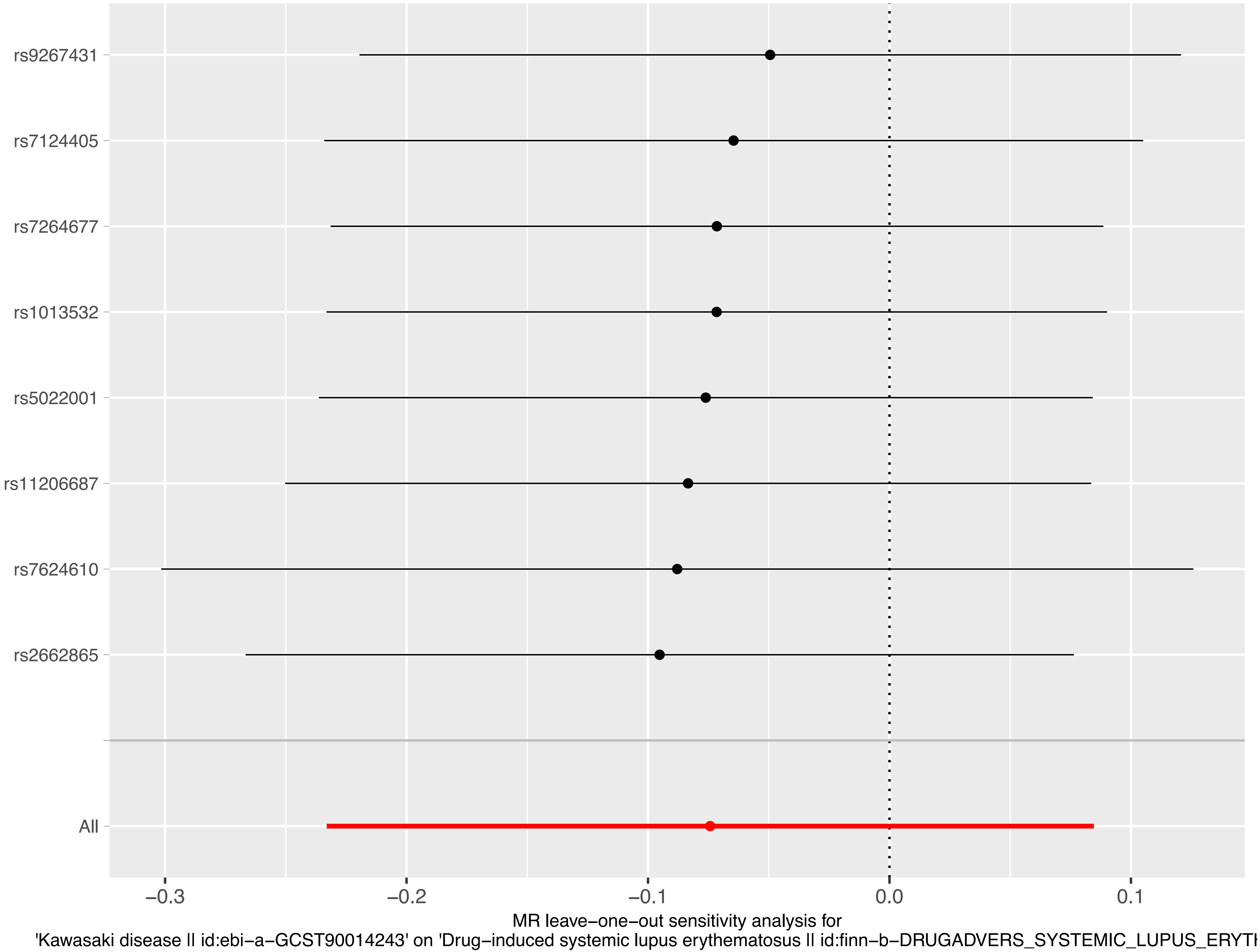
rs9267431
rs7124405
rs7264677
rs1013532
rs5022001
rs11206687
rs7624610
rs2662865
All
−0.3
−0.2
−0.1
0.0
0.1
MR leave−one−out sensitivity analysis for
'Kawasaki disease || id:ebi−a−GCST90014243' on 'Drug−induced systemic lupus erythematosus || id:finn−b−DRUGADVERS_SYSTEMIC_LUPUS_ERYTH
